# Bioinformatic Prediction and Characterization of Proteins in *Porphyra dentata* by Shotgun Proteomics

**DOI:** 10.3389/fnut.2022.924524

**Published:** 2022-07-07

**Authors:** Mingchang Yang, Lizhen Ma, Xianqing Yang, Laihao Li, Shengjun Chen, Bo Qi, Yueqi Wang, Chunsheng Li, Shaoling Yang, Yongqiang Zhao

**Affiliations:** ^1^Key Laboratory of Aquatic Product Processing, Ministry of Agriculture and Rural Affairs, National R&D Center for Aquatic Product Processing, South China Sea Fisheries Research Institute, Chinese Academy of Fishery Sciences, Guangzhou, China; ^2^College of Food Science and Bioengineering, Tianjin Agricultural University, Tianjin, China; ^3^Co-innovation Center of Jiangsu Marine Bio-Industry Technology, Jiangsu Ocean University, Lianyungang, China; ^4^Collaborative Innovation Center of Seafood Deep Processing, Dalian Polytechnic University, Dalian, China

**Keywords:** *Porphyra dentata*, proteomics, amino acid metabolism, carbohydrate metabolism, bioactive peptides

## Abstract

*Porphyra dentata* is an edible red seaweed with high nutritional value. It is widely cultivated and consumed in East Asia and has vast economic benefits. Studies have found that *P. dentata* is rich in bioactive substances and is a potential natural resource. In this study, label-free shotgun proteomics was first applied to identify and characterize different harvest proteins in *P. dentata.* A total of 13,046 different peptides were identified and 419 co-expression target proteins were characterized. Bioinformatics was used to study protein characteristics, functional expression, and interaction of two important functional annotations, amino acid, and carbohydrate metabolism. Potential bioactive peptides, protein structure, and potential ligand conformations were predicted, and the results suggest that bioactive peptides may be utilized as high-quality active fermentation substances and potential targets for drug production. Our research integrated the global protein database, the first time bioinformatic analysis of the *P. dentata* proteome during different harvest periods, improves the information database construction and provides a framework for future research based on a comprehensive understanding.

## Introduction

As an excellent source of bioactive substances, marine algae possess unique developmental potential owing to the diversity of natural products (1). *Porphyra* processing, utilization, and health promotion are representative of red algae research; they are mainly distributed in the intertidal and subtidal zones of the ocean. Moreover, they are large, ancient, and rich in protein, polyunsaturated fatty acids, β*-*carotene, polyphenols, and polysaccharides. They also possess antibacterial, anti-inflammatory, antioxidant, and anti-tumor properties (2–4). With the development of modern biochemistry and molecular biotechnology, *Porphyra* has been used to develop numerous resources, including drugs, food, cosmetics, bioactive products, etc (5). As a staple food and drug resource, *Porphyra dentata* (*P. dentata*) is widely cultivated in East Asia. The total production output of China, Japan, and South Korea has reached more than 95% of the world (6, 7). It also contains numerous amino acids and carbohydrates and is considered a high-quality food supplement and dietary source (8–10).

In *P. dentata*, amino acids and carbohydrate quality are the criteria for nutrient value of intensive processing. In previous studies, plants were subjected to abiotic stress during growth, and produce molecular metabolites with biological functions and health promotion characteristics. Amino acid and carbohydrate metabolism protein can be used as potential active health components. They are not only pivotal to the expression of structural composition and functional characteristics but also are important reactants of plant life activities (11–13). *Porphyra* proteins and their bioactive peptides play an important role in the metabolic function of organisms and human health. They have dietary and pharmaceutical activities and can promote the regulation of body function (14). Although correlations between structural and functional properties have not been fully determined, the use of bioinformatic characterizations and bioactive peptide databases such as APD3 and BIOPepDB can improve the analysis and exploration of the physical and chemical properties of bioactive peptides (15, 16). But the harvest period and growing cycle of *P. dentata* remain difficult and uncertain for development, utilization, and intensive processing (17).

Omics analysis is used to obtain unique nutrients and potential health promotion functions (18). Proteomics is a powerful approach used for protein identification and analysis in *Porphyra* (19, 20). In the label-free proteomics method, a mixture of protein samples was digested by proteases (trypsin) and quantitative information from peptides is obtained and analyzed by liquid chromatography-tandem mass spectrometry (LC-MS/MS). Then, the peptide sequences are loaded into a database program to search and identify matching proteins (21). Label-free proteomics is widely used to predict and identify unknown proteins and peptides with complete matching numbers. This method has high sensitivity and wide application and is suitable for analyzing algal research with small molecular weight proteins (22). In a recent study, the label-free proteomic analysis revealed specific changes in muscle proteins in pike eel (*Muraenesox cinereus*) under cold stress and freshness-related proteins in sea bass (*Lateolabrax japonicus*) filets, during cold storage; however, no reports are available on protein changes at different harvest periods in seaweeds (23, 24).

This is the first study employing label-free shotgun proteomics to comprehensively identify the proteome of *P. dentata* during different harvest periods. Moreover, different bioinformatics program repositories were used to characterize amino acid and carbohydrate metabolism protein in first and fifth harvest co-expression, and explored potential bioactive peptides, providing new understanding in the study of *P. dentata*.

## Materials and Methods

### Chemicals and Reagents

Iodoacetamide (IAM), and triethylammonium bicarbonate buffer (TEAB) were purchased from Sigma (St. Louis, MO, United States). Sodium dodecyl sulfate (SDS) and acetone were obtained from Sinopharm Chemical Reagent (Shanghai, China). Coomassie brilliant blue staining solution (Beyotime, China) and sequencing modified trypsin (mass spectrometry grade) were sourced from Promega (Madison, WI, United States). Oasis^®^ HLB 96-Well Plate 30 μm and Oasis^®^ MCX μ Elution Plate 30 μm were from Waters (Milford, MA, United States). Protein ladder, Pierce™ BCA (bicinchoninic acid) protein assay kit, Halt™ Protease inhibitor cocktail, NUPAGE™ 12% BT GEL 1.0MM 12W, Bond-Breaker TCEP solution [Tris(2-carboxyethyl) phosphine hydrochloride], Pierce quantitative colorimetric peptide assay were obtained from Thermo Fisher Scientific (United States). All other chemicals were reagent/analytical grade.

### *Porphyra dentata* Samples

First, third, and fifth harvests of *P. dentata* were obtained from Shen’ao Bay (23.46°N, 117.09°E), Nan’ao island, Shantou City, Guangdong Province, China, in December 2020, with each harvest having two biological replicates. After collection, they were cleaned with sterilized water to remove sediment, appressorium, and other impurities, placed in sterile sealed tubes, and stored in a −80°C ultra-low temperature freezer after transportation by a liquid nitrogen container.

### Total Protein Extraction and Sodium Dodecyl Sulfate-Polyacrylamide Gel Electrophoresis

The samples were ground in liquid nitrogen and BPP [borax, polyvinylpolypyrroli-done (PVPP), and phenol] mixed in a 1:10 ratio. The suspensions were centrifuged at 12,000 × *g* and 4°C for 20 min and the supernatants were collected. An equal volume of Tris-saturated phenol was added to each supernatant and each mixture was vortexed at 4°C for 10 min. The mixtures were centrifuged at 12,000 × *g* and 4°C for 20 min and the phenol phases were collected. An equal volume of BPP was added to each supernatant and the mixtures were vortexed at 4°C for 10 min. The solutions were centrifuged at 12,000 × *g* and 4°C for 20 min and the phenol phases were collected. Five times volumes of pre-cooled 0.1M ammonium acetate in methanol were added and the proteins were precipitated at −20°C overnight. The supernatants were discarded by centrifugation and the precipitates were washed twice with 90% (*v/v*) acetone. The supernatants were discarded by centrifugation and the precipitates were air-dried. The precipitates were re-suspended in lysis buffer [1% (*v/v*) SDS plus 8M urea] and sonicated on ice for 3 min. The lysates were centrifuged, and the supernatants were collected to determine the protein content in a BCA Protein Assay Kit (Thermo Fisher Scientific, United States).

The NuPAGE™ 12% BIS-Tris Protein Gels electrophoresis gel (Thermo Fisher Scientific, United States) was removed from the packaging and bottom seal and fixed in the electrophoresis tank. Sodium dodecyl sulfate-polyacrylamide gel electrophoresis (SDS-PAGE) electrophoresis buffer (1×) was added. The samples were prepared by mixing 4 μL of protein sample and 1 μL of sample loading buffer solution. After fully mixing, the samples were heated at 100°C for 3–5 min. After the protein was fully denatured, the samples were loaded, and the voltage was set to 200 V for 45 min. The results were observed and photographed by an automatic digital gel image analyzer.

### Protein Digestion, Peptide Desalination, and Quantification

Protein digestion was performed according to the standard procedure. Briefly, for each sample tube containing 150 μg protein, appropriate TCEP was added to achieve a final concentration of 10 mol/L and the tubes were incubated at 37°C for 60 min. Appropriate IAM was added to the final concentration of 40 mM and reaction for 40 min in dark. Six volumes of cold acetone as added to the sample tube. The tube was inverted three times and incubated at −20°C until a precipitate formed, which was approximately 4 h. The acetone was removed by centrifugation at 10,000 *g* for 20 min and the precipitated protein was resuspended with 150 μL 100 mol/L TEAB buffer. To each sample tube, a 1:50 proportion ratio of sample to trypsin solution was added and incubated at 37°C overnight.

The peptides were vacuum-dried and resuspended in 2% (*v/v*) acetonitrile and 0.1% (*v/v*) trifluoroacetic acid (TF). The samples were desalted with Sep-Pak and vacuum-dried. The peptide concentrations were determined using a Peptide Quantification Kit (Thermo Fisher Scientific, United States). Loading buffer was added to each tube to prepare the samples for mass spectrometry (MS) analysis. The concentration of each sample was 0.5 μg/μL.

### Shotgun Liquid Chromatography-Tandem Mass Spectrometry Analysis

Peptides were dissolved using a mass spectrometry loading buffer and LC-MS/MS analysis was performed. The peptide samples were separated by the EASY-nLC 1200 liquid system, and the chromatographic column was a C_18_ column (75 μm × 25 cm, Thermo, United States). Mobile phase A was 2% acetonitrile and 0.1% formic acid, and mobile phase B was 80% acetonitrile and 0.1% formic acid. The separation gradient was 0–2 min and mobile phase B increased linearly from 0 to 6%; when the separation gradient was 2–105 min, mobile phase B linearly increased from 6 to 23%; when the separation gradient was 105–130 min, mobile phase B linearly increased from 23 to 29%; when the separation gradient was 130–147 min, mobile phase B linearly increased from 29 to 38%; when the separation gradient was 147–148 min, mobile phase B linearly increased from 38 to 48%; when the separation gradient was 148–149 min, mobile phase B linearly increased from 48 to 100%; when the separation gradient was 149–155 min, mobile phase B linearly was maintained at 100%. Using Q-Exactive HF-X (Thermo, United States) for mass spectrometry analysis, MS scanning range was 350–1 300 (m/z), acquisition mode was DDA, fragmentation mode was HCD, resolution of the primary mass spectrometer was 70,000, and resolution of secondary was 17,500. A total of four replicates (*n* = 4) were analyzed independently.

### Sequence Database Searching

Using Proteome Discoverer™ Software 2.2 (Thermo Fisher Scientific, United States) (25), MS/MS spectra were searched against the database. The highest score for a given peptide mass (best match to that predicted in the database) was used to identify parent proteins. The parameters for protein searches were set as follows: tryptic digestion with up to two missed cleavages, carbamidomethylation of cysteine as a fixed modification, and oxidation of methionine and protein N-terminal acetylation as variable modifications. Peptide spectral matches were validated based on *q*-values at a 1% false discovery rate (FDR).

### Bioinformatics Analysis

The gene ontology (GO) database^1^ and Kyoto Encyclopedia of Genes and Genomes (KEGG) pathway database^2^ were used to perform GO function annotations and participate in metabolic pathway analysis of proteins to obtain information about biological functions, biological processes involved, cell location, functional domain analysis using Pfam^3^ position (14).

### Network Analysis and Sequence Similarities

Network analysis was performed by submitting the protein dataset to STRING (Search Tool for the Retrieval of Interacting Genes) software (version 11.5).^4^ This database is known and used to predict protein interactions. Proteins were represented with nodes and their interactions with continuous lines to represent direct physical interactions, while indirect interactions and functional information were presented by interrupted lines. Utilizing the string software, we minimized false positives as well as false negatives, tagged all interactions as “Medium-confidence” (<0.4), and eliminated them from the analysis. Proteins not recorded were removed from the database. Cluster networks were created using the MCL inflation algorithm, which is included in the STRING search tool and a value of three was selected for all the analyses (26).

Sequence similarities of identified amino acid and carbohydrate *Porphyra* proteins involved in metabolism were searched using the BLAST program.^5^ We utilized the NCBI Conserved Domain Search Service^6^ to discover nucleotide sequences, and Texmaker to draw sequence alignment diagrams (27).

### Potential Bioactive Peptide Prediction

Potential bioactive peptides were performed *in silico* using the MS-Digest software, which is included in Protein Prospector (version 6.3.1)^7^ (28). Pepsin and trypsin enzymes are commonly chosen. ‘‘Per peptide’’ with a minimum of six residues and no missed cleavages, were selected as parameters. To evaluate the results, all digested peptides were ranked using the Peptide Ranker.^8^ This is a prediction of bioactive peptides tool based on a novel N-to-1 neural network (29). Any peptide predicted with an overtop score is labeled as bioactive for the objective protein. In addition, potential peptides were searched against CAMP^9^ to identify antibacterial properties and Toxin Pred^10^ was used to identify high toxic or non-toxic peptides (30, 31). Finally, the Swiss-Model serve^11^ was used to create 3D models and predict bioactive ligand mechanisms (32).

## Results and Discussion

### Identification of *Porphyra dentata* Proteome by Label-Free Proteomic Analysis

**FIGURE 1:**
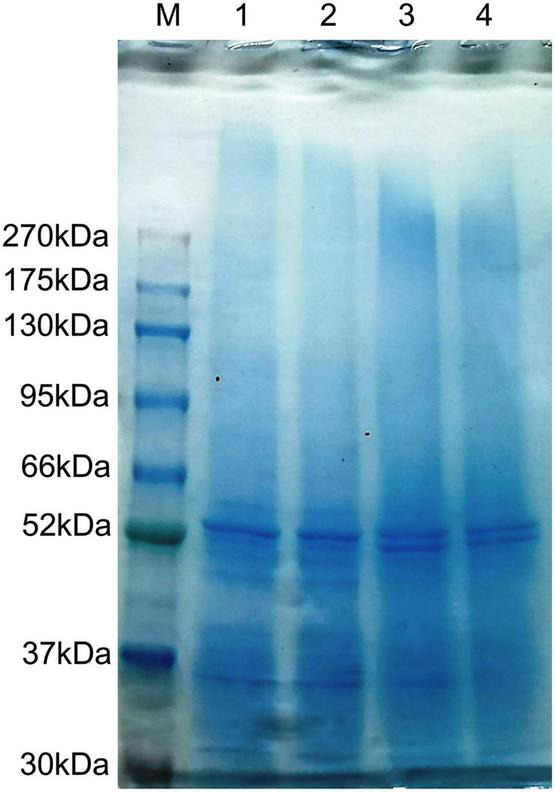
Sodium dodecyl sulfate-polyacrylamide gel electrophoresis 12% proteins of the *Porphyra dentata* samples (M, marker; 1–2, first harvest; 3–4, fifth harvest).

Based on LC-MS/MS and Proteome Discoverer analyses, a trypsin digestion search of protein extracts from *P. dentata* was performed. The repository stores 288,272 identification profiles from 13,046 different peptides and 2,187 different non-redundant annotated proteins with different sample repeats (*n* = 6). Results showed that the molecular weight of proteins was concentrated in the 1–21 and 21–41 k Da range, and were mostly small molecular proteins; most peptides’ length distribution was 10–15 bp. both the first and fifth harvest co-expression resulted in 419 non-redundant annotated proteins (*n* = 4). To further observe and compare protein extraction quality and characteristic distribution of the two harvests, whole protein extracts were separated by 12% SDS-PAGE electrophoresis, with two biological repetitions for each harvest period (Figure 1). The results showed that protein mass for each harvest was evenly distributed.

Through repository matching, we found less protein information and more species annotation, including proteins related to other red algae such as *Pyropia yezoensis* and *Porphyra umbilicalis.* In the repository, 77 labeled and unnamed protein products appeared and were annotated as *Chondrus crispus*, which has developmental similarities to *P. dentata.* In addition, there are 88 proteins that are not applicable (N/A). Further research should consider the limitations and characteristics of organisms sequencing, providing database updated accordingly. Supplementary Table 1 shows the complete annotated protein information. The raw data and analysis outputs (including all protein abundance) are publicly available at iProX data repository^12^ (Reference: PXD033319).

### Functional Analysis: Gene Ontologies and Pathways Analysis

**FIGURE 2:**
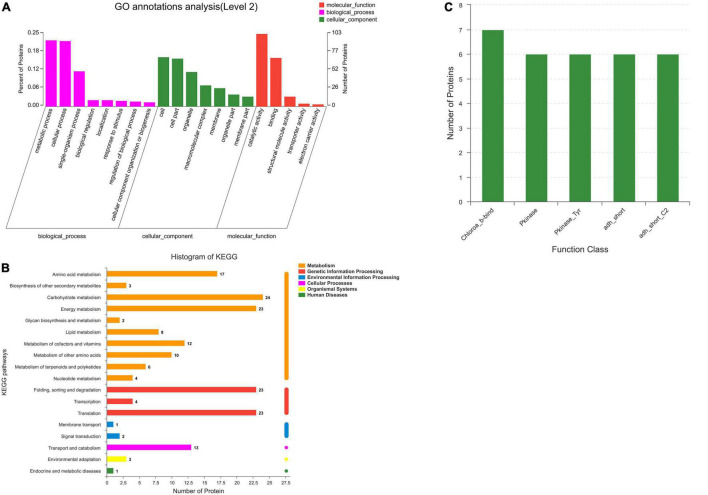
GO, KEGG pathway and function class analysis of *Porphyra dentata.* **(A)** Protein classes of *Porphyra dentata* by GO. **(B)** KEGG pathway analysis of *Porphyra dentata*. **(C)** Function class analysis of *Porphyra dentata*.

Go analysis was carried out on 419 annotated co-expression proteins of *P. dentata*; a total of 28 results were counted (Figure 2A). In the biological process, proteins are mainly enriched in metabolic (GO: 0008152, 94), cellular (GO: 0009987, 93), and single organizational pathways (Go: 0044699, 50). Cellular components consist of the cell (GO: 0005623, 70), cellular compartments (GO: 00044464, 68), and organelles (GO: 0043226, 49). Catalytic activity (GO:0003824, 103) and binding (GO:0005488, 69) are the main molecular function. The KEGG pathway showed that most identified proteins are involved in amino acid (17), carbohydrate (24), and energy metabolism (23). Identified proteins also participate in protein translation (23), folding, sorting, degradation, and genetic information processing (Figure 2B).

Moreover, the functional domain of *P. dentata* was studied and analyzed by Pfam software. Sequence functional regions in protein annotation were focused on chlorophyll A-B binding protein (Chloroa_b-bind); protein kinase domain (Pkinase); protein tyrosine kinase (Pkinase_Tyr); short-chain dehydrogenase (adh_short); enoyl-(Acyl carrier protein); reductase (adh_short_C2). Figure 2C shows the location number. Most of these domains are classified as proteases and may be related to quality differences in *P. dentata*.

### Interactive Network Analysis and Sequence Similarities

**FIGURE 3:**
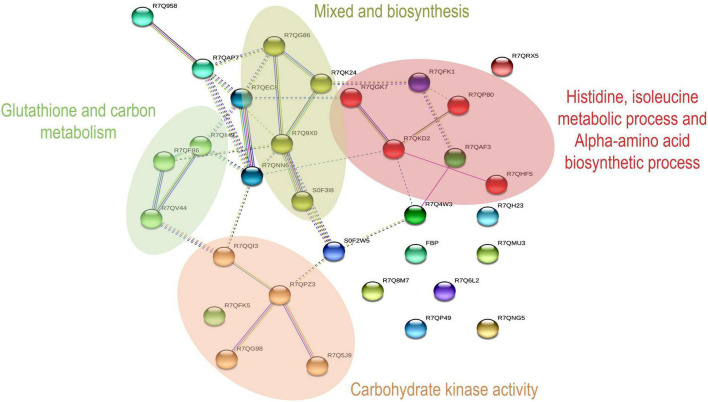
Network analysis by KEGG pathway in Amino acid metabolism and Carbohydrate metabolism.

Proteins involved in amino acid and carbohydrate metabolism from KEGG are summarized in Tables 1, 2. In STRING software network interaction analysis. *P. dentata* was not annotated in the software and not Auto-detect was not supported. According to the database search results, *C. crispus* was selected as a reference for the biological pattern analysis. Based on MCL clustering (MCL = 3), 32 nodes (proteins) and 37 edges (interactions) were obtained. The predicted network interaction was 10 and a significant increase in the number of edges may be attributed to increased protein expression and biological activity in *P. dentata* (Figure 3). Physical direct interactions are represented by continuous lines, while functional interactions are represented by discontinuous lines. In carbohydrate metabolism, have a protein indicated as N/A, no annotation information will be discussed temporarily.

**TABLE 1:** Amino acid metabolism protein.

Number	Description	Gene	Unique peptides	Coverage	MW (kDa)
1	Unnamed protein product = *Chondrus crispus*	CHC_T00005217001	11	40	31.4
2	Indole-3-glycerol phosphate synthase = *Chondrus crispus*	CHC_T00008561001	5	19	44.3
3	Putrescine aminopropyltransferase	CHC_T00009140001	1	3	40.1
4	Aminomethyltransferase = *Chondrus crispus*	CHC_T00008727001	11	38	41.4
5	5-methyltetrahydropteroyltriglutamate–homocysteine methyltransferase = *Chondrus crispus*	CHC_T00009326001	2	4	79.4
6	Dihydrodipicolinate synthase = *Galdieria sulphuraria*	Gasu_29240	12	46	34.2
7	4-hydroxyphenylpyruvate dioxygenase, 4HPPD = *Chondrus crispus*	CHC_T00009321001	7	24	43.7
8	Alanine transaminase = *Dumontia simplex*	CHC_T00009321001	21	74	54.8
9	Putative S-adenosylmethionine synthetase = *Pyropia yezoensis*	N/A	28	76	42.6
10	Maleylacetoacetate isomerase = *Cystobacter fuscus*	N/A	6	68	23
11	Cystathionine beta-lyase METC = *Chondrus crispus*	CHC_T00008829001	4	19	48.2
12	3-phosphoshikimate 1-carboxyvinyltransferase = *Chondrus crispus*	CHC_T00009255001	18	48	69.7
13	PREDICTED: acetyl-CoA acetyltransferase, mitochondrial isoform X2 = *Serinus canaria*	N/A	2	20	11.5
14	AGAP004880-PB = *Anopheles gambiae str. PEST*	AgaP_AGAP004880	2	9	34.8
15	Diaminopimelate epimerase, chloroplastic = *Auxenochlorella protothecoides*	F751_4193	4	25	30.9
16	Unnamed protein product = *Chondrus crispus*	CHC_T00000167001	4	18	36.6
17	Unnamed protein product = *Chondrus crispus*	CHC_T00006620001	4	15	47.2
					

**TABLE 2:** Carbohydrate metabolism protein.

Number	Description	Gene	Unique peptides	Coverage	MW (kDa)
1	Transketolase = *Pyropia yezoensis*	N/A	29	63	77.6
2	Hypothetical protein AMAG_02467 = *Allomyces macrogynus ATCC 38327*	N/A	1	24	16.4
3	Pyrophosphate–fructose-6-phosphate 1-phosphotransferase = *Galdieria sulphuraria*	Gasu_20900	13	34	64.2
4	Unnamed protein product = *Chondrus crispus*	CHC_T00006831001	12	44	44.2
5	L-galactose dehydrogenase = *Chondrus crispus*	CHC_T00010069001	16	81	34
6	Glyceraldehyde-3-phosphate dehydrogenase precursor = *Phaeodactylum tricornutum CCAP 1055/1*	GapC1	3	20	26.3
7	Phosphoglycerate kinase = *Pyropia yezoensis*	N/A	31	62	51.6
8	Unnamed protein product = *Chondrus crispus*	CHC_T00002260001	1	11	33.8
9	Ascorbate peroxidase = *Galdieria sulphuraria*	Gasu_16980	12	48	33.6
10	Probable inositol 2-dehydrogenase = *Chondrus crispus*	CHC_T00009082001	7	20	51
11	Aminomethyltransferase = *Chondrus crispus*	CHC_T00008727001	11	38	41.4
12	Trehalose-6-phosphate synthase = *Pyropia haitanensis*	N/A	24	40	101.7
13	Glucose-6-phosphate 1-dehydrogenase = *Phytophthora infestans T30-4*	PITG_00146	27	60	59.7
14	Fructose-1,6-bisphosphatase I = *Galdieria sulphuraria*	Gasu_02220	2	8	37.8
15	PREDICTED: glyceraldehyde-3-phosphate dehydrogenase-like = *Acropora digitifera*	LOC107332108	2	10	30.1
16	PREDICTED: acetyl-CoA acetyltransferase, mitochondrial isoform X2 = *Serinus canaria*	N/A	2	20	11.5
17	Triosephosphate isomerase = *Pyropia haitanensis*	N/A	16	56	40.2
18	Alpha-amylase = *Calothrix sp. PCC 7507*	N/A	5	14	53.4
19	Myo-inositol dehydrogenase = *Chondrus crispus*	CHC_T00008315001	22	77	44.2
20	AGAP004880-PB = *Anopheles gambiae str. PEST*	AgaP_AGAP004880	2	9	34.8
21	UDP-glucose dehydrogenase = *Chondrus crispus*	CHC_T00008869001	1	39	51.7
22	PREDICTED: 1-phosphatidylinositol 4,5-bisphosphate phosphodiesterase delta-3-A-like = *Poecilia latipinna*	Plcd3b	2	6	55.3
23	Isocitrate dehydrogenase	Gasu_48810	7	24	43.7

The interaction analysis shows nine different sub-networks, with enriched color annotations focused on four aspects. Red stands for the metabolic process of histidine, isoleucine, and the alpha-amino acid biosynthetic process. Brown is rumen kinase activity. In addition, dark golden-red is described and mixed, including the poly metallic process, and serine-type exercise activity. Green is glutathione metabolism and carbon metabolism, and they participate in endoplasmic reticulum protein processing, induce synthesis of specific and differential proteins, stimulate unique biological activities, and induce the formation of active peptides in *P. dentata.*

Other molecular networks such as phosphoglycerate kinase, annotated in Cyan, are a major protein involved in the ATP production stage of glycolysis and concerted catalysis with pyruvate kinase. Our previous study found that ATP could significantly affect the quality of *Porphyra* while harvesting during photosynthesis. Thus, the overall quality of *P. dentata* results in protein and carbohydrate changes, as well as flavor differences (17). During the first and fifth harvest of *P. dentata*, this network is the first and most comprehensive interactomics map of interactions between amino acids and carbohydrate metabolism functional substances involved (Supplementary Table 2 for full-color annotation).

**FIGURE 4:**
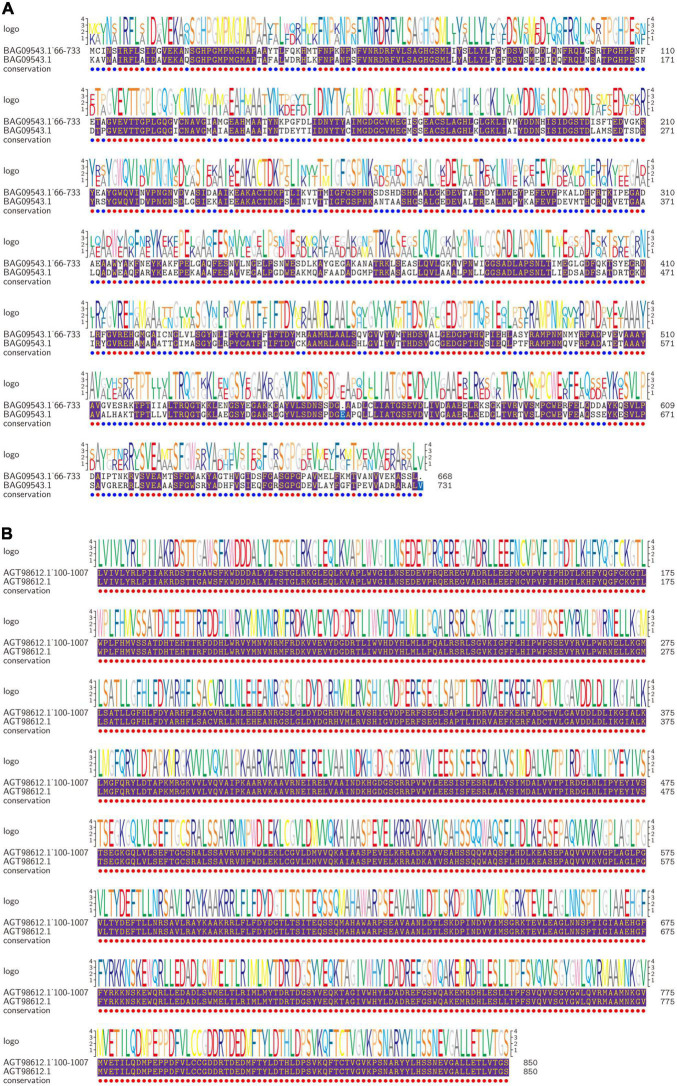
Proteins amino acid residues of predicted motifs comparison. The first layer is *NeoPyropia* protein, and second layer is target protein. The purple part is sequence homology (**A:** Transketolase; **B:** Trehalose-6-phosphate synthase).

To further analyze the biological functions of annotated proteins, the *Porphyra* biological information resource library was examined, and annotated proteins were screened out as *P. yezoensis*. We compared proteins’ active functional domains identified from amino acid sequences. Results showed that the most abundant functional domains in the target protein were transketolase (A) and trehalose-6-phosphate synthase (B); functional domains, respectively, were 72–731 and 76–850. The first layer was homologous closest *NeoPyropia yezoensis* protein and the second layer was our target protein in Figure 4 (Other small domains protein figure in Supplementary Figure 1). Trehalose-6-phosphate synthase (T6P) is essential for carbohydrate synthesis in plants (33). Previous studies found that T6P promoted the growth process and biosynthesis of the final substances in plant starch and promoted growth recovery after abiotic stress (34). Our study confirmed that with the gradual extension of harvest time, excitation of specific proteins reduced the production of proteins involved in photoinhibition and ensured stable the photosynthesis cycle. *P. dentata* gradually darkened, thickened, increased carbohydrate content, and decreased protein content (17). In addition, T6P can increase fatty acid synthesis and accumulation in *Porphyra* tissues, which was consistent with our results (determination not published). The amino acid content of the fifth harvest was higher than the first harvest.

### Prediction of Bioactive Peptides

Bioactive peptides are specific protein fragments that positively affect and regulate human functions and their characteristics are determined by amino acid composition. They can be released and become active to function as enzymes and improve both human immunity sources and the quality of functional food to provide nourishment (35, 36). Proteins were hydrolyzed with pepsin and trypsin in the MS digest *in silico* program, predicted peptides for each enzymatic hydrolysis are shown in Supplementary Table 3 (pepsin and trypsin). Pepsin cleavage sites P1 and P1′, primarily cleave proteins on Phe, Tyr, Trp, and Leu residues (37). A total of 1,271 and 2,191 different polypeptides were released from proteins involved in amino acid and carbohydrate metabolism (5–31 amino acid residues).

We utilized Peptide Ranker to sequence hydrolyzed peptides for each protein and select the peptide with the greatest biological activity. Referring to the Pan method (14), peptides with the highest Peptide Ranker score are considered potential bioactive peptides for the target protein (Table 3 is amino acid metabolism, Table 4 is carbohydrate metabolism). Based on the analysis, we found that the Alanine transaminase protein cannot be characterized by Amino acid metabolism. The putrescine amino propyltransferase protein predicted high peptide scores but was toxic. Finally, an unrecorded protein was characterized in carbohydrate metabolism and the presumed active protein was unique to *P. dentata*, which is not listed in the table.

**TABLE 3:** Selected potential bioactive peptides of the amino acid metabolism proteome predicted by *in silico* digestions with pepsin.

Number	Proteins	Peptides	Peptide ranker score	Anti-microbial peptide (AMP)	Toxin prediction
1	Unnamed protein product = *Chondrus crispus*	SGNWGDMF	0.949722	Non-AMP	Non-Toxin
2	Indole-3-glycerol phosphate synthase = *Chondrus crispus*	RKPPGCL	0.817088	Non-AMP	Non-Toxin
3	Putrescine aminopropyltransferase	CCQGECMWL	0.940187	Non-AMP	Toxin
4	Amino methyltransferase = *Chondrus crispus*	GIPCHVTRCGY	0.761282	Non-AMP	Non-Toxin
5	5-methyltetrahydropteroyltriglutamate–homocysteine methyltransferase = *Chondrus crispus*	GDTFPW	0.934555	Non-AMP	Non-Toxin
6	Dihydrodipicolinate synthase = *Galdieria sulphuraria*	SPAPPPPA	0.872731	Non-AMP	Non-Toxin
7	4-hydroxyphenylpyruvate dioxygenase, 4HPPD = *Chondrus crispus*	QPVGERGFGF	0.775063	Non-AMP	Non-Toxin
9	Putative S-adenosylmethionine synthetase = *Pyropia yezoensis*	PPPPPSVTMSAMKNF	0.900203	Non-AMP	Non-Toxin
10	Maleylacetoacetate isomerase = *Cystobacter fuscus*	GGTPSSPPPLRI	0.70861	Non-AMP	Non-Toxin
11	Cystathionine beta-lyase METC = *Chondrus crispus*	PFTGMW	0.964074	Non-AMP	Non-Toxin
12	3-phosphoshikimate 1-carboxyvinyltransferase = *Chondrus crispus*	GGPGGRFF	0.971099	Non-AMP	Non-Toxin
13	PREDICTED: acetyl-CoA acetyltransferase, mitochondrial isoform X2 = *Serinus canaria*	RTPIGSF	0.425774	Non-AMP	Non-Toxin
14	AGAP004880-PB = *Anopheles gambiae str. PEST*	CFPSVGGGGDRGVGSL	0.799074	Non-AMP	Non-Toxin
15	Diaminopimelate epimerase, chloroplastic = *Auxenochlorella protothecoides*	NSDGSEPEMCGNGVRCL	0.799423	Non-AMP	Non-Toxin
16	Unnamed protein product = C*hondrus crispus*	GVPMAW	0.853694	AMP	Non-Toxin
17	Unnamed protein product = *Chondrus crispus*	GRAPHPL	0.686141	Non-AMP	Non-Toxin
					

**TABLE 4:** Selected potential bioactive peptides of the carbohydrate metabolism proteome predicted by *in silico* digestions with pepsin.

Number	Proteins	Peptides	Peptide ranker score	Anti-microbial peptide (AMP)	Toxin prediction
1	Transketolase = *Pyropia yezoensis*	PCWEVF	0.895658	Non-AMP	Non-Toxin
2	Hypothetical protein AMAG_02467 = *Allomyces macrogynus ATCC 38327*	ARCDVPL	0.656485	Non-AMP	Non-Toxin
3	Pyrophosphate–fructose-6-phosphate 1-phosphotransferase = *Galdieria sulphuraria*	KCGGFPL	0.903338	Non-AMP	Non-Toxin
4	Unnamed protein product = *Chondrus crispus*	NDGWVF	0.889047	Non-AMP	Non-Toxin
5	L-galactose dehydrogenase = *Chondrus crispus*	GPPPWHPA	0.942954	Non-AMP	Non-Toxin
6	Glyceraldehyde-3-phosphate dehydrogenase precursor = *Phaeodactylum tricornutum CCAP 1055/1*	MLNPNF	0.830595	Non-AMP	Non-Toxin
7	Phosphoglycerate kinase = *Pyropia yezoensis*	PSCFPVA	0.789912	Non-AMP	Non-Toxin
8	Unnamed protein product = *Chondrus crispus*	GPPGVYSF	0.811631	Non-AMP	Non-Toxin
9	Ascorbate peroxidase = *Galdieria sulphuraria*	FERMTL	0.586192	Non-AMP	Non-Toxin
10	Probable inositol 2-dehydrogenase = *Chondrus crispus*	VGFQRRF	0.803701	Non-AMP	Non-Toxin
11	Aminomethyltransferase = *Chondrus crispus*	GIPCHTRCGY	0.77892	Non-AMP	Toxin
12	Trehalose-6-phosphate synthase = *Pyropia haitanensis*	QDMPEPPDFVL	0.7332	Non-AMP	Non-Toxin
13	Glucose-6-phosphate 1-dehydrogenase = *Phytophthora infestans T30-4*	SYGSRF	0.821114	Non-AMP	Non-Toxin
14	Fructose-1,6-bisphosphatase I = *Galdieria sulphuraria*	PWTRRSRCW	0.798827	Non-AMP	Non-Toxin
15	PREDICTED: glyceraldehyde-3-phosphate dehydrogenase-like = *Acropora digitifera*	QLSPTF	0.676813	Non-AMP	Non-Toxin
16	PREDICTED: acetyl-CoA acetyltransferase, mitochondrial isoform X2 = *Serinus canaria*	RTPIGSF	0.425774	Non-AMP	Non-Toxin
17	Triosephosphate isomerase = *Pyropia haitanensis*	CTCSCPSPFPPRSPSPVRKL	0.903692	Non-AMP	Non-Toxin
18	Alpha-amylase = *Calothrix sp. PCC 7507*	FRTVGF	0.690715	Non-AMP	Non-Toxin
19	Myo-inositol dehydrogenase = *Chondrus crispus*	PPPVGYL	0.842791	Non-AMP	Non-Toxin
20	AGAP004880-PB = *Anopheles gambiae str. PEST*	CFPSVGGGGDRGVGSL	0.799074	Non-AMP	Non-Toxin
21	UDP-glucose dehydrogenase = *Chondrus crispus*	QDGMVKPCFVF	0.849819	Non-AMP	Non-Toxin
22	PREDICTED: 1-phosphatidylinositol 4,5-bisphosphate phosphodiesterase delta-3-A-like = *Poecilia latipinna*	GPRLTF	0.841844	Non-AMP	Non-Toxin
23	Isocitrate dehydrogenase	GIGPGPGPGA	0.76398	Non-AMP	Non-Toxin

During trypsin digestion, it was found that Lys and Arg residues at the P1 position of the target protein were preferentially cleaved; however, the P1’ site was involved in the cleavage of pro (14). In total, 1,046 protein peptides were involved in amino acid metabolism and 1,870 protein peptides involved in carbohydrate metabolism were predicted (5–40 amino acid residues). Results from the Peptide Ranker prediction server showed that the activity of 3–phosphokinase–1–carboxyvinyltransferase protein was the highest, with an increased number of peptide toxicity predictions in carbohydrate metabolism proteins and no predicted peptides with antibacterial activity in trypsin digest (Table 5, Amino acid metabolism; Table 6, Carbohydrate metabolism).

**TABLE 5:** Selected potential bioactive peptides of the amino acid metabolism proteome predicted by *in silico* digestions with trypsin.

Number	Proteins	Peptides	Peptide ranker score	Anti-microbial peptide (AMP)	Toxin prediction
1	Unnamed protein product = *Chondrus crispus*	WGDGWR	0.957707	Non-AMP	Non-Toxin
2	Indole-3-glycerol phosphate synthase = *Chondrus crispus*	STPIPPGMAAFVPGAAAALR	0.807687	Non-AMP	Non-Toxin
3	Putrescine aminopropyltransferase	FSAAPASLAVAIMGLCGK	0.886888	Non-AMP	Non-Toxin
4	Aminomethyltransferase = *Chondrus crispus*	ASHLWVR	0.71333	Non-AMP	Non-Toxin
5	5-methyltetrahydropteroyltriglutamate–homocysteine methyltransferase = *Chondrus crispus*	EGLPLKRPAWAADVAWAVR	0.799328	Non-AMP	Non-Toxin
6	Dihydrodipicolinate synthase = *Galdieria sulphuraria*	LFADLFCMANPIPTK	0.740127	Non-AMP	Non-Toxin
7	4-hydroxyphenylpyruvate dioxygenase, 4HPPD = *Chondrus crispus*	GFGFGGGFLG	0.95293	Non-AMP	Non-Toxin
8	Alanine transaminase = *Dumontia simplex*	N/A	N/A	N/A	N/A
9	Putative S-adenosylmethionine synthetase = *Pyropia yezoensis*	PPPPPSVTMSAMK	0.86552	Non-AMP	Non-Toxin
10	Maleylacetoacetate isomerase = *Cystobacter fuscus*	SSCAWR	0.868969	Non-AMP	Non-Toxin
11	Cystathionine beta-lyase METC = *Chondrus crispus*	GFCDKFLGR	0.94263	Non-AMP	Toxin
12	3-phosphoshikimate 1-carboxyvinyltransferase = *Chondrus crispus*	MAMAFALAACGKVGVD ICDPGCTAK	0.998508	Non-AMP	Non-Toxin
13	PREDICTED: acetyl-CoA acetyltransferase, mitochondrial isoform X2 = *Serinus canaria*	VCASGLKAVALAADSLALGR	0.780587	Non-AMP	Non-Toxin
14	AGAP004880-PB = *Anopheles gambiae str. PEST*	GAKVTIVGCGSVGMACASAILS TGLASTLVFADVDAK	0.901105	Non-AMP	Non-Toxin
15	Diaminopimelate epimerase, chloroplastic = *Auxenochlorella protothecoides*	ACGTGACAVVVAAVLTGRTER	0.901392	Non-AMP	Non-Toxin
16	Unnamed protein product = *Chondrus crispus*	ASLSAPGPTGDALRGLCR	0.785594	Non-AMP	Non-Toxin
17	Unnamed protein product = *Chondrus crispus*	GVVQGEPLFKLPLTVLLTK	0.756829	Non-AMP	Non-Toxin

**TABLE 6:** Selected potential bioactive peptides of the carbohydrate metabolism proteome predicted by *in silico* digestions with trypsin.

Number	Proteins	Peptides	Peptide ranker score	Anti-microbial peptide (AMP)	Toxin prediction
1	Transketolase = *Pyropia yezoensis*	MAFVAASLSAGCLAGGRPVRK	0.971674	Non-AMP	Non-Toxin
2	Hypothetical protein AMAG_02467 = *Allomyces macrogynus* ATCC 38327	PPLAYGTAGFR	0.825692	Non-AMP	Non-Toxin
3	Pyrophosphate–fructose-6-phosphate 1-phosphotransferase = *Galdieria sulphuraria*	CGGFPLTMMMNIERR	0.873887	Non-AMP	Non-Toxin
4	Unnamed protein product = *Chondrus crispus*	VDGVFGSRITGGGFGGCTVSLAK	0.749533	Non-AMP	Non-Toxin
5	L-galactose dehydrogenase = *Chondrus crispus*	AIFKPVANVAWASGKFPGA	0.770358	Non-AMP	Non-Toxin
6	Glyceraldehyde-3-phosphate dehydrogenase precursor = *Phaeodactylum tricornutum CCAP 1055/1*	DWRGGR	0.802119	Non-AMP	Non-Toxin
7	Phosphoglycerate kinase = *Pyropia yezoensis*	SSLPLPITMAFVSAPAALR	0.760034	Non-AMP	Non-Toxin
8	Unnamed protein product = *Chondrus crispus*	FRLDDPSPVVLPARPGAAS DDDDDADAQR	0.719285	Non-AMP	Non-Toxin
9	Ascorbate peroxidase = *Galdieria sulphuraria*	DGVSVADFFAFAGAVAVEVAAGPR	0.753986	Non-AMP	Non-Toxin
10	Probable inositol 2-dehydrogenase = *Chondrus crispus*	SGGIFLDMASHDFDMAR	0.768419	Non-AMP	Non-Toxin
11	Aminomethyltransferase = *Chondrus crispus*	ASHLWVR	0.71333	Non-AMP	Non-Toxin
12	Trehalose-6-phosphate synthase = *Pyropia haitanensis*	FDDHLWR	0.909053	Non-AMP	Non-Toxin
13	Glucose-6-phosphate 1-dehydrogenase = *Phytophthora infestans T30-4*	GGYFDSFGIIR	0.904428	Non-AMP	Non-Toxin
14	Fructose-1,6-bisphosphatase I = *Galdieria sulphuraria*	DMGAIFGVFR	0.837907	Non-AMP	Non-Toxin
15	PREDICTED: glyceraldehyde-3-phosphate dehydrogenase-like = *Acropora digitifera*	SMNVVSNASCTTNCLAPLAKVINDK	0.851479	Non-AMP	Non-Toxin
16	PREDICTED: acetyl-CoA acetyltransferase, mitochondrial isoform X2 = *Serinus canaria*	VCASGLKAVALAADSLALGR	0.780587	Non-AMP	Non-Toxin
17	Triosephosphate isomerase = Pyropia haitanensis	CRAIACTCSCPSPFPPR	0.894172	Non-AMP	Toxin
18	Alpha-amylase = *Calothrix sp. PCC 7507*	FTFPGR	0.904087	Non-AMP	Non-Toxin
19	Myo-inositol dehydrogenase = *Chondrus crispus*	VHVGIIGCGRIGQCHAANLANK	0.965053	Non-AMP	Non-Toxin
20	AGAP004880-PB = *Anopheles gambiae str. PEST*	GAKVTIVGCGSVGMACASAI LSTGLASTLVFADVDAK	0.901105	Non-AMP	Non-Toxin
21	UDP-glucose dehydrogenase = *Chondrus crispus*	MVSNPVQTGLRICCIGAG YVGGPTMAMMALK	0.897035	Non-AMP	Toxin
22	PREDICTED: 1-phosphatidylinositol 4,5-bisphosphate phosphodiesterase delta-3-A-like = *Poecilia latipinna*	MATHLTAGLGELLRLPQPA DVDALPTLGSLLGK	0.839827	Non-AMP	Non-Toxin
23	Isocitrate dehydrogenase	SADGLFLDCCKR	0.866763	Non-AMP	Toxin

In Peptide Ranker analysis, peptides with scores higher than 0.9 were considered to have more biological potential activity (38). Peptide 3–phosphokinase–1–carboxyvinyltransferase’s in amino acid metabolism ranker scores reached 0.971099 and 0.998508. A previous study confirmed and identified a potential drug target in *Brucella millitensis* (16M simulated protein screening library) for the drug treatment of brucellosis (39). Therefore, we identified a potential pepsin/trypsin digest of peptides (GGPGGRFF, MAMAFALAACGKVGVDICDPGCTAK) that may be used as a natural plant preparation in antibacterial research to improve the health and stability of animal-derived foods and drugs.

The predictive peptide scores for Cystathionine beta-lyase MetC are 0.964074 and 0.94263. It is the key factor of methionine synthesis and can be used as a growth regulator or for flavor preparation in food production (40). In terms of lactobacillus fermentation products, volatile sulfur compounds (VSCs) are important substances that help achieve the desired flavor of fermented foods. Cystathionine beta lyase MetC can stimulate cysteine synthesis and improve amino acid metabolism. Presently, Cystathione beta lyase MetC can be inoculated and is preserved as the dominant strain in cheese production (41, 42). In the future, the natural bioactive peptide from *P. dentata* can be applied to the fermentation of food products, such as yogurt, cheese, and bacon. It can also be used to stimulate diversified applications and development advantages in seafood. Unfortunately, trypsin digestion causes toxicity; therefore, the production and application of this peptide need to be further studied.

**FIGURE 5:**
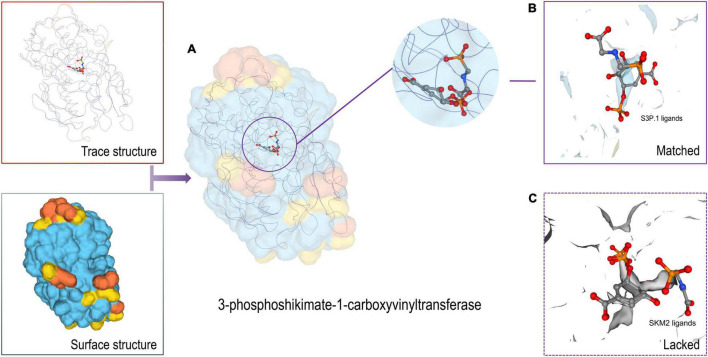
3D model structure of protein and ligands (**A**: 3-phosphoshikimate-1-carboxyvinyltransferase; **B**: S3P.1 ligands; **C**: SKM2 ligands).

In protein structure, the ligand is a signaling molecule and matches specific receptor protein sites on the walls of an organism’s somatic cell. When the ligand binds to the specific protein site, it will change the physical morphology of the protein, and a change in the shape of the receptor protein may activate or inhibit another biological mechanism, which is related to this specific interaction. Protein-ligand interactions are crucial to the creation of new drugs for the treatment of diseases and the development of bioactive substances (43). Here, we used 3D-model constructs to predict the protein structure of 3-phoshikimate-1-carboxyvinyltransferase and cystathionine beta lyase MetC. Predicted peptides are located in the protein structure. The target protein was predicted by Swiss-model. 3-phoshikimate-1-carboxyvinyltransferase protein had the highest matching degree of 1.02-angstrom resolution crystal structure with 3–phosphoshikimate–1–carboxyvinyltransfer from *Vibrio cholerae* in complex with shikimate-3-phosphate (partially photolyzed) and glyphosate (SMTL ID: 3nvs.1.A). The GMQE score was 0.51 and Seq Identity was 54.85% (Figure 5A). The monomer is similar to 1 × glyphosate and 1 × shikimate-3-phosphate. In *P. dentata*, a total of 14 residues within 4 Å, correlated with 3nvs.1.A. less A119. The ligand co-expression site of S3P.1 and the lack of site SKM2 are shown in Figures 5B,C. We hypothesized that 3–phosphoshikimate–1–carboxyvinyltransferase protein lacks ligands, but its structural similarity is low. The unique functional domain structure can cooperate with the S3P.1 site to stimulate the creation and expression of biological activity, resulting in a better therapeutic effect.

**FIGURE 6:**
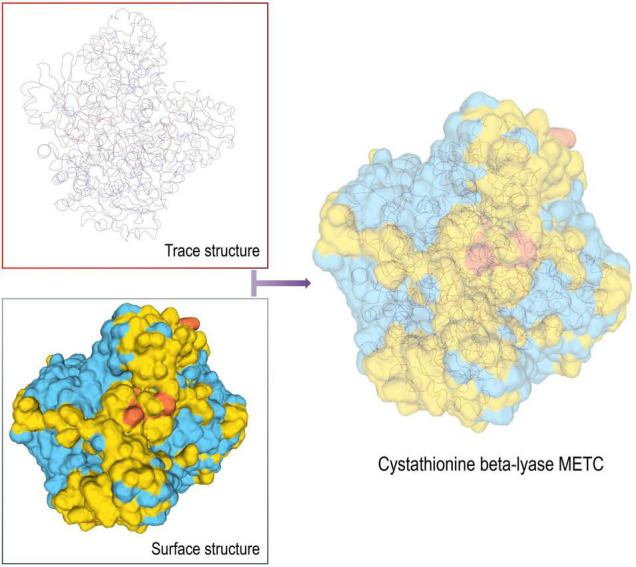
3D model structure of Cystathionine beta-lyase METC protein.

Compared with 3–phosphoshikimate–1–carboxyvinyltransferase, the tertiary structure of Cystathionine beta-lyase METC was obtained by sequence alignment, and the P113S Mutant of E. coli Cystathionine beta-lyase MetC structure (STML ID: 4ITG.1a) was used as a template. The GMQE score was 0.68 and the seq identity was 40.26% (Figure 6). Our previous determination confirmed that amino acid content increased with gradually prolonging the harvest (determination not published), and palate fullness in *P. dentata*. Unfortunately, no ligand was found in the predicted protein, suggesting that bioactive sites were stored in the protein as a whole. This research focuses on the production of diversified bioactive peptides, enriches sustainable development in the seaweed industry, and highlights the developmental potential of *P. dentata* an edible seaweed source and bioactive compound.

### Others Bioactive Peptides Expectation

In conclusion, active peptide hydrolyzed with trypsin scores better than pepsin in carbohydrate metabolism. In pepsin-predicted peptides, acetyl-CoA acetyltransferase, mitochondrial isoform X2, and Ascorbate peroxidase protein ranker scores are lower than 0.6, with no biological significance (38). It has been reported that most of the active peptides in *Porphyra* are released by pepsin, but peptides hydrolyzed by trypsin in *Porphyra haitanensis* have better anti-inflammatory and hypoglycemic activities, which are similar to our prediction results. Carbohydrates can be fully digested, and this approach can be applied to potential drugs and biological additives (44).

Transketolase is an important glycolytic synthase in the pentose phosphate pathway and is involved in oxidative stress processes in cells. Clinical studies confirmed that Transketolase can affect the production of NAPDH, inhibition of oxidative stress, and promote the growth of cancer cells (45). Our data results show that the peptide obtained by trypsin (MAFVAASLSAGCLAGGRPVRK) has a score of 0.971674 and is not classified as an anti-microbial. It can be used as a potential anti-inflammatory and targeted biological regulatory peptide in the treatment of cancer cells or used in the molecular synthesis of antioxidants for cancer research.

Myo-inositol dehydrogenase is the first step in catalyzing the catabolism of inositol. It is closely related to NAD, a homologous biological substance that can be used in the production of medicine and food. VHVGIIGCGRIGQCHAANLANK ranker score is 0.965053. Not only is it used for cheese fermentation, but it has become the dominant strain to improve the activity of fermentation substrates and is also a potential drug for treating Alzheimer’s disease (46, 47).

Finally, the Collection of Anti-Microbial Peptides (CAMP) database showed that only one unnamed protein product [*C. Crispus* (GVPMAW)] was determined with a potential anti-microbial peptide. Further studies are required because the benefits of *P. dentata* are yet to be elucidated.

For *P. dentata*, a synthetic bioactivity analysis is required to discuss and apply potential bioactive peptides. The bioinformatics method provides a faster and lower cost potential prediction method to screen and locate potential targets and provides a macro-analysis and demonstration for development.

## Conclusion

In this study, Label-free shotgun proteomics was used for the first time to identify protein changes and characterization of different harvest periods of *P. dentata*. A total of 13,046 different peptides were identified and 419 co-expression target proteins were obtained. Bioinformatics is used for research, including GO, KEGG, and STRING network interaction analysis. Results showed that metabolic process, cell, and catalytic activity were enrichment in GO and Amino acid metabolism, Carbohydrate metabolism, Energy metabolism, Genetic Folding, sorting, and degradation, and Translation in KEGG most prominent. Squido Jumbo network diagrams of amino acid metabolism and carbohydrate metabolism containing 32 protein interaction nodes were constructed, this is the first time to analyze interaction functional annotation proteins of *P. dentata* during the first and fifth harvest period and compared with the *Porphyra* resource library to enrich the expression of information. In addition, pepsin and trypsin were used for different digestion hydrolysis, predicting potential bioactive peptides. The results show that bioactive peptides can be high-quality fermentation active substances and drug activity, are potential targets for production. By combining proteomic results and bioinformatics analysis, we comprehensive understanding of functional changes in different harvest periods of *P. dentata*, providing a potential development direction for application as a source of food and biomedical compounds.

## Data Availability Statement

The original contributions presented in the study are included in the article/Supplementary Material, further inquiries can be directed to the corresponding author.

## Author Contributions

MY, LM, and YZ wrote the first draft of the manuscript. XY, LL, and SC wrote sections of the manuscript. BQ, YW, CL, and SY organized data and contributed to visualization. YZ conceptualized the idea. All authors contributed to manuscript revision, read, and approved the submitted version.

## Conflict of Interest

The authors declare that the research was conducted in the absence of any commercial or financial relationships that could be construed as a potential conflict of interest.

## Publisher’s Note

All claims expressed in this article are solely those of the authors and do not necessarily represent those of their affiliated organizations, or those of the publisher, the editors and the reviewers. Any product that may be evaluated in this article, or claim that may be made by its manufacturer, is not guaranteed or endorsed by the publisher.

## Footnotes

1
http://geneontology.org/

2
http://www.genome.jp/kegg

3
http://pfam.xfam.org/

4
http://stringdb.org/

5
http://blast.ncbi.nlm.nih.gov/

6
https://www.ncbi.nlm.nih.gov/Structure/cdd/wrpsb.cgi

7
http://prospector.ucsf.edu/prospector/mshome.htm

8
http://distilldeep.ucd.ie/PeptideRanker/

9
http://www.bicnirrh.res.in/antimicrobial/

10
https://webs.iiitd.edu.in/raghava/toxinpred/multi_submit.php

11
http://swissmodel.expasy.org

12
https://www.iprox.cn
