# Supplementary material for: Bioinformatic Prediction and Characterization of Proteins in Porphyra dentata by Shotgun Proteomics
Source: Front Nutr. 2022 Jul 7;9:924524. doi: 10.3389/fnut.2022.924524 (PMC9301277; doi:10.3389/fnut.2022.924524)
Supplement: Supplementary file 1 [file Data_Sheet_1.DOCX]

Supplementary Material


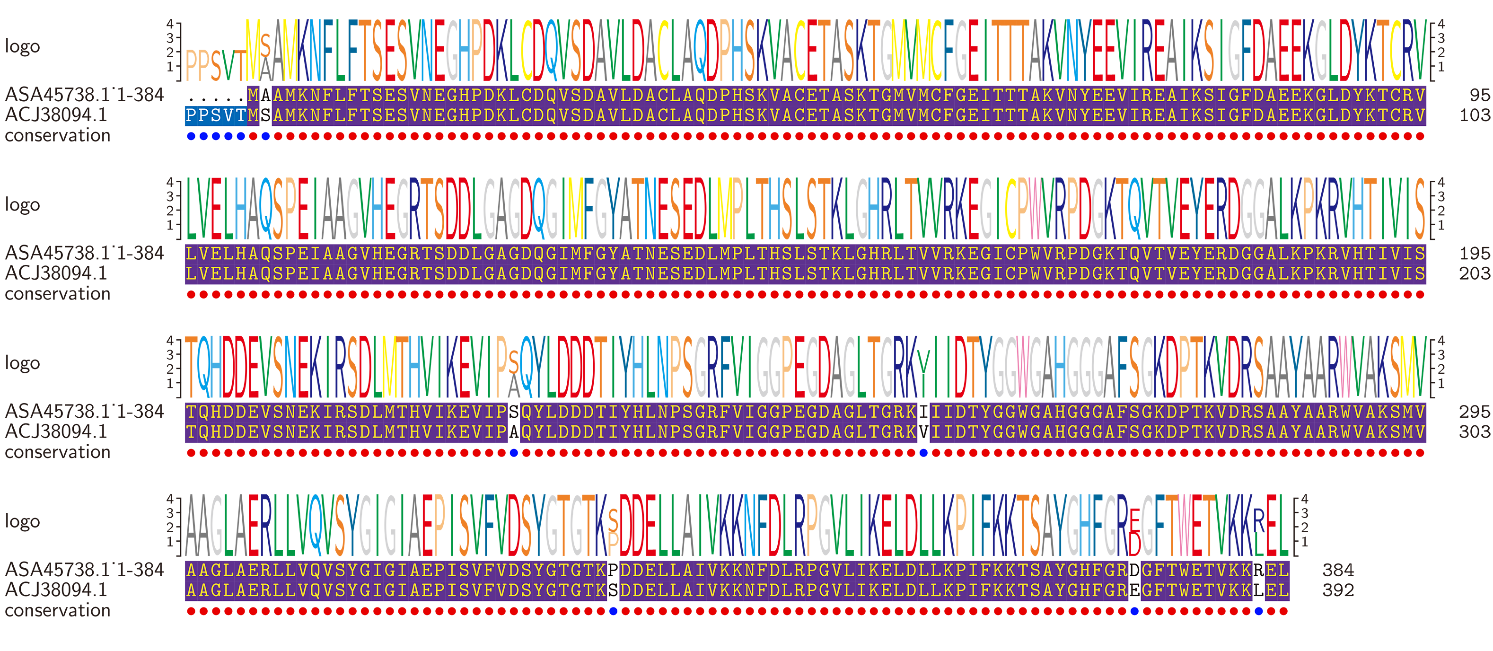


A. Putative S-adenosylmethionine synthetase. (locus of 4 – 392)


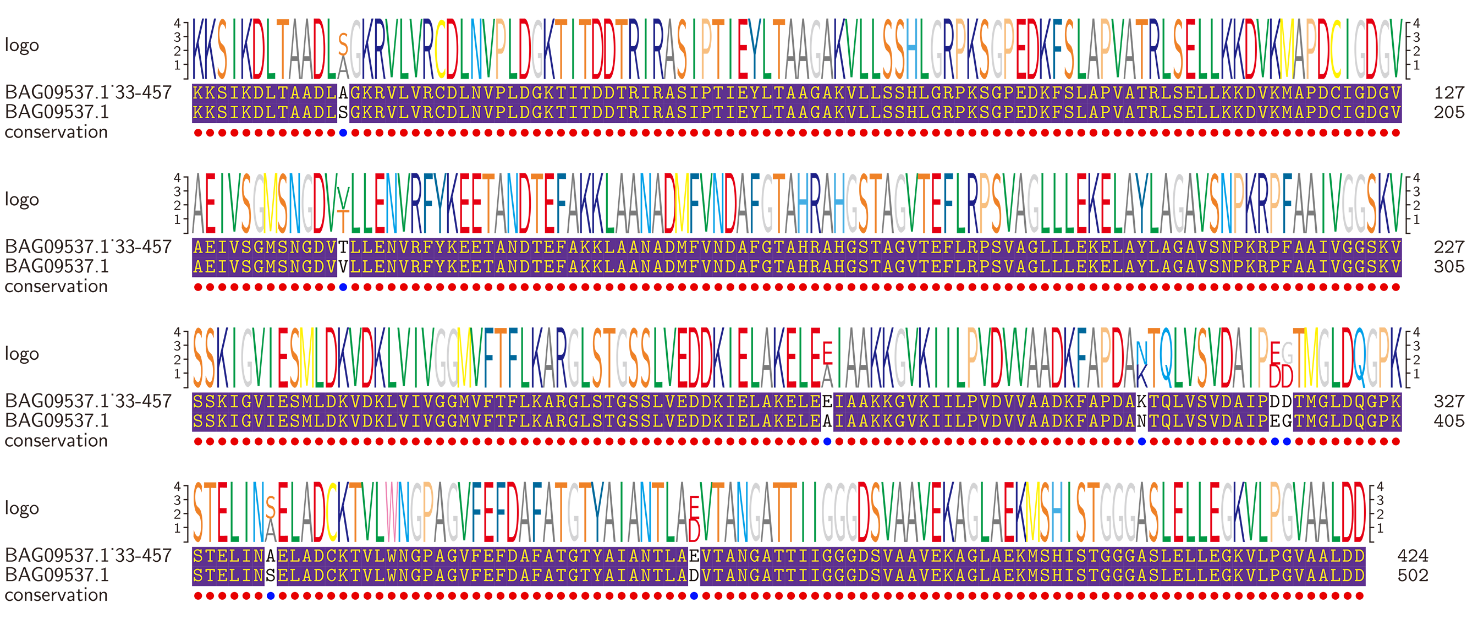


B. Phosphoglycerate kinase (locus of 106 – 502)


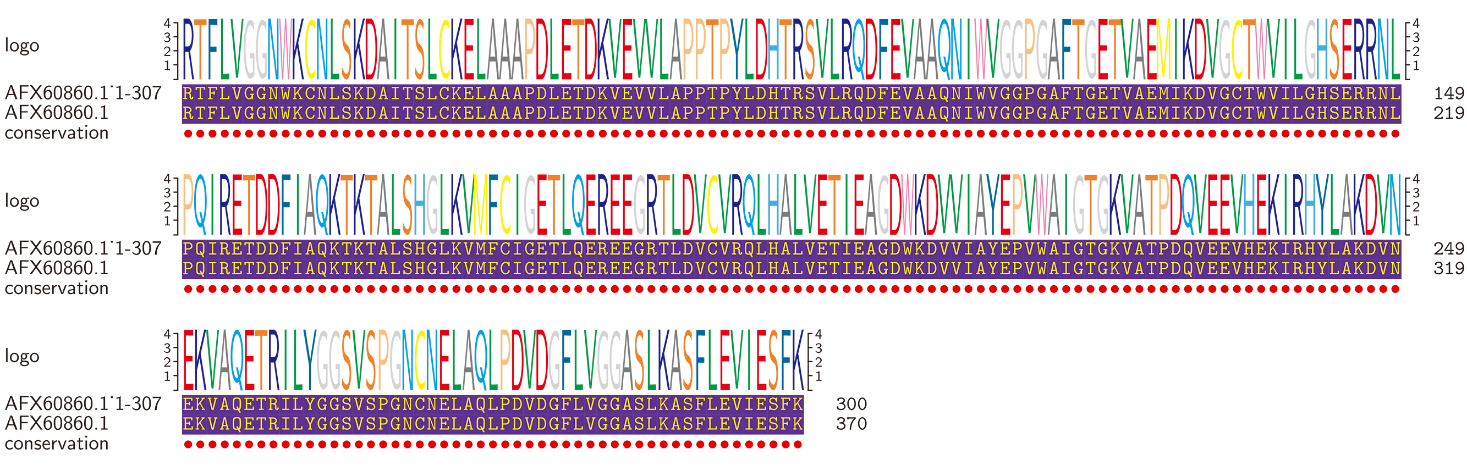


C. Triosephosphate isomerase (locus of 120 – 370)

**Figure S1.** Proteins amino acid residues of predicted motifs comparison (others).

Note: The first layer is *NeoPyropia yezoensis* protein, and second layer is target protein. The purple part is sequence homology.
