# Supplementary material for: Bioinformatic Prediction and Characterization of Proteins in Porphyra dentata by Shotgun Proteomics
Source: Front Nutr. 2022 Jul 7;9:924524. doi: 10.3389/fnut.2022.924524 (PMC9301277; doi:10.3389/fnut.2022.924524)
Supplement: Supplementary file 2 [file Table_1.DOCX]

Supplementary Material

Table S1. *Porphyra dentata* by Proteome Discoverer proteins (FDR < 1%, remove N/A proteins, n=331).

| N | Accession_id | Description | Gene | Cov% | PSM | Uni.Pep. |
| --- | --- | --- | --- | --- | --- | --- |
| 1 | TRINITY_DN10075_c6_g5_i3_m.106396 | Hypothetical protein=*Sulfitobacter noctilucicola* | N/A | 85 | 620 | 7 |
| 2 | TRINITY_DN10419_c2_g1_i1_m.270 | Protein disulfide-isomerase, PDIA-4 Endoplasmic reticulum lumen=*Chondrus crispus* | CHC_T00009389001 | 72 | 499 | 43 |
| 3 | TRINITY_DN9929_c0_g4_i1_m.42114 | Phosphoglycerate kinase=*Pyropia yezoensis* | N/A | 62 | 566 | 31 |
| 4 | TRINITY_DN10628_c5_g1_i1_m.19390 | Hypothetical protein EMIHUDRAFT_439617=*Emiliania huxleyi CCMP1516* | EMIHUDRAFT_439617 | 78 | 596 | 19 |
| 5 | TRINITY_DN9786_c0_g1_i2_m.47457 | Unnamed protein product= *Chondrus crispus* | CHC_T00003048001 | 73 | 380 | 35 |
| 6 | TRINITY_DN9892_c2_g3_i1_m.94778 | Putative S-adenosylmethionine synthetase=*Pyropia yezoensis* | N/A | 76 | 383 | 28 |
| 7 | TRINITY_DN9754_c0_g1_i1_m.50420 | Transketolase=Pyropia yezoensis | N/A | 63 | 313 | 29 |
| 8 | TRINITY_DN10232_c0_g3_i2_m.30734 | Heat shock protein 70-2=*Pyropia haitanensis* | N/A | 57 | 273 | 27 |
| 9 | TRINITY_DN9112_c0_g1_i1_m.79261 | Disulfide-isomerase, PDIA-3 Endoplasmic reticulum lumen=*Chondrus crispus* | CHC_T00008626001 | 63 | 193 | 32 |
| 10 | TRINITY_DN10152_c1_g8_i1_m.68988 | Unnamed protein product=*Chondrus crispus* | CHC_T00005189001 | 51 | 180 | 31 |
| 11 | TRINITY_DN5594_c0_g3_i1_m.52487 | Elongation factor Tu | TufA | 80 | 654 | 12 |
| 12 | TRINITY_DN10134_c4_g3_i1_m.69375 | D-inositol-3-phosphate glycosyltransferase=*Chondrus crispus* | CHC_T00010280001 | 59 | 228 | 30 |
| 13 | TRINITY_DN10202_c2_g1_i1_m.31055 | Alanine transaminase=*Dumontia simplex* | N/A | 74 | 181 | 21 |
| 14 | TRINITY_DN10216_c0_g7_i1_m.34043 | Clathrin heavy chain=*Chondrus crispus* | CHC_T00008543001 | 32 | 147 | 33 |
| 15 | TRINITY_DN8881_c0_g1_i2_m.9251 | Myo-inositol dehydrogenase=*Chondrus crispus* | CHC_T00008315001 | 77 | 206 | 22 |
| 16 | TRINITY_DN10738_c1_g2_i2_m.120403 | Unnamed protein product=*Chondrus crispus* | CHC_T00002029001 | 51 | 207 | 27 |
| 17 | TRINITY_DN10607_c3_g2_i1_m.20594 | Plastid Clp protease precursor=*Karenia brevis* | N/A | 42 | 157 | 31 |
| 18 | TRINITY_DN10411_c0_g1_i1_m.2324 | Glucose-6-phosphate 1-dehydrogenase=*Phytophthora infestans T30-4* | PITG_00146 | 60 | 223 | 27 |
| 19 | TRINITY_DN10451_c0_g1_i4_m.970 | Phycobilisome 31.8kD linker polypeptide=*Chondrus crispus* | CHC_T00009048001 | 55 | 287 | 4 |
| 20 | TRINITY_DN10083_c2_g1_i6_m.103849 | 14-3-3 protein-like protein=*Galdieria sulphuraria* | Gasu_35850 | 54 | 180 | 11 |
| 21 | TRINITY_DN10232_c0_g1_i6_m.30722 | Heat shock protein 70-5=*Pyropia haitanensis* | N/A | 41 | 159 | 27 |
| 22 | TRINITY_DN8194_c0_g1_i1_m.51012 | Photosystem II 44 kDa protein | Psbc | 34 | 305 | 13 |
| 23 | TRINITY_DN9170_c0_g1_i1_m.79246 | Phytochelatin synthase=*Nostoc sp. PCC7107* | N/A | 52 | 289 | 14 |
| 24 | TRINITY_DN5594_c0_g1_i1_m.52485 | Elongation factor Tu=*Porphyra purpurea* | TufA | 77 | 358 | 10 |
| 25 | TRINITY_DN4741_c0_g1_i1_m.90605 | Glutathione reductase=*Pyropia haitanensis* | N/A | 44 | 119 | 18 |
| 26 | TRINITY_DN9927_c1_g7_i1_m.42688 | Hypothetical protein=*Absidia glauca* | N/A | 67 | 172 | 9 |
| 27 | TRINITY_DN9475_c0_g1_i1_m.23040 | Glycerol-3-phosphate dehydrogenase=*Pyropia haitanensis* | N/A | 60 | 159 | 18 |
| 28 | TRINITY_DN10711_c2_g11_i1_m.119233 | Sulfate adenylyltransferase, SAT=*Chondrus crispus* | CHC_T00008316001 | 50 | 181 | 21 |
| 29 | TRINITY_DN10069_c3_g10_i1_m.105368 | Unnamed protein product=*Chondrus crispus* | CHC_T00001335001 | 58 | 119 | 17 |
| 30 | TRINITY_DN9835_c0_g2_i1_m.92475 | Triosephosphate isomerase=*Pyropia haitanensis* | N/A | 56 | 225 | 16 |
| 31 | TRINITY_DN10743_c3_g22_i1_m.122031 | Unnamed protein product=*Chondrus crispus* | CHC_T00005217001 | 40 | 165 | 11 |
| 32 | TRINITY_DN10251_c2_g4_i1_m.30011 | Heat shock protein 22=*Pyropia haitanensis* | N/A | 82 | 122 | 12 |
| 33 | TRINITY_DN10380_c0_g2_i5_m.116477 | Tubulin binding protein=*Cryptococcus gattii WM276* | CGB_B6140C | 52 | 245 | 11 |
| 34 | TRINITY_DN10561_c4_g7_i2_m.60509 | TBB_PYRYE RecName: Full=*Tubulin beta chain* | N/A | 58 | 86 | 19 |
| 35 | TRINITY_DN10574_c5_g1_i1_m.56344 | L-galactose dehydrogenase=*Chondrus crispus* | CHC_T00010069001 | 81 | 95 | 16 |
| 36 | TRINITY_DN9612_c0_g6_i1_m.99113 | 3-phosphoshikimate 1-carboxyvinyltransferase=*Chondrus crispus* | CHC_T00009255001 | 48 | 134 | 18 |
| 37 | TRINITY_DN10420_c3_g1_i1_m.7575 | Unnamed protein product=*Chondrus crispus* | CHC_T00003260001 | 75 | 109 | 14 |
| 38 | TRINITY_DN10402_c4_g3_i1_m.641 | Chaperonine 60=*Chondrus crispus* | CHC_T00008986001 | 46 | 105 | 22 |
| 39 | TRINITY_DN10269_c2_g2_i1_m.35819 | Trehalose-6-phosphate synthase=*Pyropia haitanensis* | N/A | 40 | 91 | 24 |
| 40 | TRINITY_DN10712_c5_g9_i1_m.118638 | Heat shock protein 70b=*Pyropia seriata* | N/A | 43 | 92 | 22 |
| 41 | TRINITY_DN7537_c0_g1_i1_m.77363 | unnamed protein product=*Chondrus crispus* | CHC_T00006831001 | 44 | 104 | 12 |
| 42 | TRINITY_DN13207_c0_g1_i1_m.24141 | NADPH:protochlorophyllide oxidoreductase=*Chondrus crispus* | CHC_T00009425001 | 67 | 84 | 15 |
| 43 | TRINITY_DN10274_c0_g2_i1_m.35338 | Unnamed protein product=*Chondrus crispus* | CHC_T00005387001 | 70 | 100 | 11 |
| 44 | TRINITY_DN9495_c0_g1_i1_m.24029 | Uroporphyrinogen decarboxylase 2, chloroplastic=*Chondrus crispus* | CHC_T00008531001 | 37 | 95 | 11 |
| 45 | TRINITY_DN9448_c0_g1_i1_m.23401 | Transducin family protein / WD-40 repeat family protein=*Klebsormidium flaccidum* | N/A | 40 | 96 | 18 |
| 46 | TRINITY_DN10660_c2_g7_i1_m.13547 | Unnamed protein product=*Chondrus crispus* | CHC_T00001448001 | 41 | 99 | 10 |
| 47 | TRINITY_DN10732_c0_g2_i1_m.118766 | Tubulin alpha chain=*Coccomyxa subellipsoidea C-169* | COCSUDRAFT_39871 | 40 | 91 | 4 |
| 48 | TRINITY_DN10579_c4_g4_i1_m.61140 | Starch synthase=*Chondrus crispus* | CHC_T00009277001 | 20 | 67 | 22 |
| 49 | TRINITY_DN5882_c0_g1_i1_m.24735 | Unnamed protein product=*Chondrus crispus* | CHC_T00001552001 | 66 | 56 | 10 |
| 50 | TRINITY_DN9175_c0_g1_i1_m.79367 | Unnamed protein product= *Chondrus crispus* | CHC_T00005732001 | 53 | 74 | 11 |
| 51 | TRINITY_DN9752_c0_g2_i1_m.48770 | Translation elongation factor EF-Tu, mitochondrial=*Chondrus crispus* | CHC_T00009351001 | 38 | 118 | 12 |
| 52 | TRINITY_DN10003_c0_g2_i2_m.102247 | Hypothetical protein PTSG_09571=*Salpingoeca rosetta* | PTSG_09571 | 52 | 76 | 9 |
| 53 | TRINITY_DN10025_c1_g5_i2_m.100861 | Heme oxygenase=*Cyanidioschyzon merolae strain 10D* | CYME_CMH209C | 48 | 101 | 10 |
| 54 | TRINITY_DN10727_c2_g1_i1_m.121525 | Plastid oxygen-evolving enhancer 1 precursor=*Pyropia yezoensis* | N/A | 55 | 102 | 17 |
| 55 | TRINITY_DN8194_c0_g1_i1_m.51013 | Photosystem II protein D2=*Porphyra purpurea* | PsbD | 30 | 174 | 11 |
| 56 | TRINITY_DN10131_c3_g2_i3_m.75781 | Hypothetical protein=*Magnetococcus marinus* | N/A | 45 | 100 | 13 |
| 57 | TRINITY_DN10119_c4_g3_i1_m.72174 | Unnamed protein product= *Chondrus crispus* | CHC_T00007779001 | 46 | 72 | 10 |
| 58 | TRINITY_DN10761_c3_g6_i1_m.123061 | Ascorbate peroxidase=*Galdieria sulphuraria* | Gasu_16980 | 48 | 82 | 12 |
| 59 | TRINITY_DN5992_c0_g1_i1_m.11379 | Putative glutathione S-transferase=*Pyropia yezoensis* | N/A | 75 | 126 | 12 |
| 60 | TRINITY_DN10074_c3_g5_i1_m.103412 | Na+-ATPase=*Pyropia yezoensis* | N/A | 22 | 60 | 22 |
| 61 | TRINITY_DN10030_c5_g10_i1_m.104223 | Pyrophosphate--fructose-6-phosphate 1-phosphotransferase=*Galdieria sulphuraria* | Gasu_20900 | 34 | 85 | 13 |
| 62 | TRINITY_DN10356_c1_g2_i1_m.114383 | Aminomethyltransferase =*Chondrus crispus* | CHC_T00008727001 | 38 | 87 | 11 |
| 63 | TRINITY_DN9517_c0_g2_i1_m.25503 | Alkene reductase=*Prochlorothrix hollandica* | N/A | 55 | 58 | 15 |
| 64 | TRINITY_DN8808_c0_g1_i1_m.9575 | Short-chain dehydrogenase=*Mucilaginibacter sp. PAMC 26640* | N/A | 62 | 54 | 10 |
| 65 | TRINITY_DN9917_c0_g1_i2_m.45015 | Photosystem II 12 kDa extrinsic protein, chloroplastic=*Griffithsia japonica* | N/A | 55 | 114 | 6 |
| 66 | TRINITY_DN9959_c6_g3_i1_m.42962 | Acetolactate synthase large subunit=*Galdieria sulphuraria* | Gasu_12440 | 38 | 51 | 11 |
| 67 | TRINITY_DN13885_c0_g1_i1_m.46848 | Photosystem II protein D1 | PsbA | 26 | 171 | 9 |
| 68 | TRINITY_DN10580_c0_g1_i1_m.54592 | UDP-glucose dehydrogenase=*Chondrus crispus* | CHC_T00008869001 | 39 | 64 | 1 |
| 69 | TRINITY_DN9347_c0_g1_i1_m.66749 | Small GTPase rab11-3=*Gracilariopsis lemaneiformis* | N/A | 71 | 99 | 12 |
| 70 | TRINITY_DN10235_c0_g2_i2_m.32025 | Phycocyanin operon protein Z=*Gloeobacter violaceus* | N/A | 59 | 39 | 9 |
| 71 | TRINITY_DN10144_c3_g7_i2_m.74876 | Hypothetical protein MAPG_02531=*Magnaporthiopsis poae ATCC 64411* | N/A | 42 | 29 | 9 |
| 72 | TRINITY_DN10450_c2_g16_i1_m.4584 | HAD-like hydrolase family protein=*Chondrus crispus* | CHC_T00008449001 | 65 | 50 | 11 |
| 73 | TRINITY_DN10237_c1_g3_i1_m.32438 | Unnamed protein product=*Chondrus crispus* | CHC_T00002546001 | 17 | 42 | 16 |
| 74 | TRINITY_DN10099_c7_g5_i1_m.105576 | PREDICTED: venom protease isoform X1=*Tribolium castaneum* | LOC103313165 | 33 | 51 | 7 |
| 75 | TRINITY_DN9851_c4_g7_i1_m.96050 | Acid-thiol ligase=*Chondrus crispus* | CHC_T00008318001 | 40 | 39 | 14 |
| 76 | TRINITY_DN10396_c0_g1_i1_m.116048 | Dihydrodipicolinate synthase=*Galdieria sulphuraria* | Gasu_29240 | 46 | 62 | 12 |
| 77 | TRINITY_DN10565_c0_g2_i1_m.58673 | Unnamed protein product=*Chondrus crispus* | CHC_T00005486001 | 50 | 90 | 8 |
| 78 | TRINITY_DN10500_c1_g15_i1_m.56242 | Putative chloroplast 4-hydroxy-3-methylbut-2-en-1-yl diphosphate synthase precursor=*Pyropia yezoensis* | N/A | 34 | 57 | 13 |
| 79 | TRINITY_DN10110_c1_g2_i1_m.71284 | Hypothetical protein MNEG_7049=*Monoraphidium neglectum* | MNEG_7049 | 39 | 51 | 6 |
| 80 | TRINITY_DN9910_c0_g2_i1_m.37534 | Unnamed protein product=*Chondrus crispus* | CHC_T00003004001 | 35 | 39 | 9 |
| 81 | TRINITY_DN10579_c3_g1_i1_m.61114 | Hypothetical protein GUITHDRAFT_72700=*Guillardia theta CCMP2712* | GUITHDRAFT_72700 | 12 | 74 | 3 |
| 82 | TRINITY_DN10243_c0_g2_i1_m.28117 | Heoxyhypusine synthase=*Klebsormidium flaccidum* | N/A | 40 | 20 | 10 |
| 83 | TRINITY_DN10637_c2_g2_i1_m.20953 | Unnamed protein product=*Chondrus crispus* | CHC_T00005217001 | 27 | 68 | 8 |
| 84 | TRINITY_DN8301_c0_g1_i1_m.90058 | 20S proteasome subunit alpha 4 | Gasu_20880 | 50 | 44 | 10 |
| 85 | TRINITY_DN9829_c0_g3_i1_m.94185 | Short-chain dehydrogenase=*Actinobacteria bacterium OK074* | N/A | 45 | 45 | 9 |
| 86 | TRINITY_DN10191_c6_g2_i1_m.73050 | Unnamed protein product=*Chondrus crispus* | CHC_T00002514001 | 49 | 67 | 11 |
| 87 | TRINITY_DN10348_c3_g4_i3_m.110869 | Ferredoxin component=*Chondrus crispus* | CHC_T00009355001 | 22 | 187 | 5 |
| 88 | TRINITY_DN10203_c6_g3_i1_m.30476 | CDGSH iron sulfur domain-containing protein 2=*Galdieria sulphuraria* | Gasu_46730 | 43 | 97 | 7 |
| 89 | TRINITY_DN10466_c4_g12_i1_m.3214 | PBS lyase HEAT domain protein repeat-containing protein=*Stanieria sp. NIES-3757* | N/A | 56 | 93 | 11 |
| 90 | TRINITY_DN10639_c3_g1_i2_m.19145 | ElR domain-containing protein=*Salpingoeca rosetta* | PTSG_10592 | 45 | 55 | 8 |
| 91 | TRINITY_DN10726_c2_g9_i1_m.118718 | Unnamed protein product=*Chondrus crispus* | CHC_T00007975001 | 38 | 41 | 11 |
| 92 | TRINITY_DN9274_c0_g1_i1_m.116760 | PREDICTED: peptidyl-prolyl cis-trans isomerase B-like=*Amphimedon queenslandica* | LOC100633079 | 45 | 113 | 8 |
| 93 | TRINITY_DN9277_c0_g1_i1_m.116686 | Unnamed protein product=*Chondrus crispus* | CHC_T00001115001 | 39 | 44 | 11 |
| 94 | TRINITY_DN7664_c0_g1_i1_m.68143 | Indole-3-glycerol phosphate synthase=*Chondrus crispus* | CHC_T00008561001 | 19 | 36 | 5 |
| 95 | TRINITY_DN9135_c0_g1_i1_m.79053 | Peroxisomal membrane MPV17/PMP22-like protein | Gasu_17910 | 63 | 51 | 8 |
| 96 | TRINITY_DN9911_c1_g1_i1_m.44192 | Unnamed protein product=*Chondrus crispus* | CHC_T00007692001 | 40 | 128 | 8 |
| 97 | TRINITY_DN10347_c0_g6_i1_m.110036 | Protein-serine/threonine kinases Rhodoplastic=*Chondrus crispus* | CHC_T00009242001 | 29 | 28 | 10 |
| 98 | TRINITY_DN13145_c0_g1_i1_m.68657 | Putative rubisco expression protein=*Pyropia perforata* | CbbX | 65 | 56 | 6 |
| 99 | TRINITY_DN8593_c0_g1_i1_m.91502 | Hypothetical protein Gasu_13160=*Galdieria sulphuraria* | Gasu_13160 | 53 | 89 | 5 |
| 100 | TRINITY_DN10697_c3_g2_i1_m.12820 | Peptidase=*Nannochloropsis gaditana* | N/A | 26 | 69 | 9 |
| 101 | TRINITY_DN9390_c0_g1_i1_m.67347 | Ubiquinol-cytochrome c reductase iron-sulfur subunit=*Chondrus crispus* | CHC_T00008759001 | 43 | 32 | 7 |
| 102 | TRINITY_DN10666_c2_g2_i1_m.20364 | 4-hydroxyphenylpyruvate dioxygenase, 4HPPD=*Chondrus crispus* | CHC_T00009321001 | 24 | 31 | 7 |
| 103 | TRINITY_DN7648_c0_g2_i1_m.68234 | Maleylacetoacetate isomerase=*Cystobacter fuscus* | N/A | 68 | 21 | 6 |
| 104 | TRINITY_DN9999_c6_g1_i1_m.40167 | Repeat protein=*Collinsella sp. CAG:398* | N/A | 30 | 28 | 8 |
| 105 | TRINITY_DN9057_c0_g1_i1_m.84045 | TCP-1/cpn60 chaperonin family protein=Klebsormidium flaccidum=*Galdieria sulphuraria* | N/A | 25 | 25 | 9 |
| 106 | TRINITY_DN10275_c0_g8_i2_m.29040 | HAD-superfamily hydrolase, subfamily IA, variant 3=*Saccharothrix espanaensis DSM 44229* | N/A | 24 | 31 | 7 |
| 107 | TRINITY_DN10195_c1_g27_i1_m.73472 | Protein of unknown function DUF1499=*Nannochloropsis gaditana* | N/A | 36 | 31 | 3 |
| 108 | TRINITY_DN7036_c0_g1_i1_m.22409 | Unnamed protein product=*Chondrus crispus* | CHC_T00003041001 | 23 | 31 | 9 |
| 109 | TRINITY_DN10751_c6_g1_i1_m.121172 | Ferrochelatase=*Chondrus crispus* | CHC_T00008918001 | 41 | 47 | 10 |
| 110 | TRINITY_DN10425_c2_g6_i1_m.7901 | PREDICTED: divinyl chlorophyllide a 8-vinyl-reductase, chloroplastic=*Jatropha curcas* | LOC105631824 | 32 | 22 | 4 |
| 111 | TRINITY_DN10716_c0_g1_i10_m.122406 | Actin=*Bicyclus anynana* | LOC112049886 | 33 | 12 | 1 |
| 112 | TRINITY_DN10169_c2_g1_i1_m.72787 | Unnamed protein product=*Vitrella brassicaformis CCMP3155* | N/A | 11 | 33 | 7 |
| 113 | TRINITY_DN10241_c2_g3_i1_m.34391 | Peptidylprolyl isomerase=*Galdieria sulphuraria* | Gasu_42850 | 33 | 21 | 9 |
| 114 | TRINITY_DN10295_c0_g4_i2_m.28025 | Unnamed protein product=*Chondrus crispus* | CHC_T00006717001 | 51 | 48 | 9 |
| 115 | TRINITY_DN9364_c0_g1_i1_m.67040 | Electron transfer flavoprotein subunit beta=*Ideonella sp. B508-1* | N/A | 30 | 21 | 6 |
| 116 | TRINITY_DN10593_c0_g2_i4_m.59078 | PREDICTED: chymotrypsin-like protease CTRL-1=*Microtus ochrogaster* | N/A | 35 | 14 | 5 |
| 117 | TRINITY_DN10105_c1_g1_i1_m.69158 | Unnamed protein product=*Chondrus crispus* | CHC_T00003973001 | 29 | 36 | 7 |
| 118 | TRINITY_DN9846_c1_g29_i1_m.94735 | Unnamed protein product=*Chondrus crispus* | CHC_T00001598001 | 41 | 20 | 9 |
| 119 | TRINITY_DN10207_c0_g6_i2_m.34594 | Low molecular mass early light-inducible protein HV60=*Griffithsia japonica* | N/A | 26 | 61 | 5 |
| 120 | TRINITY_DN10268_c4_g2_i1_m.34104 | Unnamed protein product=*Chondrus crispus* | N/A | 26 | 23 | 4 |
| 121 | TRINITY_DN10359_c6_g3_i1_m.114777 | Nitrate ABC transporter ATP-binding protein=*Cyanidioschyzon merolae strain 10D* | N/A | 19 | 21 | 6 |
| 122 | TRINITY_DN10620_c2_g2_i1_m.14118 | HAD-superfamily hydrolase=*Chondrus crispus* | CHC_T00008946001 | 31 | 25 | 6 |
| 123 | TRINITY_DN9954_c1_g1_i2_m.38034 | 20S core proteasome subunit alpha 1=*Chondrus crispus* | CHC_T00009373001 | 38 | 31 | 8 |
| 124 | TRINITY_DN10261_c0_g1_i3_m.29533 | Phasin protein=*Rhodobacteraceae bacterium HLUCCA08* | N/A | 41 | 8 | 1 |
| 125 | TRINITY_DN10209_c6_g1_i1_m.35544 | Hypothetical protein Gasu_59000=*Galdieria sulphuraria* | Gasu_59000 | 38 | 30 | 7 |
| 126 | TRINITY_DN10433_c3_g1_i2_m.7736 | Hypothetical protein=*Cupriavidus sp. HP* | N/A | 28 | 11 | 1 |
| 127 | TRINITY_DN10535_c3_g3_i1_m.59418 | B-cell receptor-associated protein 31-like protein=*Galdieria sulphuraria* | Gasu_39720 | 37 | 32 | 5 |
| 128 | TRINITY_DN10505_c7_g1_i1_m.57531 | Unnamed protein product*=Vitrella brassicaformis CCMP3155* | N/A | 27 | 30 | 6 |
| 129 | TRINITY_DN12155_c0_g1_i1_m.86526 | Unknown=*Picea sitchensis* | N/A | 34 | 11 | 6 |
| 130 | TRINITY_DN10701_c0_g3_i1_m.124156 | Exportin 1 | CHC_T00009059001 | 9 | 23 | 7 |
| 131 | TRINITY_DN9829_c0_g2_i1_m.94170 | Short-chain dehydrogenase=*Actinobacteria bacterium OK074* | N/A | 31 | 25 | 5 |
| 132 | TRINITY_DN8568_c0_g1_i1_m.90887 | Hypothetical protein, conserved=*Cyanidioschyzon merolae strain 10D* | CYME_CMC040C | 19 | 34 | 6 |
| 133 | TRINITY_DN10395_c1_g2_i1_m.115451 | WD40-repeat containing protein=*Chondrus crispus* | CHC_T00008529001 | 22 | 27 | 6 |
| 134 | TRINITY_DN10393_c5_g2_i1_m.111775 | Glutaredoxin=*Spizellomyces punctatus DAOM BR117* | SPPG_00747 | 38 | 19 | 4 |
| 135 | TRINITY_DN9939_c1_g1_i1_m.41298 | 9-cis-epoxycarotenoid dioxygenase, neoxanthin cleavage enzyme-like protein=*Cyanidioschyzon merolae strain 10D* | CYME_CMS362C | 20 | 17 | 6 |
| 136 | TRINITY_DN10484_c0_g1_i1_m.1555 | Translation initiation factor eIF4, subunit G=*Chondrus crispus* | CHC_T00008675001 | 20 | 20 | 5 |
| 137 | TRINITY_DN9967_c4_g1_i1_m.43495 | Unnamed protein product=*Chondrus crispus* | CHC_T00003309001 | 33 | 26 | 7 |
| 138 | TRINITY_DN9652_c0_g2_i1_m.98670 | Unnamed protein product=*Chondrus crispus* | CHC_T00004290001 | 29 | 43 | 7 |
| 139 | TRINITY_DN358_c0_g1_i1_m.126242 | Asparagine--tRNA ligase, chloroplast precursor=*Chondrus crispus* | CHC_T00008942001 | 22 | 24 | 7 |
| 140 | TRINITY_DN10751_c7_g3_i1_m.121201 | Isocitrate dehydrogenase | Gasu_48810 | 24 | 20 | 7 |
| 141 | TRINITY_DN10705_c2_g12_i1_m.124268 | Unnamed protein product=*Chondrus crispus* | CHC_T00007705001 | 14 | 14 | 4 |
| 142 | TRINITY_DN4975_c0_g1_i1_m.80927 | Oxidative stress-dependent heat shock protein 33, GWD=*Chondrus crispus* | CHC_T00010293001 | 29 | 22 | 7 |
| 143 | TRINITY_DN10363_c2_g6_i1_m.108288 | Similar to histidinol-phosphate aminotransferase=*Chondrus crispus* | CHC_T00008605001 | 25 | 31 | 8 |
| 144 | TRINITY_DN10017_c4_g7_i1_m.105717 | Cyclin delta-3=*Nannochloropsis gaditana* | N/A | 28 | 19 | 4 |
| 145 | TRINITY_DN10730_c2_g8_i1_m.121836 | Unnamed protein product=*Chondrus crispus* | CHC_T00004887001 | 15 | 14 | 8 |
| 146 | TRINITY_DN9424_c0_g3_i1_m.23833 | Glutamate N-acetyltransferase=*Galdieria sulphuraria* | Gasu_08880 | 8 | 31 | 5 |
| 147 | TRINITY_DN10631_c0_g1_i3_m.16917 | Protein fucoxanthin chlorophyll a/c protein=*Phaeodactylum tricornutum CCAP 1055/1* | Lhcf15 | 28 | 12 | 2 |
| 148 | TRINITY_DN10275_c0_g1_i1_m.29005 | Unnamed protein product=*Chondrus crispus* | CHC_T00000290001 | 11 | 17 | 4 |
| 149 | TRINITY_DN9799_c1_g7_i2_m.50272 | Glutathione S-transferase=*Caenispirillum salinarum AK4* | N/A | 11 | 14 | 4 |
| 150 | TRINITY_DN10037_c0_g2_i1_m.104437 | Probable inositol 2-dehydrogenase=*Chondrus crispus* | CHC_T00009082001 | 20 | 18 | 7 |
| 151 | TRINITY_DN13791_c0_g1_i1_m.88043 | Clp protease ATP binding subunit=*Pyropia fucicola* | N/A | 74 | 42 | 6 |
| 152 | TRINITY_DN10218_c3_g3_i1_m.34865 | Tyrosine--tRNA ligase, mitochondrial or chloroplast=*Chondrus crispus* | CHC_T00009128001 | 25 | 27 | 6 |
| 153 | TRINITY_DN10227_c2_g3_i1_m.32614 | Unnamed protein product=*Chondrus crispus* | CHC_T00005778001 | 24 | 14 | 5 |
| 154 | TRINITY_DN10241_c1_g4_i2_m.34376 | SCP-like extracellular protein=*Phytophthora infestans T30-4* | PITG_10035 | 53 | 19 | 4 |
| 155 | TRINITY_DN10177_c4_g6_i1_m.71669 | Hypothetical protein VOLCADRAFT_76143=*Volvox carteri f. nagariensis* | VOLCADRAFT_76143 | 17 | 14 | 8 |
| 156 | TRINITY_DN10433_c3_g3_i1_m.7745 | Hypothetical protein=*Tolypothrix sp. PCC 7601* | N/A | 21 | 19 | 4 |
| 157 | TRINITY_DN9785_c7_g3_i1_m.49954 | Unnamed protein product=*Chondrus crispus* | CHC_T00001105001 | 29 | 18 | 3 |
| 158 | TRINITY_DN9882_c3_g1_i2_m.96267 | Geranylgeranyl diphosphate synthase=*Porphyra umbilicalis* | N/A | 29 | 20 | 5 |
| 159 | TRINITY_DN10195_c1_g2_i1_m.73290 | Unnamed protein product=*Chondrus crispus* | CHC_T00000167001 | 18 | 16 | 4 |
| 160 | TRINITY_DN10006_c4_g1_i1_m.102018 | Uncharacterized protein Dvir_GJ13097=*Drosophila virilis* | LOC6623776 | 15 | 9 | 4 |
| 161 | TRINITY_DN10529_c1_g1_i2_m.54814 | Unnamed protein product=*Chondrus crispus* | CHC_T00007160001 | 10 | 10 | 4 |
| 162 | TRINITY_DN9014_c0_g1_i1_m.83833 | Unnamed protein product=*Vitis vinifera* | LOC100263010 | 20 | 18 | 3 |
| 163 | TRINITY_DN10062_c4_g5_i1_m.107263 | 40S ribosomal protein S15=*Chroomonas mesostigmatica CCMP1168* | N/A | 31 | 34 | 3 |
| 164 | TRINITY_DN10423_c1_g5_i1_m.795 | 20S core proteasome subunit alpha 6=*Chondrus crispus* | CHC_T00009004001 | 21 | 9 | 4 |
| 165 | TRINITY_DN10225_c2_g3_i1_m.34962 | FG-GAP repeat domain protein=*uncultured marine thaumarchaeote KM3_186_G04* | N/A | 15 | 11 | 3 |
| 166 | TRINITY_DN9851_c0_g1_i1_m.95996 | Septin-like protein=*Chlamydomonas reinhardtii* | CHLRE_12g556250v5 | 25 | 21 | 5 |
| 167 | TRINITY_DN10639_c2_g3_i1_m.19048 | Diaminopimelate epimerase, chloroplastic=*Auxenochlorella protothecoides* | F751_4193 | 25 | 11 | 4 |
| 168 | TRINITY_DN7476_c0_g1_i3_m.36994 | Hypothetical protein H257_02801=*Aphanomyces astaci* | H257_02801 | 45 | 16 | 4 |
| 169 | TRINITY_DN8507_c0_g2_i1_m.90847 | Unnamed protein product=*Chondrus crispus* | CHC_T00006620001 | 15 | 9 | 4 |
| 170 | TRINITY_DN5368_c0_g2_i1_m.89257 | Hypothetical protein Gasu_37840=*Galdieria sulphuraria* | Gasu_37840 | 21 | 26 | 3 |
| 171 | TRINITY_DN10631_c0_g1_i4_m.16923 | Protein fucoxanthin chlorophyll a/c protein=*Phaeodactylum tricornutum CCAP 1055/1* | Lhcf15 | 85 | 9 | 1 |
| 172 | TRINITY_DN9989_c0_g4_i1_m.43652 | Signal recognition particle SRP54 homolog ffh=*Chondrus crispus* | CHC_T00009028001 | 14 | 11 | 4 |
| 173 | TRINITY_DN9482_c0_g3_i1_m.23459 | Ribosomal 60S subunit protein L23B=*Rhizophagus irregularis DAOM 197198w* | N/A | 29 | 13 | 4 |
| 174 | TRINITY_DN10634_c0_g5_i4_m.13321 | Putative plastid 1-deoxy-D-xylulose 5-phosphate synthase precursor=*Pyropia yezoensis* | N/A | 12 | 10 | 5 |
| 175 | TRINITY_DN10238_c4_g2_i2_m.27806 | Hypothetical protein=*Mastigocoleus testarum* | N/A | 10 | 16 | 5 |
| 176 | TRINITY_DN10028_c3_g4_i2_m.100639 | Hypothetical protein GUITHDRAFT_162600=*Guillardia theta CCMP2712* | GUITHDRAFT_162600 | 33 | 15 | 3 |
| 177 | TRINITY_DN10115_c4_g1_i1_m.75595 | Hsp90 co-chaperone p23=*Cyanidioschyzon merolae strain 10D* | CYME_CMQ190C | 21 | 9 | 2 |
| 178 | TRINITY_DN10643_c3_g9_i1_m.14024 | PREDICTED: 1-phosphatidylinositol 4,5-bisphosphate phosphodiesterase delta-3-A-like=*Poecilia latipinna* | plcd3b | 6 | 7 | 2 |
| 179 | TRINITY_DN9728_c0_g10_i1_m.48132 | Unnamed protein product=*Chondrus crispus* | CHC_T00006226001 | 26 | 4 | 2 |
| 180 | TRINITY_DN1968_c0_g1_i1_m.83229 | Hypothetical protein=*Thiorhodovibrio sp.970* | N/A | 20 | 10 | 2 |
| 181 | TRINITY_DN10304_c0_g2_i1_m.112210 | Casein kinase I=*Chondrus crispus* | CHC_T00008932001 | 17 | 11 | 4 |
| 182 | TRINITY_DN8185_c0_g1_i1_m.51106 | Alpha-amylase=*Calothrix sp. PCC 7507* | N/A | 14 | 13 | 5 |
| 183 | TRINITY_DN10633_c2_g5_i1_m.20740 | Unnamed protein product=*Chondrus crispus* | N/A | 21 | 4 | 2 |
| 184 | TRINITY_DN9856_c7_g2_i1_m.97239 | Unnamed protein product=*Chondrus crispus* | CHC_T00004601001 | 17 | 12 | 3 |
| 185 | TRINITY_DN9916_c3_g12_i1_m.43898 | Neutral alpha-glucosidase AB=*Chondrus crispus* | CHC_T00008497001 | 10 | 8 | 4 |
| 186 | TRINITY_DN9131_c0_g1_i1_m.79796 | Unnamed protein product=*Chondrus crispus* | N/A | 20 | 4 | 4 |
| 187 | TRINITY_DN7613_c0_g1_i1_m.67985 | Hypothetical protein, conserved=*Cyanidioschyzon merolae strain 10D* | CYME_CML200C | 25 | 14 | 2 |
| 188 | TRINITY_DN4672_c0_g1_i1_m.65412 | Translation initiation factor eIF2, subunit alpha=*Chondrus crispus* | CHC_T00009249001 | 17 | 5 | 4 |
| 189 | TRINITY_DN7442_c0_g2_i1_m.37288 | MULTISPECIES: endonuclease=*Cyanothece* | N/A | 18 | 10 | 3 |
| 190 | TRINITY_DN10695_c1_g2_i1_m.17362 | Methyltransferase FkbM family=*Oscillatoriales cyanobacterium JSC-12* | N/A | 15 | 4 | 3 |
| 191 | TRINITY_DN10308_c2_g2_i2_m.107713 | Histone H2A=*Chondrus crispus* | CHC_T00009247001 | 16 | 20 | 3 |
| 192 | TRINITY_DN7473_c0_g1_i1_m.37236 | Expressed unknown protein=*Ectocarpus siliculosus* | N/A | 26 | 10 | 3 |
| 193 | TRINITY_DN9160_c0_g1_i1_m.79692 | Unnamed protein product=*Chondrus crispus* | CHC_T00006923001 | 42 | 11 | 3 |
| 194 | TRINITY_DN9947_c0_g1_i1_m.38747 | Unnamed protein product=*Chondrus crispus* | N/A | 14 | 7 | 4 |
| 195 | TRINITY_DN6805_c0_g1_i1_m.85959 | Unnamed protein product=*Chondrus crispus* | CHC_T00000550001 | 13 | 8 | 4 |
| 196 | TRINITY_DN9854_c5_g1_i1_m.93020 | Hypothetical protein RSOL_067030=*Rhizoctonia solani AG-3 Rhs1AP* | N/A | 8 | 14 | 3 |
| 197 | TRINITY_DN10342_c3_g4_i1_m.115289 | RNA recognition motif | Gasu_28750 | 14 | 19 | 2 |
| 198 | TRINITY_DN9308_c0_g1_i1_m.66906 | Cystathionine beta-lyase METC=*Chondrus crispus* | CHC_T00008829001 | 19 | 6 | 4 |
| 199 | TRINITY_DN9669_c5_g1_i1_m.98755 | Hypothetical protein Gasu_61470=*Galdieria sulphuraria* | Gasu_61470 | 32 | 6 | 3 |
| 200 | TRINITY_DN10155_c0_g2_i2_m.70775 | Hypothetical protein VOLCADRAFT_93964=*Volvox carteri f. nagariensis* | VOLCADRAFT_93964 | 7 | 11 | 3 |
| 201 | TRINITY_DN14417_c0_g1_i1_m.86608 | Histidine kinase=*Dermacoccus nishinomiyaensis* | HX89_RS06960 | 32 | 9 | 3 |
| 202 | TRINITY_DN6500_c0_g1_i1_m.88882 | Unnamed protein product=*Chondrus crispus* | CHC_T00003111001 | 19 | 4 | 4 |
| 203 | TRINITY_DN9770_c0_g1_i2_m.49000 | Translocon-associated protein alpha subunit=*Blastocystis sp. Nand II* | N/A | 16 | 6 | 3 |
| 204 | TRINITY_DN10567_c2_g1_i1_m.60549 | Unnamed protein product=*Chondrus crispus* | CHC_T00006428001 | 21 | 6 | 3 |
| 205 | TRINITY_DN10414_c5_g8_i1_m.5228 | Hypothetical protein, conserved =*Cyanidioschyzon merolae strain 10D* | CYME_CMM247C | 28 | 3 | 2 |
| 206 | TRINITY_DN10611_c5_g6_i1_m.14547 | Flavocytochrome c, variant=*Allomyces macrogynus ATCC 38327* | N/A | 24 | 3 | 2 |
| 207 | TRINITY_DN10002_c1_g3_i3_m.105027 | Light-harvesting protein=*Pyropia yezoensis* | N/A | 20 | 14 | 2 |
| 208 | TRINITY_DN7660_c0_g1_i1_m.67813 | Unnamed protein product=*Chondrus crispus* | CHC_T00003467001 | 10 | 9 | 2 |
| 209 | TRINITY_DN9918_c3_g2_i1_m.42599 | Hypothetical protein CYME_CMT076C=*Cyanidioschyzon merolae strain 10D* | CYME_CMT076C | 22 | 7 | 2 |
| 210 | TRINITY_DN9921_c0_g1_i1_m.44347 | Glyceraldehyde-3-phosphate dehydrogenase precursor=*Phaeodactylum tricornutum CCAP 1055/1* | GapC1 | 20 | 15 | 3 |
| 211 | TRINITY_DN10679_c2_g1_i1_m.13830 | Unnamed protein product=*Chondrus crispus* | CHC_T00007328001 | 10 | 6 | 3 |
| 212 | TRINITY_DN10097_c2_g2_i1_m.101407 | Fructose-1,6-bisphosphatase I=*Galdieria sulphuraria* | Gasu_02220 | 8 | 7 | 2 |
| 213 | TRINITY_DN10629_c0_g1_i1_m.15360 | Unnamed protein product=*Chondrus crispus* | CHC_T00007985001 | 28 | 4 | 3 |
| 214 | TRINITY_DN7992_c0_g1_i1_m.9932 | U6 snRNA-associated Sm-like protein=*Plasmopara halstedii* | PHALS_11230 | 25 | 3 | 2 |
| 215 | TRINITY_DN10608_c3_g4_i1_m.21071 | Probable cyclophilin b=*Sporisorium reilianum SRZ2* | N/A | 13 | 5 | 3 |
| 216 | TRINITY_DN10404_c3_g4_i1_m.238 | Unnamed protein product=*Chondrus crispus* | CHC_T00001826001 | 12 | 3 | 1 |
| 217 | TRINITY_DN10135_c6_g1_i1_m.71760 | Hypothetical protein CYME_CMG185C=*Cyanidioschyzon merolae strain 10D* | CYME_CMG185C | 16 | 4 | 3 |
| 218 | TRINITY_DN421_c0_g1_i1_m.64082 | Hypothetical protein GLOINDRAFT_336065=*Rhizophagus irregularis DAOM 181602* | N/A | 13 | 7 | 2 |
| 219 | TRINITY_DN8512_c0_g2_i1_m.91575 | Unnamed protein product=*Chondrus crispus* | CHC_T00002260001 | 11 | 2 | 1 |
| 220 | TRINITY_DN9965_c3_g1_i1_m.38118 | Mitogen-activated protein kinase 6=*Pyropia yezoensis* | N/A | 7 | 11 | 1 |
| 221 | TRINITY_DN10741_c3_g21_i1_m.120634 | Hypothetical protein PHYSODRAFT_505357=*Phytophthora sojae* | PHYSODRAFT_505357 | 10 | 3 | 2 |
| 222 | TRINITY_DN8419_c0_g1_i1_m.45776 | Peptidyl-prolyl cis-trans isomerase B | N/A | 21 | 44 | 1 |
| 223 | TRINITY_DN9972_c1_g1_i1_m.37568 | PREDICTED: acetyl-CoA acetyltransferase, mitochondrial isoform X2=*Serinus canaria* | N/A | 20 | 7 | 2 |
| 224 | TRINITY_DN10478_c1_g1_i2_m.3733 | Hypothetical protein BRAFLDRAFT_128223=*Branchiostoma floridae* | N/A | 31 | 11 | 2 |
| 225 | TRINITY_DN10067_c5_g1_i1_m.100108 | Aspartyl/glutamyl-tRNA amidotransferase subunit B=*Neosynechococcus sphagnicola* | N/A | 22 | 7 | 2 |
| 226 | TRINITY_DN9683_c2_g1_i2_m.98203 | Unnamed protein product=*Chondrus crispus* | CHC_T00007335001 | 6 | 7 | 2 |
| 227 | TRINITY_DN10532_c1_g3_i1_m.60761 | Unnamed protein product=*Chondrus crispus* | CHC_T00001399001 | 8 | 8 | 2 |
| 228 | TRINITY_DN8546_c0_g2_i1_m.90930 | Similar to aspartyl beta-hydroxylase=*Cyanidioschyzon merolae* *strain 10D* | CYME_CMQ214C | 15 | 3 | 3 |
| 229 | TRINITY_DN8911_c0_g1_i1_m.62738 | Hypothetical protein, conserved=*Cyanidioschyzon merolae strain 10D* | CYME_CMM306C | 4 | 11 | 1 |
| 230 | TRINITY_DN9466_c0_g1_i1_m.23560 | Poly-A RNA export protein DBP5=*Chondrus crispus* | CHC_T00009449001 | 7 | 6 | 3 |
| 231 | TRINITY_DN9737_c0_g3_i1_m.48785 | Unnamed protein product=*Chondrus crispus* | CHC_T00007173001 | 5 | 3 | 2 |
| 232 | TRINITY_DN10098_c3_g1_i1_m.101139 | Glycerol kinase=*Pyropia haitanensis* | N/A | 8 | 3 | 3 |
| 233 | TRINITY_DN10272_c3_g4_i1_m.32945 | Unnamed protein product=*Chondrus crispus* | CHC_T00007243001 | 23 | 5 | 3 |
| 234 | TRINITY_DN9642_c1_g1_i1_m.98533 | Chloroplast sedoheptulose-1,7-bisphosphatase=*Pyropia yezoensis* | N/A | 11 | 5 | 1 |
| 235 | TRINITY_DN10676_c6_g10_i1_m.12984 | Unnamed protein product=*Chondrus crispus* | CHC_T00002318001 | 4 | 6 | 2 |
| 236 | TRINITY_DN10016_c0_g1_i2_m.105069 | Hypothetical protein AMAG_02467=*Allomyces macrogynus ATCC 38327* | N/A | 24 | 3 | 1 |
| 237 | TRINITY_DN10270_c2_g8_i1_m.28520 | Protoporphyrinogen oxidase=*Gemmatimonas phototrophica* | N/A | 5 | 4 | 2 |
| 238 | TRINITY_DN12896_c0_g1_i1_m.117700 | RuvB-like DNA/RNA helicase pontin=*Chondrus crispus* | CHC_T00008567001 | 7 | 4 | 2 |
| 239 | TRINITY_DN10093_c2_g1_i1_m.99436 | rRNA 2'-O-methyltransferase fibrillarin, putative=*Phytophthora infestans T30-4* | PITG_07308 | 10 | 7 | 3 |
| 240 | TRINITY_DN10449_c5_g3_i1_m.7525 | Unnamed protein product=*Chondrus crispus* | CHC_T00006303001 | 17 | 4 | 3 |
| 241 | TRINITY_DN10072_c7_g1_i1_m.102899 | Acid-thiol ligase=*Chondrus crispus* | CHC_T00008318001 | 11 | 7 | 1 |
| 242 | TRINITY_DN10490_c6_g5_i2_m.6960 | DUF3727 domain-containing protein=*Leptolyngbya sp.PCC6406* | N/A | 13 | 2 | 1 |
| 243 | TRINITY_DN9646_c0_g1_i1_m.99037 | Hypothetical protein X777_14195, partial=*Cerapachys biroi* | LOC105285905 | 12 | 3 | 2 |
| 244 | TRINITY_DN10334_c6_g3_i4_m.109325 | Hypothetical protein=*Elstera litoralis* | N/A | 6 | 1 | 1 |
| 245 | TRINITY_DN8316_c0_g1_i1_m.89980 | Unnamed protein product=*Chondrus crispus* | CHC_T00006636001 | 20 | 3 | 3 |
| 246 | TRINITY_DN10622_c2_g8_i1_m.15065 | Cop9 signalosome subunit 7=*Colletotrichum orbiculare MAFF 240422* | N/A | 17 | 2 | 2 |
| 247 | TRINITY_DN7108_c0_g1_i1_m.86916 | PREDICTED: glyceraldehyde-3-phosphate dehydrogenase-like=*Acropora digitifera* | LOC107332108 | 10 | 21 | 2 |
| 248 | TRINITY_DN9131_c0_g2_i1_m.79804 | Hypothetical protein =*Jiangella gansuensis* | N/A | 14 | 5 | 4 |
| 249 | TRINITY_DN10067_c1_g1_i1_m.100078 | Hypothetical protein =*Bacillus sp. AM 1* | N/A | 11 | 14 | 2 |
| 250 | TRINITY_DN10141_c6_g11_i1_m.76921 | Hypothetical protein KFL_000360150=*Klebsormidium flaccidum* | N/A | 26 | 6 | 2 |
| 251 | TRINITY_DN10349_c3_g6_i1_m.116121 | Unnamed protein product=*Chondrus crispus* | CHC_T00004887001 | 12 | 4 | 2 |
| 252 | TRINITY_DN10133_c8_g4_i1_m.69770 | Unnamed protein product=*Vitrella brassicaformis CCMP3155* | N/A | 6 | 2 | 2 |
| 253 | TRINITY_DN9894_c3_g2_i1_m.93982 | AMP-dependent synthetase=*Microbacterium sp. MEJ108Y* | N/A | 22 | 4 | 1 |
| 254 | TRINITY_DN9979_c4_g3_i1_m.44169 | MULTISPECIES: sulfurtransferase=*Cupriavidus* | N/A | 20 | 2 | 2 |
| 255 | TRINITY_DN7878_c0_g1_i1_m.82338 | Chlorophyll a synthase=*Chondrus crispus* | CHC_T00008377001 | 4 | 2 | 1 |
| 256 | TRINITY_DN8559_c0_g1_i1_m.91226 | Light-harvest protein=*Griffithsia japonica* | N/A | 6 | 3 | 1 |
| 257 | TRINITY_DN10408_c6_g12_i1_m.5949 | Protease inhibitor=*Salpingoeca rosetta* | PTSG_06045 | 6 | 2 | 1 |
| 258 | TRINITY_DN10380_c0_g4_i4_m.116505 | JC7151 vacuolar H+-ATPase | N/A | 11 | 6 | 1 |
| 259 | TRINITY_DN4776_c0_g1_i1_m.90669 | Dynein light chain Tctex-type=*Chondrus crispus* | CHC_T00009472001 | 8 | 3 | 1 |
| 260 | TRINITY_DN10672_c2_g1_i1_m.15710 | Unnamed protein product=*Vitrella brassicaformis CCMP3155* | N/A | 4 | 6 | 1 |
| 261 | TRINITY_DN6119_c0_g1_i1_m.85713 | Peptidyl-prolyl cis-trans isomerase C=*Galdieria sulphuraria* | Gasu_17550 | 10 | 2 | 2 |
| 262 | TRINITY_DN4449_c0_g1_i1_m.60 | TdcF protein=*Galdieria sulphuraria* | Gasu_39010 | 5 | 10 | 1 |
| 263 | TRINITY_DN10676_c6_g7_i1_m.12972 | Glutathione S-transferase=*Rubellimicrobium thermophilum* | N/A | 8 | 10 | 1 |
| 264 | TRINITY_DN10449_c3_g2_i1_m.7406 | Unknown hydrolase =*Cyanidioschyzon merolae strain 10D* | CYME_CMN100C | 16 | 1 | 1 |
| 265 | TRINITY_DN3003_c0_g1_i1_m.36290 | P450-type beta-carotene hydroxylase CYP97B29=*Porphyra umbilicalis* | N/A | 2 | 3 | 2 |
| 266 | TRINITY_DN10238_c5_g1_i3_m.27826 | Serine/threonine protein kinase=*Galdieria sulphuraria* | Gasu_50310 | 5 | 2 | 2 |
| 267 | TRINITY_DN2766_c0_g1_i1_m.89055 | Ribosomal protein S16 | Rps16 | 20 | 5 | 1 |
| 268 | TRINITY_DN7228_c0_g1_i1_m.8633 | Hypothetical protein SARC_08809=*Sphaeroforma arctica JP610* | SARC_08809 | 13 | 12 | 1 |
| 269 | TRINITY_DN7808_c0_g1_i1_m.82442 | PREDICTED: proteasome subunit alpha type-5-like isoform X2=*Lingula anatina* | LOC106177094 | 10 | 5 | 1 |
| 270 | TRINITY_DN10274_c1_g5_i1_m.35388 | Unnamed protein product=*Vitrella brassicaformis CCMP3155* | N/A | 8 | 4 | 1 |
| 271 | TRINITY_DN9965_c3_g8_i2_m.38170 | Hypothetical protein=*Chamaesiphon minutus* | N/A | 8 | 2 | 1 |
| 272 | TRINITY_DN10359_c5_g5_i1_m.114735 | 5-methyltetrahydropteroyltriglutamate--homocysteine methyltransferase=*Chondrus crispus* | CHC_T00009326001 | 4 | 2 | 2 |
| 273 | TRINITY_DN9842_c2_g13_i1_m.97446 | Hypothetical protein CISIN_1g021661mg=*Citrus sinensis* | N/A | 14 | 1 | 1 |
| 274 | TRINITY_DN9886_c2_g7_i1_m.94264 | Ribosomal protein S16 | THAPSDRAFT_5135 | 7 | 1 | 1 |
| 275 | TRINITY_DN10304_c1_g1_i2_m.112339 | CG7768=*Drosophila busckii* | LOC108598794 | 7 | 1 | 1 |
| 276 | TRINITY_DN10523_c0_g2_i2_m.59232 | Diphthine synthase=*Chondrus crispus* | CHC_T00008650001 | 11 | 6 | 2 |
| 277 | TRINITY_DN12081_c0_g1_i1_m.63959 | Mitochondrial DNA replication protein YHM2=*Cyanidioschyzon merolae strain 10D* | CYME_CMP013C | 7 | 1 | 1 |
| 278 | TRINITY_DN3314_c0_g1_i1_m.24980 | Unnamed protein product=*Chondrus crispus* | CHC_T00005150001 | 5 | 2 | 1 |
| 279 | TRINITY_DN10233_c4_g5_i1_m.29711 | Unnamed protein product=*Chondrus crispus* | CHC_T00004428001 | 7 | 2 | 1 |
| 280 | TRINITY_DN8119_c0_g1_i1_m.51073 | Phenylalanyl-tRNA synthetase alpha chain=*Chondrus crispus* | CHC_T00009560001 | 6 | 1 | 1 |
| 281 | TRINITY_DN10615_c5_g2_i1_m.16716 | Unnamed protein product=*Chondrus crispus* | CHC_T00005961001 | 2 | 1 | 1 |
| 282 | TRINITY_DN10338_c5_g1_i3_m.116356 | Unnamed protein product=*Vitrella brassicaformis CCMP3155* | N/A | 9 | 1 | 1 |
| 283 | TRINITY_DN10297_c3_g1_i1_m.33925 | Beta-lactamase=*Gemmatirosa kalamazoonesis* | N/A | 3 | 1 | 1 |
| 284 | TRINITY_DN10568_c1_g3_i2_m.58719 | Putative plastid light harvesting protein isoform 35=*Aureococcus anophagefferens* | LHC35 | 6 | 1 | 1 |
| 285 | TRINITY_DN9867_c2_g2_i1_m.93330 | Probable arginine methyltransferase | CHC_T00008919001 | 2 | 1 | 1 |
| 286 | TRINITY_DN1669_c0_g1_i1_m.64724 | DUF89/ Fructose-1,6-bisphosphatase=*Micromonas commoda* | FBPASE_2 | 8 | 8 | 1 |
| 287 | TRINITY_DN10234_c5_g1_i1_m.32747 | Farnesyl diphosphate synthase=*Chlamydomonas reinhardtii* | CHLRE_03g207700v5 | 4 | 1 | 1 |
| 288 | TRINITY_DN10696_c7_g5_i1_m.19009 | PREDICTED: glycogen synthase kinase-3 beta-like isoform X5=*Cephus cinctus* | N/A | 3 | 3 | 1 |
| 289 | TRINITY_DN10738_c1_g1_i3_m.120364 | Heat shock protein 90-1=*Pyropia haitanensis* | A1BG | 1 | 2 | 1 |
| 290 | TRINITY_DN10028_c2_g3_i1_m.100611 | Putative glutathione S-transferase=*Pyropia yezoensis* | N/A | 3 | 1 | 1 |
| 291 | TRINITY_DN10421_c1_g1_i1_m.1041 | Predicted protein=*Micromonas commoda* | MICPUN_98844 | 2 | 1 | 1 |
| 292 | TRINITY_DN9520_c1_g1_i1_m.25462 | ASPO2608=*Pyropia yezoensis* | N/A | 18 | 1 | 1 |
| 293 | TRINITY_DN10269_c3_g4_i1_m.35865 | Unnamed protein product=*Chondrus crispus* | CHC_T00003033001 | 5 | 3 | 1 |
| 294 | TRINITY_DN10412_c1_g2_i1_m.4786 | Hypothetical protein EMIHUDRAFT_103709=*Emiliania huxleyi CCMP1516* | EMIHUDRAFT_103709 | 21 | 1 | 1 |
| 295 | TRINITY_DN10055_c6_g3_i1_m.99912 | Mitogen-activated protein kinase 1=*Pyropia haitanensis* | N/A | 2 | 1 | 1 |
| 296 | TRINITY_DN8197_c0_g1_i1_m.50737 | Hypothetical protein SPPG_03224=*Spizellomyces punctatus DAOM BR117* | SPPG_03224 | 6 | 1 | 1 |
| 297 | TRINITY_DN5829_c0_g1_i1_m.24696 | PREDICTED: pentatricopeptide repeat-containing protein At4g11690=*Malus domestica* | LOC103420964 | 4 | 2 | 1 |
| 298 | TRINITY_DN10351_c5_g1_i1_m.107966 | Unnamed protein product=*Chondrus crispus* | CHC_T00000427001 | 1 | 4 | 1 |
| 299 | TRINITY_DN9974_c2_g9_i1_m.43347 | Unnamed protein product=*Chondrus crispus* | CHC_T00005806001 | 19 | 2 | 2 |
| 300 | TRINITY_DN9435_c0_g1_i2_m.23255 | Coatomer protein complex, subunit zeta=*Galdieria sulphuraria* | Gasu _ 30240 | 4 | 1 | 1 |
| 301 | TRINITY_DN10597_c2_g1_i1_m.59849 | Hypothetical protein PDIP_78200=*Penicillium digitatum Pd1* | PDIP_78200 | 11 | 2 | 1 |
| 302 | TRINITY_DN4809_c0_g1_i1_m.91826 | Putative 40S ribosomal protein RPS23=*Flustra foliacea* | N/A | 8 | 5 | 1 |
| 303 | TRINITY_DN15541_c0_g1_i1_m.125958 | Hypothetical protein, conserved=*Cyanidioschyzon merolae strain 10D* | CYME_CMD028C | 26 | 2 | 1 |
| 304 | TRINITY_DN10203_c2_g1_i2_m.30400 | Vitamin K epoxide reductase=*Nannochloropsis gaditana* | N/A | 8 | 2 | 1 |
| 305 | TRINITY_DN12116_c0_g1_i1_m.86313 | Putative 40S ribosomal protein RPS27=*Flustra foliaceaEmiliania huxleyi CCMP1516* | N/A | 9 | 5 | 1 |
| 306 | TRINITY_DN10417_c3_g7_i1_m.5570 | AGAP004880-PB=*Anopheles gambiae str. PEST* | AgaP_AGAP004880 | 9 | 3 | 2 |
| 307 | TRINITY_DN10221_c9_g10_i1_m.30865 | Monogalactosyldiacylglycerol synthase, family GT28=*Ectocarpus siliculosus* | N/A | 7 | 2 | 1 |
| 308 | TRINITY_DN10417_c3_g6_i2_m.5582 | Unnamed protein product=*Chondrus crispus* | CHC_T00001837001 | 4 | 1 | 1 |
| 309 | TRINITY_DN8898_c0_g1_i1_m.9428 | Hypothetical protein NGA_0717900=*Nannochloropsis gaditana CCMP526* | NGA_0717900 | 8 | 2 | 1 |
| 310 | TRINITY_DN10750_c3_g2_i1_m.119887 | Unnamed protein product=*Chondrus crispus* | CHC_T00007329001 | 2 | 1 | 1 |
| 311 | TRINITY_DN10705_c0_g1_i1_m.124209 | Unknown kinase with aarF domain=*Chondrus crispus* | CHC_T00009527001 | 2 | 1 | 1 |
| 312 | TRINITY_DN10094_c2_g17_i1_m.106827 | PREDICTED: cytoplasmic dynein 1 heavy chain 1-like isoform X1=*Lingula anatina* | LOC106167712 | 3 | 10 | 1 |
| 313 | TRINITY_DN10096_c1_g1_i1_m.104482 | Glyoxalase bleomycin resistance protein dioxygenase=*Nannochloropsis gaditana* | N/A | 9 | 1 | 1 |
| 314 | TRINITY_DN9652_c0_g2_i1_m.98668 | Unnamed protein product=*Chondrus crispus* | CHC_T00004960001 | 2 | 1 | 1 |
| 315 | TRINITY_DN7407_c0_g1_i1_m.37241 | Putrescine aminopropyltransferase | CHC_T00009140001 | 3 | 1 | 1 |
| 316 | TRINITY_DN10088_c0_g2_i1_m.101752 | Unnamed protein product=*Chondrus crispus* | CHC_T00000308001 | 18 | 2 | 1 |
| 317 | TRINITY_DN10025_c1_g2_i1_m.100836 | 60S ribosomal protein L37a=*Chondrus crispus* | CHC_T00010262001 | 7 | 7 | 1 |
| 318 | TRINITY_DN8844_c0_g1_i1_m.9409 | Unnamed protein product=*Chondrus crispus* | CHC_T00004683001 | 2 | 1 | 1 |
| 319 | TRINITY_DN10467_c3_g6_i1_m.2637 | Hypothetical protein=*Synechococcus sp. JA-3-3Ab* | N/A | 10 | 1 | 1 |
| 320 | TRINITY_DN10515_c5_g2_i1_m.57714 | Unnamed protein product=*Chondrus crispus* | CHC_T00005827001 | 3 | 1 | 1 |
| 321 | TRINITY_DN10197_c0_g3_i1_m.69418 | Corrinoid ABC transporter substrate-binding protein=*Planctomyces sp.SH-PL14* | N/A | 7 | 1 | 1 |
| 322 | TRINITY_DN10488_c2_g4_i1_m.2986 | Fucoxanthin chlorophyll a/c protein=*Fistulifera solaris* | N/A | 9 | 1 | 1 |
| 323 | TRINITY_DN12902_c0_g1_i1_m.26689 | Peptide deformylase=*Chrysochromulina sp.CCMP291* | N/A | 4 | 1 | 1 |
| 324 | TRINITY_DN13396_c0_g1_i1_m.87723 | Hypothetical protein OXYTRI_09166=*Oxytricha trifallax* | N/A | 10 | 1 | 1 |
| 325 | TRINITY_DN2551_c0_g1_i1_m.37384 | Ribosomal protein L20 | rpl20 | 8 | 1 | 1 |
| 326 | TRINITY_DN9995_c4_g1_i1_m.41454 | Histone demethylases | CHC_T00010075001 | 2 | 6 | 1 |
| 327 | TRINITY_DN10063_c1_g1_i1_m.101807 | Unnamed protein product=*Chondrus crispus* | CHC_T00001757001 | 1 | 1 | 1 |
| 328 | TRINITY_DN7950_c0_g1_i1_m.9985 | VAMP7a=*Chondrus crispus* | CHC_T00010136001 | 12 | 1 | 1 |
| 329 | TRINITY_DN10278_c3_g4_i1_m.28616 | Hypothetical protein GUITHDRAFT_76774, partial=*Guillardia theta CCMP2712* | GUITHDRAFT_76774 | 2 | 1 | 1 |
| 330 | TRINITY_DN7613_c0_g1_i1_m.67983 | Hypothetical protein DAPPUDRAFT_302806=*Daphnia pulex* | N/A | 5 | 1 | 1 |
| 331 | TRINITY_DN6572_c0_g2_i1_m.88910 | Adenylyl cyclase-associated protein 1=*Crassostrea gigas* | N/A | 26 | 2 | 1 |

(N=Number; FDR=False Discovery Rate; Cov.=Protein Coverage; PSM=Peptide Spectrum Matches; Uni. Pep.=Unique Peptides)
