# Supplementary material for: Bioinformatic Prediction and Characterization of Proteins in Porphyra dentata by Shotgun Proteomics
Source: Front Nutr. 2022 Jul 7;9:924524. doi: 10.3389/fnut.2022.924524 (PMC9301277; doi:10.3389/fnut.2022.924524)
Supplement: Supplementary file 3 [file Table_2.DOCX]

Supplementary Material

Table S2. Network analysis function color annotation.

| Cluster number | Cluster color | Gene count | Protein name | Protein identifier | Protein description |
| --- | --- | --- | --- | --- | --- |
| 1 | Red | 4 | R7QGK7 | 2769.R7QGK7 | Uncharacterized protein |
| 1 | Red | 4 | R7QHF5 | 2769.R7QHF5 | Phosphoinositide phospholipase C |
| 1 | Red | 4 | R7QKD2 | 2769.R7QKD2 | Uncharacterized protein |
| 1 | Red | 4 | R7QP80 | 2769.R7QP80 | Uncharacterized protein |
| 2 | Brown | 4 | R7Q5J9 | 2769.R7Q5J9 | Myo-inositol dehydrogenase |
| 2 | Brown | 4 | R7QG98 | 2769.R7QG98 | Probable inositol 2-dehydrogenase |
| 2 | Brown | 4 | R7QPZ3 | 2769.R7QPZ3 | Uncharacterized protein |
| 2 | Brown | 4 | R7QQI3 | 2769.R7QQI3 | Pyrophosphate--fructose 6-phosphate 1-phosphotransferase; Catalyzes the phosphorylation of D-fructose 6-phosphate, the first committing step of glycolysis. Uses inorganic phosphate (PPi) as phosphoryl donor instead of ATP like common ATP-dependent phosphofructokinases (ATP-PFKs), which renders the reaction reversible, and can thus function both in glycolysis and gluconeogenesis. Consistently, PPi-PFK can replace the enzymes of both the forward (ATP- PFK) and reverse (fructose-bisphosphatase (FBPase)) reactions. |
| 3 | Dark Golden Rod | 4 | R7Q9X0 | 2769.R7Q9X0 | 5-methyltetrahydropteroyltriglutamate--homocystei ne methyltransferase |
| 3 | Dark Golden Rod | 4 | R7QG86 | 2769.R7QG86 | Aminomethyltransferase; The glycine cleavage system catalyzes the degradation of glycine; Belongs to the GcvT family. |
| 3 | Dark Golden Rod | 4 | R7QK24 | 2769.R7QK24 | Cystathionine beta-lyase METC |
| 3 | Dark Golden Rod | 4 | S0F3I8 | 2769.S0F3I8 | S-adenosylmethionine synthase; Catalyzes the formation of S-adenosylmethionine from methionine and ATP. |
| 4 | Green | 3 | R7QF86 | 2769.R7QF86 | Putrescine aminopropyltransferase (Spermidine synthase); Belongs to the spermidine/spermine synthase family. |
| 4 | Green | 3 | R7QIH9 | 2769.R7QIH9 | Glucose-6-phosphate 1-dehydrogenase; Catalyzes the rate-limiting step of the oxidative pentose- phosphate pathway, which represents a route for the dissimilation of carbohydrates besides glycolysis. |
| 4 | Green | 3 | R7QV44 | 2769.R7QV44 | Isocitrate dehydrogenase [NAD] subunit, mitochondrial |
| 5 | Green 2 | 2 | R7Q4W3 | 2769.R7Q4W3 | Uncharacterized protein |
| 5 | Green 2 | 2 | R7QAF3 | 2769.R7QAF3 | Glutathione S-transferase; Belongs to the GST superfamily. |
| 6 | Cyan | 2 | R7Q958 | 2769.R7Q958 | Acetyl-CoA acetyltransferase; Belongs to the thiolase-like superfamily. Thiolase family. |
| 6 | Cyan | 2 | R7QAP7 | 2769.R7QAP7 | Phosphoglycerate kinase |
| 7 | Dark Cyan | 2 | R7QEC8 | 2769.R7QEC8 | Glyceraldehyde-3-phosphate dehydrogenase; Belongs to the glyceraldehyde-3-phosphate dehydrogenase family. |
| 7 | Dark Cyan | 2 | R7QNN6 | 2769.R7QNN6 | Triosephosphate isomerase |
| 8 | Blue | 1 | S0F2W5 | 2769.S0F2W5 | UDP-glucose dehydrogenase |
| 9 | Medium Blue | 1 | R7QFK1 | 2769.R7QFK1 | 4-hydroxyphenylpyruvate dioxygenase |
