# Supplementary material for: Bioinformatic Prediction and Characterization of Proteins in Porphyra dentata by Shotgun Proteomics
Source: Front Nutr. 2022 Jul 7;9:924524. doi: 10.3389/fnut.2022.924524 (PMC9301277; doi:10.3389/fnut.2022.924524)
Supplement: Supplementary file 4 [file Table_3.docx]

Supplementary Material

Table S3. Potential bioactive peptides predicted after pepsin or trypsin digestion.

S3-1.Potential bioactive peptides of the Amino acid metabolism-Pepsin.

| **Number** | **Sequence** |
| --- | --- |
| **1** | HGAPHVA |
| **1** | GGGVAGGCI |
| **1** | AHGGVQF |
| **1** | NSGAGGMGA |
| **1** | DQFTTI |
| **1** | NSGAGGMGA |
| **1** | SGNPSQY |
| **1** | TLPCVGY |
| **1** | HGGVQFL |
| **1** | RWGDGW |
| **1** | HRVNGQA |
| **1** | RTRQNI |
| **1** | NTDRVW |
| **1** | DQPGTINA |
| **1** | DQKRRL |
| **1** | SVFGTDVL |
| **1** | GTDVLGGGVA |
| **1** | HRVNGQAA |
| **1** | GGCIPDGPY |
| **1** | DQKRRLA |
| **1** | DPTVVLPF |
| **1** | QFDQPGTI |
| **1** | QFDQPGTI |
| **1** | SGNWGDMF |
| **1** | DWDNPVVA |
| **1** | SGNWGDMF |
| **1** | VDQSPNDPA |
| **1** | DNPVVASVF |
| **1** | SADQKRRL |
| **1** | NTDRVWW |
| **1** | GDGWRQCTA |
| **1** | RSIDPTVVL |
| **1** | NFRTRQNI |
| **1** | DGRRVSDTF |
| **1** | LVDQSPNDPA |
| **1** | VDQSPNDPAF |
| **1** | TTIHRVNGQA |
| **1** | VHANTDRVW |
| **1** | RTRQNIESL |
| **1** | FDGRRVSDTF |
| **1** | RQRASGNPSQY |
| **1** | DGRRVSDTFTL |
| **1** | RTPTTRRGRTA |
| **1** | RQPTPHCVTRGF |
| **1** | RTPTTRRGRTAR |
| **1** | SARTPTTRRGRTA |
| **1** | DGTHQGRPVSPTDSL |
| **1** | QARQPTPHCVTRGF |
| **1** | QARQPTPHCVTRGF |
| **1** | DGTHQGRPVSPTDSLSA |
| **1** | RQPTPHCVTRGFNSGA |
| **1** | SGNPSQYDGTHQGRPVSPTDSL |
| **2** | GDDHPI |
| **2** | FGKGEGA |
| **2** | GGCMADL |
| **2** | PGPSVVL |
| **2** | VVKEQA |
| **2** | EECLDA |
| **2** | KGSVKY |
| **2** | GGCMADL |
| **2** | IDPVQI |
| **2** | GIPEGVI |
| **2** | GAPRSKA |
| **2** | RTGELL |
| **2** | DGSDTEA |
| **2** | PRSKAF |
| **2** | SSGYNNA |
| **2** | ETAERL |
| **2** | AGDDHPI |
| **2** | GDDHPIA |
| **2** | AETHRL |
| **2** | PDVLVSI |
| **2** | SDIKDPA |
| **2** | EVSLETA |
| **2** | AKGSVKY |
| **2** | RKKGQF |
| **2** | DGSDTEAA |
| **2** | RKPPGCL |
| **2** | GVNNRDL |
| **2** | PPPPSQPA |
| **2** | ETHRLGL |
| **2** | VGDIPDVL |
| **2** | GEARTGEL |
| **2** | RKPPGCLA |
| **2** | RTFEVSL |
| **2** | PVSRSTPI |
| **2** | RVTSSRSA |
| **2** | EAVVKEQA |
| **2** | SRHYNEA |
| **2** | ESGIEECL |
| **2** | IRKPPGCL |
| **2** | SVLTDGPGF |
| **2** | RVRDMGY |
| **2** | IGVNNRDL |
| **2** | RVRDMGY |
| **2** | ERKADEVA |
| **2** | ARVTSSRSA |
| **2** | LPVSRSTPI |
| **2** | GKGEGAKESL |
| **2** | PPPPSQPASL |
| **2** | KESLGEMTV |
| **2** | KESLGEMTV |
| **2** | TDGPGFGGCMA |
| **2** | TDGPGFGGCMA |
| **2** | KRRSPSKGL |
| **2** | TFPPPPSQPA |
| **2** | RVRDMGYSA |
| **2** | VEVHDEPEL |
| **2** | RVRDMGYSA |
| **2** | RVTSSRSAPL |
| **2** | WRVRDMGY |
| **2** | WRVRDMGY |
| **2** | TMDADGSDTEA |
| **2** | DNSKTDGHSY |
| **2** | TMDADGSDTEA |
| **2** | VEVHDEPELA |
| **2** | KRRSPSKGLI |
| **2** | IVEVHDEPEL |
| **2** | GVNNRDLRTF |
| **2** | ADNSKTDGHSY |
| **2** | KGSVKYGPSTTA |
| **2** | PGPSVVLRKEF |
| **2** | PVSRSTPIPPGMA |
| **2** | PVSRSTPIPPGMA |
| **2** | GEIKRRSPSKGL |
| **2** | RKKGQFPGPSVVL |
| **2** | VVKEQARKKGQF |
| **2** | DNSKTDGHSYSSGY |
| **3** | EGDAPVA |
| **3** | DHTGAY |
| **3** | SGRSDF |
| **3** | FTERGA |
| **3** | EVTEVL |
| **3** | SAGRGHL |
| **3** | MADHTGA |
| **3** | PGQAMSL |
| **3** | PSGQIGF |
| **3** | MADHTGA |
| **3** | PGQAMSL |
| **3** | TDSSDPI |
| **3** | ACRRVF |
| **3** | PVDVLVI |
| **3** | SPAMHRA |
| **3** | SPAMHRA |
| **3** | GSLRPGGVA |
| **3** | PSRTPPPA |
| **3** | YSGRSDF |
| **3** | MGLCGKDL |
| **3** | RPSHPPF |
| **3** | VIGGGDGGVL |
| **3** | ITDSSDPI |
| **3** | HPAPVDVL |
| **3** | MGLCGKDL |
| **3** | EVTEVLY |
| **3** | RRAVEGGGA |
| **3** | KHPSVRRA |
| **3** | CCQGECMW |
| **3** | TDSSDPIGPA |
| **3** | VPTYPSGQI |
| **3** | VVEVSKRF |
| **3** | CCQGECMW |
| **3** | QVTTRDEF |
| **3** | QVTTRDEF |
| **3** | RPSHPPFY |
| **3** | MSLEVTEVL |
| **3** | SGRSDFQDL |
| **3** | AVVEVSKRF |
| **3** | MSLEVTEVL |
| **3** | DDPRVEVVI |
| **3** | CRRVFPTVA |
| **3** | QVTTRDEFA |
| **3** | QVTTRDEFA |
| **3** | CCQGECMWL |
| **3** | VVEVSKRFL |
| **3** | CCQGECMWL |
| **3** | VVAPSRTPPPA |
| **3** | DDPRVEVVIA |
| **3** | PSRTPPPAVQA |
| **3** | GGGDGGVLREVL |
| **3** | GGPLRPSHPPF |
| **3** | KHPSVRRAVL |
| **3** | GFDDPRVEVVI |
| **3** | DGVIQVTTRDEF |
| **3** | REVLKHPSVRRA |
| **3** | RPGGVACCQGECMW |
| **3** | RPGGVACCQGECMW |
| **4** | RGKEVA |
| **4** | TTESGGI |
| **4** | VRSSAGL |
| **4** | SSEGIKA |
| **4** | WVRSSA |
| **4** | AGKDVDL |
| **4** | SVDNSQA |
| **4** | SMPVQY |
| **4** | LKRTVL |
| **4** | SMPVQY |
| **4** | GKRRRA |
| **4** | RDSLRL |
| **4** | NKTGTKL |
| **4** | EVLDESA |
| **4** | SNLGDGSI |
| **4** | RGKEVAI |
| **4** | TTESGGII |
| **4** | GCKDKDL |
| **4** | HMREHL |
| **4** | HMREHL |
| **4** | PGTGTLSVL |
| **4** | GDGSIGMVI |
| **4** | GCKDKDLA |
| **4** | GDGSIGMVI |
| **4** | TGEDGFEI |
| **4** | PLRGKEVA |
| **4** | NKTGTKLI |
| **4** | SVDNSQAVA |
| **4** | AHMREHL |
| **4** | AHMREHL |
| **4** | RGKDAVPW |
| **4** | PPRGHEKI |
| **4** | GYSMPVQY |
| **4** | DETTSPVEA |
| **4** | TIGKRRRA |
| **4** | GYSMPVQY |
| **4** | EISVDNSQA |
| **4** | NAGCKDKDL |
| **4** | SVLTTESGGI |
| **4** | TRRRMTF |
| **4** | HMREHLSA |
| **4** | TRRRMTF |
| **4** | GKDVDLEVL |
| **4** | HMREHLSA |
| **4** | GDTVIMKQL |
| **4** | KRTVLHDF |
| **4** | DETTSPVEAA |
| **4** | GDTVIMKQL |
| **4** | PCHVTRCGY |
| **4** | PPRGHEKIL |
| **4** | DVSHMGQVRL |
| **4** | DVSHMGQVRL |
| **4** | GKRRRAEGGGF |
| **4** | SMPVQYSSEGI |
| **4** | GIPCHVTRCGY |
| **4** | GEEVGEVTSGGW |
| **4** | SMPVQYSSEGI |
| **4** | HESHGGKMVPF |
| **4** | HESHGGKMVPF |
| **4** | DGITRRRMTF |
| **4** | STPRRHMSSEA |
| **4** | KKPFNKTGTKL |
| **4** | DGITRRRMTF |
| **4** | STPRRHMSSEA |
| **4** | FDVSHMGQVRL |
| **4** | HESHGGKMVPFA |
| **4** | FDVSHMGQVRL |
| **4** | HESHGGKMVPFA |
| **4** | QVVKSPVVKTSY |
| **4** | QVVKSPVVKTSY |
| **4** | GNDIDETTSPVEA |
| **4** | STPRRHMSSEAL |
| **4** | STPRRHMSSEAL |
| **4** | TASTPRRHMSSEA |
| **4** | TASTPRRHMSSEA |
| **4** | IQVVKSPVVKTSY |
| **4** | QVVKSPVVKTSYF |
| **4** | QVVKSPVVKTSYF |
| **4** | VVEKGAPPRGHEKI |
| **4** | NEAGEEVGEVTSGGW |
| **4** | GEEVGEVTSGGWGPTA |
| **4** | TRRRMTFVVEKGA |
| **4** | TRRRMTFVVEKGA |
| **4** | HDFHESHGGKMVPF |
| **4** | HDFHESHGGKMVPF |
| **4** | PCHVTRCGYTGEDGF |
| **4** | DVSHMGQVRLRGKDA |
| **4** | DVSHMGQVRLRGKDA |
| **5** | QVDEPA |
| **5** | SREAQA |
| **5** | VTKAGW |
| **5** | PEGVRL |
| **5** | SRVVPI |
| **5** | VKSRLA |
| **5** | LDEVEA |
| **5** | TRGDNL |
| **5** | MNDLDA |
| **5** | PLKRPA |
| **5** | CVRPPI |
| **5** | REGLPL |
| **5** | PGWTDL |
| **5** | SPDLCSA |
| **5** | HVPLDL |
| **5** | MNDLDA |
| **5** | VRVAGEA |
| **5** | TGPTTIL |
| **5** | LSGGVTVA |
| **5** | DTTTRL |
| **5** | VGDTSSAA |
| **5** | SDPDVLA |
| **5** | VRSVGSL |
| **5** | GDTFPW |
| **5** | PSDEYL |
| **5** | RSVYEA |
| **5** | VGVGDATL |
| **5** | PGVKAVGL |
| **5** | GVPEVQL |
| **5** | KQKLDL |
| **5** | TPVKGML |
| **5** | ERAEPF |
| **5** | EKMNGVA |
| **5** | TRVRAF |
| **5** | PRHRAL |
| **5** | QVDEPAL |
| **5** | QKDTLGL |
| **5** | PWSVRL |
| **5** | VTSDGPAL |
| **5** | TPVKGML |
| **5** | EKMNGVA |
| **5** | LRTHGW |
| **5** | PAVKSRL |
| **5** | IQVDEPA |
| **5** | QVDEPAL |
| **5** | VARVRTA |
| **5** | QKDTLGL |
| **5** | ADTTTRL |
| **5** | LDMSKW |
| **5** | DDLGDTF |
| **5** | HWRGDL |
| **5** | SRVVPIL |
| **5** | SGGVTVASL |
| **5** | LDMSKW |
| **5** | CVRPPII |
| **5** | PEGVRLGA |
| **5** | GASRVVPI |
| **5** | GTMSIGSF |
| **5** | GSFPQTPA |
| **5** | HVLVPEI |
| **5** | GTMSIGSF |
| **5** | AEKMNGVA |
| **5** | TVECVRL |
| **5** | HSEVVPSA |
| **5** | AEKMNGVA |
| **5** | EAPRHRA |
| **5** | GVGLVGVGDA |
| **5** | TRGDNLGL |
| **5** | SFPRKDL |
| **5** | TMTTTVGY |
| **5** | DTTTRLGL |
| **5** | DVLVHGEL |
| **5** | VPEIGGEGGA |
| **5** | TMTTTVGY |
| **5** | ATVECVRL |
| **5** | GEAVGDTSSA |
| **5** | HSEVVPSAA |
| **5** | VVVREEVA |
| **5** | EVATRVRA |
| **5** | GKLPSDEY |
| **5** | SVRLPGVKA |
| **5** | VCVQPSCSL |
| **5** | DFTRGDNL |
| **5** | QNTNFHVL |
| **5** | GWVRSVGSL |
| **5** | AVVVREEVA |
| **5** | QNTNFHVL |
| **5** | GVVDGRSVW |
| **5** | GDATPVKGML |
| **5** | SDFSDPDVL |
| **5** | RVRTAQGTI |
| **5** | GDATPVKGML |
| **5** | VCVQPSCSLA |
| **5** | PSTQVVTHF |
| **5** | VVPDCGQKTA |
| **5** | PCMGGRREL |
| **5** | ERTDMVEF |
| **5** | PCMGGRREL |
| **5** | SVNGGLGTMSI |
| **5** | ERTDMVEF |
| **5** | GVVDGRSVWA |
| **5** | EVRRSKQF |
| **5** | SVNGGLGTMSI |
| **5** | DIHSEVVPSA |
| **5** | PRKDLSREA |
| **5** | APSTQVVTHF |
| **5** | REAGVPEVQL |
| **5** | GAGVVDGRSVW |
| **5** | VAVCVQPSCSL |
| **5** | TVENTRSGGEA |
| **5** | VVVREEVADL |
| **5** | EKMNGVAVTKA |
| **5** | EKMNGVAVTKA |
| **5** | ERTDMVEFF |
| **5** | ERTDMVEFF |
| **5** | WVVPDCGQKTA |
| **5** | HEPTLVTSDGPA |
| **5** | PTSATMTTTVGY |
| **5** | PTSATMTTTVGY |
| **5** | TVENTRSGGEAF |
| **5** | DMSKWQNTNF |
| **5** | PSTQVVTHFCY |
| **5** | DMSKWQNTNF |
| **5** | RTHGWPEGVRL |
| **5** | TPVKGMLTGPTTI |
| **5** | PCMGGRRELKTA |
| **5** | GVPEVQLHEPTL |
| **5** | TPVKGMLTGPTTI |
| **5** | PCMGGRRELKTA |
| **5** | VRSVGSLCVRPPI |
| **5** | TVECVRLQKDTL |
| **5** | EPFEVRRSKQF |
| **5** | VVPDCGQKTASPDL |
| **5** | DVLTVENTRSGGEA |
| **5** | SRRQVPMTVREW |
| **5** | SRRQVPMTVREW |
| **5** | VHGELERTDMVEF |
| **5** | EVRRSKQFSVNGGL |
| **5** | VHGELERTDMVEF |
| **5** | SRRQVPMTVREWKVA |
| **5** | SRRQVPMTVREWKVA |
| **5** | TMTTTVGYPCMGGRREL |
| **5** | TMTTTVGYPCMGGRREL |
| **5** | TMTTTVGYPCMGGRREL |
| **5** | HGDLSRRQVPMTVREW |
| **5** | HGDLSRRQVPMTVREW |
| **6** | GHGSDAL |
| **6** | QFDGTL |
| **6** | CMANPI |
| **6** | TYGRTA |
| **6** | QFDGTL |
| **6** | EVTARL |
| **6** | DGRVADA |
| **6** | AVGPPRL |
| **6** | KTHRGGA |
| **6** | RRNTDA |
| **6** | SPAPPPPA |
| **6** | NPIPTKA |
| **6** | QVVPYY |
| **6** | QRMVKL |
| **6** | GATGVVSVA |
| **6** | SEQYEL |
| **6** | QVVPYY |
| **6** | QRMVKL |
| **6** | QRMVKL |
| **6** | RLDNVVA |
| **6** | QRMVKL |
| **6** | QHFKHI |
| **6** | GSNSTEEA |
| **6** | QHFKHI |
| **6** | CMSAEVTA |
| **6** | VKEASGDL |
| **6** | NWSEQY |
| **6** | CMSAEVTA |
| **6** | MEATRRA |
| **6** | NIPGRSSI |
| **6** | VGPPRLPL |
| **6** | MEATRRA |
| **6** | TRRAKKL |
| **6** | PPPPATETA |
| **6** | NKPPQEGI |
| **6** | SHFVGPEL |
| **6** | TPEVEAKI |
| **6** | HMYSGDDA |
| **6** | HMYSGDDA |
| **6** | QRMVKLY |
| **6** | GAGSNSTEEA |
| **6** | TDATPEVEA |
| **6** | QRMVKLY |
| **6** | QRMVKLY |
| **6** | DNVVAVKEA |
| **6** | QRMVKLY |
| **6** | DGTLQVVPY |
| **6** | RRNTDADF |
| **6** | TGVVSVASHF |
| **6** | PGRSSICMSA |
| **6** | CEPDLPVML |
| **6** | PGRSSICMSA |
| **6** | CEPDLPVML |
| **6** | YNKPPQEGI |
| **6** | NKPPQEGIY |
| **6** | TDIRRNTDA |
| **6** | TPFKTHRGGA |
| **6** | VVGGTTGESPTL |
| **6** | GSNSTEEAMEA |
| **6** | GSNSTEEAMEA |
| **6** | LVVGGTTGESPTL |
| **6** | KTHRGGAEVDY |
| **6** | VGPELQRMVKL |
| **6** | VGPELQRMVKL |
| **6** | VVGGTTGESPTLNW |
| **6** | NVVRQTVGDKVKL |
| **6** | NVVRQTVGDKVKLI |
| **6** | FNVVRQTVGDKVKL |
| **7** | DPDEVI |
| **7** | TVPDTY |
| **7** | DETGYL |
| **7** | LRTTDI |
| **7** | RTTDIL |
| **7** | FRVDDA |
| **7** | RRRAVA |
| **7** | EPGGVTVA |
| **7** | LRSVVVA |
| **7** | ETGSRSI |
| **7** | DMSRHL |
| **7** | ERVLGW |
| **7** | GVVTVATI |
| **7** | DMSRHL |
| **7** | AEPGGVTVA |
| **7** | ITVPDTY |
| **7** | VGTPGLGVI |
| **7** | RRRSQI |
| **7** | PDTTHTF |
| **7** | DMSRHLA |
| **7** | DMSRHLA |
| **7** | RNRGVTF |
| **7** | VTLVGTPGL |
| **7** | TVPDTYY |
| **7** | ETGSRSIL |
| **7** | QPVGERGF |
| **7** | DHVVSNHA |
| **7** | KMPVNEPA |
| **7** | GGGGDPLVTL |
| **7** | QPVGERGF |
| **7** | KMPVNEPA |
| **7** | EDLRRRA |
| **7** | VDFDETGY |
| **7** | RNRGVTFI |
| **7** | THVVTQGDI |
| **7** | PARRRSQI |
| **7** | KDEAGVVTVA |
| **7** | LQPVGERGF |
| **7** | WPDTTHTF |
| **7** | THGDGVHDVA |
| **7** | GVSVSPPQEL |
| **7** | KMPVNEPAPA |
| **7** | KMPVNEPAPA |
| **7** | PDTTHTFVSA |
| **7** | RGLETGSRSI |
| **7** | QPVGERGFGF |
| **7** | ATHGDGVHDVA |
| **7** | LTHVVTQGDI |
| **7** | AGVSVSPPQEL |
| **7** | EPGGVTVAEDL |
| **7** | QPVGERGFGF |
| **7** | DGDMEGVVSW |
| **7** | DGDMEGVVSW |
| **7** | GVIDHVVSNHA |
| **7** | THGDGVHDVAF |
| **7** | THVVTQGDITL |
| **7** | KPGVRTTPVTY |
| **7** | SVDDKMHTEY |
| **7** | SVDDKMHTEY |
| **7** | VSTLRNRGVTF |
| **7** | RRRSQIQHVA |
| **7** | TPDNADMSRHL |
| **7** | DGDMEGVVSWY |
| **7** | TPDNADMSRHL |
| **7** | DGDMEGVVSWY |
| **7** | AKPGVRTTPVTY |
| **7** | RSVVVADPDEVI |
| **7** | KPGVRTTPVTYSA |
| **7** | SVDDKMHTEYSA |
| **7** | SVDDKMHTEYSA |
| **7** | WSVDDKMHTEY |
| **7** | WSVDDKMHTEY |
| **7** | GVSVSPPQELKDEA |
| **7** | DPDEVIKMPVNEPA |
| **7** | DPDEVIKMPVNEPA |
| **7** | RDSRPVGEGGSGGGRRA |
| **7** | DHVVSNHADGDMEGVVSW |
| **7** | DHVVSNHADGDMEGVVSW |
| **7** | PGYRDSRPVGEGGSGGGRRA |
| **7** | RDSRPVGEGGSGGGRRAGGGGDPL |
| **9** | RPGVLI |
| **9** | KEVIPA |
| **9** | TGRKVI |
| **9** | NPSGRF |
| **9** | FKKTSA |
| **9** | LDDDTI |
| **9** | EPISVF |
| **9** | GEITTTA |
| **9** | KKTSAY |
| **9** | DTYGGW |
| **9** | VAKSMVA |
| **9** | LVQVSY |
| **9** | ERDGGAL |
| **9** | ETVKKL |
| **9** | KTCRVL |
| **9** | VAKSMVA |
| **9** | CDQVSDA |
| **9** | DDDTIY |
| **9** | IVKKNF |
| **9** | DAEEKGL |
| **9** | VQVSYGI |
| **9** | GDQGIMF |
| **9** | DLRPGVL |
| **9** | GGPEGDAGL |
| **9** | HAQSPEI |
| **9** | GDQGIMF |
| **9** | TGRKVII |
| **9** | MPLTHSL |
| **9** | TNESEDL |
| **9** | VIGGPEGDA |
| **9** | MPLTHSL |
| **9** | SVFVDSY |
| **9** | GLTGRKVI |
| **9** | EEVIREA |
| **9** | GREGFTW |
| **9** | EEKGLDY |
| **9** | VKKNFDL |
| **9** | QDPHSKVA |
| **9** | ATNESEDL |
| **9** | QDPHSKVA |
| **9** | THSLSTKL |
| **9** | NPSGRFVI |
| **9** | TTTAKVNY |
| **9** | TVVRKEGI |
| **9** | GHFGREGF |
| **9** | STKLGHRL |
| **9** | HLNPSGRF |
| **9** | CDQVSDAVL |
| **9** | AQDPHSKVA |
| **9** | ETVKKLEL |
| **9** | PPPPPSVTMSA |
| **9** | KPKRVHTI |
| **9** | PPPPPSVTMSA |
| **9** | KVNYEEVI |
| **9** | DYKTCRVL |
| **9** | SKTGMVMCF |
| **9** | TWETVKKL |
| **9** | SKTGMVMCF |
| **9** | GTGTKSDDEL |
| **9** | SKTGMVMCF |
| **9** | KTCRVLVEL |
| **9** | MTHVIKEVI |
| **9** | RSDLMTHVI |
| **9** | PPPPPSVTMSA |
| **9** | MTHVIKEVI |
| **9** | RSDLMTHVI |
| **9** | LKPKRVHTI |
| **9** | PPPPPSVTMSA |
| **9** | GTGTKSDDELL |
| **9** | TNESEDLMPL |
| **9** | TNESEDLMPL |
| **9** | GVHEGRTSDDL |
| **9** | KPKRVHTIVI |
| **9** | AGVHEGRTSDDL |
| **9** | SGKDPTKVDRSA |
| **9** | QDPHSKVACETA |
| **9** | QDPHSKVACETA |
| **9** | TVVRKEGICPW |
| **9** | SKTGMVMCFGEI |
| **9** | GVHEGRTSDDLGA |
| **9** | SKTGMVMCFGEI |
| **9** | SGKDPTKVDRSAA |
| **9** | SKTGMVMCFGEI |
| **9** | GHRLTVVRKEGI |
| **9** | CETASKTGMVMCF |
| **9** | FSGKDPTKVDRSA |
| **9** | TSESVNEGHPDKL |
| **9** | CETASKTGMVMCF |
| **9** | CETASKTGMVMCF |
| **9** | VDSYGTGTKSDDEL |
| **9** | VRPDGKTQVTVEY |
| **9** | PPPPPSVTMSAMKNF |
| **9** | STQHDDEVSNEKI |
| **9** | PPPPPSVTMSAMKNF |
| **9** | PPPPPSVTMSAMKNF |
| **9** | FTSESVNEGHPDKL |
| **9** | PPPPPSVTMSAMKNF |
| **9** | PPPPPSVTMSAMKNF |
| **9** | PPPPPSVTMSAMKNF |
| **9** | VISTQHDDEVSNEKI |
| **9** | CPWVRPDGKTQVTVEY |
| **9** | STQHDDEVSNEKIRSDL |
| **9** | VRPDGKTQVTVEYERDGGA |
| **9** | TSESVNEGHPDKLCDQVSDA |
| **10** | RHGGGGL |
| **10** | KSSCAW |
| **10** | WKSSCA |
| **10** | PSPAVTL |
| **10** | KDTDAF |
| **10** | VLRRVA |
| **10** | EVYPSGA |
| **10** | RHGGGGLA |
| **10** | RITQSVA |
| **10** | RDGGEQF |
| **10** | VARHGGGGL |
| **10** | RFKDTDA |
| **10** | PRFGVDVA |
| **10** | GVQPLQNL |
| **10** | SSQPRVVL |
| **10** | GGTPSSPPPL |
| **10** | LRDGGEQF |
| **10** | MASSQPRVVL |
| **10** | AGGTPSSPPPL |
| **10** | MASSQPRVVL |
| **10** | MASSQPRVVL |
| **10** | MASSQPRVVL |
| **10** | MASSQPRVVL |
| **10** | RDGGEQFDPA |
| **10** | MASSQPRVVL |
| **10** | VEVVNAGVQPL |
| **10** | VRELVEVVNA |
| **10** | GGTPSSPPPLRI |
| **10** | SSQPRVVLHSY |
| **11** | EDTADL |
| **11** | GVETTY |
| **11** | GVRLRA |
| **11** | QMTGKGA |
| **11** | SASDEPA |
| **11** | DPSRGF |
| **11** | GQTLRI |
| **11** | QMTGKGA |
| **11** | QMTGKGA |
| **11** | CVTGSGVA |
| **11** | IGGHSDL |
| **11** | MTDAVY |
| **11** | VESPASL |
| **11** | GGGLGQTL |
| **11** | QMTGKGA |
| **11** | PAPHSSL |
| **11** | SMGFSW |
| **11** | MTDAVY |
| **11** | RGLRTL |
| **11** | KGGHRPA |
| **11** | GPNTKVI |
| **11** | SMGFSW |
| **11** | PFTGMW |
| **11** | QMTGKGAA |
| **11** | PFTGMW |
| **11** | SDEPANGA |
| **11** | EVMDVPA |
| **11** | QMTGKGAA |
| **11** | HASTVTF |
| **11** | QMTGKGAA |
| **11** | CVTGSGVAA |
| **11** | ETGDHVL |
| **11** | EARPEVA |
| **11** | TTRECY |
| **11** | GVDVSINA |
| **11** | TIEGGDNA |
| **11** | EVMDVPA |
| **11** | QMTGKGAA |
| **11** | ESHPQHA |
| **11** | RVMHPGL |
| **11** | RTLGVRL |
| **11** | DLGVDVSI |
| **11** | RAVKSSVA |
| **11** | RVMHPGL |
| **11** | GGHSDLML |
| **11** | GVETTYY |
| **11** | VKSSVAEL |
| **11** | RSVKYW |
| **11** | ATTRECY |
| **11** | GGHSDLML |
| **11** | RDNTKVL |
| **11** | EWSTDPL |
| **11** | EVMDVPAL |
| **11** | DEMLDGMA |
| **11** | LETGDHVL |
| **11** | EVMDVPAL |
| **11** | YGPNTKVI |
| **11** | DEMLDGMA |
| **11** | DEMLDGMA |
| **11** | ESHPQHAL |
| **11** | STVTFPTVA |
| **11** | NSARSVKY |
| **11** | VYDPSRGF |
| **11** | DNTWGPMF |
| **11** | DNTWGPMF |
| **11** | FRDNTKVL |
| **11** | RDNTKVLF |
| **11** | SFEVMDVPA |
| **11** | TTRECYRA |
| **11** | SFEVMDVPA |
| **11** | GCPPGSDDCY |
| **11** | GRHGNPTTW |
| **11** | GRFGVETTY |
| **11** | KGGHRPAEW |
| **11** | GRSVVNPPVY |
| **11** | GRHGNPTTWA |
| **11** | GCPPGSDDCYL |
| **11** | DPSRGFCDKF |
| **11** | ETGDHVLMTDA |
| **11** | YGRHGNPTTW |
| **11** | ETGDHVLMTDA |
| **11** | PHSSLQMTGKGA |
| **11** | PHSSLQMTGKGA |
| **11** | EGGDNACVTGSGVA |
| **11** | ELGCPPGSDDCY |
| **11** | GRSVVNPPVYHA |
| **11** | RPEVARVMHPGL |
| **11** | RPEVARVMHPGL |
| **11** | GPNTKVIKGGHRPA |
| **11** | RVMHPGLESHPQHA |
| **11** | STDPLGRSVVNPPVY |
| **11** | RVMHPGLESHPQHA |
| **12** | YGTVTL |
| **12** | RAPPTI |
| **12** | GEGEPPA |
| **12** | TGGVRPA |
| **12** | LVSDDI |
| **12** | VKTKDA |
| **12** | CDPGCTA |
| **12** | PTGQTY |
| **12** | RPEPTA |
| **12** | KGRTTI |
| **12** | PMASTTA |
| **12** | MRPLTA |
| **12** | PMASTTA |
| **12** | SNRVLL |
| **12** | MRPLTA |
| **12** | EVTDEL |
| **12** | QGDTRF |
| **12** | TSPGEAF |
| **12** | SKAKSKA |
| **12** | TGKSSGLA |
| **12** | QGDTRF |
| **12** | LKRHVA |
| **12** | TYMKNA |
| **12** | RRMSAI |
| **12** | VEGDASSA |
| **12** | ARPEPTA |
| **12** | TYMKNA |
| **12** | RNKGRL |
| **12** | DDHRMA |
| **12** | PGDRQTA |
| **12** | GTAMRPL |
| **12** | AKGRTTI |
| **12** | CKGKTVI |
| **12** | RRMSAI |
| **12** | TPPPGGLI |
| **12** | DDHRMA |
| **12** | IGGPGGRF |
| **12** | GTAMRPL |
| **12** | LTGGVRPA |
| **12** | LVKTKDA |
| **12** | QGDTRFA |
| **12** | RPEPTAL |
| **12** | CGKVGVDI |
| **12** | QGDTRFA |
| **12** | GGPGGRFF |
| **12** | KLMKQF |
| **12** | TKEQSPL |
| **12** | SKSYGTY |
| **12** | TSKPYVL |
| **12** | KLMKQF |
| **12** | VSDDITY |
| **12** | TGGVRPASA |
| **12** | KRHVAPI |
| **12** | SVLEKMGA |
| **12** | TRRLPMA |
| **12** | SVLEKMGA |
| **12** | RNVYNW |
| **12** | TRRLPMA |
| **12** | TTELRKL |
| **12** | ACGKVGVDI |
| **12** | GWRRMSA |
| **12** | SSTQSTPPA |
| **12** | VIPTGQTY |
| **12** | GWRRMSA |
| **12** | GEGEPPAGW |
| **12** | DDHRMAMA |
| **12** | ASSTQSTPPA |
| **12** | SLCKGKTVI |
| **12** | VKTKDAVKA |
| **12** | DDHRMAMA |
| **12** | DDHRMAMA |
| **12** | HREPQCPL |
| **12** | PPTIDHVVL |
| **12** | RPGVAVETY |
| **12** | TVSWTPNSI |
| **12** | EKMGATVSW |
| **12** | TVSRSRDSI |
| **12** | EKMGATVSW |
| **12** | CDPGCTAKTF |
| **12** | KGVDEDCGDI |
| **12** | GTVTLPGSKSL |
| **12** | TKEQSPLVY |
| **12** | PGGTTRVSGQI |
| **12** | VPVFTGKSSGL |
| **12** | RMSAGEGEPPA |
| **12** | RNKGRLSVCA |
| **12** | VEPTGHGDGTF |
| **12** | RMSAGEGEPPA |
| **12** | SSTQSTPPANGA |
| **12** | FHREPQCPL |
| **12** | CKGKTVIRNL |
| **12** | HREPQCPLSA |
| **12** | PGSKSLSNRVL |
| **12** | TVSRSRDSIF |
| **12** | FKGVDEDCGDI |
| **12** | VVEEGRDSCVI |
| **12** | KGRTTIRNVY |
| **12** | PTGQTYTSPGEA |
| **12** | SKVTIEVTDEL |
| **12** | VETYDDHRMA |
| **12** | RVKETERMKA |
| **12** | VETYDDHRMA |
| **12** | VEPTGHGDGTFF |
| **12** | RVKETERMKA |
| **12** | EVTDELTSKPY |
| **12** | PAVEPTGHGDGTF |
| **12** | DRTITKEQSPL |
| **12** | DHVVLPGDRQTA |
| **12** | DGTPRMRERPI |
| **12** | KGVDEDCGDIPDA |
| **12** | GAVVEEGRDSCVI |
| **12** | TGGSVTVVGCGSDSL |
| **12** | DGTPRMRERPI |
| **12** | RVKETERMKAI |
| **12** | RVKETERMKAI |
| **12** | CGKVGVDICDPGCTA |
| **12** | DGTPRMRERPII |
| **12** | LDGTPRMRERPI |
| **12** | ITGGSVTVVGCGSDSL |
| **12** | DGTPRMRERPII |
| **12** | LDGTPRMRERPI |
| **12** | PGDRQTARNKGRL |
| **12** | GVTVETNNEMSRF |
| **12** | GVTVETNNEMSRF |
| **12** | TPNSITVSRSRDSI |
| **12** | PGGTTRVSGQISSQY |
| **12** | NWRVKETERMKA |
| **12** | NWRVKETERMKA |
| **12** | GVTVETNNEMSRFVI |
| **12** | GVTVETNNEMSRFVI |
| **12** | VVEEGRDSCVITPPPGGL |
| **12** | MKQFGVTVETNNEMSRF |
| **12** | MKQFGVTVETNNEMSRF |
| **12** | TGGSVTVVGCGSDSLQGDTRF |
| **12** | MKQFGVTVETNNEMSRF |
| **12** | HVTCSDTGCPPVVVNTTGEGL |
| **12** | GAHVTCSDTGCPPVVVNTTGEGL |
| **12** | GVPVTSSTRDPSTVTVQGQGGEW |
| **12** | GVPVTSSTRDPSTVTVQGQGGEWPA |
| **12** | SGLGVPVTSSTRDPSTVTVQGQGGEW |
| **12** | HVTCSDTGCPPVVVNTTGEGLPGGTTRVSGQI |
| **13** | LGRTRL |
| **13** | VRECVL |
| **13** | GSLRGVPA |
| **13** | GRTRLPA |
| **13** | RTPIGSF |
| **13** | STLHDVY |
| **13** | RGVPAVEL |
| **13** | DAVRECVL |
| **13** | VRECVLGNVL |
| **13** | TVCTTVNKVCA |
| **13** | ATVCTTVNKVCA |
| **13** | TVCTTVNKVCASGL |
| **14** | RSPDAI |
| **14** | RMTDGA |
| **14** | KHVIGL |
| **14** | LDSSRL |
| **14** | DFTHGGA |
| **14** | TNWGVGA |
| **14** | SMANVGGA |
| **14** | HARVVGA |
| **14** | VARSPDA |
| **14** | NVGGARF |
| **14** | SMANVGGA |
| **14** | VDELCF |
| **14** | GSLVDEL |
| **14** | VSNPVDI |
| **14** | RGAKVTI |
| **14** | EEDVFL |
| **14** | KGYTNW |
| **14** | RVPLSEA |
| **14** | VGCGSVGMA |
| **14** | VGCGSVGMA |
| **14** | GIEEDVF |
| **14** | EHTKLHA |
| **14** | RMTDGAW |
| **14** | PKSVHASI |
| **14** | PNYDGSEA |
| **14** | RMTDGAW |
| **14** | IVSNPVDI |
| **14** | VSNPVDIL |
| **14** | GEHGDSSVA |
| **14** | EVARSVEA |
| **14** | GVAPKSVHA |
| **14** | DIRMTDGA |
| **14** | DGSEASDVI |
| **14** | DIRMTDGA |
| **14** | KKVEGEVL |
| **14** | DSSRLRVA |
| **14** | SEAEHTKL |
| **14** | GGGNDSGGSSGGI |
| **14** | GRGGVERVL |
| **14** | GRVFGSGTY |
| **14** | VGCGSVGMACA |
| **14** | VGCGSVGMACA |
| **14** | LGEHGDSSVA |
| **14** | GGGNDSGGSSGGIA |
| **14** | GGGNDSGGSSGGI |
| **14** | GEHGDSSVAVA |
| **14** | GGGNDSGGSSGGIA |
| **14** | RQRPGESRL |
| **14** | VLGRGGVERVL |
| **14** | KKVEGEVLDF |
| **14** | RQRPGESRLA |
| **14** | PSVGGGGDRGVGSL |
| **14** | KVTIVGCGSVGMA |
| **14** | GARQRPGESRL |
| **14** | KVTIVGCGSVGMA |
| **14** | DVDAKKVEGEVL |
| **14** | RDEKRVVPVSVA |
| **14** | GRGGVERVLRVPL |
| **14** | RDEKRVVPVSVAA |
| **14** | CFPSVGGGGDRGVGSL |
| **14** | LRDEKRVVPVSVA |
| **14** | PSVGGGGDRGVGSLRGA |
| **15** | VSADDF |
| **15** | GGDGVIF |
| **15** | GKYQGL |
| **15** | VPTTLPA |
| **15** | PGGDLQI |
| **15** | MTGPAEL |
| **15** | QGLGNDF |
| **15** | GMDPTTL |
| **15** | MTGPAEL |
| **15** | FTGSVMV |
| **15** | GMDPTTL |
| **15** | QGLGNDF |
| **15** | GVIVPTVA |
| **15** | FTGSVMV |
| **15** | RVNTEF |
| **15** | CTASMRI |
| **15** | VTVDAPDA |
| **15** | VRMVVW |
| **15** | CTASMRI |
| **15** | CDRHTGI |
| **15** | GPQFENL |
| **15** | VRMVVW |
| **15** | ENLTSVF |
| **15** | VPTVAPGGGA |
| **15** | ERLGPQF |
| **15** | DKDLERL |
| **15** | LCDRHTGI |
| **15** | NEADGRVY |
| **15** | PARVNTEF |
| **15** | GMDPTTLVL |
| **15** | GRTRACGTGA |
| **15** | DLGMDPTTL |
| **15** | GMDPTTLVL |
| **15** | ERGAGRTRA |
| **15** | DLGMDPTTL |
| **15** | VDNRDSTDL |
| **15** | PVTVDGREW |
| **15** | DGRVYMTGPA |
| **15** | PDAVRMVVW |
| **15** | DGRVYMTGPA |
| **15** | PDAVRMVVW |
| **15** | LVDNRDSTDL |
| **15** | TMTGVSMGNPHA |
| **15** | VRMVVWERGA |
| **15** | TMTGVSMGNPHA |
| **15** | VRMVVWERGA |
| **15** | TMTGVSMGNPHA |
| **15** | VDNRDSTDLTL |
| **15** | RVNTEFVTVDA |
| **15** | TGRTERTVTVGL |
| **15** | CDRHTGIGGDGVI |
| **15** | TTAPVTVDGREW |
| **15** | VRVDMGVPVTDPPA |
| **15** | VRVDMGVPVTDPPA |
| **15** | VLTGRTERTVTVGL |
| **15** | TMTGVSMGNPHAVTF |
| **15** | TMTGVSMGNPHAVTF |
| **15** | TMTGVSMGNPHAVTF |
| **15** | TGRTERTVTVGLPGGDL |
| **15** | NSDGSEPEMCGNGVRCL |
| **15** | NSDGSEPEMCGNGVRCL |
| **15** | PGGGAVRVDMGVPVTDPPA |
| **15** | PGGGAVRVDMGVPVTDPPA |
| **15** | NSDGSEPEMCGNGVRCLA |
| **15** | NSDGSEPEMCGNGVRCLA |
| **15** | INSDGSEPEMCGNGVRCL |
| **15** | INSDGSEPEMCGNGVRCL |
| **15** | VRVDMGVPVTDPPAVPTTL |
| **15** | VRVDMGVPVTDPPAVPTTL |
| **15** | PVTVDGREWTMTGVSMGNPHA |
| **15** | PVTVDGREWTMTGVSMGNPHA |
| **15** | PVTVDGREWTMTGVSMGNPHA |
| **16** | GVPMAW |
| **16** | VSSGVTL |
| **16** | AGMDNY |
| **16** | DVIVDL |
| **16** | GVPMAW |
| **16** | AGMDNY |
| **16** | PGVEVW |
| **16** | STQMGTA |
| **16** | PHVSSVA |
| **16** | PVRTRA |
| **16** | VERIDA |
| **16** | QRPPDI |
| **16** | STQMGTA |
| **16** | VGNRASL |
| **16** | RVATKF |
| **16** | GYGGCRL |
| **16** | PVTYVF |
| **16** | QRPPDI |
| **16** | RTPRSTI |
| **16** | PGPTGDAL |
| **16** | HEDLGY |
| **16** | VKMDGAL |
| **16** | LEDVGMA |
| **16** | QLVGNRA |
| **16** | VKMDGAL |
| **16** | LEDVGMA |
| **16** | QLVGNRA |
| **16** | SAPGPTGDA |
| **16** | LNPRQY |
| **16** | CGMDGPTI |
| **16** | PGVEVWL |
| **16** | CGMDGPTI |
| **16** | QQLGVGGTA |
| **16** | NGGTLVTSA |
| **16** | PSKGGMSSA |
| **16** | QQLGVGGTA |
| **16** | RTPRSTI |
| **16** | KEINGGTL |
| **16** | PSKGGMSSA |
| **16** | LQRPPDI |
| **16** | GMDNYEL |
| **16** | RGLCREL |
| **16** | PVRTRAF |
| **16** | NPRQYVA |
| **16** | GMDNYEL |
| **16** | ELVKMDGA |
| **16** | ELVKMDGA |
| **16** | WPHVSSVA |
| **16** | EASTQMGTA |
| **16** | VVPKDSVY |
| **16** | EASTQMGTA |
| **16** | PHVSSVADL |
| **16** | IPSKGGMSSA |
| **16** | RGSSMNDVA |
| **16** | IPSKGGMSSA |
| **16** | RGSSMNDVA |
| **16** | EDVGMAVQL |
| **16** | EQESKRW |
| **16** | EDVGMAVQL |
| **16** | VDLVSSGVTL |
| **16** | CRELVERI |
| **16** | MGQQQCNVI |
| **16** | STQMGTADVI |
| **16** | RNAPGVEVW |
| **16** | RSVGGSGVCVL |
| **16** | MGQQQCNVI |
| **16** | STQMGTADVI |
| **16** | RTPRSTIRVA |
| **16** | TKFPVRTRA |
| **16** | GLVVPKDSVY |
| **16** | EHGGSDGRVVA |
| **16** | MGQQQCNVIA |
| **16** | MGQQQCNVIA |
| **16** | VVPKDSVYRA |
| **16** | EHGGSDGRVVAA |
| **16** | RTPRSTIRVA |
| **16** | NIRGSSMNDVA |
| **16** | EQESKRWHA |
| **16** | VSSGVTLRENL |
| **16** | NIRGSSMNDVA |
| **16** | VFEQESKRW |
| **16** | QTDLCGMDGPTI |
| **16** | QTDLCGMDGPTI |
| **16** | QTDLCGMDGPTI |
| **16** | VAEHGGSDGRVVA |
| **16** | QTDLCGMDGPTI |
| **16** | HLMGQQQCNVI |
| **16** | VRKVRDGDVDL |
| **16** | HLMGQQQCNVI |
| **16** | PSKGGMSSATTRL |
| **16** | PSKGGMSSATTRL |
| **16** | RGSSMNDVARRL |
| **16** | RGSSMNDVARRL |
| **16** | QQLRSVGGSGVCVL |
| **16** | QQLRSVGGSGVCVL |
| **16** | VRKVRDGDVDLGF |
| **16** | RSVGGSGVCVLPVTY |
| **16** | NSVVPPRGTETGMY |
| **16** | NSVVPPRGTETGMY |
| **16** | NSVVPPRGTETGMYA |
| **16** | NSVVPPRGTETGMYA |
| **16** | QRPPDIVRKVRDGDVDL |
| **16** | QRPPDIVRKVRDGDVDL |
| **16** | CGMDGPTINSVVPPRGTETGMY |
| **16** | CGMDGPTINSVVPPRGTETGMY |
| **16** | CGMDGPTINSVVPPRGTETGMY |
| **17** | EGAGQEA |
| **17** | RGSGTLA |
| **17** | VDNGVY |
| **17** | EEDDAL |
| **17** | GYPRSL |
| **17** | SLKQRA |
| **17** | LRGSGTL |
| **17** | FGRTRA |
| **17** | SDKSSW |
| **17** | TREELA |
| **17** | GRAPHPL |
| **17** | LNHNPY |
| **17** | VDEADPL |
| **17** | DTDDWI |
| **17** | IRERSL |
| **17** | DAVDVSVA |
| **17** | LVDNGVY |
| **17** | VDNGVYL |
| **17** | RDRGMY |
| **17** | GQEAGSSVA |
| **17** | GRTRAVF |
| **17** | RDRGMY |
| **17** | LSDKSSW |
| **17** | SLRRKVA |
| **17** | ERKVRF |
| **17** | GKMEQVY |
| **17** | GQKTNAEL |
| **17** | VVDRNPY |
| **17** | ARDRGMY |
| **17** | RDRGMYA |
| **17** | GKMEQVY |
| **17** | NHNPYCGA |
| **17** | ARDRGMY |
| **17** | RDRGMYA |
| **17** | DTFQENF |
| **17** | VSERNEVA |
| **17** | RERSLGDA |
| **17** | DRYPMEL |
| **17** | DRYPMEL |
| **17** | TGTGYVCVF |
| **17** | ERKVRFL |
| **17** | MPADTDDW |
| **17** | MPADTDDW |
| **17** | TEEEMDSGA |
| **17** | RRKVAGEY |
| **17** | TTYGQKTNA |
| **17** | TEEEMDSGA |
| **17** | RWTREEL |
| **17** | SDKSSWGRA |
| **17** | YERKVRF |
| **17** | QENFTGTGY |
| **17** | VDVSVAVDEA |
| **17** | QENFTGTGY |
| **17** | VKGVVQGEPL |
| **17** | PRSLEEDDA |
| **17** | VVDRNPYDA |
| **17** | GFVVDRNPY |
| **17** | VSERNEVAVA |
| **17** | VCVFTDRPY |
| **17** | RQVRGEKRI |
| **17** | VKGVVQGEPLF |
| **17** | VAVKGVVQGEPL |
| **17** | GKMEQVYTTY |
| **17** | GDFVSERNEVA |
| **17** | GKMEQVYTTY |
| **17** | CVATEEEMDSGA |
| **17** | LRQVRGEKRI |
| **17** | RQVRGEKRIL |
| **17** | CVATEEEMDSGA |
| **17** | TEEEMDSGADW |
| **17** | TEEEMDSGADW |
| **17** | NETTDDGEPSGRGL |
| **17** | NTQKEMQTPRML |
| **17** | TDRPYGKMEQVY |
| **17** | NTQKEMQTPRML |
| **17** | TDRPYGKMEQVY |
| **17** | NTQKEMQTPRML |
| **17** | ANETTDDGEPSGRGL |
| **17** | ANTQKEMQTPRML |
| **17** | ANTQKEMQTPRML |
| **17** | ANTQKEMQTPRML |
| **17** | NETTDDGEPSGRGLVA |
| **17** | NTQKEMQTPRMLF |
| **17** | NTQKEMQTPRMLF |
| **17** | NTQKEMQTPRMLF |

Number = Table 3 Number

S3-2. Potential bioactive peptides of the Carbohydrate metabolism-Pepsin.

| **Number** | **Sequence** |
| --- | --- |
| **1** | NIVTTI |
| **1** | KTTPTL |
| **1** | GQGICNA |
| **1** | CNAVGMA |
| **1** | GVREHA |
| **1** | PGDWEA |
| **1** | EAKMQA |
| **1** | DGSTDLA |
| **1** | CNAVGMA |
| **1** | EDSADF |
| **1** | RQLNSA |
| **1** | EAKMQA |
| **1** | EGSYDGA |
| **1** | TDYCKA |
| **1** | RVVTPAA |
| **1** | GHGSMLL |
| **1** | RRRVGA |
| **1** | DHFVSI |
| **1** | TDKAVNA |
| **1** | VSIEQF |
| **1** | YDDNSI |
| **1** | GHGSMLL |
| **1** | LGEDEVA |
| **1** | GEDEVAL |
| **1** | EQLPTF |
| **1** | NPANPSF |
| **1** | SAGHGSML |
| **1** | ESAVVEGA |
| **1** | DDNSISI |
| **1** | CTDKPSL |
| **1** | KTTPTLL |
| **1** | SAGHGSML |
| **1** | TVRTVSL |
| **1** | GFGSPNKA |
| **1** | PCWEVF |
| **1** | ARRRVGA |
| **1** | RRRVGAA |
| **1** | KRGGYVL |
| **1** | QQFRQL |
| **1** | YNTDEY |
| **1** | VNRDRF |
| **1** | SIDGSTDL |
| **1** | MQTGEPF |
| **1** | EAQSSEY |
| **1** | DRHLKF |
| **1** | TGSEVDVI |
| **1** | GFTPEVVA |
| **1** | QQFRQL |
| **1** | DGAKRGGY |
| **1** | MQTGEPF |
| **1** | KESVLPSA |
| **1** | GGRPVRKA |
| **1** | NTDEYTI |
| **1** | GSPNKANTA |
| **1** | TRQGTGKL |
| **1** | MPNMQVF |
| **1** | HAKTTPTL |
| **1** | VGARVVTPA |
| **1** | GVREHAMA |
| **1** | DGMPTRKA |
| **1** | CTDKPSLI |
| **1** | MPNMQVF |
| **1** | VGRERRL |
| **1** | GVREHAMA |
| **1** | ATGSEVDVI |
| **1** | SDNSPDGEA |
| **1** | DGMPTRKA |
| **1** | DSVSMEDI |
| **1** | MPNMQVF |
| **1** | KEAEPEKA |
| **1** | TDRTGKNI |
| **1** | DSVSMEDI |
| **1** | AGGRPVRKA |
| **1** | DVPNGNSDL |
| **1** | TRQGTGKLA |
| **1** | TPEVVADRA |
| **1** | KACTDKPSL |
| **1** | GRSGPGDEVL |
| **1** | RYGVREHA |
| **1** | ERLREDGL |
| **1** | VNRDRFVL |
| **1** | GGRPVRKAW |
| **1** | RQGTPRMDA |
| **1** | DGMPTRKASA |
| **1** | TGSEVDVIVGA |
| **1** | RQGTPRMDA |
| **1** | DGMPTRKASA |
| **1** | ELMQTGEPF |
| **1** | GRSGPGDEVLA |
| **1** | DADGMPTRKA |
| **1** | SATDRTGKNI |
| **1** | ELMQTGEPF |
| **1** | VLTRQGTGKL |
| **1** | DADGMPTRKA |
| **1** | RAMPNMQVF |
| **1** | GFDSVSMEDI |
| **1** | ARQGTPRMDA |
| **1** | MSEDTSDRY |
| **1** | VLSDNSPDGEA |
| **1** | RAMPNMQVF |
| **1** | GFDSVSMEDI |
| **1** | ARQGTPRMDA |
| **1** | MSEDTSDRY |
| **1** | RAMPNMQVF |
| **1** | PSAVGRERRL |
| **1** | QSSEYKESVL |
| **1** | TVRTVSLPCW |
| **1** | QSSEYKESVL |
| **1** | AMSEDTSDRY |
| **1** | QSGHPGMPMGMA |
| **1** | DVPNGNSDLGSI |
| **1** | AMSEDTSDRY |
| **1** | MPNMQVFRPA |
| **1** | QSGHPGMPMGMA |
| **1** | QSGHPGMPMGMA |
| **1** | RQGTPRMDAVA |
| **1** | MPNMQVFRPA |
| **1** | QSGHPGMPMGMA |
| **1** | QSGHPGMPMGMA |
| **1** | RQGTPRMDAVA |
| **1** | MPNMQVFRPA |
| **1** | TDRTGKNIRY |
| **1** | MQTGEPFTDKA |
| **1** | SDNSPDGEAPQL |
| **1** | QSGHPGMPMGMA |
| **1** | QSGHPGMPMGMA |
| **1** | MQTGEPFTDKA |
| **1** | QSGHPGMPMGMA |
| **1** | NPSFVNRDRF |
| **1** | QVIDVPNGNSDL |
| **1** | QVIDVPNGNSDL |
| **1** | VGRERRLSVEA |
| **1** | DSVSMEDIQQF |
| **1** | DSVSMEDIQQF |
| **1** | REDGLTVRTVSL |
| **1** | EQFGRSGPGDEVL |
| **1** | MGDGCVMEGMSSEA |
| **1** | MGDGCVMEGMSSEA |
| **1** | MGDGCVMEGMSSEA |
| **1** | MGDGCVMEGMSSEA |
| **1** | QSGHPGMPMGMAPTA |
| **1** | QSGHPGMPMGMAPTA |
| **1** | QSGHPGMPMGMAPTA |
| **1** | QSGHPGMPMGMAPTA |
| **1** | QSGHPGMPMGMAPTA |
| **1** | QSGHPGMPMGMAPTA |
| **1** | QSGHPGMPMGMAPTA |
| **1** | MSEDTSDRYRSY |
| **1** | QSGHPGMPMGMAPTA |
| **1** | MSEDTSDRYRSY |
| **1** | CIMGDGCVMEGMSSEA |
| **1** | VEKAQSGHPGMPMGMA |
| **1** | CIMGDGCVMEGMSSEA |
| **1** | VEKAQSGHPGMPMGMA |
| **1** | CIMGDGCVMEGMSSEA |
| **1** | VEKAQSGHPGMPMGMA |
| **1** | CIMGDGCVMEGMSSEA |
| **1** | VEKAQSGHPGMPMGMA |
| **1** | MGDGCVMEGMSSEACSL |
| **1** | MGDGCVMEGMSSEACSL |
| **1** | MGDGCVMEGMSSEACSL |
| **1** | MGDGCVMEGMSSEACSL |
| **1** | VTTHDSVGCGEDGPTHQSI |
| **1** | TPGHPESNDTPGVEVTTGPL |
| **1** | EVPDEVMTHCRQKVETGA |
| **1** | EVPDEVMTHCRQKVETGA |
| **1** | EVPDEVMTHCRQKVETGAA |
| **1** | YVTTHDSVGCGEDGPTHQSI |
| **1** | EVPDEVMTHCRQKVETGAA |
| **1** | FEVPDEVMTHCRQKVETGA |
| **1** | FEVPDEVMTHCRQKVETGA |
| **1** | NSATPGHPESNDTPGVEVTTGPL |
| **1** | VTTHDSVGCGEDGPTHQSIEQL |
| **1** | TPGHPESNDTPGVEVTTGPLGQGI |
| **2** | RCDVPL |
| **2** | ATGVVVTA |
| **2** | ARCDVPL |
| **2** | PVSATVVI |
| **2** | VNAPTVDA |
| **2** | TTPAVHY |
| **2** | VPPPGAPVSA |
| **2** | SHNPVADNGI |
| **2** | VVRRRHRA |
| **2** | VVRRRHRAA |
| **2** | KMMEPDGSML |
| **2** | KMMEPDGSML |
| **2** | KMMEPDGSML |
| **2** | GRDTRPSSSPL |
| **2** | KMMEPDGSML |
| **2** | RCDVPLVPPPGA |
| **2** | GRDTRPSSSPLA |
| **2** | TGVVVTASHNPVA |
| **2** | KMMEPDGSMLEA |
| **2** | KMMEPDGSMLEA |
| **2** | KMMEPDGSMLEA |
| **2** | KMMEPDGSMLEA |
| **2** | VHYVVRRRHRA |
| **2** | DNGIKMMEPDGSML |
| **2** | DNGIKMMEPDGSML |
| **2** | DNGIKMMEPDGSML |
| **2** | TVVIGRDTRPSSSPL |
| **2** | DNGIKMMEPDGSML |
| **3** | PPPTTAA |
| **3** | TNVLHA |
| **3** | SPVAHF |
| **3** | IDGPSGI |
| **3** | TVEKLA |
| **3** | SLPSNF |
| **3** | AKRKCA |
| **3** | RNQGGF |
| **3** | SGGHNVI |
| **3** | GQPTVAL |
| **3** | KKQTLA |
| **3** | RVGFVL |
| **3** | QTHPNL |
| **3** | SAKKTY |
| **3** | KVTGNGGA |
| **3** | STPEQF |
| **3** | QTHPNL |
| **3** | DHATRI |
| **3** | QQLNDI |
| **3** | FGQPTVA |
| **3** | KCGGFPL |
| **3** | EAGKTGY |
| **3** | DCNLCY |
| **3** | QQLNDI |
| **3** | SELTGNL |
| **3** | TMMMNI |
| **3** | IGEEVEA |
| **3** | MPDVCAL |
| **3** | VAPPPTTA |
| **3** | TMMMNI |
| **3** | DANEVTI |
| **3** | TEVTAEL |
| **3** | MPDVCAL |
| **3** | ESRNLF |
| **3** | TMMMNI |
| **3** | GEKVPPF |
| **3** | KVTGNGGAA |
| **3** | ESDVDVI |
| **3** | ESERLF |
| **3** | TMMMNI |
| **3** | NEVTITL |
| **3** | PEGVIEF |
| **3** | VVLEEMA |
| **3** | RLTEDY |
| **3** | DTATKVY |
| **3** | DEVVVKL |
| **3** | DAESRNL |
| **3** | VVLEEMA |
| **3** | RATVEKL |
| **3** | EQRDSW |
| **3** | EMVSNEL |
| **3** | LQTHPNL |
| **3** | KKTYHF |
| **3** | FRNQGGF |
| **3** | EMVSNEL |
| **3** | TKVYSEL |
| **3** | TSPPEEW |
| **3** | GGDDSNTNA |
| **3** | MGRSASHI |
| **3** | SAKVTGNGGA |
| **3** | SVVHQSPL |
| **3** | RPRMPPL |
| **3** | MGRSASHI |
| **3** | STPEQFTA |
| **3** | RPRMPPL |
| **3** | VRLMGRSA |
| **3** | AEQRDSW |
| **3** | EMVSNELA |
| **3** | NGLKTRVI |
| **3** | VRLMGRSA |
| **3** | QTHPNLTL |
| **3** | EMVSNELA |
| **3** | PSNFDCNL |
| **3** | EFMPDVCA |
| **3** | QTHPNLTL |
| **3** | EFMPDVCA |
| **3** | RCPGPIQY |
| **3** | IEMVSNEL |
| **3** | ASVVHQSPL |
| **3** | SGGHNVISGL |
| **3** | QVVEGAPTSA |
| **3** | IEMVSNEL |
| **3** | PLTMMMNI |
| **3** | QVVEGAPTSA |
| **3** | PLTMMMNI |
| **3** | RPRMPPLL |
| **3** | PLTMMMNI |
| **3** | DEVVVKLDA |
| **3** | QCVTSMVEL |
| **3** | RPRMPPLL |
| **3** | PLTMMMNI |
| **3** | QCVTSMVEL |
| **3** | QCVTSMVEL |
| **3** | GCPKTIDGDL |
| **3** | QCVTSMVEL |
| **3** | SFDEVVVKL |
| **3** | CTGTYTEVTA |
| **3** | SGGPASGGHNVI |
| **3** | RGCFQVVEGA |
| **3** | DGPSGICTGTY |
| **3** | AQCVTSMVEL |
| **3** | HMVGSGRTKI |
| **3** | EQRDSWRL |
| **3** | GGDDSNTNAML |
| **3** | AQCVTSMVEL |
| **3** | HMVGSGRTKI |
| **3** | QCVTSMVELI |
| **3** | GGDDSNTNAML |
| **3** | SYRPRMPPL |
| **3** | QCVTSMVELI |
| **3** | QCVTSMVELI |
| **3** | GEKVPPFSPVA |
| **3** | PTSAESDVDVI |
| **3** | ESDVDVIRSL |
| **3** | SYRPRMPPL |
| **3** | QCVTSMVELI |
| **3** | RNEHVEMSF |
| **3** | TEDYRCPGPI |
| **3** | SNLTSPPEEW |
| **3** | VVIGGDDSNTNA |
| **3** | SVVHQSPLHSA |
| **3** | RNEHVEMSF |
| **3** | KTRVIGCPKTI |
| **3** | GEEVEAKKQTL |
| **3** | MERNMDSKMF |
| **3** | ERRKGKNVPVI |
| **3** | MERNMDSKMF |
| **3** | MERNMDSKMF |
| **3** | MERNMDSKMF |
| **3** | TSPPEEWKCGGF |
| **3** | RNEHVEMSFGF |
| **3** | KRKCAGEKVPPF |
| **3** | RNEHVEMSFGF |
| **3** | VPNRGTPVTMGNVL |
| **3** | VPNRGTPVTMGNVL |
| **3** | MERNMDSKMFGF |
| **3** | MERNMDSKMFGF |
| **3** | MERNMDSKMFGF |
| **3** | MERNMDSKMFGF |
| **3** | DGDLRNEHVEMSF |
| **3** | DGDLRNEHVEMSF |
| **3** | LVPNRGTPVTMGNVL |
| **3** | LVPNRGTPVTMGNVL |
| **3** | ERRKGKNVPVIKKA |
| **3** | DHLMERNMDSKMF |
| **3** | DHLMERNMDSKMF |
| **3** | DHLMERNMDSKMF |
| **3** | DHLMERNMDSKMF |
| **3** | MMDRDPHGNVQVSKI |
| **3** | MMDRDPHGNVQVSKI |
| **3** | RNQGGFHMVGSGRTKI |
| **3** | MMDRDPHGNVQVSKI |
| **3** | RNQGGFHMVGSGRTKI |
| **3** | HMVGSGRTKISTPEQF |
| **3** | HMVGSGRTKISTPEQF |
| **3** | VPNRGTPVTMGNVLRVGF |
| **3** | VPNRGTPVTMGNVLRVGF |
| **3** | TMMMNIERRKGKNVPVI |
| **3** | TMMMNIERRKGKNVPVI |
| **3** | TMMMNIERRKGKNVPVI |
| **3** | TMMMNIERRKGKNVPVI |
| **3** | EQLMMDRDPHGNVQVSKI |
| **3** | EQLMMDRDPHGNVQVSKI |
| **3** | EQLMMDRDPHGNVQVSKI |
| **3** | MMDRDPHGNVQVSKIESERL |
| **3** | MMDRDPHGNVQVSKIESERL |
| **3** | MMDRDPHGNVQVSKIESERL |
| **4** | KTYDGA |
| **4** | PGRVNL |
| **4** | LTSKPL |
| **4** | DATERA |
| **4** | EEGGIPA |
| **4** | DPKTRA |
| **4** | AKVPCGI |
| **4** | GGGVSSSAA |
| **4** | ASVSDDL |
| **4** | GGCTVSLA |
| **4** | LEMSTGA |
| **4** | SDPSVVI |
| **4** | GEHTDY |
| **4** | LEMSTGA |
| **4** | APGRVNL |
| **4** | TSKPLDA |
| **4** | RAHHVI |
| **4** | NDGWVF |
| **4** | LEKSTY |
| **4** | EMSTGAF |
| **4** | SGDRKW |
| **4** | TEHEYA |
| **4** | EMSTGAF |
| **4** | MVGARRA |
| **4** | CKVDGVF |
| **4** | SLRDDY |
| **4** | PGRVNLI |
| **4** | EVSVPEI |
| **4** | FVSDVPI |
| **4** | MVGARRA |
| **4** | RVLEVPA |
| **4** | KREAVDA |
| **4** | EGDVVEF |
| **4** | TCFVTRA |
| **4** | SGDRKWA |
| **4** | MGESHASL |
| **4** | EKAEEGGI |
| **4** | IGEHTDY |
| **4** | ACKVDGVF |
| **4** | MGESHASL |
| **4** | GSRITGGGF |
| **4** | NYVVGMVA |
| **4** | NYVVGMVA |
| **4** | SGGVKATCF |
| **4** | VVGMVAMY |
| **4** | VVGMVAMY |
| **4** | VVGMVAMY |
| **4** | DVAGDVKPL |
| **4** | DPKTRARA |
| **4** | FEGDVVEF |
| **4** | EVSVPEIDA |
| **4** | GDFVTVGRL |
| **4** | EKSTYMVGA |
| **4** | EKSTYMVGA |
| **4** | SEDVRTQEA |
| **4** | TGGGFGGCTVSL |
| **4** | PERRDSCKA |
| **4** | EGDVVEFDVA |
| **4** | RGQATEHEY |
| **4** | KVPCGIMDQL |
| **4** | KVPCGIMDQL |
| **4** | PERRDSCKAA |
| **4** | CKVDGVFGSRI |
| **4** | GEHTDYNDGW |
| **4** | KHGSEEKPVKA |
| **4** | VSDVPIGGGVSSSA |
| **4** | VVTNSHVEHDL |
| **4** | VTVGRLMGESHA |
| **4** | VTVGRLMGESHA |
| **4** | AKHGSEEKPVKA |
| **4** | GGEGTKSRVVCEA |
| **4** | SVSDDLDPKTRA |
| **4** | RDDYEVSVPEI |
| **4** | KHGSEEKPVKAL |
| **4** | DCRSHEVTPVPL |
| **4** | GDVKPLSGDRKW |
| **4** | SEDVRTQEARKA |
| **4** | GGEGTKSRVVCEAF |
| **4** | IDCRSHEVTPVPL |
| **4** | HHVISEDVRTQEA |
| **4** | SGSEYPERRDSCKA |
| **4** | RRAGGEGTKSRVVCEA |
| **4** | VVTNSHVEHDLSGSEY |
| **4** | SDPSVVIVVTNSHVEHDL |
| **4** | DCRSHEVTPVPLSDPSVVI |
| **5** | SVEESL |
| **5** | SGKFPGA |
| **5** | SPLGGVF |
| **5** | DVVLSY |
| **5** | KPVANVA |
| **5** | LRDVKA |
| **5** | RDVKAI |
| **5** | VQCHDI |
| **5** | ETVLGKA |
| **5** | MTSGLSGA |
| **5** | FDVSPY |
| **5** | SVEESLA |
| **5** | DVSPLGF |
| **5** | MTSGLSGA |
| **5** | DVSPYY |
| **5** | TKAETVL |
| **5** | VKLGVNF |
| **5** | GESTFDF |
| **5** | DSGLSPHL |
| **5** | TDAGPPPW |
| **5** | NVDRLDL |
| **5** | DRVPPGTL |
| **5** | GPPPWHPA |
| **5** | SVTSTVVGI |
| **5** | CKERGSSL |
| **5** | RLNVDRL |
| **5** | RESGKVRA |
| **5** | DQVVNETL |
| **5** | DLVQCHDI |
| **5** | GSTGIDVSPL |
| **5** | RRSLGSTGI |
| **5** | CKERGSSLA |
| **5** | VVGSKVGRY |
| **5** | GGVFGDVDEA |
| **5** | SPHLRRSL |
| **5** | DGVRSVHEA |
| **5** | VQCHDIEF |
| **5** | KELPRDSF |
| **5** | LCKERGSSL |
| **5** | VMMTPMDQA |
| **5** | VMMTPMDQA |
| **5** | VMMTPMDQA |
| **5** | VLDRVPPGTL |
| **5** | VMMTPMDQA |
| **5** | DQVVNETLPA |
| **5** | RVTASVEESL |
| **5** | VMMTPMDQAL |
| **5** | VMMTPMDQAL |
| **5** | VMMTPMDQAL |
| **5** | RESGKVRAVGI |
| **5** | VMMTPMDQAL |
| **5** | DPLSVTSTVVGI |
| **5** | GDLDQVVNETL |
| **5** | SVTSTVVGIDSTA |
| **5** | VKLRESGKVRA |
| **5** | DRVPPGTLDVVL |
| **5** | DGVRSVHEAVKL |
| **5** | KNVAVMMTPMDQA |
| **5** | KNVAVMMTPMDQA |
| **5** | KNVAVMMTPMDQA |
| **5** | KNVAVMMTPMDQA |
| **5** | VVGSKVGRYGESTF |
| **5** | GDVDEADGVRSVHEA |
| **5** | PRDSFVVGSKVGRY |
| **6** | MKHVAA |
| **6** | PMVKAI |
| **6** | MTTVHA |
| **6** | DGSEDF |
| **6** | MKHVAA |
| **6** | PMVKAI |
| **6** | MTTVHA |
| **6** | LTKEKA |
| **6** | SCTTNGL |
| **6** | AKETTY |
| **6** | PSLVGKL |
| **6** | FRVPTI |
| **6** | MLNPNF |
| **6** | SGRVVDL |
| **6** | DVSVVDL |
| **6** | MLNPNF |
| **6** | ESTGVFL |
| **6** | KAVTKVI |
| **6** | DNEYGY |
| **6** | SCTTNGLA |
| **6** | LMTTVHA |
| **6** | VGKLTGMA |
| **6** | GYSDEPL |
| **6** | DGSEDFI |
| **6** | DGGAKKVI |
| **6** | LMTTVHA |
| **6** | HDEFEI |
| **6** | VGKLTGMA |
| **6** | CAHVKKL |
| **6** | KDDSQTI |
| **6** | NPNFVKL |
| **6** | VTKVIPSL |
| **6** | EGDLRSSI |
| **6** | SEGEMKGF |
| **6** | SEGEMKGF |
| **6** | TKEKAQSI |
| **6** | SCASCTTNGL |
| **6** | MTTVHAMTA |
| **6** | GYSGRVVDL |
| **6** | PAKDDSQTI |
| **6** | MTTVHAMTA |
| **6** | VSTDFEGDL |
| **6** | MTTVHAMTA |
| **6** | SEGEMKGFL |
| **6** | KETTYEEI |
| **6** | SEGEMKGFL |
| **6** | VMGVNQETY |
| **6** | VMGVNQETY |
| **6** | VVDSSRKDW |
| **6** | SDEPLVSTDF |
| **6** | DVSVVDLTCRL |
| **6** | SGRVVDLMKHVA |
| **6** | RVPTIDVSVVDL |
| **6** | SGRVVDLMKHVA |
| **6** | TQAVVDSSRKDW |
| **6** | HVKKLSEGEMKGF |
| **6** | HVKKLSEGEMKGF |
| **6** | VVDSSRKDWRGGRA |
| **6** | VMGVNQETYDGSEDF |
| **6** | VMGVNQETYDGSEDF |
| **6** | KDDSQTIVMGVNQETY |
| **6** | KDDSQTIVMGVNQETY |
| **7** | PVATRL |
| **7** | LEGKVL |
| **7** | SGKRVL |
| **7** | GDGVAEI |
| **7** | NGPAGVF |
| **7** | GVTEFL |
| **7** | PVDVVAA |
| **7** | KKGVKI |
| **7** | IGGGDSVA |
| **7** | KATTSVA |
| **7** | DCKTVL |
| **7** | SRTQSI |
| **7** | NDTEFA |
| **7** | LSRTQSI |
| **7** | RPSVAGL |
| **7** | PEGTMGL |
| **7** | LPVDVVA |
| **7** | DKVDKL |
| **7** | VEDDKI |
| **7** | TRLSEL |
| **7** | PEGTMGL |
| **7** | PSCFPVA |
| **7** | TDDTRI |
| **7** | YKEETA |
| **7** | AKKGVKI |
| **7** | EKMSHI |
| **7** | GVIESML |
| **7** | ADCKTVL |
| **7** | PDANTQL |
| **7** | EKMSHI |
| **7** | GVIESML |
| **7** | LENVRF |
| **7** | SSSSVFVA |
| **7** | KKGVKII |
| **7** | PSGVVMQA |
| **7** | PSGVVMQA |
| **7** | LSRTQSI |
| **7** | DMFVNDA |
| **7** | AEKMSHI |
| **7** | IPEGTMGL |
| **7** | KKDVKMA |
| **7** | VIVGGMVF |
| **7** | DMFVNDA |
| **7** | ENVRFY |
| **7** | AEKMSHI |
| **7** | KKSIKDL |
| **7** | IPEGTMGL |
| **7** | KKDVKMA |
| **7** | HRAHGSTA |
| **7** | VIVGGMVF |
| **7** | PDCIGDGVA |
| **7** | VGGMVFTF |
| **7** | APSGVVMQA |
| **7** | DCKTVLW |
| **7** | EGKVLPGVA |
| **7** | VGGMVFTF |
| **7** | APSGVVMQA |
| **7** | RGLSTGSSL |
| **7** | DLSGKRVL |
| **7** | DKVDKLVI |
| **7** | LKKDVKMA |
| **7** | VSNPKRPF |
| **7** | NTQLVSVDA |
| **7** | TDDTRIRA |
| **7** | LKKDVKMA |
| **7** | NVPLDGKTI |
| **7** | PSGVVMQATA |
| **7** | VEDDKIEL |
| **7** | VGGSKVSSKI |
| **7** | DQGPKSTEL |
| **7** | PSGVVMQATA |
| **7** | HGSTAGVTEF |
| **7** | VSNPKRPFA |
| **7** | SNRRVQVPA |
| **7** | VRCDLNVPL |
| **7** | TTSVASSSSVF |
| **7** | GAVSNPKRPF |
| **7** | IVGGSKVSSKI |
| **7** | VSGMSNGDVVL |
| **7** | DQGPKSTELI |
| **7** | VSGMSNGDVVL |
| **7** | SNRRVQVPAA |
| **7** | SRTQSISSCSI |
| **7** | EKMSHISTGGGA |
| **7** | ESMLDKVDKL |
| **7** | KEETANDTEF |
| **7** | EKMSHISTGGGA |
| **7** | VSGMSNGDVVLL |
| **7** | ESMLDKVDKL |
| **7** | VASNRRVQVPA |
| **7** | VSGMSNGDVVLL |
| **7** | GRPKSGPEDKF |
| **7** | VGGSKVSSKIGVI |
| **7** | DGKTITDDTRI |
| **7** | SGKRVLVRCDL |
| **7** | KKDVKMAPDCI |
| **7** | STGSSLVEDDKI |
| **7** | KKDVKMAPDCI |
| **7** | EIVSGMSNGDVVL |
| **7** | EIVSGMSNGDVVL |
| **7** | GRPKSGPEDKFSL |
| **7** | SSHLGRPKSGPEDKF |
| **7** | VPSSPSSKRRDRSSL |
| **7** | PEGTMGLDQGPKSTEL |
| **7** | PEGTMGLDQGPKSTEL |
| **7** | VAVPSSPSSKRRDRSSL |
| **7** | VPSSPSSKRRDRSSLPL |
| **8** | SIETPL |
| **8** | PSTELL |
| **8** | AGVVDVI |
| **8** | ASGSSCY |
| **8** | HLTTGF |
| **8** | SHGTLVA |
| **8** | EGGGRPASA |
| **8** | GSRRLI |
| **8** | ETPLVF |
| **8** | GGTATDGY |
| **8** | VEHAGGTA |
| **8** | SDDDDDA |
| **8** | PPDVPTGA |
| **8** | QSTHRF |
| **8** | GLGPPGVY |
| **8** | QSTHRF |
| **8** | GCCVYSSA |
| **8** | EGGGRPASA |
| **8** | VDLQQQA |
| **8** | HPVATQY |
| **8** | ASDDDDDA |
| **8** | GPPGVYSF |
| **8** | MTRGVVVA |
| **8** | EMGVTDDA |
| **8** | DDPSPVVL |
| **8** | MTRGVVVA |
| **8** | EMGVTDDA |
| **8** | LQSTHRF |
| **8** | PPDVPTGAY |
| **8** | RSLSHDGY |
| **8** | SDDDDDADA |
| **8** | LMTRGVVVA |
| **8** | PMASHPPVL |
| **8** | LMTRGVVVA |
| **8** | PMASHPPVL |
| **8** | GVVDVIEKL |
| **8** | VFPPDVPTGA |
| **8** | DDPSPVVLPA |
| **8** | QSTHRFRL |
| **8** | TDGYGRRVL |
| **8** | GQREQGVGTI |
| **8** | QSTHRFRL |
| **8** | EMGVTDDAHL |
| **8** | SGSSCYSHGTL |
| **8** | SHPPVLSSEF |
| **8** | EMGVTDDAHL |
| **8** | RLDDPSPVVL |
| **8** | AGQREQGVGTI |
| **8** | GQREQGVGTIW |
| **8** | EEMRRDETDG |
| **8** | EEMRRDETDG |
| **8** | QRRPRRCGGVY |
| **8** | QRRPRRCGGVY |
| **8** | MTRGVVVAPSTEL |
| **8** | MTRGVVVAPSTEL |
| **8** | KRKSPTGTRGRY |
| **8** | VAEEMRRDETDG |
| **8** | GRRVLEMGVTDDA |
| **8** | VAEEMRRDETDG |
| **8** | GRRVLEMGVTDDA |
| **8** | DAQRRPRRCGGVY |
| **8** | KRKSPTGTRGRYVDL |
| **8** | SVYKRKSPTGTRGRY |
| **8** | QRRPRRCGGVYGSRRL |
| **8** | QRRPRRCGGVYGSRRL |
| **9** | GPRIVI |
| **9** | NGSLRL |
| **9** | VGVLGDI |
| **9** | TRPSLL |
| **9** | ESVKLL |
| **9** | INRTKA |
| **9** | GASREEA |
| **9** | GKDGVSVA |
| **9** | HDTLTY |
| **9** | VGLHDTL |
| **9** | EDPACRA |
| **9** | ERMTLL |
| **9** | QLGREDA |
| **9** | SREEANA |
| **9** | ERMTLL |
| **9** | QLGREDA |
| **9** | FERMTL |
| **9** | AGKDGVSVA |
| **9** | FERMTL |
| **9** | NRTKANL |
| **9** | EEEEVDA |
| **9** | VNSNVSTL |
| **9** | TLGGKTKF |
| **9** | GRPQNAGVA |
| **9** | ELGRPQNA |
| **9** | EKAGKKGTL |
| **9** | VGVKKRKGA |
| **9** | STRDRVVL |
| **9** | GDKEMKVSA |
| **9** | GDKEMKVSA |
| **9** | TEPDPPSSSA |
| **9** | GKDGVSVADF |
| **9** | GGKTKFGNGY |
| **9** | GGGPTGNEDVGA |
| **9** | GTRFESVKL |
| **9** | VLVNSNVSTL |
| **9** | VNSNVSTLTL |
| **9** | GDAEEEEVDA |
| **9** | GDKEMKVSAI |
| **9** | TVDVDTDRY |
| **9** | GDKEMKVSAI |
| **9** | TEPDPPSSSAL |
| **9** | ATVDVDTDRY |
| **9** | VGVKKRKGAVL |
| **9** | EEEEVDAMTY |
| **9** | EEEEVDAMTY |
| **9** | STRDRVVLEDPA |
| **9** | MTYGGGPTGNEDVGA |
| **9** | MTYGGGPTGNEDVGA |
| **9** | GREDATEPDPPSSSA |
| **9** | GKKGTLSTRDRVVL |
| **9** | GGGPTGNEDVGAVGVKKRKGA |
| **9** | TVDVDTDRYGDKEMKVSA |
| **9** | TVDVDTDRYGDKEMKVSA |
| **10** | AVRMDA |
| **10** | STTWVA |
| **10** | FVTMVA |
| **10** | VTAVGVGA |
| **10** | PTRSTL |
| **10** | AVRMDA |
| **10** | FVTMVA |
| **10** | GDVRTF |
| **10** | GQVHAKA |
| **10** | VRMDAGA |
| **10** | GRAVPRA |
| **10** | VRMDAGA |
| **10** | FCEKPI |
| **10** | PTRRHA |
| **10** | EIESVY |
| **10** | PTRSTLA |
| **10** | DTVVTVL |
| **10** | GYDQRI |
| **10** | KMSNGAF |
| **10** | EFSTTW |
| **10** | KMSNGAF |
| **10** | DPFEKF |
| **10** | SVQEGRL |
| **10** | IGDVRTF |
| **10** | APTRRHA |
| **10** | GSPTPFHA |
| **10** | SHDFDMA |
| **10** | DMASHDF |
| **10** | ESVYVTGA |
| **10** | SHDFDMA |
| **10** | DMASHDF |
| **10** | PTRRHAL |
| **10** | QDGAVPVGGA |
| **10** | RDSHRDA |
| **10** | QDGAVPVGGA |
| **10** | DEVDLVKA |
| **10** | VTMVAQDGA |
| **10** | EKFGRSVA |
| **10** | VPVGGADGRA |
| **10** | DQRIEVF |
| **10** | VTMVAQDGA |
| **10** | GDVRTFTI |
| **10** | VGFQRRF |
| **10** | DTVTVSTEA |
| **10** | NPDVDGVVI |
| **10** | VRLDEVDL |
| **10** | ENSRRCGF |
| **10** | GCGRIGQVHA |
| **10** | QLSVQEGRL |
| **10** | CEKPISNDL |
| **10** | TSRDPSPPPA |
| **10** | QLSVQEGRL |
| **10** | GDLDTVVTVL |
| **10** | QRRFDSNF |
| **10** | TRSRPVGGRA |
| **10** | VGVGAPTRSTL |
| **10** | QRRFDSNF |
| **10** | QKVKSVVSSGA |
| **10** | QKVKSVVSSGA |
| **10** | DTVTVSTEAGI |
| **10** | SGSPGTPKPVGI |
| **10** | VANPDVDGVVI |
| **10** | GSPTPTDECSL |
| **10** | TRSRPVGGRAA |
| **10** | EVVERTGVKL |
| **10** | RDSHRDAGVGA |
| **10** | GGKGSVTGSNKSA |
| **10** | SVQEGRLVRL |
| **10** | LTRSRPVGGRA |
| **10** | QKVKSVVSSGAI |
| **10** | ENSRRCGFGY |
| **10** | QKVKSVVSSGAI |
| **10** | NNSVPSTPPDSL |
| **10** | TITSRDPSPPPA |
| **10** | GTIENSRRCGF |
| **10** | EVVERTGVKLL |
| **10** | FGSPTPTDECSL |
| **10** | SGSPGTPKPVGIGI |
| **10** | EASGSPGTPKPVGI |
| **10** | ANNSVPSTPPDSL |
| **10** | TSRDPSPPPADY |
| **10** | DTVVTVLKMSNGA |
| **10** | DTVVTVLKMSNGA |
| **10** | NPDVDGVVIGSPTPF |
| **10** | EVFGGKGSVTGSNKSA |
| **10** | DSCLEVVERTGVKL |
| **10** | DSNFQKVKSVVSSGA |
| **10** | NNSVPSTPPDSLPHRTL |
| **10** | GSPTPTDECSLRDSHRDA |
| **10** | GGKGSVTGSNKSADTVTVSTEA |
| **10** | PVPHPDSTPPPHRVCCRSQSA |
| **10** | PVPHPDSTPPPHRVCCRSQSAPPRSI |
| **10** | PHRTLPVPHPDSTPPPHRVCCRSQSA |
| **11** | RGKEVA |
| **11** | NKGTKL |
| **11** | TTESGGI |
| **11** | GLGDTVI |
| **11** | VRSSAGL |
| **11** | SSEGIKA |
| **11** | WVRSSA |
| **11** | AGKDVDL |
| **11** | SVDNSQA |
| **11** | SMPVQY |
| **11** | LKRTVL |
| **11** | SMPVQY |
| **11** | RDSLRL |
| **11** | EVLDESA |
| **11** | SNLGDGSI |
| **11** | NKGTKLI |
| **11** | TTESGGII |
| **11** | GDKDKDL |
| **11** | HMREHL |
| **11** | HMREHL |
| **11** | GDGSIGMVI |
| **11** | GDKDKDLA |
| **11** | GDGSIGMVI |
| **11** | TGEDGFEI |
| **11** | PLRGKEVA |
| **11** | SVDNSQAVA |
| **11** | AHMREHL |
| **11** | AHMREHL |
| **11** | RGKDAVPW |
| **11** | PCHTRCGY |
| **11** | GYSMPVQY |
| **11** | DETTSPVEA |
| **11** | GYSMPVQY |
| **11** | EISVDNSQA |
| **11** | SVLTTESGGI |
| **11** | TWKRRRA |
| **11** | NAGDKDKDL |
| **11** | HMREHLSA |
| **11** | GKDVDLEVL |
| **11** | HMREHLSA |
| **11** | GDTVIMKQL |
| **11** | KRTVLHDF |
| **11** | DETTSPVEAA |
| **11** | GDTVIMKQL |
| **11** | KRRRAEGGL |
| **11** | GIPCHTRCGY |
| **11** | DVSHMGQVRL |
| **11** | DVSHMGQVRL |
| **11** | KKPFNKGTKL |
| **11** | SMPVQYSSEGI |
| **11** | GEEVGEVTSGGW |
| **11** | SMPVQYSSEGI |
| **11** | QVKSPVVKTSY |
| **11** | HESHGGKMVPF |
| **11** | QVKSPVVKTSY |
| **11** | HESHGGKMVPF |
| **11** | STPRRHMSSEA |
| **11** | STPRRHMSSEA |
| **11** | FDVSHMGQVRL |
| **11** | HESHGGKMVPFA |
| **11** | FDVSHMGQVRL |
| **11** | HESHGGKMVPFA |
| **11** | GNDIDETTSPVEA |
| **11** | QVKSPVVKTSYF |
| **11** | STPRRHMSSEAL |
| **11** | QVKSPVVKTSYF |
| **11** | STPRRHMSSEAL |
| **11** | TASTPRRHMSSEA |
| **11** | TASTPRRHMSSEA |
| **11** | NEAGEEVGEVTSGGW |
| **11** | PCHTRCGYTGEDGF |
| **11** | GEEVGEVTSGGWGPKA |
| **11** | TRRRMRTVVEKGGA |
| **11** | HDFHESHGGKMVPF |
| **11** | TRRRMRTVVEKGGA |
| **11** | HDFHESHGGKMVPF |
| **11** | DVSHMGQVRLRGKDA |
| **11** | DVSHMGQVRLRGKDA |
| **11** | RGKEVAQVKSPVVKTSY |
| **11** | DGITRRRMRTVVEKGGA |
| **11** | DGITRRRMRTVVEKGGA |
| **11** | TRRRMRTVVEKGGARKKI |
| **11** | TRRRMRTVVEKGGARKKI |
| **12** | QVRMAA |
| **12** | NRSAVL |
| **12** | MTPGSVA |
| **12** | TPDEGF |
| **12** | SPEVEL |
| **12** | QVRMAA |
| **12** | QVRMAA |
| **12** | VEQKTA |
| **12** | DGDRTL |
| **12** | DCTVLGA |
| **12** | MTPGSVA |
| **12** | NCVPVF |
| **12** | VNDGEF |
| **12** | PSSEVY |
| **12** | DYDGTL |
| **12** | RDGLNL |
| **12** | EQLKVA |
| **12** | VDDLDL |
| **12** | QVRMAA |
| **12** | IPHDTL |
| **12** | AVRNEI |
| **12** | DGTLTSI |
| **12** | TGCSRAL |
| **12** | CKGTLW |
| **12** | SGVKIGF |
| **12** | EESISF |
| **12** | NLEHEA |
| **12** | HDLKEA |
| **12** | NRGSLGL |
| **12** | LQVRMA |
| **12** | RPSEAVA |
| **12** | KEASEPA |
| **12** | MTPGSVAA |
| **12** | LQVRMA |
| **12** | SFESRL |
| **12** | ASPEVEL |
| **12** | KWDDDA |
| **12** | MTPGSVAA |
| **12** | DADREF |
| **12** | EENRTL |
| **12** | EKLCGVL |
| **12** | VRVNPW |
| **12** | HSSQQW |
| **12** | FHMVSSA |
| **12** | LTPDEGF |
| **12** | VHDYHL |
| **12** | DGDRTLI |
| **12** | DMVVQKA |
| **12** | NCVPVFI |
| **12** | FHMVSSA |
| **12** | HSSNEVGA |
| **12** | MGFQRY |
| **12** | DMVVQKA |
| **12** | PWRNEL |
| **12** | GLNNSPTI |
| **12** | NNSPTIGI |
| **12** | MGFQRY |
| **12** | GVDPERF |
| **12** | DPSVKQF |
| **12** | DGRHVML |
| **12** | KRDSTTGA |
| **12** | QGFCKGTL |
| **12** | TDRVAEF |
| **12** | SVQVVSGY |
| **12** | HSSQQWA |
| **12** | DGRHVML |
| **12** | RKGLEQL |
| **12** | KRRADKA |
| **12** | VEQKTAGI |
| **12** | QGFCKGTL |
| **12** | EFKERF |
| **12** | ETLVTGSY |
| **12** | PTLTDRVA |
| **12** | TEQSSQMA |
| **12** | DTLSKDPI |
| **12** | DREFGSW |
| **12** | TEQSSQMA |
| **12** | SGQKRRGL |
| **12** | TTRSGHMI |
| **12** | DMVVQKAI |
| **12** | AKRDSTTGA |
| **12** | TPDEGFGDA |
| **12** | FEENRTL |
| **12** | MNVNRMF |
| **12** | LHSSNEVGA |
| **12** | HSSNEVGAL |
| **12** | TDRTDGSY |
| **12** | TTRSGHMI |
| **12** | DMVVQKAI |
| **12** | QVVVKVGPL |
| **12** | MNVNRMF |
| **12** | KEMRDHL |
| **12** | TSTGLRKGL |
| **12** | QVVVKVGPL |
| **12** | MNVNRMF |
| **12** | KEMRDHL |
| **12** | SEFTGCSRA |
| **12** | PWPSSEVY |
| **12** | VVTPIRDGL |
| **12** | SGQKRRGLA |
| **12** | QVVVKVGPLA |
| **12** | PHDTLKHF |
| **12** | VRVNPWDL |
| **12** | VSTSEGKGQL |
| **12** | RDKVVEVY |
| **12** | QVVVKVGPLA |
| **12** | EHEANRGSL |
| **12** | SSAVRVNPW |
| **12** | RSRLSGVKI |
| **12** | MSGRKTEVL |
| **12** | KRDSTTGAW |
| **12** | PKMRGKVVL |
| **12** | VRNEIREL |
| **12** | VSAHSSQQW |
| **12** | MSGRKTEVL |
| **12** | PKMRGKVVL |
| **12** | PSSEVYRVL |
| **12** | SKDPINDVY |
| **12** | VNDGEFGKW |
| **12** | QDMPEPPDF |
| **12** | DTMDGSMKW |
| **12** | QDMPEPPDF |
| **12** | QDMPEPPDF |
| **12** | RKKNSKEW |
| **12** | TCTVGVKPSNA |
| **12** | SVQVVSGYGW |
| **12** | EEFNCVPVF |
| **12** | DTMDGSMKW |
| **12** | TEQSSQMAHA |
| **12** | QDMPEPPDF |
| **12** | DTMDGSMKW |
| **12** | TEQSSQMAHA |
| **12** | DYDGRHVML |
| **12** | QAKEMRDHL |
| **12** | IVSTSEGKGQL |
| **12** | DYDGRHVML |
| **12** | MNKGVMVETI |
| **12** | QAKEMRDHL |
| **12** | QAKEMRDHL |
| **12** | IMSGRKTEVL |
| **12** | MNKGVMVETI |
| **12** | QAKEMRDHL |
| **12** | IMSGRKTEVL |
| **12** | MNKGVMVETI |
| **12** | CGVLDMVVQKA |
| **12** | CGVLDMVVQKA |
| **12** | PRGVRPRGRGA |
| **12** | TSITEQSSQMA |
| **12** | TPFSVQVVSGY |
| **12** | SPEVELKRRA |
| **12** | LQDMPEPPDF |
| **12** | AMNKGVMVETI |
| **12** | TSITEQSSQMA |
| **12** | LQDMPEPPDF |
| **12** | GVDPERFSEGL |
| **12** | MYTDRTDGSY |
| **12** | AMNKGVMVETI |
| **12** | VSTSEGKGQLVL |
| **12** | MSGRKTEVLEA |
| **12** | MYTDRTDGSY |
| **12** | AMNKGVMVETI |
| **12** | MNKGVMVETIL |
| **12** | MSGRKTEVLEA |
| **12** | YRKKNSKEW |
| **12** | TDHTEHTTRF |
| **12** | MNKGVMVETIL |
| **12** | KEMRDHLESL |
| **12** | MNKGVMVETIL |
| **12** | QDMPEPPDFVL |
| **12** | KEMRDHLESL |
| **12** | NDVDEGRVKCF |
| **12** | QDMPEPPDFVL |
| **12** | DTHLDPSVKQF |
| **12** | QDMPEPPDFVL |
| **12** | QDMPEPPDFVL |
| **12** | DTMDGSMKWEL |
| **12** | DTAPKMRGKVVL |
| **12** | SEPAQVVVKVGPL |
| **12** | DTMDGSMKWEL |
| **12** | RVYMNVNRMF |
| **12** | DTAPKMRGKVVL |
| **12** | DTMDGSMKWEL |
| **12** | RVYMNVNRMF |
| **12** | RVYMNVNRMF |
| **12** | PRGVRPRGRGASI |
| **12** | NDVDEGRVKCFL |
| **12** | TCTVGVKPSNARY |
| **12** | CCGDDRTDEDMF |
| **12** | RVSHIGVDPERF |
| **12** | DGRHVMLRVSHI |
| **12** | CCGDDRTDEDMF |
| **12** | PKMRGKVVLVQVA |
| **12** | DGRHVMLRVSHI |
| **12** | PKMRGKVVLVQVA |
| **12** | GSVGADTMDGSMKW |
| **12** | GSVGADTMDGSMKW |
| **12** | RKKNSKEWQRL |
| **12** | GSVGADTMDGSMKW |
| **12** | TTRSGHMIVNDGEF |
| **12** | TDRTDGSYVEQKTA |
| **12** | TTRSGHMIVNDGEF |
| **12** | NDKHGDGSGRRPVW |
| **12** | VLCCGDDRTDEDMF |
| **12** | VLCCGDDRTDEDMF |
| **12** | EENRTLTTRSGHMI |
| **12** | GKWNDVDEGRVKCF |
| **12** | EENRTLTTRSGHMI |
| **12** | RDKVVEVYDGDRTL |
| **12** | CCGDDRTDEDMFTY |
| **12** | VTGSYPRGVRPRGRGA |
| **12** | CCGDDRTDEDMFTY |
| **12** | INDKHGDGSGRRPVW |
| **12** | NSEDEVPRQEREGVA |
| **12** | TDHTEHTTRFDDHL |
| **12** | NDKHGDGSGRRPVWY |
| **12** | LNSEDEVPRQEREGVA |
| **12** | HMVSSATDHTEHTTRF |
| **12** | HMVSSATDHTEHTTRF |
| **12** | DPSVKQFTCTVGVKPSNA |
| **12** | MNVNRMFRDKVVEVY |
| **12** | MNVNRMFRDKVVEVY |
| **12** | MNVNRMFRDKVVEVY |
| **12** | NSEDEVPRQEREGVADRL |
| **12** | VPDDEGSSSTPPMPQRTNGPRTSA |
| **12** | VPDDEGSSSTPPMPQRTNGPRTSA |
| **12** | DLVPDDEGSSSTPPMPQRTNGPRTSA |
| **12** | DLVPDDEGSSSTPPMPQRTNGPRTSA |
| **12** | VPDDEGSSSTPPMPQRTNGPRTSASGQKRRGL |
| **12** | VPDDEGSSSTPPMPQRTNGPRTSASGQKRRGL |
| **13** | SRGPAEA |
| **13** | LSKTGW |
| **13** | PDDGSMA |
| **13** | VMKAGKA |
| **13** | DSAEDVA |
| **13** | PDDGSMA |
| **13** | SYGSRF |
| **13** | AVPPSVF |
| **13** | VPPSVFA |
| **13** | PKQMTI |
| **13** | VVEKPF |
| **13** | VMKAGKA |
| **13** | GKDTASF |
| **13** | GEEERI |
| **13** | SKTINGL |
| **13** | PKQMTI |
| **13** | EDVAGVF |
| **13** | TPEQTY |
| **13** | VRDDEL |
| **13** | TFKEDI |
| **13** | QRDPTY |
| **13** | PDIRVPA |
| **13** | SLDKCVI |
| **13** | QRDPTY |
| **13** | MDPPVRA |
| **13** | VDNPRW |
| **13** | GTEGRGGY |
| **13** | ERPPKVA |
| **13** | RVPANEL |
| **13** | GENFGDY |
| **13** | MDPPVRA |
| **13** | RSEKTDA |
| **13** | RVQPNEA |
| **13** | EELSKTI |
| **13** | TTMFPDI |
| **13** | LPKQMTI |
| **13** | NSMFEPL |
| **13** | TTMFPDI |
| **13** | YRRGQY |
| **13** | LPKQMTI |
| **13** | GVPFVMKA |
| **13** | NSMFEPL |
| **13** | HPPCKSDA |
| **13** | AMDPPVRA |
| **13** | MDPPVRAA |
| **13** | VDNPRWA |
| **13** | TAPDDGSMA |
| **13** | GVPFVMKA |
| **13** | ERPPKVAA |
| **13** | PPTQEEVA |
| **13** | DEFVSKF |
| **13** | AMDPPVRA |
| **13** | MDPPVRAA |
| **13** | ARSEKTDA |
| **13** | TAPDDGSMA |
| **13** | FTPEQTY |
| **13** | TPEQTYF |
| **13** | DCLRGKQA |
| **13** | FVRDDEL |
| **13** | VESVVITF |
| **13** | PDDGSMAGY |
| **13** | GEEERIDA |
| **13** | DKCVIGQY |
| **13** | PDDGSMAGY |
| **13** | GKEMNQNL |
| **13** | PPTQEEVAA |
| **13** | YQRDPTY |
| **13** | GTEGRGGYF |
| **13** | GKEMNQNL |
| **13** | GKKQPTKY |
| **13** | RRGQYDSA |
| **13** | SKTGWNRL |
| **13** | VAERPPKVA |
| **13** | VRDDELRA |
| **13** | EMEERETA |
| **13** | GAPPTQEEVA |
| **13** | DGQEVANRL |
| **13** | EMEERETA |
| **13** | RDVMQNHL |
| **13** | VLRVQPNEA |
| **13** | GSRFDDKMA |
| **13** | RDVMQNHL |
| **13** | DDKMADSPSA |
| **13** | PKQMTIVGY |
| **13** | GSRFDDKMA |
| **13** | LGKEMNQNL |
| **13** | GKEMNQNLL |
| **13** | RPVLVKQHA |
| **13** | RSEKTDAEL |
| **13** | DDKMADSPSA |
| **13** | PKQMTIVGY |
| **13** | LGKEMNQNL |
| **13** | GKEMNQNLL |
| **13** | AEMEERETA |
| **13** | RVQPNEAVY |
| **13** | QRDPTYEW |
| **13** | AEMEERETA |
| **13** | VRDEKVKVL |
| **13** | MKTNVKTPGL |
| **13** | QRDPTYEW |
| **13** | NRLVVEKPF |
| **13** | MKTNVKTPGL |
| **13** | HPPCKSDAKM |
| **13** | IRDVMQNHL |
| **13** | HPPCKSDAKM |
| **13** | IRDVMQNHL |
| **13** | GKKQPTKYKA |
| **13** | EAGKKQPTKY |
| **13** | MKTNVKTPGLA |
| **13** | EWHPPCKSDA |
| **13** | MKTNVKTPGLA |
| **13** | VMYVDNPRW |
| **13** | VVEKPFGKDTA |
| **13** | VMYVDNPRW |
| **13** | NRHYVESVVI |
| **13** | KEDIGTEGRGGY |
| **13** | VRDEKVKVLHA |
| **13** | VKQHAGEEERI |
| **13** | VYMKTNVKTPGL |
| **13** | VYMKTNVKTPGL |
| **13** | DEKKVEVRMKF |
| **13** | GDYVRDEKVKVL |
| **13** | DEKKVEVRMKF |
| **13** | RDVMQNHLTQML |
| **13** | RDVMQNHLTQML |
| **13** | RDVMQNHLTQML |
| **13** | LDEKKVEVRMKF |
| **13** | LDEKKVEVRMKF |
| **13** | EMEERETADGQEVA |
| **13** | EMEERETADGQEVA |
| **13** | TEDDTVPDDSVTPTY |
| **13** | TEDDTVPDDSVTPTYA |
| **13** | DEKKVEVRMKFHTPA |
| **13** | DEKKVEVRMKFHTPA |
| **13** | GYTEDDTVPDDSVTPTY |
| **14** | EQAGGQA |
| **14** | RRSSSA |
| **14** | SINEGY |
| **14** | GPPRGGAA |
| **14** | STGSKPI |
| **14** | VVFDPL |
| **14** | QPARQL |
| **14** | QPARQL |
| **14** | KEEPTL |
| **14** | GVFRQL |
| **14** | ARSVGSF |
| **14** | SDVLQPA |
| **14** | RRSSSAA |
| **14** | EECEEPI |
| **14** | PCLSDVL |
| **14** | GRPNGRL |
| **14** | RRTHSI |
| **14** | TPGEAPCL |
| **14** | DVQPESL |
| **14** | KEEPTLA |
| **14** | STGSKPIL |
| **14** | DEDIDGF |
| **14** | VLTHPHI |
| **14** | DPSLNEF |
| **14** | RGRRSTL |
| **14** | EECEEPI |
| **14** | EECEEPII |
| **14** | RQLTPGEA |
| **14** | CLRRSSSA |
| **14** | LRRTHSI |
| **14** | HHRVPVF |
| **14** | LDVQPESL |
| **14** | NEGYCSSF |
| **14** | DPPTKDMGA |
| **14** | GSGPKSDPMA |
| **14** | DPPTKDMGA |
| **14** | RRTHSIDA |
| **14** | EECEEPII |
| **14** | GSGPKSDPMA |
| **14** | TRRSRCW |
| **14** | TTGQGSVDGF |
| **14** | HAGRPNGRL |
| **14** | VKEPGTGQY |
| **14** | ADPPTKDMGA |
| **14** | GGQASTGSKPI |
| **14** | HHRVPVFI |
| **14** | GRPNGRLKL |
| **14** | ADPPTKDMGA |
| **14** | DPPTKDMGAI |
| **14** | NTLKEEPTL |
| **14** | VKEPGTGQYA |
| **14** | GSGPKSDPMAI |
| **14** | DPPTKDMGAI |
| **14** | DGDPRSCRY |
| **14** | GSGPKSDPMAI |
| **14** | THPHIQVPF |
| **14** | IVKEPGTGQY |
| **14** | GGRSPTRGVTL |
| **14** | ADGDPRSCRY |
| **14** | RSPRVRRRA |
| **14** | VLTTGQGSVDGF |
| **14** | DGDPRSCRYI |
| **14** | TTGQGSVDGFTL |
| **14** | GSMVPEVHRTL |
| **14** | PWGSGPKSDPMA |
| **14** | GSMVPEVHRTL |
| **14** | PWGSGPKSDPMA |
| **14** | PWTRRSRCW |
| **14** | LRSPRVRRRA |
| **14** | GSMVPEVHRTLL |
| **14** | IGSMVPEVHRTL |
| **14** | GSMVPEVHRTLL |
| **14** | IGSMVPEVHRTL |
| **14** | GSEDDVSMCCTHF |
| **14** | RSPRVRRRAPW |
| **14** | RGRRSTLGPPRGGA |
| **14** | GSEDDVSMCCTHF |
| **14** | IGSEDDVSMCCTHF |
| **14** | IGSEDDVSMCCTHF |
| **14** | GGRSPTRGVTLDEDI |
| **14** | DVQPESLHHRVPVF |
| **14** | RSVGSFGGRSPTRGVTL |
| **14** | GSEDDVSMCCTHFRTA |
| **14** | GSEDDVSMCCTHFRTA |
| **14** | TRRSRCWRGRRSTL |
| **15** | MTTIHA |
| **15** | QLSPTF |
| **15** | MTTIHA |
| **15** | QLSPTF |
| **15** | NDKFGI |
| **15** | DEIKKA |
| **15** | PFPGTVEA |
| **15** | KAVGKVI |
| **15** | VVEGKAI |
| **15** | TKVVISA |
| **15** | KNSTNY |
| **15** | VKLVSW |
| **15** | SCTTNCL |
| **15** | CKDPATI |
| **15** | SAHMKGGA |
| **15** | DNEYGY |
| **15** | PELNGKL |
| **15** | KKAVQEA |
| **15** | SAHMKGGA |
| **15** | PWKDDGA |
| **15** | NGKLTGMA |
| **15** | SPTFVKL |
| **15** | RDGRGAY |
| **15** | KDDGAEY |
| **15** | GYSCRVI |
| **15** | SCRVIDL |
| **15** | NGKLTGMA |
| **15** | TTTEKASA |
| **15** | SCTTNCLA |
| **15** | WRDGRGA |
| **15** | PFPGTVEA |
| **15** | KGDARSSI |
| **15** | VVESTGVF |
| **15** | SETSMKGI |
| **15** | VGKVIPEL |
| **15** | KVINDKF |
| **15** | SETSMKGI |
| **15** | EEGLMTTI |
| **15** | EEGLMTTI |
| **15** | TVHACKDPA |
| **15** | SETSMKGIL |
| **15** | SETSMKGIL |
| **15** | NHMNKTDTA |
| **15** | NHMNKTDTA |
| **15** | QKSMNVVSNA |
| **15** | QKSMNVVSNA |
| **15** | QKSMNVVSNA |
| **15** | KNSTNYDEI |
| **15** | QKSMNVVSNA |
| **15** | VGVGNNEDKY |
| **15** | PGTVEAKDGKL |
| **15** | EYVVESTGVF |
| **15** | HMKGGATKVVI |
| **15** | KDGKLVVEGKA |
| **15** | LNHMNKTDTA |
| **15** | HMKGGATKVVI |
| **15** | LNHMNKTDTA |
| **15** | TEDQVVSQDF |
| **15** | TVRLKNSTNY |
| **15** | TQKTVDGPSGKL |
| **15** | VQEASETSMKGI |
| **15** | RVPVPDVSVVDL |
| **15** | VQEASETSMKGI |
| **15** | GYTEDQVVSQDF |
| **15** | TQKTVDGPSGKLW |
| **15** | FRVPVPDVSVVDL |
| **15** | VVESTGVFTTTEKA |
| **15** | PMFVGVGNNEDKY |
| **15** | PMFVGVGNNEDKY |
| **15** | VTATQKTVDGPSGKL |
| **15** | TEDQVVSQDFKGDA |
| **15** | RVPVPDVSVVDLTVRL |
| **15** | QKSMNVVSNASCTTNCL |
| **15** | QKSMNVVSNASCTTNCL |
| **15** | QKSMNVVSNASCTTNCL |
| **15** | QKSMNVVSNASCTTNCL |
| **15** | VGVGNNEDKYQKSMNVVSNA |
| **15** | VGVGNNEDKYQKSMNVVSNA |
| **16** | LGRTRL |
| **16** | VRECVL |
| **16** | GSLRGVPA |
| **16** | GRTRLPA |
| **16** | RTPIGSF |
| **16** | STLHDVY |
| **16** | RGVPAVEL |
| **16** | DAVRECVL |
| **16** | VRECVLGNVL |
| **16** | TVCTTVNKVCA |
| **16** | ATVCTTVNKVCA |
| **16** | TVCTTVNKVCASGL |
| **17** | AVCPVPA |
| **17** | QKTKTA |
| **17** | VETIEA |
| **17** | QKTKTA |
| **17** | TWSMSA |
| **17** | PPTPYL |
| **17** | TWSMSA |
| **17** | EVIESF |
| **17** | FTGETVA |
| **17** | KVMFCI |
| **17** | QETRIL |
| **17** | AQKTKTA |
| **17** | KVMFCI |
| **17** | QETRIL |
| **17** | PDVDGFL |
| **17** | QKTKTAL |
| **17** | RETDDF |
| **17** | QKTKTAL |
| **17** | TSLCKEL |
| **17** | KDVGCTW |
| **17** | VCPVPAGPA |
| **17** | ESFKSDL |
| **17** | DHTRSVL |
| **17** | DVCVRQL |
| **17** | RQDFEVA |
| **17** | QLPDVDGF |
| **17** | KCNLSKDA |
| **17** | QLPDVDGF |
| **17** | RETDDFI |
| **17** | KDVNEKVA |
| **17** | SHGLKVMF |
| **17** | GDWKDVVI |
| **17** | SHGLKVMF |
| **17** | LDHTRSVL |
| **17** | CTCSCPSPF |
| **17** | TGETVAEMI |
| **17** | TGETVAEMI |
| **17** | GHSERRNL |
| **17** | AKDVNEKVA |
| **17** | SPTPPRPSHI |
| **17** | VGGNWKCNL |
| **17** | ACTCSCPSPF |
| **17** | KDVGCTWVI |
| **17** | ETDKVEVVL |
| **17** | DVCVRQLHA |
| **17** | LGHSERRNL |
| **17** | SPTPPRPSHI |
| **17** | SPTPPRPSHIL |
| **17** | QEREEGRTL |
| **17** | ETDKVEVVLA |
| **17** | QEREEGRTL |
| **17** | PQIRETDDF |
| **17** | GGSVSPGNCNEL |
| **17** | EMIKDVGCTW |
| **17** | GHDERSRCRA |
| **17** | EMIKDVGCTW |
| **17** | SPTPPRPSHIL |
| **17** | GGSVSPGNCNELA |
| **17** | PPRSPSPVRKL |
| **17** | YGGSVSPGNCNEL |
| **17** | GHDERSRCRAI |
| **17** | GHSERRNLPQI |
| **17** | PDLETDKVEVVL |
| **17** | DHTRSVLRQDF |
| **17** | TPDQVEEVHEKI |
| **17** | PPRSPSPVRKLPL |
| **17** | GETLQEREEGRTL |
| **17** | KDVNEKVAQETRI |
| **17** | SCEIGHDERSRCRA |
| **17** | TPDQVEEVHEKIRHY |
| **17** | QEREEGRTLDVCVRQL |
| **17** | QEREEGRTLDVCVRQL |
| **17** | GTGKVATPDQVEEVHEKI |
| **17** | CTCSCPSPFPPRSPSPVRKL |
| **18** | NHRVGA |
| **18** | SPVADW |
| **18** | GGGSRQL |
| **18** | LEGKSF |
| **18** | GGAGGTVY |
| **18** | GEEVDF |
| **18** | VKHMDA |
| **18** | VATTPTL |
| **18** | DQGNTTA |
| **18** | VKHMDA |
| **18** | GVYDTY |
| **18** | LRRGGY |
| **18** | DKYTSL |
| **18** | FRTVGF |
| **18** | EGNGVLF |
| **18** | VRRELA |
| **18** | GGITRNGA |
| **18** | GADTDTDA |
| **18** | DRGSFDA |
| **18** | DVGYGVY |
| **18** | RRGGYPA |
| **18** | ADQGNTTA |
| **18** | DMTRIF |
| **18** | GGGSRQLI |
| **18** | RRMRVA |
| **18** | YDMTRI |
| **18** | DMTRIF |
| **18** | TQQVITL |
| **18** | ENNVQSL |
| **18** | RRMRVA |
| **18** | VDNHDTL |
| **18** | YDMTRI |
| **18** | TRAGRGKA |
| **18** | RRDHAY |
| **18** | DGTEEVTA |
| **18** | FGGGSRQL |
| **18** | RTVGFDGA |
| **18** | HMHEASL |
| **18** | GHVKGEVT |
| **18** | PGRRGKY |
| **18** | PSHRQVI |
| **18** | HMHEASL |
| **18** | GEEVDFF |
| **18** | VVLSSGPGGA |
| **18** | PPPYKGDA |
| **18** | YGEEVDF |
| **18** | RRMRVAA |
| **18** | QVAEGNGVL |
| **18** | MDVPLHY |
| **18** | RRMRVAA |
| **18** | DAVKHMDA |
| **18** | QVAEGNGVL |
| **18** | HDAGVQVY |
| **18** | MDVPLHY |
| **18** | NNTAGSMSL |
| **18** | EWDRGSF |
| **18** | DAVKHMDA |
| **18** | NNTAGSMSL |
| **18** | ERGNYDY |
| **18** | RLRRDHA |
| **18** | HYHMHEA |
| **18** | VDNHDTLL |
| **18** | DQGNTTARA |
| **18** | TSLERGNY |
| **18** | LGHVKGEVT |
| **18** | HYHMHEA |
| **18** | VREVRPDA |
| **18** | DTDTDAEW |
| **18** | EGKSFDKY |
| **18** | EWVRREL |
| **18** | WENNVQSL |
| **18** | PGRRGKYSA |
| **18** | ENNVQSLQA |
| **18** | VKHMDARF |
| **18** | VKHMDARF |
| **18** | GSMSLMDVPL |
| **18** | TLPSHRQVI |
| **18** | GSMSLMDVPL |
| **18** | DVVLNHRVGA |
| **18** | GSMSLMDVPL |
| **18** | TFPGRRGKY |
| **18** | TRNGATQQVI |
| **18** | VREVRPDAF |
| **18** | PAVREVRPDA |
| **18** | VTFVDNHDTL |
| **18** | PSHRQVIDVL |
| **18** | DQKGSVRTKY |
| **18** | DGTEEVTATPY |
| **18** | SSGPGGARRMRVA |
| **18** | SSGPGGARRMRVA |
| **18** | NHRVGADGTEEVTA |
| **18** | NDRSKPSGPPRRI |
| **18** | GEFDQKGSVRTKY |
| **18** | DQKGSVRTKYGTKA |
| **18** | ANDRSKPSGPPRRI |
| **18** | NDRSKPSGPPRRIRA |
| **19** | GVKLMI |
| **19** | PNTATY |
| **19** | VCVSDF |
| **19** | GVKLMI |
| **19** | NGCIGTI |
| **19** | GYDQKA |
| **19** | YGDSKL |
| **19** | KPDHEA |
| **19** | TKVSDSA |
| **19** | TVNNNF |
| **19** | KNSGGVF |
| **19** | ANKVPDA |
| **19** | TRTDSF |
| **19** | LGTPVKL |
| **19** | HDFDMA |
| **19** | FCEKPI |
| **19** | DQKAEF |
| **19** | NNADVHA |
| **19** | PPPVGYL |
| **19** | HDFDMA |
| **19** | SEIDPSL |
| **19** | SVIDESL |
| **19** | QKRYDA |
| **19** | KVHVGII |
| **19** | DKTLSVI |
| **19** | QKRYDA |
| **19** | GDYDNTI |
| **19** | GFQKRY |
| **19** | DNTICHL |
| **19** | VCVSDFF |
| **19** | VGSDVVEI |
| **19** | TKVSDSAL |
| **19** | ITVNNNF |
| **19** | KNSGGVFL |
| **19** | LKNSGGVF |
| **19** | GTIDNSRA |
| **19** | VEEAGVKL |
| **19** | CTDYKDL |
| **19** | MYKPDHEA |
| **19** | EESARRL |
| **19** | GTPVKLHL |
| **19** | EDSVSEEA |
| **19** | DQTIHDF |
| **19** | MYKPDHEA |
| **19** | NKVPDAEL |
| **19** | HLTSRDPA |
| **19** | MYKPDHEA |
| **19** | MYKPDHEA |
| **19** | ELVCVSDF |
| **19** | DNSRATPY |
| **19** | VCSPTDTHA |
| **19** | LVGSDVVEI |
| **19** | KRPDKSTL |
| **19** | KQYNVPMA |
| **19** | KQYNVPMA |
| **19** | VGSDVVEIY |
| **19** | MYKPDHEA |
| **19** | KPDHEAGKL |
| **19** | GCGRIGQCHA |
| **19** | MYKPDHEA |
| **19** | KENRPVRI |
| **19** | NVPMACTDY |
| **19** | DRTGCHTDL |
| **19** | HKVMVQQSA |
| **19** | NVPMACTDY |
| **19** | IVCSPTDTHA |
| **19** | HKVMVQQSA |
| **19** | CEKPIDKTL |
| **19** | ESFTKVSDSA |
| **19** | ADRTGCHTDL |
| **19** | TVNNNFPNTA |
| **19** | RLEDSVSEEA |
| **19** | VCSPTDTHADI |
| **19** | TRTDSFDRNL |
| **19** | GKLKRPDKSTL |
| **19** | TSRDPAPPPVGY |
| **19** | KENRPVRISEI |
| **19** | DRTGCHTDLPVY |
| **19** | HKVMVQQSAESF |
| **19** | HKVMVQQSAESF |
| **19** | TKSIKENRPVRI |
| **19** | EDSVSEEAKVHVGI |
| **19** | GDSKLHKVMVQQSA |
| **19** | GDSKLHKVMVQQSA |
| **19** | VQDTPVPCTGNDGRI |
| **19** | KRPDKSTLTRTDSF |
| **19** | DCIVQDTPVPCTGNDGRI |
| **19** | VQDTPVPCTGNDGRIPVVY |
| **20** | RSPDAI |
| **20** | RMTDGA |
| **20** | KHVIGL |
| **20** | LDSSRL |
| **20** | DFTHGGA |
| **20** | TNWGVGA |
| **20** | SMANVGGA |
| **20** | HARVVGA |
| **20** | VARSPDA |
| **20** | NVGGARF |
| **20** | SMANVGGA |
| **20** | VDELCF |
| **20** | GSLVDEL |
| **20** | VSNPVDI |
| **20** | RGAKVTI |
| **20** | EEDVFL |
| **20** | KGYTNW |
| **20** | RVPLSEA |
| **20** | VGCGSVGMA |
| **20** | VGCGSVGMA |
| **20** | GIEEDVF |
| **20** | EHTKLHA |
| **20** | RMTDGAW |
| **20** | PKSVHASI |
| **20** | PNYDGSEA |
| **20** | RMTDGAW |
| **20** | IVSNPVDI |
| **20** | VSNPVDIL |
| **20** | GEHGDSSVA |
| **20** | EVARSVEA |
| **20** | GVAPKSVHA |
| **20** | DIRMTDGA |
| **20** | DGSEASDVI |
| **20** | DIRMTDGA |
| **20** | KKVEGEVL |
| **20** | DSSRLRVA |
| **20** | SEAEHTKL |
| **20** | GGGNDSGGSSGGI |
| **20** | GRGGVERVL |
| **20** | GRVFGSGTY |
| **20** | VGCGSVGMACA |
| **20** | VGCGSVGMACA |
| **20** | LGEHGDSSVA |
| **20** | GGGNDSGGSSGGIA |
| **20** | GGGNDSGGSSGGI |
| **20** | GEHGDSSVAVA |
| **20** | GGGNDSGGSSGGIA |
| **20** | RQRPGESRL |
| **20** | VLGRGGVERVL |
| **20** | KKVEGEVLDF |
| **20** | RQRPGESRLA |
| **20** | PSVGGGGDRGVGSL |
| **20** | KVTIVGCGSVGMA |
| **20** | GARQRPGESRL |
| **20** | KVTIVGCGSVGMA |
| **20** | DVDAKKVEGEVL |
| **20** | RDEKRVVPVSVA |
| **20** | GRGGVERVLRVPL |
| **20** | RDEKRVVPVSVAA |
| **20** | CFPSVGGGGDRGVGSL |
| **20** | LRDEKRVVPVSVA |
| **20** | PSVGGGGDRGVGSLRGA |
| **21** | CQSMGL |
| **21** | DPKVEA |
| **21** | DKVVKA |
| **21** | QRVSSI |
| **21** | DGRNVL |
| **21** | RDADVI |
| **21** | QRVSSI |
| **21** | CRGKNL |
| **21** | PDVAEY |
| **21** | SNPEFL |
| **21** | VTLCDSA |
| **21** | DEFKGL |
| **21** | LTTNVW |
| **21** | LCQSMGL |
| **21** | EKYVTL |
| **21** | VSRERI |
| **21** | AQRVSSI |
| **21** | WNSDEL |
| **21** | SSELSKL |
| **21** | LCQSMGL |
| **21** | VGMDSRI |
| **21** | NSDELPI |
| **21** | EICRRL |
| **21** | VGMDSRI |
| **21** | YDPKVEA |
| **21** | TEWDEF |
| **21** | DNPDRVL |
| **21** | STDVDQGI |
| **21** | EGTAMTDL |
| **21** | CRGKNLF |
| **21** | GYVGGPTMA |
| **21** | EGTAMTDL |
| **21** | QRVSSINA |
| **21** | SVGFGGSCF |
| **21** | SGADVDEVA |
| **21** | GKPLDPKL |
| **21** | GYVGGPTMA |
| **21** | VSRERIL |
| **21** | HGAGKTRF |
| **21** | DVDEVARA |
| **21** | QRVSSINA |
| **21** | NTVTGKRI |
| **21** | VDISVPRI |
| **21** | EERASVCI |
| **21** | MVSNPVQTGL |
| **21** | VFDGRNVL |
| **21** | DVLSNPEF |
| **21** | GKTRFDVL |
| **21** | MVSNPVQTGL |
| **21** | DNPDRVLI |
| **21** | DPKLNTNF |
| **21** | GGSCFQKDI |
| **21** | MVSNPVQTGL |
| **21** | MVSNPVQTGL |
| **21** | NTVTGKRIA |
| **21** | GGNMNESGQA |
| **21** | VGGPTMAMMA |
| **21** | GGNMNESGQA |
| **21** | VGGPTMAMMA |
| **21** | FSTDVDQGI |
| **21** | KCPEVTVTI |
| **21** | DGRNVLDHA |
| **21** | VGGPTMAMMA |
| **21** | TRLDRTEL |
| **21** | RAVGMDSRI |
| **21** | QDGMVKPCF |
| **21** | VGGPTMAMMA |
| **21** | DPKVEANQI |
| **21** | CQSMGLPDVA |
| **21** | RAVGMDSRI |
| **21** | QDGMVKPCF |
| **21** | QDGMVKPCF |
| **21** | GGNMNESGQAA |
| **21** | FNTVTGKRI |
| **21** | CQSMGLPDVA |
| **21** | TTNVWSSEL |
| **21** | QDGMVKPCF |
| **21** | MVSNPVQTGL |
| **21** | GGNMNESGQAA |
| **21** | DRTELEKY |
| **21** | EPGLDKVVKA |
| **21** | VNTPTKKVGL |
| **21** | MVSNPVQTGL |
| **21** | IGGNMNESGQA |
| **21** | HWVSRERI |
| **21** | IGGNMNESGQA |
| **21** | LKCPEVTVTI |
| **21** | STDVDQGIRDA |
| **21** | MVSNPVQTGLRI |
| **21** | VNTPTKKVGLGA |
| **21** | VGMDSRIGPKF |
| **21** | KKDTGDTRESA |
| **21** | MVSNPVQTGLRI |
| **21** | VGMDSRIGPKF |
| **21** | MVSNPVQTGLRI |
| **21** | MVSNPVQTGLRI |
| **21** | VAVNTPTKKVGL |
| **21** | QDGMVKPCFVF |
| **21** | KIQDGMVKPCF |
| **21** | QDGMVKPCFVF |
| **21** | QDGMVKPCFVF |
| **21** | KKDTGDTRESAA |
| **21** | KIQDGMVKPCF |
| **21** | QDGMVKPCFVF |
| **21** | MTDLDNPDRVL |
| **21** | MTDLDNPDRVL |
| **21** | MVSNPVQTGLRI |
| **21** | KCPEVTVTIVDI |
| **21** | MVSNPVQTGLRI |
| **21** | DKVVKACRGKNL |
| **21** | FKKDTGDTRESA |
| **21** | ESPKVVVEKSTVPI |
| **21** | HSVVTMNDRQKERF |
| **21** | HSVVTMNDRQKERF |
| **21** | SVAESPKVVVEKSTVPI |
| **21** | ESPKVVVEKSTVPIKTA |
| **21** | FHSVVTMNDRQKERF |
| **21** | FHSVVTMNDRQKERF |
| **21** | HSVVTMNDRQKERFVSRI |
| **21** | HSVVTMNDRQKERFVSRI |
| **22** | VVEGLF |
| **22** | LQGDQL |
| **22** | TVRGTAA |
| **22** | TSKVPF |
| **22** | RLPQPA |
| **22** | DPTVAW |
| **22** | TRAEVL |
| **22** | GPRLTF |
| **22** | DVVAKY |
| **22** | CVVPPSL |
| **22** | QQVRMA |
| **22** | LTVRGTA |
| **22** | ENHCCL |
| **22** | RLTESL |
| **22** | QQVRMA |
| **22** | QQVRMA |
| **22** | PQFVTF |
| **22** | NHGRFL |
| **22** | QQVRMA |
| **22** | KAKKGGVA |
| **22** | DPAPVTPA |
| **22** | DGDRSGGL |
| **22** | SSSHNTY |
| **22** | PQPADVDA |
| **22** | KHRARF |
| **22** | VTLGGGSRA |
| **22** | CVVPPSLL |
| **22** | ENHCCLL |
| **22** | PVTPADDGA |
| **22** | RVRVDKA |
| **22** | LQQVRMA |
| **22** | DHSAEEVA |
| **22** | LQQVRMA |
| **22** | CGYVPRPA |
| **22** | VKYGDDPA |
| **22** | RRLHVNA |
| **22** | VSSDDDSF |
| **22** | RDVIDVVA |
| **22** | DMYRVVL |
| **22** | NWQEHDA |
| **22** | RNVTRVY |
| **22** | SSSHNTYL |
| **22** | ISSSHNTY |
| **22** | DMYRVVL |
| **22** | EACVVPPSL |
| **22** | HGCRCVEI |
| **22** | SLENHCCL |
| **22** | SRVNSSNY |
| **22** | HVNAVVEGL |
| **22** | AVSSDDDSF |
| **22** | QSDSSADMY |
| **22** | ASRVNSSNY |
| **22** | QSDSSADMY |
| **22** | QSDSSADMY |
| **22** | RADGDRSGGL |
| **22** | QEHDAGMQL |
| **22** | QSDSSADMY |
| **22** | QEHDAGMQL |
| **22** | QEHDAGMQL |
| **22** | LHGCRCVEI |
| **22** | GSKGKKKVKA |
| **22** | GSGGSGGSGGGGSSA |
| **22** | QEHDAGMQL |
| **22** | GMQLNHGRF |
| **22** | QQVRMATHL |
| **22** | RVRVDKADI |
| **22** | GMQLNHGRF |
| **22** | RNVTRVYPA |
| **22** | DGDEGEPVVY |
| **22** | QQVRMATHL |
| **22** | QQVRMATHL |
| **22** | NARNVTRVY |
| **22** | QQVRMATHL |
| **22** | AGSKGKKKVKA |
| **22** | DGDRSGGLTRA |
| **22** | NETKVESMVA |
| **22** | GSGGSGGSGGGGSSAA |
| **22** | QGDQLQSDSSA |
| **22** | NETKVESMVA |
| **22** | QGDQLQSDSSA |
| **22** | TSKVPFRDVI |
| **22** | SAGSGGSGGSGGGGSSA |
| **22** | GSKGKKKVKAVA |
| **22** | TVEVSGDDDSDA |
| **22** | HGHTLTSKVPF |
| **22** | TESLRVRVDKA |
| **22** | HGCRCVEIDVW |
| **22** | VSSDDDSFSSSDL |
| **22** | TVEVSGDDDSDASA |
| **22** | SRVNSSNYDPTVA |
| **22** | GPATVEVSGDDDSDA |
| **22** | DVWDGDEGEPVVY |
| **22** | NETKVESMVAKHRA |
| **22** | NETKVESMVAKHRA |
| **22** | DGDEGEPVVYHGHTL |
| **22** | EGGDTHPPGQPVTDVCSF |
| **22** | FEGGDTHPPGQPVTDVCSF |
| **22** | EGGDTHPPGQPVTDVCSFNETKVESMVA |
| **22** | EGGDTHPPGQPVTDVCSFNETKVESMVA |
| **23** | QKNLVA |
| **23** | DKHADA |
| **23** | TPDIGSA |
| **23** | NIGETGA |
| **23** | GEEVTGA |
| **23** | NIMKSA |
| **23** | QKNLVA |
| **23** | VSLNPF |
| **23** | NWSSNA |
| **23** | VDVFEA |
| **23** | NIMKSA |
| **23** | VHKANI |
| **23** | EGNGNKA |
| **23** | NQMQTA |
| **23** | NQMQTA |
| **23** | DVPLEF |
| **23** | MKSADGL |
| **23** | EFEGMI |
| **23** | PTLEVTA |
| **23** | GNIVTNI |
| **23** | MKSADGL |
| **23** | EFEGMI |
| **23** | PGVKSRL |
| **23** | SGMEHEA |
| **23** | GEEVTGAL |
| **23** | SGMEHEA |
| **23** | GIGPGPGPGA |
| **23** | DCCKRVA |
| **23** | SNYEDGL |
| **23** | VPGVVESL |
| **23** | GGKKKLGL |
| **23** | TQLDKHA |
| **23** | GSATTTDY |
| **23** | RRDFDL |
| **23** | GVDVVVMI |
| **23** | GPGPGPGANI |
| **23** | GVDVVVMI |
| **23** | TTTDYTY |
| **23** | KSFQKNL |
| **23** | DCCKRVAA |
| **23** | ESAVMRVL |
| **23** | NQMQTATL |
| **23** | LDCCKRVA |
| **23** | ESAVMRVL |
| **23** | NQMQTATL |
| **23** | TTPVKSKY |
| **23** | NNRKKVTA |
| **23** | PQEVIKSF |
| **23** | SQGLRHGSL |
| **23** | SIPGVKSRL |
| **23** | TEVRSMRI |
| **23** | RENTEGEY |
| **23** | EDGLPQEVI |
| **23** | ANNRKKVTA |
| **23** | TEVRSMRI |
| **23** | GGTLNQMQTA |
| **23** | SPTMQSTRL |
| **23** | GGTLNQMQTA |
| **23** | SPTMQSTRL |
| **23** | VPGVVESLKI |
| **23** | TEVRSMRIA |
| **23** | EGHGHGHGGRA |
| **23** | TEVRSMRIA |
| **23** | ITEVRSMRI |
| **23** | PGEGIGEEVTGA |
| **23** | EGNGNKATPDI |
| **23** | ITEVRSMRI |
| **23** | TLSPTMQSTRL |
| **23** | SPTMQSTRLL |
| **23** | TLSPTMQSTRL |
| **23** | SPTMQSTRLL |
| **23** | FEGHGHGHGGRA |
| **23** | TTPVKSKYTSI |
| **23** | TLSPTMQSTRL |
| **23** | TLSPTMQSTRL |
| **23** | VMRVLEGNGNKA |
| **23** | VMRVLEGNGNKA |
| **23** | KGPFTTPVKSKY |
| **23** | NNRKKVTAVHKA |
| **23** | SGMEHEAVPGVVESL |
| **23** | SGMEHEAVPGVVESL |
| **23** | PGVKSRLGVDVVVMI |
| **23** | PGVKSRLGVDVVVMI |
| **23** | EGHGHGHGGRAGGKKKL |
| **23** | RENTEGEYSGMEHEA |
| **23** | RENTEGEYSGMEHEA |
| **23** | VDNTCMQMVSRPQQF |
| **23** | VDNTCMQMVSRPQQF |
| **23** | GVDVVVMIRENTEGEY |
| **23** | VDNTCMQMVSRPQQF |
| **23** | GVDVVVMIRENTEGEY |
| **23** | EGMIVDNTCMQMVSRPQQF |
| **23** | EGMIVDNTCMQMVSRPQQF |
| **23** | VDNTCMQMVSRPQQFDVMI |
| **23** | EGMIVDNTCMQMVSRPQQF |
| **23** | VDNTCMQMVSRPQQFDVMI |
| **23** | EGMIVDNTCMQMVSRPQQF |
| **23** | VDNTCMQMVSRPQQFDVMI |
| **23** | VDNTCMQMVSRPQQFDVMI |

Number=Table4 Number

S3-3 Potential bioactive peptides of the Amino acid metabolism-Trypsin.

| **Number** | **Sequence** |
| --- | --- |
| **1** | TPTTRR |
| **1** | WGDGWR |
| **1** | QCTAANFR |
| **1** | QCTAANFR |
| **1** | QPTPHCVTR |
| **1** | QPTPHCVTR |
| **1** | VWWLRQR |
| **1** | QCTAANFRTR |
| **1** | QCTAANFRTR |
| **1** | QNIESLSADQK |
| **1** | QNIESLSADQK |
| **1** | PAAAYRWGDGWR |
| **1** | LFVLELEQALR |
| **1** | QNIESLSADQKR |
| **1** | QNIESLSADQKR |
| **1** | PAAAYRWGDGWR |
| **1** | TRQNIESLSADQK |
| **1** | WGDGWRQCTAANFR |
| **1** | VSDTFTLPCVGYASAR |
| **1** | RVSDTFTLPCVGYASAR |
| **1** | VNGQAAHGGVQFLPWHR |
| **1** | VSDTFTLPCVGYASARTPTTR |
| **1** | LAAAFNALISQGLYDQFTTIHR |
| **1** | RLAAAFNALISQGLYDQFTTIHR |
| **1** | ASGNPSQYDGTHQGRPVSPTDSLSAFDGR |
| **1** | ASGNPSQYDGTHQGRPVSPTDSLSAFDGRR |
| **1** | VNGQAAHGGVQFLPWHRLFVLELEQALR |
| **1** | QRASGNPSQYDGTHQGRPVSPTDSLSAFDGR |
| **1** | QRASGNPSQYDGTHQGRPVSPTDSLSAFDGR |
| **2** | AARVTSSR |
| **2** | GLISDIK |
| **2** | DPALLSR |
| **2** | AFVTAIR |
| **2** | ADEVAALK |
| **2** | AARVTSSR |
| **2** | ESLGEMTV |
| **2** | ESLGEMTV |
| **2** | AFLAKGSVK |
| **2** | KADEVAALK |
| **2** | GLIKAFLAK |
| **2** | SKAFVTAIR |
| **2** | YLFALPVSR |
| **2** | TGELLAETHR |
| **2** | SPSKGLISDIK |
| **2** | GQFPGPSVVLR |
| **2** | YGPSTTAAFFGK |
| **2** | TFEVSLETAER |
| **2** | KGQFPGPSVVLR |
| **2** | GQFPGPSVVLRK |
| **2** | GEGAKESLGEMTV |
| **2** | GEGAKESLGEMTV |
| **2** | KPPGCLAVIGEIK |
| **2** | KPPGCLAVIGEIKR |
| **2** | GLISDIKDPALLSR |
| **2** | TDGHSYSSGYNNAK |
| **2** | TFEVSLETAERLR |
| **2** | DMGYSAVLVGEALVK |
| **2** | DMGYSAVLVGEALVK |
| **2** | GSVKYGPSTTAAFFGK |
| **2** | DLRTFEVSLETAER |
| **2** | YGPSTTAAFFGKGEGAK |
| **2** | VRDMGYSAVLVGEALVK |
| **2** | VRDMGYSAVLVGEALVK |
| **2** | STPIPPGMAAFVPGAAAALR |
| **2** | STPIPPGMAAFVPGAAAALR |
| **2** | TDGHSYSSGYNNAKGLIK |
| **2** | STPIPPGMAAFVPGAAAALRR |
| **2** | AEVAAAGDDHPIAALLAAGAPR |
| **2** | STPIPPGMAAFVPGAAAALRR |
| **2** | AFVTAIRKPPGCLAVIGEIK |
| **2** | AADNSKTDGHSYSSGYNNAK |
| **2** | DMGYSAVLVGEALVKAADNSK |
| **2** | DMGYSAVLVGEALVKAADNSK |
| **2** | GAFLGTPALAAAPPPAQASVAANR |
| **2** | AEVAAAGDDHPIAALLAAGAPRSK |
| **2** | RGAFLGTPALAAAPPPAQASVAANR |
| **2** | GAFLGTPALAAAPPPAQASVAANRR |
| **2** | AGIPEGVIAVAESGIEECLDAWR |
| **2** | AGIPEGVIAVAESGIEECLDAWRVR |
| **2** | LRAGIPEGVIAVAESGIEECLDAWR |
| **2** | SAPLGATFPPPPSQPASLLPLLHLCR |
| **2** | ADEVAALKAEVAAAGDDHPIAALLAAGAPR |
| **2** | YLFALPVSRSTPIPPGMAAFVPGAAAALR |
| **2** | YLFALPVSRSTPIPPGMAAFVPGAAAALR |
| **2** | HYNEAGAAAISVLTDGPGFGGCMADLEAVVK |
| **2** | HYNEAGAAAISVLTDGPGFGGCMADLEAVVK |
| **2** | EFIIDPVQIAEAAVAGAHAVLLIVAALGEAR |
| **2** | LGLDAIVEVHDEPELAAAVAVGAEIIGVNNR |
| **2** | VTSSRSAPLGATFPPPPSQPASLLPLLHLCR |
| **2** | KEFIIDPVQIAEAAVAGAHAVLLIVAALGEAR |
| **2** | LGLDAIVEVHDEPELAAAVAVGAEIIGVNNRDLR |
| **2** | AAALTMDADGSDTEAAAPQYAVGDIPDVLVSILER |
| **2** | HYNEAGAAAISVLTDGPGFGGCMADLEAVVKEQAR |
| **2** | AAALTMDADGSDTEAAAPQYAVGDIPDVLVSILER |
| **2** | HYNEAGAAAISVLTDGPGFGGCMADLEAVVKEQAR |
| **2** | AAALTMDADGSDTEAAAPQYAVGDIPDVLVSILERK |
| **2** | AAALTMDADGSDTEAAAPQYAVGDIPDVLVSILERK |
| **2** | RAAALTMDADGSDTEAAAPQYAVGDIPDVLVSILER |
| **2** | RAAALTMDADGSDTEAAAPQYAVGDIPDVLVSILER |
| **2** | SAPLGATFPPPPSQPASLLPLLHLCRYLFALPVSR |
| **2** | DPALLSRHYNEAGAAAISVLTDGPGFGGCMADLEAVVK |
| **2** | DPALLSRHYNEAGAAAISVLTDGPGFGGCMADLEAVVK |
| **3** | TPALCR |
| **3** | TPALCRR |
| **3** | HPSVRR |
| **3** | TPALCRR |
| **3** | DGWFTER |
| **3** | YYSPAMHR |
| **3** | AANAAVVAPSR |
| **3** | YYSPAMHR |
| **3** | EVLKHPSVR |
| **3** | TPPPAVQAGLR |
| **3** | AAFVLPEFAR |
| **3** | GHLLGCSLLLAR |
| **3** | AAFVLPEFARR |
| **3** | DLIKDGWFTER |
| **3** | FLPALAAGFDDPR |
| **3** | GHLLGCSLLLARR |
| **3** | AVLVEIDAAVVEVSK |
| **3** | RFLPALAAGFDDPR |
| **3** | RAVLVEIDAAVVEVSK |
| **3** | AVLVEIDAAVVEVSKR |
| **3** | FSAAPASLAVAIMGLCGK |
| **3** | FSAAPASLAVAIMGLCGK |
| **3** | RFSAAPASLAVAIMGLCGK |
| **3** | RFSAAPASLAVAIMGLCGK |
| **3** | AVEGGGAAEGDAPVAAAAAGGDSE |
| **3** | TPPPAVQAGLRYYSPAMHR |
| **3** | AANAAVVAPSRTPPPAVQAGLR |
| **3** | YYSPAMHRAAFVLPEFAR |
| **3** | TPPPAVQAGLRYYSPAMHR |
| **3** | YYSPAMHRAAFVLPEFAR |
| **3** | RAVEGGGAAEGDAPVAAAAAGGDSE |
| **3** | FSAAPASLAVAIMGLCGKDLIK |
| **3** | FSAAPASLAVAIMGLCGKDLIK |
| **3** | GALWPGQAMSLEVTEVLYSGR |
| **3** | GALWPGQAMSLEVTEVLYSGR |
| **3** | VFPTVAYAYTAVPTYPSGQIGFVVCAK |
| **3** | RVFPTVAYAYTAVPTYPSGQIGFVVCAK |
| **3** | DGWFTERGALWPGQAMSLEVTEVLYSGR |
| **3** | DGWFTERGALWPGQAMSLEVTEVLYSGR |
| **3** | SDFQDLLVFTNPAYGTVLVLDGVIQVTTR |
| **3** | DEFAYQEMIAHLPLYAHPAPVDVLVIGGGDGGVLR |
| **3** | DEFAYQEMIAHLPLYAHPAPVDVLVIGGGDGGVLR |
| **4** | LIIPLR |
| **4** | DLAHMR |
| **4** | DLAHMR |
| **4** | EHLSAAR |
| **4** | ALGMGYAK |
| **4** | ALGMGYAK |
| **4** | MTFVVEK |
| **4** | QLADGITR |
| **4** | ASHLWVR |
| **4** | MTFVVEK |
| **4** | QLADGITR |
| **4** | TSYFSLTG |
| **4** | EVAIQVVK |
| **4** | HMSSEALK |
| **4** | LIIPLRGK |
| **4** | HMSSEALK |
| **4** | GAPPRGHEK |
| **4** | DKDLAHMR |
| **4** | DKDLAHMR |
| **4** | RMTFVVEK |
| **4** | QLADGITRR |
| **4** | KPFNKTGTK |
| **4** | RMTFVVEK |
| **4** | QLADGITRR |
| **4** | RHMSSEALK |
| **4** | HMSSEALKR |
| **4** | GKEVAIQVVK |
| **4** | RHMSSEALK |
| **4** | HMSSEALKR |
| **4** | EHLSAARAAGK |
| **4** | TGTKLIIPLR |
| **4** | PAAAAAAVATASTPR |
| **4** | PAAAAAAVATASTPR |
| **4** | MTFVVEKGAPPR |
| **4** | MTFVVEKGAPPR |
| **4** | PAAAAAAVATASTPRR |
| **4** | SPVVKTSYFSLTG |
| **4** | AEGGGFLGDTVIMK |
| **4** | EVAIQVVKSPVVK |
| **4** | AEGGGFLGDTVIMK |
| **4** | ALGMGYAKKPFNK |
| **4** | ALGMGYAKKPFNK |
| **4** | TVLHDFHESHGGK |
| **4** | PAAAAAAVATASTPRR |
| **4** | DLAHMREHLSAAR |
| **4** | DLAHMREHLSAAR |
| **4** | RAEGGGFLGDTVIMK |
| **4** | RAEGGGFLGDTVIMK |
| **4** | SSAGLFDVSHMGQVR |
| **4** | SSAGLFDVSHMGQVR |
| **4** | RTVLHDFHESHGGK |
| **4** | SSAGLFDVSHMGQVRLR |
| **4** | SSAGLFDVSHMGQVRLR |
| **4** | DVDLEVLDESALLALQGPK |
| **4** | MVPFAGYSMPVQYSSEGIK |
| **4** | MVPFAGYSMPVQYSSEGIK |
| **4** | MVPFAGYSMPVQYSSEGIK |
| **4** | AEGGGFLGDTVIMKQLADGITR |
| **4** | ILNEAGEEVGEVTSGGWGPTAGK |
| **4** | AEGGGFLGDTVIMKQLADGITR |
| **4** | AAGKDVDLEVLDESALLALQGPK |
| **4** | ASHLWVRSSAGLFDVSHMGQVR |
| **4** | ASHLWVRSSAGLFDVSHMGQVR |
| **4** | GHEKILNEAGEEVGEVTSGGWGPTAGK |
| **4** | MVPFAGYSMPVQYSSEGIKASHLWVR |
| **4** | MVPFAGYSMPVQYSSEGIKASHLWVR |
| **4** | MVPFAGYSMPVQYSSEGIKASHLWVR |
| **4** | ILNEAGEEVGEVTSGGWGPTAGKALGMGYAK |
| **4** | ILNEAGEEVGEVTSGGWGPTAGKALGMGYAK |
| **4** | LEAGLCLYGNDIDETTSPVEAALTWTIGK |
| **4** | LEAGLCLYGNDIDETTSPVEAALTWTIGKR |
| **4** | TVLHDFHESHGGKMVPFAGYSMPVQYSSEGIK |
| **4** | TVLHDFHESHGGKMVPFAGYSMPVQYSSEGIK |
| **4** | DSLRLEAGLCLYGNDIDETTSPVEAALTWTIGK |
| **4** | TVLHDFHESHGGKMVPFAGYSMPVQYSSEGIK |
| **4** | AASVLAELAPSLNLSTMAFMSSAAADVAGIPCHVTR |
| **4** | AASVLAELAPSLNLSTMAFMSSAAADVAGIPCHVTR |
| **4** | AASVLAELAPSLNLSTMAFMSSAAADVAGIPCHVTR |
| **5** | EGLPLK |
| **5** | YFAAAR |
| **5** | SGGEAFR |
| **5** | LDLLVR |
| **5** | AQFKAGK |
| **5** | LGLEAPR |
| **5** | LRAQFK |
| **5** | SRLAFAK |
| **5** | LAFAKQK |
| **5** | ALPAGLDR |
| **5** | QVPMTVR |
| **5** | MNGVAVTK |
| **5** | QVPMTVR |
| **5** | QVPMTVR |
| **5** | MNGVAVTK |
| **5** | LGAGVVDGR |
| **5** | QVPMTVR |
| **5** | AEPFEVR |
| **5** | GDNLGLLR |
| **5** | ALAAAGYAR |
| **5** | AVGLDFTR |
| **5** | TAQGTIGASR |
| **5** | QKLDLLVR |
| **5** | TALEAHWR |
| **5** | QKLDLLVR |
| **5** | RQVPMTVR |
| **5** | RQVPMTVR |
| **5** | AEPFEVRR |
| **5** | AMVDAAADVR |
| **5** | AMVDAAADVR |
| **5** | THGWPEGVR |
| **5** | LGLEAPRHR |
| **5** | AFLAAGLAPDR |
| **5** | HRALPAGLDR |
| **5** | GAPGVAALDMSK |
| **5** | GAPGVAALDMSK |
| **5** | LWVVPDCGQK |
| **5** | IIQVDEPALR |
| **5** | TASPDLCSAALK |
| **5** | VAQAAAGDATPVK |
| **5** | TDMVEFFAEK |
| **5** | VRTAQGTIGASR |
| **5** | EEVADLEAAGAR |
| **5** | TDMVEFFAEK |
| **5** | QVPMTVREWK |
| **5** | QVPMTVREWK |
| **5** | QVPMTVREWK |
| **5** | QVPMTVREWK |
| **5** | EAQAMQLAVVVR |
| **5** | EAQAMQLAVVVR |
| **5** | ELKTALEAHWR |
| **5** | VRAFLAAGLAPDR |
| **5** | LPGVKAVGLDFTR |
| **5** | AMVDAAADVRAASK |
| **5** | MNGVAVTKAGWVR |
| **5** | AMVDAAADVRAASK |
| **5** | MNGVAVTKAGWVR |
| **5** | RPAWAADVAWAVR |
| **5** | ALPAGLDRYFAAAR |
| **5** | SGGEAFRALAAAGYAR |
| **5** | EWKVAQAAAGDATPVK |
| **5** | DTLGLDVLVHGELER |
| **5** | AVGLDFTRGDNLGLLR |
| **5** | SVWAEEAASAALLDAVR |
| **5** | DLSREAQAMQLAVVVR |
| **5** | GMLTGPTTILNWSFPR |
| **5** | IIQVDEPALREGLPLK |
| **5** | YFAAARGAPGVAALDMSK |
| **5** | DLSREAQAMQLAVVVR |
| **5** | GMLTGPTTILNWSFPR |
| **5** | YFAAARGAPGVAALDMSK |
| **5** | RPAWAADVAWAVRAFR |
| **5** | THGWPEGVRLGAGVVDGR |
| **5** | GDNLGLLRTHGWPEGVR |
| **5** | MAPTSATMTTTVGYPCMGGR |
| **5** | SVGSLCVRPPIIHGDLSR |
| **5** | MAPTSATMTTTVGYPCMGGR |
| **5** | GMLTGPTTILNWSFPRK |
| **5** | MAPTSATMTTTVGYPCMGGR |
| **5** | MAPTSATMTTTVGYPCMGGR |
| **5** | GMLTGPTTILNWSFPRK |
| **5** | MAPTSATMTTTVGYPCMGGR |
| **5** | MAPTSATMTTTVGYPCMGGR |
| **5** | MAPTSATMTTTVGYPCMGGR |
| **5** | MAPTSATMTTTVGYPCMGGR |
| **5** | MAPTSATMTTTVGYPCMGGR |
| **5** | MAPTSATMTTTVGYPCMGGR |
| **5** | TDMVEFFAEKMNGVAVTK |
| **5** | MAPTSATMTTTVGYPCMGGR |
| **5** | TDMVEFFAEKMNGVAVTK |
| **5** | LQKDTLGLDVLVHGELER |
| **5** | MAPTSATMTTTVGYPCMGGR |
| **5** | TDMVEFFAEKMNGVAVTK |
| **5** | LPSDEYLAAMDAATVECVR |
| **5** | MAPTSATMTTTVGYPCMGGRR |
| **5** | SVGSLCVRPPIIHGDLSRR |
| **5** | MAPTSATMTTTVGYPCMGGR |
| **5** | LPSDEYLAAMDAATVECVR |
| **5** | MAPTSATMTTTVGYPCMGGRR |
| **5** | MAPTSATMTTTVGYPCMGGR |
| **5** | MAPTSATMTTTVGYPCMGGRR |
| **5** | MAPTSATMTTTVGYPCMGGRR |
| **5** | MAPTSATMTTTVGYPCMGGRR |
| **5** | MAPTSATMTTTVGYPCMGGRR |
| **5** | EGLPLKRPAWAADVAWAVR |
| **5** | MAPTSATMTTTVGYPCMGGRR |
| **5** | MAPTSATMTTTVGYPCMGGRR |
| **5** | MAPTSATMTTTVGYPCMGGRR |
| **5** | MAPTSATMTTTVGYPCMGGRR |
| **5** | TASPDLCSAALKAMVDAAADVR |
| **5** | MAPTSATMTTTVGYPCMGGRR |
| **5** | TASPDLCSAALKAMVDAAADVR |
| **5** | MAPTSATMTTTVGYPCMGGRR |
| **5** | MAPTSATMTTTVGYPCMGGRR |
| **5** | AFLAAGLAPDRLWVVPDCGQK |
| **5** | MAPTSATMTTTVGYPCMGGRR |
| **5** | LWVVPDCGQKTASPDLCSAALK |
| **5** | AGKLPSDEYLAAMDAATVECVR |
| **5** | AGKLPSDEYLAAMDAATVECVR |
| **5** | QFSVNGGLGTMSIGSFPQTPAIR |
| **5** | QFSVNGGLGTMSIGSFPQTPAIR |
| **5** | QFSVNGGLGTMSIGSFPQTPAIR |
| **5** | EEVADLEAAGARIIQVDEPALR |
| **5** | DLAPGVFDIHSEVVPSAAEVATR |
| **5** | QFSVNGGLGTMSIGSFPQTPAIR |
| **5** | EAGVPEVQLHEPTLVTSDGPALR |
| **5** | LPSDEYLAAMDAATVECVRLQK |
| **5** | LPSDEYLAAMDAATVECVRLQK |
| **5** | AGWVRSVGSLCVRPPIIHGDLSR |
| **5** | QFSVNGGLGTMSIGSFPQTPAIRR |
| **5** | QFSVNGGLGTMSIGSFPQTPAIRR |
| **5** | QFSVNGGLGTMSIGSFPQTPAIRR |
| **5** | EAQAMQLAVVVREEVADLEAAGAR |
| **5** | QFSVNGGLGTMSIGSFPQTPAIRR |
| **5** | EAQAMQLAVVVREEVADLEAAGAR |
| **5** | SKQFSVNGGLGTMSIGSFPQTPAIR |
| **5** | LGAGVVDGRSVWAEEAASAALLDAVR |
| **5** | SKQFSVNGGLGTMSIGSFPQTPAIR |
| **5** | DLAPGVFDIHSEVVPSAAEVATRVR |
| **5** | ALREAGVPEVQLHEPTLVTSDGPALR |
| **5** | DTLGLDVLVHGELERTDMVEFFAEK |
| **5** | DTLGLDVLVHGELERTDMVEFFAEK |
| **5** | VAQAAAGDATPVKGMLTGPTTILNWSFPR |
| **5** | VAQAAAGDATPVKGMLTGPTTILNWSFPR |
| **5** | ALAAAGYARDLAPGVFDIHSEVVPSAAEVATR |
| **5** | AAAGAAVAVCVQPSCSLAHVPLDLDAETALAPAVK |
| **5** | AAAGAAVAVCVQPSCSLAHVPLDLDAETALAPAVKSR |
| **5** | WQNTNFHVLVPEIGGEGGAALPPPADAPGWTDLVAR |
| **5** | VAGEAVGDTSSAASDATPGAAVVNASAAITSAIDEALFER |
| **5** | VVPILLGPVTYAAGCALSGGVTVASLVEAITPSFVAALR |
| **6** | TAAPFGR |
| **6** | IATLFR |
| **6** | LYLDGR |
| **6** | QTVGDKVK |
| **6** | LDNVVAVK |
| **6** | QTVGDKVK |
| **6** | LQGWAVGPPR |
| **6** | MVKLYLDGR |
| **6** | MVKLYLDGR |
| **6** | IVTAMITPFK |
| **6** | IVTAMITPFK |
| **6** | LARLDNVVAVK |
| **6** | IATLFRELGLV |
| **6** | SSICMSAEVTAR |
| **6** | SSICMSAEVTAR |
| **6** | LPLTDATPEVEAK |
| **6** | VADATGLHLHYSR |
| **6** | EASGDLDLFTDIR |
| **6** | AGLRLQGWAVGPPR |
| **6** | IVTAMITPFKTHR |
| **6** | IVTAMITPFKTHR |
| **6** | SSICMSAEVTARLAR |
| **6** | EASGDLDLFTDIRR |
| **6** | SSICMSAEVTARLAR |
| **6** | LFADLFCMANPIPTK |
| **6** | LFADLFCMANPIPTK |
| **6** | LIAGAGSNSTEEAMEATR |
| **6** | TAAPFGRIVTAMITPFK |
| **6** | LIAGAGSNSTEEAMEATR |
| **6** | TAAPFGRIVTAMITPFK |
| **6** | PASPAPPPPATETALATYGR |
| **6** | LIAGAGSNSTEEAMEATRR |
| **6** | PASPAPPPPATETALATYGR |
| **6** | LIAGAGSNSTEEAMEATRR |
| **6** | VKLIAGAGSNSTEEAMEATR |
| **6** | VKLIAGAGSNSTEEAMEATR |
| **6** | LFADLFCMANPIPTKAGLR |
| **6** | LPLTDATPEVEAKIATLFR |
| **6** | LFADLFCMANPIPTKAGLR |
| **6** | HIAACEPDLPVMLYNIPGR |
| **6** | HIAACEPDLPVMLYNIPGR |
| **6** | LYLDGRVADATGLHLHYSR |
| **6** | LDNVVAVKEASGDLDLFTDIR |
| **6** | LQGWAVGPPRLPLTDATPEVEAK |
| **6** | PASPAPPPPATETALATYGRTAAPFGR |
| **6** | PASPAPPPPATETALATYGRTAAPFGR |
| **6** | VADATGLHLHYSRLFADLFCMANPIPTK |
| **6** | LQFDGTLQVVPYYNKPPQEGIYQHFK |
| **6** | VADATGLHLHYSRLFADLFCMANPIPTK |
| **6** | KLQFDGTLQVVPYYNKPPQEGIYQHFK |
| **6** | HIAACEPDLPVMLYNIPGRSSICMSAEVTAR |
| **6** | HIAACEPDLPVMLYNIPGRSSICMSAEVTAR |
| **6** | HIAACEPDLPVMLYNIPGRSSICMSAEVTAR |
| **7** | TTDILR |
| **7** | GLETGSR |
| **7** | VLGWHR |
| **7** | GAFLPGYR |
| **7** | FWSVDDK |
| **7** | GFGFGGGFLG |
| **7** | AVSTLRNR |
| **7** | MTSYAKPGVR |
| **7** | MTSYAKPGVR |
| **7** | MTSYAKPGVR |
| **7** | MTSYAKPGVR |
| **7** | SQIQHVALR |
| **7** | FGFTPLAYR |
| **7** | MPVNEPAPAR |
| **7** | MPVNEPAPAR |
| **7** | MHTEYSALR |
| **7** | MTSYAKPGVR |
| **7** | MHTEYSALR |
| **7** | MTSYAKPGVR |
| **7** | RSQIQHVALR |
| **7** | DEAGVVTVATIR |
| **7** | MPVNEPAPARR |
| **7** | MPVNEPAPARR |
| **7** | SVVVADPDEVIK |
| **7** | DSRPVGEGGSGGGR |
| **7** | TTDILRAVSTLR |
| **7** | DSRPVGEGGSGGGRR |
| **7** | HLATHGDGVHDVAFR |
| **7** | VLGWHRFWSVDDK |
| **7** | AAWPDTTHTFVSAYR |
| **7** | SQIQHVALRTTDILR |
| **7** | FGFTPLAYRGLETGSR |
| **7** | GVTFITVPDTYYEDLR |
| **7** | FWSVDDKMHTEYSALR |
| **7** | FWSVDDKMHTEYSALR |
| **7** | GVTFITVPDTYYEDLRR |
| **7** | GAFLPGYRDSRPVGEGGSGGGR |
| **7** | NRGVTFITVPDTYYEDLR |
| **7** | SVVVADPDEVIKMPVNEPAPAR |
| **7** | SVVVADPDEVIKMPVNEPAPAR |
| **7** | MHTEYSALRSVVVADPDEVIK |
| **7** | MHTEYSALRSVVVADPDEVIK |
| **7** | VDDAAAAYAMAVEAAGVSVSPPQELK |
| **7** | VDDAAAAYAMAVEAAGVSVSPPQELK |
| **7** | AAWPDTTHTFVSAYRGAFLPGYR |
| **7** | DEAGVVTVATIRAAWPDTTHTFVSAYR |
| **7** | SILTHVVTQGDITLAFSSALTPDNADMSR |
| **7** | SILTHVVTQGDITLAFSSALTPDNADMSR |
| **7** | TTPVTYSAFDHLHFYVGNAAQAAAYYITR |
| **7** | GLETGSRSILTHVVTQGDITLAFSSALTPDNADMSR |
| **7** | VDDAAAAYAMAVEAAGVSVSPPQELKDEAGVVTVATIR |
| **7** | GLETGSRSILTHVVTQGDITLAFSSALTPDNADMSR |
| **7** | VDDAAAAYAMAVEAAGVSVSPPQELKDEAGVVTVATIR |
| **9** | SAAYAAR |
| **9** | LTVVRK |
| **9** | DGGALKPK |
| **9** | VACETASK |
| **9** | DPTKVDR |
| **9** | DGGALKPKR |
| **9** | GLDYKTCR |
| **9** | SIGFDAEEK |
| **9** | TSAYGHFGR |
| **9** | SMVAAGLAER |
| **9** | SMVAAGLAER |
| **9** | VNYEEVIR |
| **9** | SDLMTHVIK |
| **9** | LGHRLTVVR |
| **9** | SDLMTHVIK |
| **9** | VDRSAAYAAR |
| **9** | EGFTWETVK |
| **9** | SDDELLAIVK |
| **9** | KTSAYGHFGR |
| **9** | TQVTVEYER |
| **9** | SAAYAARWVAK |
| **9** | ELDLLKPIFK |
| **9** | EGFTWETVKK |
| **9** | SDDELLAIVKK |
| **9** | PPPPPSVTMSAMK |
| **9** | PPPPPSVTMSAMK |
| **9** | PPPPPSVTMSAMK |
| **9** | NFDLRPGVLIK |
| **9** | IRSDLMTHVIK |
| **9** | IRSDLMTHVIK |
| **9** | PPPPPSVTMSAMK |
| **9** | ELDLLKPIFKK |
| **9** | PPPPPSVTMSAMK |
| **9** | EGICPWVRPDGK |
| **9** | PPPPPSVTMSAMK |
| **9** | KNFDLRPGVLIK |
| **9** | EAIKSIGFDAEEK |
| **9** | FVIGGPEGDAGLTGR |
| **9** | VNYEEVIREAIK |
| **9** | KEGICPWVRPDGK |
| **9** | WVAKSMVAAGLAER |
| **9** | WVAKSMVAAGLAER |
| **9** | SIGFDAEEKGLDYK |
| **9** | FVIGGPEGDAGLTGRK |
| **9** | TGMVMCFGEITTTAK |
| **9** | TGMVMCFGEITTTAK |
| **9** | TGMVMCFGEITTTAK |
| **9** | NFLFTSESVNEGHPDK |
| **9** | TQVTVEYERDGGALKPK |
| **9** | VIIDTYGGWGAHGGGAFSGK |
| **9** | VHTIVISTQHDDEVSNEK |
| **9** | TSAYGHFGREGFTWETVK |
| **9** | KVIIDTYGGWGAHGGGAFSGK |
| **9** | VLVELHAQSPEIAAGVHEGR |
| **9** | RVHTIVISTQHDDEVSNEK |
| **9** | LCDQVSDAVLDACLAQDPHSK |
| **9** | VHTIVISTQHDDEVSNEKIR |
| **9** | VACETASKTGMVMCFGEITTTAK |
| **9** | VIIDTYGGWGAHGGGAFSGKDPTK |
| **9** | VACETASKTGMVMCFGEITTTAK |
| **9** | VACETASKTGMVMCFGEITTTAK |
| **9** | EVIPAQYLDDDTIYHLNPSGR |
| **9** | EGICPWVRPDGKTQVTVEYER |
| **9** | NFDLRPGVLIKELDLLKPIFK |
| **9** | TCRVLVELHAQSPEIAAGVHEGR |
| **9** | TGMVMCFGEITTTAKVNYEEVIR |
| **9** | TGMVMCFGEITTTAKVNYEEVIR |
| **9** | TGMVMCFGEITTTAKVNYEEVIR |
| **9** | LLVQVSYGIGIAEPISVFVDSYGTGTK |
| **9** | LCDQVSDAVLDACLAQDPHSKVACETASK |
| **9** | PPPPPSVTMSAMKNFLFTSESVNEGHPDK |
| **9** | PPPPPSVTMSAMKNFLFTSESVNEGHPDK |
| **9** | PPPPPSVTMSAMKNFLFTSESVNEGHPDK |
| **9** | PPPPPSVTMSAMKNFLFTSESVNEGHPDK |
| **9** | PPPPPSVTMSAMKNFLFTSESVNEGHPDK |
| **9** | PPPPPSVTMSAMKNFLFTSESVNEGHPDK |
| **9** | SDLMTHVIKEVIPAQYLDDDTIYHLNPSGR |
| **9** | SDLMTHVIKEVIPAQYLDDDTIYHLNPSGR |
| **9** | TSDDLGAGDQGIMFGYATNESEDLMPLTHSLSTK |
| **9** | TSDDLGAGDQGIMFGYATNESEDLMPLTHSLSTK |
| **9** | TSDDLGAGDQGIMFGYATNESEDLMPLTHSLSTK |
| **9** | SMVAAGLAERLLVQVSYGIGIAEPISVFVDSYGTGTK |
| **9** | SMVAAGLAERLLVQVSYGIGIAEPISVFVDSYGTGTK |
| **9** | EVIPAQYLDDDTIYHLNPSGRFVIGGPEGDAGLTGR |
| **10** | MASSQPR |
| **10** | MASSQPR |
| **10** | MASSQPR |
| **10** | AVHLLR |
| **10** | SSCAWR |
| **10** | MASSQPR |
| **10** | MASSQPR |
| **10** | FKDTDAFA |
| **10** | SSCAWRVR |
| **10** | GLAAADALVAR |
| **10** | VVLHSYWK |
| **10** | IALNLLGIDYEYK |
| **10** | MASSQPRVVLHSYWK |
| **10** | MASSQPRVVLHSYWK |
| **10** | MASSQPRVVLHSYWK |
| **10** | MASSQPRVVLHSYWK |
| **10** | VVLHSYWKSSCAWR |
| **10** | FGVDVAAYPHVAAVVAR |
| **10** | VRIALNLLGIDYEYK |
| **10** | MASSQPRVVLHSYWK |
| **10** | MASSQPRVVLHSYWK |
| **10** | FGVDVAAYPHVAAVVARFK |
| **10** | VAAVAGGDDAASAAWGAHWVAK |
| **10** | ELVEVVNAGVQPLQNLAVLR |
| **10** | IALNLLGIDYEYKAVHLLR |
| **10** | RVAAVAGGDDAASAAWGAHWVAK |
| **10** | ELVEVVNAGVQPLQNLAVLRR |
| **10** | HGGGGLAVAAAGGVTAAEAVLVPAALAAPR |
| **10** | VAAVAGGDDAASAAWGAHWVAKGLAAADALVAR |
| **10** | ITQSVAILEYLAAAFPSPAVTLYPADAAAAAAVR |
| **10** | GLAAADALVARHGGGGLAVAAAGGVTAAEAVLVPAALAAPR |
| **11** | IAAEER |
| **11** | LHAATAR |
| **11** | TLGVRLR |
| **11** | ECYRAVK |
| **11** | GLRTLGVR |
| **11** | GFCDKFLGR |
| **11** | AHEAAGYTLAR |
| **11** | GAAWAAYGPNTK |
| **11** | WLEARPEVAR |
| **11** | AAAAEAPTTATASET |
| **11** | LHAATARIAAEER |
| **11** | LRAHEAAGYTLAR |
| **11** | YWEYGGGLGQTLR |
| **11** | GAAWAAYGPNTKVIK |
| **11** | GGHRPAEWSTDPLGR |
| **11** | YAAADWPFTGMWYGR |
| **11** | YAAADWPFTGMWYGR |
| **11** | SVKYWEYGGGLGQTLR |
| **11** | YIGGHSDLMLGLIATTR |
| **11** | YIGGHSDLMLGLIATTR |
| **11** | VMHPGLESHPQHALWK |
| **11** | VMHPGLESHPQHALWK |
| **11** | VIKGGHRPAEWSTDPLGR |
| **11** | IAAEERAAAAEAPTTATASET |
| **11** | SSVAELGCPPGSDDCYLALR |
| **11** | IHAGLEDTADLLADLEAGFAR |
| **11** | SVVNPPVYHASTVTFPTVAALR |
| **11** | AVKSSVAELGCPPGSDDCYLALR |
| **11** | FGVETTYYSPTASVADVAALFR |
| **11** | AHEAAGYTLARWLEARPEVAR |
| **11** | YIGGHSDLMLGLIATTRECYR |
| **11** | SSVAELGCPPGSDDCYLALRGLR |
| **11** | YIGGHSDLMLGLIATTRECYR |
| **11** | SASDEPANGAAAAEPPAPAPHSSLQMTGK |
| **11** | SASDEPANGAAAAEPPAPAPHSSLQMTGK |
| **11** | SASDEPANGAAAAEPPAPAPHSSLQMTGK |
| **11** | SASDEPANGAAAAEPPAPAPHSSLQMTGK |
| **11** | FGVETTYYSPTASVADVAALFRDNTK |
| **11** | VLFVESPASLSFEVMDVPALAAMAHAK |
| **11** | FLGRFGVETTYYSPTASVADVAALFR |
| **11** | VLFVESPASLSFEVMDVPALAAMAHAK |
| **11** | VLFVESPASLSFEVMDVPALAAMAHAK |
| **11** | IHAGLEDTADLLADLEAGFARLHAATAR |
| **11** | VIIDNTWGPMFLQPFDLGVDVSINAATK |
| **11** | WLEARPEVARVMHPGLESHPQHALWK |
| **11** | VIIDNTWGPMFLQPFDLGVDVSINAATK |
| **11** | VLFVESPASLSFEVMDVPALAAMAHAKGAK |
| **11** | WLEARPEVARVMHPGLESHPQHALWK |
| **11** | VLFVESPASLSFEVMDVPALAAMAHAKGAK |
| **11** | VLFVESPASLSFEVMDVPALAAMAHAKGAK |
| **11** | DNTKVLFVESPASLSFEVMDVPALAAMAHAK |
| **11** | DNTKVLFVESPASLSFEVMDVPALAAMAHAK |
| **11** | GAKVIIDNTWGPMFLQPFDLGVDVSINAATK |
| **11** | DNTKVLFVESPASLSFEVMDVPALAAMAHAK |
| **11** | GAKVIIDNTWGPMFLQPFDLGVDVSINAATK |
| **11** | YWEYGGGLGQTLRIHAGLEDTADLLADLEAGFAR |
| **11** | SASDEPANGAAAAEPPAPAPHSSLQMTGKGAAWAAYGPNTK |
| **12** | TKDAVK |
| **12** | GKTVIR |
| **12** | QTARNK |
| **12** | SSGLALR |
| **12** | GRTTIR |
| **12** | QTARNK |
| **12** | VKETER |
| **12** | ETERMK |
| **12** | FASVLEK |
| **12** | AITTELR |
| **12** | ETERMK |
| **12** | NVYNWR |
| **12** | SRDSIFK |
| **12** | RPFFHR |
| **12** | LGAVVEEGR |
| **12** | AITTELRK |
| **12** | VLLLASLCK |
| **12** | KLGAVVEEGR |
| **12** | MKAITTELR |
| **12** | NVYNWRVK |
| **12** | MKAITTELR |
| **12** | EPQCPLSAAAR |
| **12** | VLLLASLCKGK |
| **12** | MAMAFALAACGK |
| **12** | MAMAFALAACGK |
| **12** | MAMAFALAACGK |
| **12** | VGVDICDPGCTAK |
| **12** | TTIRNVYNWR |
| **12** | SYGTYILDGTPR |
| **12** | MSAGEGEPPAGWR |
| **12** | MSAGEGEPPAGWR |
| **12** | FFARPEPTALVK |
| **12** | NLLVSDDITYMK |
| **12** | NLLVSDDITYMK |
| **12** | ERPIIDLIEALR |
| **12** | NALSGLGVPVTSSTR |
| **12** | APPTIDHVVLPGDR |
| **12** | MSAGEGEPPAGWRR |
| **12** | MSAGEGEPPAGWRR |
| **12** | SLSNRVLLLASLCK |
| **12** | RAPPTIDHVVLPGDR |
| **12** | SSGLALRRPFFHR |
| **12** | QFGVTVETNNEMSR |
| **12** | FFARPEPTALVKTK |
| **12** | QFGVTVETNNEMSR |
| **12** | QFGVTVETNNEMSR |
| **12** | QFGVTVETNNEMSR |
| **12** | SYGTYILDGTPRMR |
| **12** | RAPPTIDHVVLPGDR |
| **12** | SYGTYILDGTPRMR |
| **12** | LPMASTTAFVPVFTGK |
| **12** | LPMASTTAFVPVFTGK |
| **12** | MGATVSWTPNSITVSR |
| **12** | MGATVSWTPNSITVSR |
| **12** | MRERPIIDLIEALR |
| **12** | EPQCPLSAAARSAPALR |
| **12** | MRERPIIDLIEALR |
| **12** | RLPMASTTAFVPVFTGK |
| **12** | HVAPISGFYGTVTLPGSK |
| **12** | RLPMASTTAFVPVFTGK |
| **12** | TFPAYFDQLEWLVSR |
| **12** | TVIRNLLVSDDITYMK |
| **12** | LSVCAADLFLPDANLATR |
| **12** | TVIRNLLVSDDITYMK |
| **12** | SAPALRMSAGEGEPPAGWR |
| **12** | APPTIDHVVLPGDRQTAR |
| **12** | MGATVSWTPNSITVSRSR |
| **12** | SAPALRMSAGEGEPPAGWR |
| **12** | TFPAYFDQLEWLVSRS |
| **12** | MGATVSWTPNSITVSRSR |
| **12** | RPFFHREPQCPLSAAAR |
| **12** | LMKQFGVTVETNNEMSR |
| **12** | RHVAPISGFYGTVTLPGSK |
| **12** | LMKQFGVTVETNNEMSR |
| **12** | LMKQFGVTVETNNEMSR |
| **12** | LSVCAADLFLPDANLATRR |
| **12** | GRLSVCAADLFLPDANLATR |
| **12** | ATLTAPAVEPTGHGDGTFFDR |
| **12** | VTIEVTDELTSKPYVLMTIK |
| **12** | VTIEVTDELTSKPYVLMTIK |
| **12** | EQSPLVYLGVAAVLALAALLASK |
| **12** | VSGQISSQYLSALLMAAPLAASK |
| **12** | VSGQISSQYLSALLMAAPLAASK |
| **12** | LPMASTTAFVPVFTGKSSGLALR |
| **12** | LPMASTTAFVPVFTGKSSGLALR |
| **12** | HVAPISGFYGTVTLPGSKSLSNR |
| **12** | GVDEDCGDIPDAAMTLAVAALFAK |
| **12** | GVDEDCGDIPDAAMTLAVAALFAK |
| **12** | MAMAFALAACGKVGVDICDPGCTAK |
| **12** | MAMAFALAACGKVGVDICDPGCTAK |
| **12** | MAMAFALAACGKVGVDICDPGCTAK |
| **12** | FASVLEKMGATVSWTPNSITVSR |
| **12** | EQSPLVYLGVAAVLALAALLASKAK |
| **12** | FASVLEKMGATVSWTPNSITVSR |
| **12** | MSAIAGAVAVAALTGGVRPASAIGGPGGR |
| **12** | MSAIAGAVAVAALTGGVRPASAIGGPGGR |
| **12** | DAVKATLTAPAVEPTGHGDGTFFDR |
| **12** | ATLTAPAVEPTGHGDGTFFDRTITK |
| **12** | GVDEDCGDIPDAAMTLAVAALFAKGR |
| **12** | GVDEDCGDIPDAAMTLAVAALFAKGR |
| **12** | VTIEVTDELTSKPYVLMTIKLMK |
| **12** | VTIEVTDELTSKPYVLMTIKLMK |
| **12** | VTIEVTDELTSKPYVLMTIKLMK |
| **12** | RMSAIAGAVAVAALTGGVRPASAIGGPGGR |
| **12** | RMSAIAGAVAVAALTGGVRPASAIGGPGGR |
| **12** | TITKEQSPLVYLGVAAVLALAALLASK |
| **12** | DSCVITPPPGGLIRPGVAVETYDDHR |
| **12** | NLLVSDDITYMKNALSGLGVPVTSSTR |
| **12** | NLLVSDDITYMKNALSGLGVPVTSSTR |
| **12** | GLGAHVTCSDTGCPPVVVNTTGEGLPGGTTR |
| **12** | DSIFKGVDEDCGDIPDAAMTLAVAALFAK |
| **12** | DSIFKGVDEDCGDIPDAAMTLAVAALFAK |
| **12** | VGVDICDPGCTAKTFPAYFDQLEWLVSR |
| **12** | AADAAGAVADAASAAASSTQSTPPANGAATAGPVSALK |
| **12** | AADAAGAVADAASAAASSTQSTPPANGAATAGPVSALKR |
| **12** | SKAADAAGAVADAASAAASSTQSTPPANGAATAGPVSALK |
| **12** | LGAVVEEGRDSCVITPPPGGLIRPGVAVETYDDHR |
| **13** | VCASGLK |
| **13** | GALGRTR |
| **13** | LPADAVR |
| **13** | TRLPADAVR |
| **13** | TPIGSFLGSLR |
| **13** | AVALAADSLALGR |
| **13** | GVPAVELAAVAIR |
| **13** | ASLPAATVCTTVNK |
| **13** | TAASTLHDVYIVSAAR |
| **13** | TAASTLHDVYIVSAAR |
| **13** | GVPAVELAAVAIRGALGR |
| **13** | ECVLGNVLGANAGQAPAR |
| **13** | AVALAADSLALGRGGGAGAAV |
| **13** | VCASGLKAVALAADSLALGR |
| **13** | VAALRASLPAATVCTTVNK |
| **13** | ASLPAATVCTTVNKVCASGLK |
| **13** | ECVLGNVLGANAGQAPARVAALR |
| **13** | TPIGSFLGSLRGVPAVELAAVAIR |
| **13** | LPADAVRECVLGNVLGANAGQAPAR |
| **13** | TAASTLHDVYIVSAARTPIGSFLGSLR |
| **13** | TAASTLHDVYIVSAARTPIGSFLGSLR |
| **14** | LHASAR |
| **14** | HVIGLK |
| **14** | NAAIYR |
| **14** | ALAEVAR |
| **14** | LSGFPAGR |
| **14** | QRPGESR |
| **14** | QRPGESR |
| **14** | GVGSLRGAK |
| **14** | VVPVSVAAK |
| **14** | GGVERVLR |
| **14** | SILPPLVAR |
| **14** | RVVPVSVAAK |
| **14** | VPLSEAEHTK |
| **14** | ALAEVARSVEA |
| **14** | VALASELGVAPK |
| **14** | VVGAAKHVIGLK |
| **14** | VFGSGTYLDSSR |
| **14** | MTDGAWAALHAR |
| **14** | MTDGAWAALHAR |
| **14** | LALVARNAAIYR |
| **14** | LHASARALAEVAR |
| **14** | LRVALASELGVAPK |
| **14** | QRPGESRLALVAR |
| **14** | QRPGESRLALVAR |
| **14** | VLRVPLSEAEHTK |
| **14** | VFGSGTYLDSSRLR |
| **14** | NAAIYRSILPPLVAR |
| **14** | VPLSEAEHTKLHASAR |
| **14** | MTDGAWAALHARVVGAAK |
| **14** | MTDGAWAALHARVVGAAK |
| **14** | FADIRMTDGAWAALHAR |
| **14** | FADIRMTDGAWAALHAR |
| **14** | GYTNWGVGAAVGALVGFILR |
| **14** | GTYGIEEDVFLSLPAVLGR |
| **14** | LSGFPAGRVFGSGTYLDSSR |
| **14** | AAAPNYDGSEASDVIIITAGAR |
| **14** | VEGEVLDFTHGGAFYHANVK |
| **14** | SPDAILLIVSNPVDILTAIAAR |
| **14** | KVEGEVLDFTHGGAFYHANVK |
| **14** | GYTNWGVGAAVGALVGFILRDEK |
| **14** | GTYGIEEDVFLSLPAVLGRGGVER |
| **14** | SVHASILGEHGDSSVAVASMANVGGAR |
| **14** | SVHASILGEHGDSSVAVASMANVGGAR |
| **14** | HVIGLKGYTNWGVGAAVGALVGFILR |
| **14** | GGGNDSGGSSGGIAGSLVDELCFPSVGGGGDR |
| **14** | GGGNDSGGSSGGIAGSLVDELCFPSVGGGGDR |
| **14** | VVPVSVAAKGTYGIEEDVFLSLPAVLGR |
| **14** | AAAPNYDGSEASDVIIITAGARQRPGESR |
| **14** | SPDAILLIVSNPVDILTAIAARLSGFPAGR |
| **14** | SVHASILGEHGDSSVAVASMANVGGARFADIR |
| **14** | SVHASILGEHGDSSVAVASMANVGGARFADIR |
| **14** | SILPPLVARSPDAILLIVSNPVDILTAIAAR |
| **14** | VTIVGCGSVGMACASAILSTGLASTLVFADVDAK |
| **14** | VTIVGCGSVGMACASAILSTGLASTLVFADVDAK |
| **14** | GGGNDSGGSSGGIAGSLVDELCFPSVGGGGDRGVGSLR |
| **14** | VTIVGCGSVGMACASAILSTGLASTLVFADVDAKK |
| **14** | VTIVGCGSVGMACASAILSTGLASTLVFADVDAKK |
| **14** | GGGNDSGGSSGGIAGSLVDELCFPSVGGGGDRGVGSLR |
| **14** | GAKVTIVGCGSVGMACASAILSTGLASTLVFADVDAK |
| **14** | GAKVTIVGCGSVGMACASAILSTGLASTLVFADVDAK |
| **14** | VALASELGVAPKSVHASILGEHGDSSVAVASMANVGGAR |
| **14** | VALASELGVAPKSVHASILGEHGDSSVAVASMANVGGAR |
| **15** | RLAFGK |
| **15** | MVVWER |
| **15** | MVVWER |
| **15** | MVVWERGAGR |
| **15** | MVVWERGAGR |
| **15** | VAMLAESAAAAPAAR |
| **15** | VAMLAESAAAAPAAR |
| **15** | VAMLAESAAAAPAAR |
| **15** | VAMLAESAAAAPAAR |
| **15** | VAMLAESAAAAPAARR |
| **15** | VAMLAESAAAAPAARR |
| **15** | VAMLAESAAAAPAARR |
| **15** | VAMLAESAAAAPAARR |
| **15** | ACGTGACAVVVAAVLTGR |
| **15** | YQGLGNDFILVDNR |
| **15** | VNTEFVTVDAPDAVR |
| **15** | LGPQFENLTSVFPAR |
| **15** | VYMTGPAELAFTGSVMV |
| **15** | IINSDGSEPEMCGNGVR |
| **15** | VYMTGPAELAFTGSVMV |
| **15** | IINSDGSEPEMCGNGVR |
| **15** | VYMTGPAELAFTGSVMV |
| **15** | DSTDLTLSPAQAVALCDR |
| **15** | TRACGTGACAVVVAAVLTGR |
| **15** | ACGTGACAVVVAAVLTGRTER |
| **15** | LAFGKYQGLGNDFILVDNR |
| **15** | DLERLGPQFENLTSVFPAR |
| **15** | TVTVGLPGGDLQIEWNEADGR |
| **15** | VNTEFVTVDAPDAVRMVVWER |
| **15** | VNTEFVTVDAPDAVRMVVWER |
| **15** | TERTVTVGLPGGDLQIEWNEADGR |
| **15** | VDMGVPVTDPPAVPTTLPATTAPVTVDGR |
| **15** | VDMGVPVTDPPAVPTTLPATTAPVTVDGR |
| **15** | HTGIGGDGVIFLLPPNAATGAAGDACTASMR |
| **15** | HTGIGGDGVIFLLPPNAATGAAGDACTASMR |
| **15** | EWTMTGVSMGNPHAVTFVSADDFVAIDK |
| **15** | EWTMTGVSMGNPHAVTFVSADDFVAIDK |
| **15** | EWTMTGVSMGNPHAVTFVSADDFVAIDK |
| **15** | LGPQFENLTSVFPARVNTEFVTVDAPDAVR |
| **15** | YQGLGNDFILVDNRDSTDLTLSPAQAVALCDR |
| **15** | EWTMTGVSMGNPHAVTFVSADDFVAIDKDLER |
| **15** | EWTMTGVSMGNPHAVTFVSADDFVAIDKDLER |
| **15** | EWTMTGVSMGNPHAVTFVSADDFVAIDKDLER |
| **15** | CLAVFAADLGMDPTTLVLDTLAGVIVPTVAPGGGAVR |
| **15** | CLAVFAADLGMDPTTLVLDTLAGVIVPTVAPGGGAVR |
| **16** | AIQQLR |
| **16** | FPVRTR |
| **16** | QYVASLR |
| **16** | TPRSTIR |
| **16** | QYVASLR |
| **16** | GGMSSATTR |
| **16** | GGMSSATTR |
| **16** | VATKFPVR |
| **16** | GSSMNDVAR |
| **16** | GSSMNDVAR |
| **16** | STIRVAIPSK |
| **16** | GLCRELVER |
| **16** | GSSMNDVARR |
| **16** | GSSMNDVARR |
| **16** | ASLSAPGPTGDALR |
| **16** | DSVYRAIQQLR |
| **16** | VVAAHEDLGYGGCR |
| **16** | VAIPSKGGMSSATTR |
| **16** | VAIPSKGGMSSATTR |
| **16** | GTETGMYAIGLVVPK |
| **16** | GTETGMYAIGLVVPK |
| **16** | AFFAAAGMDNYELVK |
| **16** | AFFAAAGMDNYELVK |
| **16** | LLEDVGMAVQLLNPR |
| **16** | LLEDVGMAVQLLNPR |
| **16** | ASLSAPGPTGDALRGLCR |
| **16** | WHALLQQLGVGGTAAEGA |
| **16** | EINGGTLVTSAFQLVGNR |
| **16** | TRAFFAAAGMDNYELVK |
| **16** | TRAFFAAAGMDNYELVK |
| **16** | RWHALLQQLGVGGTAAEGA |
| **16** | NAPGVEVWLQRPPDIVR |
| **16** | IDAHLMGQQQCNVIANIR |
| **16** | IDAHLMGQQQCNVIANIR |
| **16** | NAPGVEVWLQRPPDIVRK |
| **16** | GTETGMYAIGLVVPKDSVYR |
| **16** | GTETGMYAIGLVVPKDSVYR |
| **16** | SVGGSGVCVLPVTYVFEQESK |
| **16** | LGAQTDLCGMDGPTINSVVPPR |
| **16** | LGAQTDLCGMDGPTINSVVPPR |
| **16** | SVGGSGVCVLPVTYVFEQESKR |
| **16** | DGDVDLGFVGYDLVAEHGGSDGR |
| **16** | RLGAQTDLCGMDGPTINSVVPPR |
| **16** | RLGAQTDLCGMDGPTINSVVPPR |
| **16** | ENLLKEINGGTLVTSAFQLVGNR |
| **16** | LLEDVGMAVQLLNPRQYVASLR |
| **16** | LLEDVGMAVQLLNPRQYVASLR |
| **16** | GGMSSATTRLLEDVGMAVQLLNPR |
| **16** | GGMSSATTRLLEDVGMAVQLLNPR |
| **16** | GGMSSATTRLLEDVGMAVQLLNPR |
| **16** | VRDGDVDLGFVGYDLVAEHGGSDGR |
| **16** | ELVERIDAHLMGQQQCNVIANIR |
| **16** | ELVERIDAHLMGQQQCNVIANIR |
| **16** | QYVASLRNAPGVEVWLQRPPDIVR |
| **16** | QYVASLRNAPGVEVWLQRPPDIVR |
| **16** | MDGALEASTQMGTADVIVDLVSSGVTLR |
| **16** | MDGALEASTQMGTADVIVDLVSSGVTLR |
| **16** | MDGALEASTQMGTADVIVDLVSSGVTLR |
| **16** | AIQQLRSVGGSGVCVLPVTYVFEQESK |
| **16** | IDAHLMGQQQCNVIANIRGSSMNDVAR |
| **16** | IDAHLMGQQQCNVIANIRGSSMNDVAR |
| **16** | IDAHLMGQQQCNVIANIRGSSMNDVAR |
| **16** | LAIGVPMAWPHVSSVADLAAVVNAPGAAALR |
| **16** | LAIGVPMAWPHVSSVADLAAVVNAPGAAALR |
| **16** | EINGGTLVTSAFQLVGNRASLSAPGPTGDALR |
| **16** | LAIGVPMAWPHVSSVADLAAVVNAPGAAALRVATK |
| **16** | MDGALEASTQMGTADVIVDLVSSGVTLRENLLK |
| **16** | LAIGVPMAWPHVSSVADLAAVVNAPGAAALRVATK |
| **16** | MDGALEASTQMGTADVIVDLVSSGVTLRENLLK |
| **16** | MDGALEASTQMGTADVIVDLVSSGVTLRENLLK |
| **16** | LGAQTDLCGMDGPTINSVVPPRGTETGMYAIGLVVPK |
| **16** | LGAQTDLCGMDGPTINSVVPPRGTETGMYAIGLVVPK |
| **16** | DGDVDLGFVGYDLVAEHGGSDGRVVAAHEDLGYGGCR |
| **16** | LGAQTDLCGMDGPTINSVVPPRGTETGMYAIGLVVPK |
| **17** | QRAALR |
| **17** | QVRGEK |
| **17** | QRAALR |
| **17** | QVRGEK |
| **17** | EMQTPR |
| **17** | TIANTQK |
| **17** | EMQTPR |
| **17** | AALRQVR |
| **17** | MLFTSLNS |
| **17** | GAAGGGDAAAASR |
| **17** | MLFTSLNS |
| **17** | RTIANTQK |
| **17** | LPLTVLLTK |
| **17** | GMYAMLSLK |
| **17** | GMYAMLSLK |
| **17** | GAAGGGDAAAASR |
| **17** | GMYAMLSLK |
| **17** | GVVQGEPLFK |
| **17** | SLEEDDALAR |
| **17** | DRGMYAMLSLK |
| **17** | GMYAMLSLKQR |
| **17** | DRGMYAMLSLK |
| **17** | GMYAMLSLKQR |
| **17** | DRGMYAMLSLK |
| **17** | YPMELIEYLR |
| **17** | GMYAMLSLKQR |
| **17** | YPMELIEYLR |
| **17** | MEQVYTTYGQK |
| **17** | MEQVYTTYGQK |
| **17** | SLEEDDALARDR |
| **17** | TIANTQKEMQTPR |
| **17** | TIANTQKEMQTPR |
| **17** | GLVAVKGVVQGEPLFK |
| **17** | EMQTPRMLFTSLNS |
| **17** | VAGEYADADAPGGLFGR |
| **17** | EMQTPRMLFTSLNS |
| **17** | EMQTPRMLFTSLNS |
| **17** | TNAELLLLYGFVVDR |
| **17** | ALEAWLVDNGVYLSDK |
| **17** | KVAGEYADADAPGGLFGR |
| **17** | EELAPLAGSPTVAAAASLR |
| **17** | VAGEYADADAPGGLFGRTR |
| **17** | EELAPLAGSPTVAAAASLRR |
| **17** | APHPLAIANETTDDGEPSGR |
| **17** | GVVQGEPLFKLPLTVLLTK |
| **17** | NEVAVAQALLGACTAALAGYPR |
| **17** | FLEGAGQEAGSSVAFPLYADR |
| **17** | WTREELAPLAGSPTVAAAASLR |
| **17** | NPYDAVDVSVAVDEADPLYER |
| **17** | ALEAWLVDNGVYLSDKSSWGR |
| **17** | VRFLEGAGQEAGSSVAFPLYADR |
| **17** | NPYDAVDVSVAVDEADPLYERK |
| **17** | FCVATEEEMDSGADWGDFVSER |
| **17** | FCVATEEEMDSGADWGDFVSER |
| **17** | AVFDAAVYTEAAWGWAFAVLFSR |
| **17** | APHPLAIANETTDDGEPSGRGLVAVK |
| **17** | SSWGRAPHPLAIANETTDDGEPSGR |
| **17** | GAAGGGDAAAASRALEAWLVDNGVYLSDK |
| **17** | SLGDASFYAPYLGVLPADAELAPLFR |
| **17** | GAAGGGDAAAASRALEAWLVDNGVYLSDK |
| **17** | TRAVFDAAVYTEAAWGWAFAVLFSR |
| **17** | AVALQTFGDAMPADTDDWIAIAALLIR |
| **17** | AVALQTFGDAMPADTDDWIAIAALLIR |
| **17** | ERSLGDASFYAPYLGVLPADAELAPLFR |
| **17** | MEQVYTTYGQKTNAELLLLYGFVVDR |
| **17** | MEQVYTTYGQKTNAELLLLYGFVVDR |
| **17** | AVFDAAVYTEAAWGWAFAVLFSRLVLLR |
| **17** | AVALQTFGDAMPADTDDWIAIAALLIRER |
| **17** | AVALQTFGDAMPADTDDWIAIAALLIRER |
| **17** | SLGDASFYAPYLGVLPADAELAPLFRWTR |
| **17** | NEVAVAQALLGACTAALAGYPRSLEEDDALAR |
| **17** | FLEGAGQEAGSSVAFPLYADRYPMELIEYLR |
| **17** | FLEGAGQEAGSSVAFPLYADRYPMELIEYLR |
| **17** | YPMELIEYLRFCVATEEEMDSGADWGDFVSER |
| **17** | YPMELIEYLRFCVATEEEMDSGADWGDFVSER |
| **17** | YPMELIEYLRFCVATEEEMDSGADWGDFVSER |
| **17** | LPLTVLLTKAVALQTFGDAMPADTDDWIAIAALLIR |

Number= Table5 Number.

S3-4. Potential bioactive peptides of the Carbohydrate metabolism-Trypsin.

| **Number** | **Sequence** |
| --- | --- |
| **1** | AWVGAR |
| **1** | AIEEAK |
| **1** | TGKNIR |
| **1** | EAEPEK |
| **1** | VVTPAAAR |
| **1** | KAWVGAR |
| **1** | EDGLTVR |
| **1** | NIRYGVR |
| **1** | VVTPAAARR |
| **1** | YKEAEPEK |
| **1** | FLAIDAVEK |
| **1** | LAEGSYDGAK |
| **1** | VGAARQGTPR |
| **1** | ESVLPSAVGR |
| **1** | EALNWPYK |
| **1** | LREDGLTVR |
| **1** | TTPTLLVLTR |
| **1** | LAEGSYDGAKR |
| **1** | FNPANPSFVNR |
| **1** | ESVLPSAVGRER |
| **1** | LSVEAAASFGWSR |
| **1** | AWVGARVVTPAAAR |
| **1** | QGTGKLAEGSYDGAK |
| **1** | MQAFAADADGMPTR |
| **1** | QGTGKLAEGSYDGAK |
| **1** | MQAFAADADGMPTR |
| **1** | MQAFAADADGMPTR |
| **1** | AFEVPDEVMTHCR |
| **1** | FNPANPSFVNRDR |
| **1** | RLSVEAAASFGWSR |
| **1** | AFEVPDEVMTHCR |
| **1** | YADHFVSIEQFGR |
| **1** | TTPTLLVLTRQGTGK |
| **1** | MQAFAADADGMPTRK |
| **1** | MQAFAADADGMPTRK |
| **1** | AVNAIRFLAIDAVEK |
| **1** | HLKFNPANPSFVNR |
| **1** | MQAFAADADGMPTRK |
| **1** | AFEVPDEVMTHCRQK |
| **1** | MAFVAASLSAGCLAGGRPVR |
| **1** | AFEVPDEVMTHCRQK |
| **1** | MAFVAASLSAGCLAGGRPVR |
| **1** | MAFVAASLSAGCLAGGRPVR |
| **1** | MAFVAASLSAGCLAGGRPVR |
| **1** | VETGAALQADWEAQFAR |
| **1** | MAFVAASLSAGCLAGGRPVRK |
| **1** | MAFVAASLSAGCLAGGRPVR |
| **1** | MAFVAASLSAGCLAGGRPVR |
| **1** | MAFVAASLSAGCLAGGRPVRK |
| **1** | ANTAASHGSALGEDEVALTR |
| **1** | MAFVAASLSAGCLAGGRPVRK |
| **1** | MAFVAASLSAGCLAGGRPVRK |
| **1** | AAAFESAVVEGALPGDWEAK |
| **1** | MAFVAASLSAGCLAGGRPVRK |
| **1** | MAFVAASLSAGCLAGGRPVRK |
| **1** | QKVETGAALQADWEAQFAR |
| **1** | TVSLPCWEVFEAQSSEYK |
| **1** | QKVETGAALQADWEAQFAR |
| **1** | VETGAALQADWEAQFARYK |
| **1** | SGPGDEVLAYFGFTPEVVADR |
| **1** | ACTDKPSLINIVTTIGFGSPNK |
| **1** | SYGWQVIDVPNGNSDLGSIEK |
| **1** | AQSGHPGMPMGMAPTAFALWDR |
| **1** | AQSGHPGMPMGMAPTAFALWDR |
| **1** | AQSGHPGMPMGMAPTAFALWDR |
| **1** | AQSGHPGMPMGMAPTAFALWDR |
| **1** | SGPGDEVLAYFGFTPEVVADRAR |
| **1** | EALNWPYKAFEVPDEVMTHCR |
| **1** | EALNWPYKAFEVPDEVMTHCR |
| **1** | MDAVAPDASAVPAELMQTGEPFTDK |
| **1** | YRSYGWQVIDVPNGNSDLGSIEK |
| **1** | MDAVAPDASAVPAELMQTGEPFTDK |
| **1** | MDAVAPDASAVPAELMQTGEPFTDK |
| **1** | EAEPEKAAAFESAVVEGALPGDWEAK |
| **1** | AQSGHPGMPMGMAPTAFALWDRHLK |
| **1** | AQSGHPGMPMGMAPTAFALWDRHLK |
| **1** | AQSGHPGMPMGMAPTAFALWDRHLK |
| **1** | AQSGHPGMPMGMAPTAFALWDRHLK |
| **1** | AMPNMQVFRPADATETAAAYAVALHAK |
| **1** | AMPNMQVFRPADATETAAAYAVALHAK |
| **1** | EDGLTVRTVSLPCWEVFEAQSSEYK |
| **1** | AMPNMQVFRPADATETAAAYAVALHAK |
| **1** | LIAIYDDNSISIDGSTDLAMSEDTSDR |
| **1** | AIEEAKACTDKPSLINIVTTIGFGSPNK |
| **1** | SYGWQVIDVPNGNSDLGSIEKAIEEAK |
| **1** | LSVEAAASFGWSRYADHFVSIEQFGR |
| **1** | LIAIYDDNSISIDGSTDLAMSEDTSDR |
| **1** | ANTAASHGSALGEDEVALTREALNWPYK |
| **1** | TVSLPCWEVFEAQSSEYKESVLPSAVGR |
| **1** | QGTPRMDAVAPDASAVPAELMQTGEPFTDK |
| **1** | QGTPRMDAVAPDASAVPAELMQTGEPFTDK |
| **1** | QGTPRMDAVAPDASAVPAELMQTGEPFTDK |
| **1** | QGTPRMDAVAPDASAVPAELMQTGEPFTDK |
| **1** | QGTPRMDAVAPDASAVPAELMQTGEPFTDK |
| **1** | QGTPRMDAVAPDASAVPAELMQTGEPFTDK |
| **1** | LGKLIAIYDDNSISIDGSTDLAMSEDTSDR |
| **1** | MDAVAPDASAVPAELMQTGEPFTDKAVNAIR |
| **1** | LGKLIAIYDDNSISIDGSTDLAMSEDTSDR |
| **1** | MDAVAPDASAVPAELMQTGEPFTDKAVNAIR |
| **1** | LIAIYDDNSISIDGSTDLAMSEDTSDRYR |
| **1** | MDAVAPDASAVPAELMQTGEPFTDKAVNAIR |
| **1** | LIAIYDDNSISIDGSTDLAMSEDTSDRYR |
| **1** | FLAIDAVEKAQSGHPGMPMGMAPTAFALWDR |
| **1** | FLAIDAVEKAQSGHPGMPMGMAPTAFALWDR |
| **1** | FLAIDAVEKAQSGHPGMPMGMAPTAFALWDR |
| **1** | FLAIDAVEKAQSGHPGMPMGMAPTAFALWDR |
| **1** | AAAFESAVVEGALPGDWEAKMQAFAADADGMPTR |
| **1** | AAAFESAVVEGALPGDWEAKMQAFAADADGMPTR |
| **1** | AAAFESAVVEGALPGDWEAKMQAFAADADGMPTR |
| **1** | GGYVLSDNSPDGEAPQLLLIATGSEVDVIVGAAER |
| **1** | EHAMAAATTGIMASGYGLRPYCATFTIFTDYCK |
| **1** | EHAMAAATTGIMASGYGLRPYCATFTIFTDYCK |
| **1** | EHAMAAATTGIMASGYGLRPYCATFTIFTDYCK |
| **1** | RGGYVLSDNSPDGEAPQLLLIATGSEVDVIVGAAER |
| **1** | FVLSAGHGSMLLYALLYLFGFDSVSMEDIQQFR |
| **1** | FVLSAGHGSMLLYALLYLFGFDSVSMEDIQQFR |
| **1** | YADHFVSIEQFGRSGPGDEVLAYFGFTPEVVADR |
| **1** | GGYVLSDNSPDGEAPQLLLIATGSEVDVIVGAAERLR |
| **1** | FVLSAGHGSMLLYALLYLFGFDSVSMEDIQQFR |
| **2** | LGAAIVR |
| **2** | HRAAAGR |
| **2** | AGLVAALR |
| **2** | AGLVAALRSR |
| **2** | PPLAYGTAGFR |
| **2** | PPLAYGTAGFR |
| **2** | AAAPRLGAAIVR |
| **2** | LGAAIVRAGLVAALR |
| **2** | PPLAYGTAGFRAAAPR |
| **2** | PPLAYGTAGFRAAAPR |
| **2** | CDVPLVPPPGAPVSATVVIGR |
| **2** | ALGGAATGVVVTASHNPVADNGIK |
| **2** | SRALGGAATGVVVTASHNPVADNGIK |
| **2** | DTRPSSSPLAAALATAIAAAGGAVVDLGAATTPAVHYVVR |
| **3** | NVPVIK |
| **3** | LDAESR |
| **3** | MPPLLR |
| **3** | MPPLLR |
| **3** | IFAEQR |
| **3** | TIDGDLR |
| **3** | LSYRPR |
| **3** | LTEDYR |
| **3** | NVPVIKK |
| **3** | TYHFVR |
| **3** | GKNVPVIK |
| **3** | TRVIGCPK |
| **3** | KTYHFVR |
| **3** | NLFLLLPR |
| **3** | DPHGNVQVSK |
| **3** | GTPVTMGNVLR |
| **3** | GTPVTMGNVLR |
| **3** | ISTPEQFTAAR |
| **3** | TFAEQLMMDR |
| **3** | NQGGFHMVGSGR |
| **3** | ALVVLEEMAFK |
| **3** | TAATNVLHAASAK |
| **3** | TFAEQLMMDR |
| **3** | NQGGFHMVGSGR |
| **3** | ALVVLEEMAFK |
| **3** | TFAEQLMMDR |
| **3** | TYHFVRLMGR |
| **3** | TYHFVRLMGR |
| **3** | IFAEQRDSWR |
| **3** | MSAAVTTSAADAMAR |
| **3** | MSAAVTTSAADAMAR |
| **3** | DSWRLTEDYR |
| **3** | MSAAVTTSAADAMAR |
| **3** | MSAAVTTSAADAMAR |
| **3** | MSAAVTTSAADAMAR |
| **3** | KALVVLEEMAFK |
| **3** | MSAAVTTSAADAMAR |
| **3** | MSAAVTTSAADAMAR |
| **3** | VIGCPKTIDGDLR |
| **3** | LFIEMVSNELAK |
| **3** | KALVVLEEMAFK |
| **3** | LFIEMVSNELAK |
| **3** | TKISTPEQFTAAR |
| **3** | MSAAVTTSAADAMAR |
| **3** | AKTAATNVLHAASAK |
| **3** | MSAAVTTSAADAMAR |
| **3** | NQGGFHMVGSGRTK |
| **3** | MSAAVTTSAADAMAR |
| **3** | NQGGFHMVGSGRTK |
| **3** | LSYRPRMPPLLR |
| **3** | LSYRPRMPPLLR |
| **3** | MSAAVTTSAADAMARAK |
| **3** | MSAAVTTSAADAMARAK |
| **3** | MSAAVTTSAADAMARAK |
| **3** | LFIEMVSNELAKR |
| **3** | MSAAVTTSAADAMARAK |
| **3** | MSAAVTTSAADAMARAK |
| **3** | LFIEMVSNELAKR |
| **3** | MSAAVTTSAADAMARAK |
| **3** | MSAAVTTSAADAMARAK |
| **3** | CGGFPLTMMMNIER |
| **3** | ILASVVHQSPLHSAR |
| **3** | CGGFPLTMMMNIER |
| **3** | CGGFPLTMMMNIER |
| **3** | CGGFPLTMMMNIER |
| **3** | MSAAVTTSAADAMARAK |
| **3** | LDAESRNLFLLLPR |
| **3** | MSAAVTTSAADAMARAK |
| **3** | MSAAVTTSAADAMARAK |
| **3** | DPHGNVQVSKIESER |
| **3** | NEHVEMSFGFDTATK |
| **3** | NEHVEMSFGFDTATK |
| **3** | ISTPEQFTAARATVEK |
| **3** | VYSELTGNLGLDAISAK |
| **3** | CGGFPLTMMMNIERR |
| **3** | CGGFPLTMMMNIERR |
| **3** | CGGFPLTMMMNIERR |
| **3** | CGGFPLTMMMNIERR |
| **3** | VPPFSPVAHFFGYEGR |
| **3** | VYSELTGNLGLDAISAKK |
| **3** | QTLAQCVTSMVELIFER |
| **3** | SLFPVLFGQPTVALVPNR |
| **3** | TGYIATASNLTSPPEEWK |
| **3** | QTLAQCVTSMVELIFER |
| **3** | QTLAQCVTSMVELIFER |
| **3** | QTLAQCVTSMVELIFER |
| **3** | ALVVLEEMAFKIFAEQR |
| **3** | IESERLFIEMVSNELAK |
| **3** | ALVVLEEMAFKIFAEQR |
| **3** | IESERLFIEMVSNELAK |
| **3** | VTGNGGAAVAPPPTTAADHATR |
| **3** | KQTLAQCVTSMVELIFER |
| **3** | KQTLAQCVTSMVELIFER |
| **3** | GCFQVVEGAPTSAESDVDVIR |
| **3** | NLFLLLPRTFAEQLMMDR |
| **3** | NLFLLLPRTFAEQLMMDR |
| **3** | NLFLLLPRTFAEQLMMDR |
| **3** | CAGEKVPPFSPVAHFFGYEGR |
| **3** | TFAEQLMMDRDPHGNVQVSK |
| **3** | TFAEQLMMDRDPHGNVQVSK |
| **3** | TFAEQLMMDRDPHGNVQVSK |
| **3** | ILASVVHQSPLHSARLSYRPR |
| **3** | QTLAQCVTSMVELIFERAALGK |
| **3** | QTLAQCVTSMVELIFERAALGK |
| **3** | QTLAQCVTSMVELIFERAALGK |
| **3** | QTLAQCVTSMVELIFERAALGK |
| **3** | CPGPIQYSADANEVTITLASEEK |
| **3** | TIDGDLRNEHVEMSFGFDTATK |
| **3** | TIDGDLRNEHVEMSFGFDTATK |
| **3** | CPGPIQYSADANEVTITLASEEKAAK |
| **3** | ASLPSNFDCNLCYSLGNVAAALIEAGK |
| **3** | VGFVLSGGPASGGHNVISGLWDHLMER |
| **3** | VGFVLSGGPASGGHNVISGLWDHLMER |
| **3** | MPPLLRGCFQVVEGAPTSAESDVDVIR |
| **3** | SASHITLEVALQTHPNLTLIGEEVEAK |
| **3** | MPPLLRGCFQVVEGAPTSAESDVDVIR |
| **3** | MFGFIDGPSGICTGTYTEVTAELVNAFR |
| **3** | MFGFIDGPSGICTGTYTEVTAELVNAFR |
| **3** | SASHITLEVALQTHPNLTLIGEEVEAKK |
| **3** | SLFPVLFGQPTVALVPNRGTPVTMGNVLR |
| **3** | SLFPVLFGQPTVALVPNRGTPVTMGNVLR |
| **3** | LALDGLVVIGGDDSNTNAMLLAENFAANGLK |
| **3** | LALDGLVVIGGDDSNTNAMLLAENFAANGLK |
| **3** | LTEDYRCPGPIQYSADANEVTITLASEEK |
| **3** | TAATNVLHAASAKVTGNGGAAVAPPPTTAADHATR |
| **3** | LMGRSASHITLEVALQTHPNLTLIGEEVEAK |
| **3** | VGFVLSGGPASGGHNVISGLWDHLMERNMDSK |
| **3** | LALDGLVVIGGDDSNTNAMLLAENFAANGLKTR |
| **3** | LMGRSASHITLEVALQTHPNLTLIGEEVEAK |
| **3** | VGFVLSGGPASGGHNVISGLWDHLMERNMDSK |
| **3** | LALDGLVVIGGDDSNTNAMLLAENFAANGLKTR |
| **3** | VGFVLSGGPASGGHNVISGLWDHLMERNMDSK |
| **3** | NEHVEMSFGFDTATKVYSELTGNLGLDAISAK |
| **3** | NEHVEMSFGFDTATKVYSELTGNLGLDAISAK |
| **3** | TGYIATASNLTSPPEEWKCGGFPLTMMMNIER |
| **3** | TGYIATASNLTSPPEEWKCGGFPLTMMMNIER |
| **3** | NMDSKMFGFIDGPSGICTGTYTEVTAELVNAFR |
| **3** | TGYIATASNLTSPPEEWKCGGFPLTMMMNIER |
| **3** | NMDSKMFGFIDGPSGICTGTYTEVTAELVNAFR |
| **3** | TGYIATASNLTSPPEEWKCGGFPLTMMMNIER |
| **3** | NMDSKMFGFIDGPSGICTGTYTEVTAELVNAFR |
| **3** | VTGNGGAAVAPPPTTAADHATRILASVVHQSPLHSAR |
| **3** | ATVEKLALDGLVVIGGDDSNTNAMLLAENFAANGLK |
| **3** | ATVEKLALDGLVVIGGDDSNTNAMLLAENFAANGLK |
| **4** | TQEARK |
| **4** | RAGGEGTK |
| **4** | ATELFAK |
| **4** | ATCFVTR |
| **4** | DSCKAAVK |
| **4** | VDGVFGSR |
| **4** | AAVKAIAAK |
| **4** | VLEVPAES |
| **4** | AGGEGTKSR |
| **4** | STYMVGAR |
| **4** | MSDDAAAAAAK |
| **4** | STYMVGAR |
| **4** | MSDDAAAAAAK |
| **4** | MSDDAAAAAAK |
| **4** | MSDDAAAAAAK |
| **4** | HGSEEKPVK |
| **4** | MSDDAAAAAAK |
| **4** | MSDDAAAAAAK |
| **4** | STYMVGARR |
| **4** | STYMVGARR |
| **4** | AVADADAAGVAR |
| **4** | LMGESHASLR |
| **4** | LMGESHASLR |
| **4** | GQATEHEYAK |
| **4** | AHHVISEDVR |
| **4** | ATAGSALLIDCR |
| **4** | ALEAGDFVTVGR |
| **4** | ATCFVTRAGAGAR |
| **4** | AGAGARVLEVPAES |
| **4** | HGSEEKPVKALR |
| **4** | KALEAGDFVTVGR |
| **4** | ITGGGFGGCTVSLAK |
| **4** | ARAHHVISEDVR |
| **4** | VPCGIMDQLISSR |
| **4** | VPCGIMDQLISSR |
| **4** | AIAAKHGSEEKPVK |
| **4** | ALRGQATEHEYAK |
| **4** | ITGGGFGGCTVSLAKR |
| **4** | TYDGARPAVAAAAPGR |
| **4** | WANYVVGMVAMYVR |
| **4** | WANYVVGMVAMYVR |
| **4** | WANYVVGMVAMYVR |
| **4** | AHHVISEDVRTQEAR |
| **4** | KWANYVVGMVAMYVR |
| **4** | KWANYVVGMVAMYVR |
| **4** | DATLDMLASVSDDLDPK |
| **4** | KWANYVVGMVAMYVR |
| **4** | DATLDMLASVSDDLDPK |
| **4** | AVADADAAGVARATELFAK |
| **4** | MSDDAAAAAAKAVADADAAGVAR |
| **4** | MSDDAAAAAAKAVADADAAGVAR |
| **4** | MSDDAAAAAAKAVADADAAGVAR |
| **4** | MSDDAAAAAAKAVADADAAGVAR |
| **4** | WANYVVGMVAMYVRAAEK |
| **4** | DATLDMLASVSDDLDPKTR |
| **4** | WANYVVGMVAMYVRAAEK |
| **4** | DATLDMLASVSDDLDPKTR |
| **4** | MSDDAAAAAAKAVADADAAGVAR |
| **4** | WANYVVGMVAMYVRAAEK |
| **4** | MSDDAAAAAAKAVADADAAGVAR |
| **4** | DDYEVSVPEIDALVEIACK |
| **4** | EAVDALLAAVEAEYPAASGGVK |
| **4** | ALRDATLDMLASVSDDLDPK |
| **4** | ALRDATLDMLASVSDDLDPK |
| **4** | VDGVFGSRITGGGFGGCTVSLAK |
| **4** | REAVDALLAAVEAEYPAASGGVK |
| **4** | ATELFAKTYDGARPAVAAAAPGR |
| **4** | ALEAGDFVTVGRLMGESHASLR |
| **4** | ALEAGDFVTVGRLMGESHASLR |
| **4** | VNLIGEHTDYNDGWVFPLALEK |
| **4** | GQATEHEYAKVPCGIMDQLISSR |
| **4** | GQATEHEYAKVPCGIMDQLISSR |
| **4** | VPCGIMDQLISSRATAGSALLIDCR |
| **4** | VPCGIMDQLISSRATAGSALLIDCR |
| **4** | VVCEAFEGDVVEFDVAGDVKPLSGDR |
| **4** | VVCEAFEGDVVEFDVAGDVKPLSGDRK |
| **4** | EAVDALLAAVEAEYPAASGGVKATCFVTR |
| **4** | DDYEVSVPEIDALVEIACKVDGVFGSR |
| **4** | SRVVCEAFEGDVVEFDVAGDVKPLSGDR |
| **4** | LMGESHASLRDDYEVSVPEIDALVEIACK |
| **4** | LMGESHASLRDDYEVSVPEIDALVEIACK |
| **4** | VNLIGEHTDYNDGWVFPLALEKSTYMVGAR |
| **4** | VNLIGEHTDYNDGWVFPLALEKSTYMVGAR |
| **4** | SHEVTPVPLSDPSVVIVVTNSHVEHDLSGSEYPER |
| **5** | ESGKVR |
| **5** | LRESGK |
| **5** | AETVLGK |
| **5** | SVHEAVK |
| **5** | ALKELPR |
| **5** | DSFVVGSK |
| **5** | EASAAAAALCK |
| **5** | AETVLGKALK |
| **5** | DSFVVGSKVGR |
| **5** | VTASVEESLAR |
| **5** | EASAAAAALCKER |
| **5** | ELPRDSFVVGSK |
| **5** | YGESTFDFSAAR |
| **5** | VPPGTLDVVLSYCR |
| **5** | MTSGLSGADSGLSPHLR |
| **5** | AIFKPVANVAWASGK |
| **5** | MTSGLSGADSGLSPHLR |
| **5** | MTSGLSGADSGLSPHLR |
| **5** | MTSGLSGADSGLSPHLR |
| **5** | VGRYGESTFDFSAAR |
| **5** | YTLADDALAGMLPALK |
| **5** | YTLADDALAGMLPALK |
| **5** | MTSGLSGADSGLSPHLR |
| **5** | NVAVMMTPMDQALLR |
| **5** | MTSGLSGADSGLSPHLR |
| **5** | NVAVMMTPMDQALLR |
| **5** | MTSGLSGADSGLSPHLRR |
| **5** | NVAVMMTPMDQALLR |
| **5** | MTSGLSGADSGLSPHLRR |
| **5** | NVAVMMTPMDQALLR |
| **5** | MTSGLSGADSGLSPHLRR |
| **5** | MTSGLSGADSGLSPHLRR |
| **5** | VTASVEESLARLNVDR |
| **5** | LGVNFFDVSPYYGATK |
| **5** | MTSGLSGADSGLSPHLRR |
| **5** | MTSGLSGADSGLSPHLRR |
| **5** | DVKAIFKPVANVAWASGK |
| **5** | AIFKPVANVAWASGKFPGA |
| **5** | AVGITGYPLDIFPYVLDR |
| **5** | NVAVMMTPMDQALLRDVK |
| **5** | NVAVMMTPMDQALLRDVK |
| **5** | NVAVMMTPMDQALLRDVK |
| **5** | NVAVMMTPMDQALLRDVK |
| **5** | VRAVGITGYPLDIFPYVLDR |
| **5** | LGVNFFDVSPYYGATKAETVLGK |
| **5** | YGESTFDFSAARVTASVEESLAR |
| **5** | SVHEAVKLGVNFFDVSPYYGATK |
| **5** | LDLVQCHDIEFGDLDQVVNETLPALVK |
| **5** | AAGVGVLNAAPLSMGLLTDAGPPPWHPASAAVK |
| **5** | SLGSTGIDVSPLGFGASPLGGVFGDVDEADGVR |
| **5** | AAGVGVLNAAPLSMGLLTDAGPPPWHPASAAVK |
| **5** | VPPGTLDVVLSYCRYTLADDALAGMLPALK |
| **5** | VPPGTLDVVLSYCRYTLADDALAGMLPALK |
| **5** | LDLVQCHDIEFGDLDQVVNETLPALVKLR |
| **5** | RSLGSTGIDVSPLGFGASPLGGVFGDVDEADGVR |
| **5** | GSSLAALALQYALNADPLSVTSTVVGIDSTATLAK |
| **5** | AVGITGYPLDIFPYVLDRVPPGTLDVVLSYCR |
| **5** | LNVDRLDLVQCHDIEFGDLDQVVNETLPALVK |
| **5** | ERGSSLAALALQYALNADPLSVTSTVVGIDSTATLAK |
| **6** | VVDLMK |
| **6** | VVDLMK |
| **6** | DWRGGR |
| **6** | LSEGEMK |
| **6** | LTGMAFR |
| **6** | LSEGEMK |
| **6** | HVAAVDAK |
| **6** | LTGMAFR |
| **6** | VIPSLVGK |
| **6** | VIYSAPAK |
| **6** | ESTGVFLTK |
| **6** | KLSEGEMK |
| **6** | KLSEGEMK |
| **6** | AQSIIDGGAK |
| **6** | KVIYSAPAK |
| **6** | ESTGVFLTK |
| **6** | AQSIIDGGAKK |
| **6** | ESTGVFLTKEK |
| **6** | HVAAVDAKVAAE |
| **6** | AVTKVIPSLVGK |
| **6** | EKAQSIIDGGAK |
| **6** | ESTGVFLTKEK |
| **6** | AASGNIIPSSTGAAK |
| **6** | ETTYEEICAHVK |
| **6** | VVDLMKHVAAVDAK |
| **6** | VVDLMKHVAAVDAK |
| **6** | VPTIDVSVVDLTCR |
| **6** | ETTYEEICAHVKK |
| **6** | VIPSLVGKLTGMAFR |
| **6** | VIPSLVGKLTGMAFR |
| **6** | GGRAASGNIIPSSTGAAK |
| **6** | LVAWYDNEYGYSGR |
| **6** | LAKETTYEEICAHVK |
| **6** | AASGNIIPSSTGAAKAVTK |
| **6** | VPTIDVSVVDLTCRLAK |
| **6** | SSIFDADAGIMLNPNFVK |
| **6** | SSIFDADAGIMLNPNFVK |
| **6** | GFLGYSDEPLVSTDFEGDLR |
| **6** | LTGMAFRVPTIDVSVVDLTCR |
| **6** | LTGMAFRVPTIDVSVVDLTCR |
| **6** | LVAWYDNEYGYSGRVVDLMK |
| **6** | LVAWYDNEYGYSGRVVDLMK |
| **6** | LSEGEMKGFLGYSDEPLVSTDFEGDLR |
| **6** | LSEGEMKGFLGYSDEPLVSTDFEGDLR |
| **6** | AIHDEFEIEEALMTTVHAMTATQAVVDSSR |
| **6** | AIHDEFEIEEALMTTVHAMTATQAVVDSSR |
| **6** | AIHDEFEIEEALMTTVHAMTATQAVVDSSR |
| **6** | AIHDEFEIEEALMTTVHAMTATQAVVDSSRK |
| **6** | AIHDEFEIEEALMTTVHAMTATQAVVDSSRK |
| **6** | AIHDEFEIEEALMTTVHAMTATQAVVDSSRK |
| **6** | SSIFDADAGIMLNPNFVKLVAWYDNEYGYSGR |
| **6** | SSIFDADAGIMLNPNFVKLVAWYDNEYGYSGR |
| **7** | LSELLK |
| **7** | TITDDTR |
| **7** | LSELLKK |
| **7** | ELEAIAAK |
| **7** | FSLAPVATR |
| **7** | ELEAIAAKK |
| **7** | DLTAADLSGK |
| **7** | CDLNVPLDGK |
| **7** | TITDDTRIR |
| **7** | VLPGVAALDDQ |
| **7** | RPFAAIVGGSK |
| **7** | IGVIESMLDK |
| **7** | IGVIESMLDK |
| **7** | DLTAADLSGKR |
| **7** | VLLSSHLGRPK |
| **7** | IILPVDVVAADK |
| **7** | EETANDTEFAK |
| **7** | GLSTGSSLVEDDK |
| **7** | SIKDLTAADLSGK |
| **7** | ELAYLAGAVSNPK |
| **7** | EETANDTEFAKK |
| **7** | IELAKELEAIAAK |
| **7** | STELINSELADCK |
| **7** | LVIVGGMVFTFLK |
| **7** | LVIVGGMVFTFLK |
| **7** | IGVIESMLDKVDK |
| **7** | IGVIESMLDKVDK |
| **7** | RPFAAIVGGSKVSSK |
| **7** | ASIPTIEYLTAAGAK |
| **7** | VSSKIGVIESMLDK |
| **7** | VSSKIGVIESMLDK |
| **7** | ARGLSTGSSLVEDDK |
| **7** | GVKIILPVDVVAADK |
| **7** | VLVRCDLNVPLDGK |
| **7** | SGPEDKFSLAPVATR |
| **7** | FSLAPVATRLSELLK |
| **7** | LVIVGGMVFTFLKAR |
| **7** | LVIVGGMVFTFLKAR |
| **7** | ATTSVASSSSVFVASNR |
| **7** | FYKEETANDTEFAK |
| **7** | VDKLVIVGGMVFTFLK |
| **7** | IRASIPTIEYLTAAGAK |
| **7** | VDKLVIVGGMVFTFLK |
| **7** | MSHISTGGGASLELLEGK |
| **7** | MSHISTGGGASLELLEGK |
| **7** | VLLSSHLGRPKSGPEDK |
| **7** | ATTSVASSSSVFVASNRR |
| **7** | GLSTGSSLVEDDKIELAK |
| **7** | CDLNVPLDGKTITDDTR |
| **7** | LAANADMFVNDAFGTAHR |
| **7** | LAANADMFVNDAFGTAHR |
| **7** | SSLPLPITMAFVSAPAALR |
| **7** | SSLPLPITMAFVSAPAALR |
| **7** | KLAANADMFVNDAFGTAHR |
| **7** | KLAANADMFVNDAFGTAHR |
| **7** | TGAAAKATTSVASSSSVFVASNR |
| **7** | DRSSLPLPITMAFVSAPAALR |
| **7** | DRSSLPLPITMAFVSAPAALR |
| **7** | VQVPAAAATPAAPSGVVMQATATAK |
| **7** | VQVPAAAATPAAPSGVVMQATATAK |
| **7** | AHGSTAGVTEFLRPSVAGLLLEK |
| **7** | AGLAEKMSHISTGGGASLELLEGK |
| **7** | AGLAEKMSHISTGGGASLELLEGK |
| **7** | ELAYLAGAVSNPKRPFAAIVGGSK |
| **7** | VQVPAAAATPAAPSGVVMQATATAKK |
| **7** | SSLPLPITMAFVSAPAALRTGAAAK |
| **7** | VQVPAAAATPAAPSGVVMQATATAKK |
| **7** | SSLPLPITMAFVSAPAALRTGAAAK |
| **7** | RVQVPAAAATPAAPSGVVMQATATAK |
| **7** | RVQVPAAAATPAAPSGVVMQATATAK |
| **7** | ASIPTIEYLTAAGAKVLLSSHLGRPK |
| **7** | FAPDANTQLVSVDAIPEGTMGLDQGPK |
| **7** | FAPDANTQLVSVDAIPEGTMGLDQGPK |
| **7** | MSHISTGGGASLELLEGKVLPGVAALDDQ |
| **7** | MSHISTGGGASLELLEGKVLPGVAALDDQ |
| **7** | MAPDCIGDGVAEIVSGMSNGDVVLLENVR |
| **7** | MAPDCIGDGVAEIVSGMSNGDVVLLENVR |
| **7** | TQSISSCSIAPSCFPVAVAVAVAVPSSPSSK |
| **7** | MAPDCIGDGVAEIVSGMSNGDVVLLENVR |
| **7** | TQSISSCSIAPSCFPVAVAVAVAVPSSPSSKR |
| **7** | LSRTQSISSCSIAPSCFPVAVAVAVAVPSSPSSK |
| **7** | DVKMAPDCIGDGVAEIVSGMSNGDVVLLENVR |
| **7** | DVKMAPDCIGDGVAEIVSGMSNGDVVLLENVR |
| **7** | LSRTQSISSCSIAPSCFPVAVAVAVAVPSSPSSK |
| **7** | DVKMAPDCIGDGVAEIVSGMSNGDVVLLENVR |
| **7** | MAPDCIGDGVAEIVSGMSNGDVVLLENVRFYK |
| **7** | MAPDCIGDGVAEIVSGMSNGDVVLLENVRFYK |
| **7** | MAPDCIGDGVAEIVSGMSNGDVVLLENVRFYK |
| **7** | AHGSTAGVTEFLRPSVAGLLLEKELAYLAGAVSNPK |
| **8** | RDETDG |
| **8** | KSPTGTR |
| **8** | CGGVYGSR |
| **8** | SPTGTRGR |
| **8** | LVAEEMR |
| **8** | LVAEEMR |
| **8** | RCGGVYGSR |
| **8** | CGGVYGSRR |
| **8** | LIARSPLAR |
| **8** | SPLARALNR |
| **8** | LVAEEMRR |
| **8** | LVAEEMRR |
| **8** | EQGVGTIWSVYK |
| **8** | EQGVGTIWSVYKR |
| **8** | YVAASGSSCYSHGTLVADAHAALMTR |
| **8** | YVAASGSSCYSHGTLVADAHAALMTR |
| **8** | VLEMGVTDDAHLTTGFLAGVVDVIEK |
| **8** | VLEMGVTDDAHLTTGFLAGVVDVIEK |
| **8** | LDDPSPVVLPARPGAASDDDDDADAQR |
| **8** | RVLEMGVTDDAHLTTGFLAGVVDVIEK |
| **8** | RVLEMGVTDDAHLTTGFLAGVVDVIEK |
| **8** | EGGGRPASAPMASHPPVLSSEFATQLLWR |
| **8** | EGGGRPASAPMASHPPVLSSEFATQLLWR |
| **8** | EGGGRPASAPMASHPPVLSSEFATQLLWR |
| **8** | EGGGRPASAPMASHPPVLSSEFATQLLWR |
| **8** | FRLDDPSPVVLPARPGAASDDDDDADAQR |
| **8** | ALNRYVAASGSSCYSHGTLVADAHAALMTR |
| **8** | ALNRYVAASGSSCYSHGTLVADAHAALMTR |
| **8** | LDDPSPVVLPARPGAASDDDDDADAQRRPR |
| **8** | GVVVAPSTELLCEAAPLALLVEHAGGTATDGYGR |
| **8** | GVVVAPSTELLCEAAPLALLVEHAGGTATDGYGRR |
| **8** | VLEMGVTDDAHLTTGFLAGVVDVIEKLVAEEMR |
| **8** | VLEMGVTDDAHLTTGFLAGVVDVIEKLVAEEMR |
| **8** | VLEMGVTDDAHLTTGFLAGVVDVIEKLVAEEMR |
| **9** | VSAINR |
| **9** | YAGNNK |
| **9** | TKANLR |
| **9** | ALLAAEK |
| **9** | KGTLSTR |
| **9** | MTLLGTR |
| **9** | FGNGYLK |
| **9** | IVIQLGR |
| **9** | MTLLGTR |
| **9** | YGDKEMK |
| **9** | YGDKEMK |
| **9** | VSAINRTK |
| **9** | GTLSTRDR |
| **9** | ALLAAEKAGK |
| **9** | FESVKLLGS |
| **9** | VVLEDPACR |
| **9** | TKFGNGYLK |
| **9** | EMKVSAINR |
| **9** | EMKVSAINR |
| **9** | AALEKYAGNNK |
| **9** | DRVVLEDPACR |
| **9** | MTLLGTRFESVK |
| **9** | AFSNDFANAFER |
| **9** | MTLLGTRFESVK |
| **9** | FGNGYLKALLAAEK |
| **9** | VVLEDPACRAALEK |
| **9** | GAVLVNSNVSTLTLGGK |
| **9** | KGAVLVNSNVSTLTLGGK |
| **9** | GAVLVNSNVSTLTLGGKTK |
| **9** | ELVLWHGAVGVLGDIGASR |
| **9** | YAGNNKAFSNDFANAFER |
| **9** | AFSNDFANAFERMTLLGTR |
| **9** | AFSNDFANAFERMTLLGTR |
| **9** | DGVSVADFFAFAGAVAVEVAAGPR |
| **9** | LELGRPQNAGVADAVSAIGALAAAGK |
| **9** | AAAAGVTAAAAVGLLPAPPPAAATVDVDTDR |
| **9** | AAAAGVTAAAAVGLLPAPPPAAATVDVDTDR |
| **9** | DGVSVADFFAFAGAVAVEVAAGPRIVIQLGR |
| **9** | AAAAGVTAAAAVGLLPAPPPAAATVDVDTDRYGDK |
| **9** | AAAAGVTAAAAVGLLPAPPPAAATVDVDTDRYGDK |
| **9** | EEANAALAGDAEEEEVDAMTYGGGPTGNEDVGAVGVK |
| **9** | EDATEPDPPSSSALWADDAPAEELVAALNASGIGGAK |
| **9** | EEANAALAGDAEEEEVDAMTYGGGPTGNEDVGAVGVK |
| **9** | EEANAALAGDAEEEEVDAMTYGGGPTGNEDVGAVGVKK |
| **9** | EEANAALAGDAEEEEVDAMTYGGGPTGNEDVGAVGVKK |
| **9** | NLLATRPSLLSSAVLVGLHDTLTYDTATGAGGLNGSLR |
| **10** | APIVAAK |
| **10** | SRPVGGR |
| **10** | VAAAAAVR |
| **10** | SQSAPPR |
| **10** | STLAPTR |
| **10** | GSVTGSNK |
| **10** | IEVFGGK |
| **10** | IGQVHAK |
| **10** | RHALTR |
| **10** | TFTITSR |
| **10** | LLVGFQR |
| **10** | AISSTLGAK |
| **10** | FDSNFQK |
| **10** | STLAPTRR |
| **10** | LDEVDLVK |
| **10** | CGFGYDQR |
| **10** | LLVGFQRR |
| **10** | SRPVGGRAAR |
| **10** | AARVAAAAAVR |
| **10** | RFDSNFQK |
| **10** | AAQLSVQEGR |
| **10** | LVAVADPFEK |
| **10** | RCGFGYDQR |
| **10** | FDSNFQKVK |
| **10** | SVVSSGAIGDVR |
| **10** | VCCRSQSAPPR |
| **10** | TGVKLLVGFQR |
| **10** | DPSPPPADYLAK |
| **10** | LVRLDEVDLVK |
| **10** | HALTRSRPVGGR |
| **10** | LDEVDLVKAAAGVA |
| **10** | VKSVVSSGAIGDVR |
| **10** | MSNGAFGTIENSR |
| **10** | MSNGAFGTIENSR |
| **10** | DAGVGASSSAQAALGR |
| **10** | AAQLSVQEGRLVR |
| **10** | LVAVADPFEKFGR |
| **10** | FFGSPTPTDECSLR |
| **10** | IEVFGGKGSVTGSNK |
| **10** | MSNGAFGTIENSRR |
| **10** | MSNGAFGTIENSRR |
| **10** | FFGSPTPTDECSLR |
| **10** | IGQVHAKAISSTLGAK |
| **10** | TLPVPHPDSTPPPHR |
| **10** | CGFGYDQRIEVFGGK |
| **10** | SVAAEFSTTWVADWK |
| **10** | APIVAAKAAQLSVQEGR |
| **10** | AAANNSVPSTPPDSLPHR |
| **10** | DAGVGASSSAQAALGRAVPR |
| **10** | SGGIFLDMASHDFDMAR |
| **10** | SGGIFLDMASHDFDMAR |
| **10** | SGGIFLDMASHDFDMAR |
| **10** | DSHRDAGVGASSSAQAALGR |
| **10** | AISSTLGAKLVAVADPFEK |
| **10** | FFGSPTPTDECSLRDSHR |
| **10** | SVVSSGAIGDVRTFTITSR |
| **10** | FFGSPTPTDECSLRDSHR |
| **10** | FGRSVAAEFSTTWVADWK |
| **10** | TFTITSRDPSPPPADYLAK |
| **10** | TLPVPHPDSTPPPHRVCCR |
| **10** | AVPRAAANNSVPSTPPDSLPHR |
| **10** | MDAGAEASGSPGTPKPVGIGIIGCGR |
| **10** | MDAGAEASGSPGTPKPVGIGIIGCGR |
| **10** | SIAFPASPSLLTMAFVTAVGVGAPTR |
| **10** | SIAFPASPSLLTMAFVTAVGVGAPTR |
| **10** | SADTVTVSTEAGIGAGLPYDFFMDR |
| **10** | SADTVTVSTEAGIGAGLPYDFFMDR |
| **10** | AIFCEKPISNDLATIDSCLEVVER |
| **10** | YAAAYTGIMGAFVTMVAQDGAVPVGGADGR |
| **10** | YAAAYTGIMGAFVTMVAQDGAVPVGGADGR |
| **10** | YAAAYTGIMGAFVTMVAQDGAVPVGGADGR |
| **10** | AIFCEKPISNDLATIDSCLEVVERTGVK |
| **10** | VAAAAAVRMDAGAEASGSPGTPKPVGIGIIGCGR |
| **10** | DPSPPPADYLAKSGGIFLDMASHDFDMAR |
| **10** | VAAAAAVRMDAGAEASGSPGTPKPVGIGIIGCGR |
| **10** | MDAGAEASGSPGTPKPVGIGIIGCGRIGQVHAK |
| **10** | DPSPPPADYLAKSGGIFLDMASHDFDMAR |
| **10** | MDAGAEASGSPGTPKPVGIGIIGCGRIGQVHAK |
| **10** | DPSPPPADYLAKSGGIFLDMASHDFDMAR |
| **10** | ELVANPDVDGVVIGSPTPFHAEQIIACAEAGK |
| **10** | SQSAPPRSIAFPASPSLLTMAFVTAVGVGAPTR |
| **10** | SIAFPASPSLLTMAFVTAVGVGAPTRSTLAPTR |
| **10** | SQSAPPRSIAFPASPSLLTMAFVTAVGVGAPTR |
| **10** | SIAFPASPSLLTMAFVTAVGVGAPTRSTLAPTR |
| **10** | GSVTGSNKSADTVTVSTEAGIGAGLPYDFFMDR |
| **10** | GSVTGSNKSADTVTVSTEAGIGAGLPYDFFMDR |
| **10** | AAANNSVPSTPPDSLPHRTLPVPHPDSTPPPHR |
| **10** | FVCGAEIESVYVTGAAIESAAQEAGDLDTVVTVLK |
| **10** | YAAAYTGIMGAFVTMVAQDGAVPVGGADGRAPIVAAK |
| **10** | YAAAYTGIMGAFVTMVAQDGAVPVGGADGRAPIVAAK |
| **10** | YAAAYTGIMGAFVTMVAQDGAVPVGGADGRAPIVAAK |
| **11** | EVAQVK |
| **11** | LIIPLR |
| **11** | DLAHMR |
| **11** | DLAHMR |
| **11** | EHLSAAR |
| **11** | ALGMGYAK |
| **11** | ALGMGYAK |
| **11** | QLADGITR |
| **11** | GKEVAQVK |
| **11** | MRTVVEK |
| **11** | ASHLWVR |
| **11** | QLADGITR |
| **11** | TSYFSLTG |
| **11** | MRTVVEK |
| **11** | HMSSEALK |
| **11** | LIIPLRGK |
| **11** | TVVEKGGAR |
| **11** | HMSSEALK |
| **11** | KPFNKGTK |
| **11** | DKDLAHMR |
| **11** | DKDLAHMR |
| **11** | GTKLIIPLR |
| **11** | QLADGITRR |
| **11** | QLADGITRR |
| **11** | RHMSSEALK |
| **11** | HMSSEALKR |
| **11** | RHMSSEALK |
| **11** | HMSSEALKR |
| **11** | EHLSAARAAGK |
| **11** | EVAQVKSPVVK |
| **11** | PAAAAAAVATASTPR |
| **11** | PAAAAAAVATASTPR |
| **11** | AEGGLGLGDTVIMK |
| **11** | AEGGLGLGDTVIMK |
| **11** | PAAAAAAVATASTPRR |
| **11** | SPVVKTSYFSLTG |
| **11** | ALGMGYAKKPFNK |
| **11** | ALGMGYAKKPFNK |
| **11** | TVLHDFHESHGGK |
| **11** | PAAAAAAVATASTPRR |
| **11** | DLAHMREHLSAAR |
| **11** | RAEGGLGLGDTVIMK |
| **11** | DLAHMREHLSAAR |
| **11** | RAEGGLGLGDTVIMK |
| **11** | SSAGLFDVSHMGQVR |
| **11** | SSAGLFDVSHMGQVR |
| **11** | RTVLHDFHESHGGK |
| **11** | SSAGLFDVSHMGQVRLR |
| **11** | SSAGLFDVSHMGQVRLR |
| **11** | DVDLEVLDESALLALQGPK |
| **11** | ILNEAGEEVGEVTSGGWGPK |
| **11** | MVPFAGYSMPVQYSSEGIK |
| **11** | MVPFAGYSMPVQYSSEGIK |
| **11** | MVPFAGYSMPVQYSSEGIK |
| **11** | KILNEAGEEVGEVTSGGWGPK |
| **11** | AEGGLGLGDTVIMKQLADGITR |
| **11** | AEGGLGLGDTVIMKQLADGITR |
| **11** | AAGKDVDLEVLDESALLALQGPK |
| **11** | ASHLWVRSSAGLFDVSHMGQVR |
| **11** | ASHLWVRSSAGLFDVSHMGQVR |
| **11** | ILNEAGEEVGEVTSGGWGPKALGMGYAK |
| **11** | ILNEAGEEVGEVTSGGWGPKALGMGYAK |
| **11** | LEAGLYGLYGNDIDETTSPVEAALTWK |
| **11** | MVPFAGYSMPVQYSSEGIKASHLWVR |
| **11** | MVPFAGYSMPVQYSSEGIKASHLWVR |
| **11** | MVPFAGYSMPVQYSSEGIKASHLWVR |
| **11** | LEAGLYGLYGNDIDETTSPVEAALTWKR |
| **11** | DSLRLEAGLYGLYGNDIDETTSPVEAALTWK |
| **11** | AASVLAELAPSLNLSTMAFMSSAAADVAGIPCHTR |
| **11** | AASVLAELAPSLNLSTMAFMSSAAADVAGIPCHTR |
| **11** | AASVLAELAPSLNLSTMAFMSSAAADVAGIPCHTR |
| **11** | TVLHDFHESHGGKMVPFAGYSMPVQYSSEGIK |
| **11** | TVLHDFHESHGGKMVPFAGYSMPVQYSSEGIK |
| **11** | TVLHDFHESHGGKMVPFAGYSMPVQYSSEGIK |
| **12** | LPIIAK |
| **12** | MAAMNK |
| **12** | TSASGQK |
| **12** | MAAMNK |
| **12** | HGDGSGR |
| **12** | GLEQLK |
| **12** | MAAMNK |
| **12** | ALSSAVR |
| **12** | SRLSGVK |
| **12** | LMGFQR |
| **12** | LMGFQR |
| **12** | GVRPRGR |
| **12** | YLDTAPK |
| **12** | LPIIAKR |
| **12** | KGLEQLK |
| **12** | TSASGQKR |
| **12** | GLASSLPSH |
| **12** | VAEFKER |
| **12** | VYMNVNR |
| **12** | SAVLRAYK |
| **12** | VYMNVNR |
| **12** | AAVRNEIR |
| **12** | HFLSACVR |
| **12** | NSKEWQR |
| **12** | EFGSWQAK |
| **12** | ELVAAINDK |
| **12** | FDDHLWR |
| **12** | WNDVDEGR |
| **12** | VNPWDLEK |
| **12** | RGLASSLPSH |
| **12** | TDGSYVEQK |
| **12** | HFYQGFCK |
| **12** | QEREGVADR |
| **12** | IMLMYTDR |
| **12** | VVEVYDGDR |
| **12** | GSLGLDYDGR |
| **12** | IMLMYTDR |
| **12** | QEREGVADR |
| **12** | VVLVQVAIPK |
| **12** | IMLMYTDR |
| **12** | YLDTAPKMR |
| **12** | DSTTGAWSFK |
| **12** | VSHIGVDPER |
| **12** | YLDTAPKMR |
| **12** | WELFEENR |
| **12** | AIAASPEVELK |
| **12** | EASEPAQVVVK |
| **12** | TNGPRTSASGQK |
| **12** | LCGVLDMVVQK |
| **12** | LLNLEHEANR |
| **12** | WNDVDEGRVK |
| **12** | LCGVLDMVVQK |
| **12** | GIALKLMGFQR |
| **12** | GIALKLMGFQR |
| **12** | GKVVLVQVAIPK |
| **12** | RDSTTGAWSFK |
| **12** | VLPWRNELLK |
| **12** | AIAASPEVELKR |
| **12** | DKVVEVYDGDR |
| **12** | VYMNVNRMFR |
| **12** | VYMNVNRMFR |
| **12** | VYMNVNRMFR |
| **12** | VVLVQVAIPKAAR |
| **12** | EFGSWQAKEMR |
| **12** | DPINDVYIMSGR |
| **12** | EFGSWQAKEMR |
| **12** | SGHMIVNDGEFGK |
| **12** | FSEGLSAPTLTDR |
| **12** | DPINDVYIMSGR |
| **12** | GQLVLSEFTGCSR |
| **12** | SGHMIVNDGEFGK |
| **12** | NEIRELVAAINDK |
| **12** | QFTCTVGVKPSNAR |
| **12** | DPINDVYIMSGRK |
| **12** | QFTCTVGVKPSNAR |
| **12** | TAGIVWHYLDADR |
| **12** | DPINDVYIMSGRK |
| **12** | LMGFQRYLDTAPK |
| **12** | LMGFQRYLDTAPK |
| **12** | WDDDALYLTSTGLR |
| **12** | ELVAAINDKHGDGSGR |
| **12** | ALSSAVRVNPWDLEK |
| **12** | GSLGLDYDGRHVMLR |
| **12** | WELFEENRTLTTR |
| **12** | GSLGLDYDGRHVMLR |
| **12** | HVMLRVSHIGVDPER |
| **12** | WDDDALYLTSTGLRK |
| **12** | HVMLRVSHIGVDPER |
| **12** | LALYSIMDALVVTPIR |
| **12** | LALYSIMDALVVTPIR |
| **12** | LLEDADLSWMELTLR |
| **12** | FADCTVLGAVDDLDLIK |
| **12** | LLEDADLSWMELTLR |
| **12** | FDDHLWRVYMNVNR |
| **12** | FDDHLWRVYMNVNR |
| **12** | VAPLWVGILNSEDEVPR |
| **12** | RPVWYLEESISFESR |
| **12** | GMLSATLLGFHLFDYAR |
| **12** | GMLSATLLGFHLFDYAR |
| **12** | IGFFLHIPWPSSEVYR |
| **12** | TLTTRSGHMIVNDGEFGK |
| **12** | FSEGLSAPTLTDRVAEFK |
| **12** | TLTTRSGHMIVNDGEFGK |
| **12** | DGLNLIPYEYIVSTSEGK |
| **12** | IMLMYTDRTDGSYVEQK |
| **12** | IMLMYTDRTDGSYVEQK |
| **12** | GQLVLSEFTGCSRALSSAVR |
| **12** | IMLMYTDRTDGSYVEQK |
| **12** | ERFADCTVLGAVDDLDLIK |
| **12** | MTPGSVAADAAGSVGADTMDGSMK |
| **12** | MTPGSVAADAAGSVGADTMDGSMK |
| **12** | LLEEFNCVPVFIPHDTLK |
| **12** | MTPGSVAADAAGSVGADTMDGSMK |
| **12** | HFLSACVRLLNLEHEANR |
| **12** | TDEDMFTYLDTHLDPSVK |
| **12** | MTPGSVAADAAGSVGADTMDGSMK |
| **12** | MTPGSVAADAAGSVGADTMDGSMK |
| **12** | MTPGSVAADAAGSVGADTMDGSMK |
| **12** | TDEDMFTYLDTHLDPSVK |
| **12** | MTPGSVAADAAGSVGADTMDGSMK |
| **12** | MTPGSVAADAAGSVGADTMDGSMK |
| **12** | MTPGSVAADAAGSVGADTMDGSMK |
| **12** | MTPGSVAADAAGSVGADTMDGSMK |
| **12** | VNPWDLEKLCGVLDMVVQK |
| **12** | VNPWDLEKLCGVLDMVVQK |
| **12** | TLIWVHDYHLMLLPQALR |
| **12** | MTPGSVAADAAGSVGADTMDGSMK |
| **12** | TLIWVHDYHLMLLPQALR |
| **12** | LLNLEHEANRGSLGLDYDGR |
| **12** | MTPGSVAADAAGSVGADTMDGSMK |
| **12** | VGPLAGLPGVLTYDEFTLLNR |
| **12** | MTPGSVAADAAGSVGADTMDGSMK |
| **12** | MTPGSVAADAAGSVGADTMDGSMK |
| **12** | FADCTVLGAVDDLDLIKGIALK |
| **12** | AYVSAHSSQQWAQSFLHDLK |
| **12** | VAPLWVGILNSEDEVPRQER |
| **12** | LCGVLDMVVQKAIAASPEVELK |
| **12** | LCGVLDMVVQKAIAASPEVELK |
| **12** | SGHMIVNDGEFGKWNDVDEGR |
| **12** | SGHMIVNDGEFGKWNDVDEGR |
| **12** | EWQRLLEDADLSWMELTLR |
| **12** | EWQRLLEDADLSWMELTLR |
| **12** | LSGVKIGFFLHIPWPSSEVYR |
| **12** | TAGIVWHYLDADREFGSWQAK |
| **12** | CFLTPDEGFGDAIESLVIVLYR |
| **12** | TLIWVHDYHLMLLPQALRSR |
| **12** | TLIWVHDYHLMLLPQALRSR |
| **12** | VSHIGVDPERFSEGLSAPTLTDR |
| **12** | NELLKGMLSATLLGFHLFDYAR |
| **12** | GTLWPLFHMVSSATDHTEHTTR |
| **12** | TDGSYVEQKTAGIVWHYLDADR |
| **12** | NELLKGMLSATLLGFHLFDYAR |
| **12** | GASISLADLVPDDEGSSSTPPMPQR |
| **12** | GTLWPLFHMVSSATDHTEHTTR |
| **12** | GASISLADLVPDDEGSSSTPPMPQR |
| **12** | GLEQLKVAPLWVGILNSEDEVPR |
| **12** | HGDGSGRRPVWYLEESISFESR |
| **12** | YYLHSSNEVGALLETLVTGSYPR |
| **12** | IGFFLHIPWPSSEVYRVLPWR |
| **12** | ADKAYVSAHSSQQWAQSFLHDLK |
| **12** | VKCFLTPDEGFGDAIESLVIVLYR |
| **12** | DSTTGAWSFKWDDDALYLTSTGLR |
| **12** | TEVLEAGLNNSPTIGIAAEHGFFYR |
| **12** | DHLESLLTPFSVQVVSGYGWLQVR |
| **12** | GRGASISLADLVPDDEGSSSTPPMPQR |
| **12** | EGVADRLLEEFNCVPVFIPHDTLK |
| **12** | GRGASISLADLVPDDEGSSSTPPMPQR |
| **12** | VGPLAGLPGVLTYDEFTLLNRSAVLR |
| **12** | GVMVETILQDMPEPPDFVLCCGDDR |
| **12** | GVMVETILQDMPEPPDFVLCCGDDR |
| **12** | GVMVETILQDMPEPPDFVLCCGDDR |
| **12** | GMLSATLLGFHLFDYARHFLSACVR |
| **12** | LLEDADLSWMELTLRIMLMYTDR |
| **12** | TEVLEAGLNNSPTIGIAAEHGFFYRK |
| **12** | KTEVLEAGLNNSPTIGIAAEHGFFYR |
| **12** | GMLSATLLGFHLFDYARHFLSACVR |
| **12** | LLEDADLSWMELTLRIMLMYTDR |
| **12** | LLEDADLSWMELTLRIMLMYTDR |
| **12** | LLEDADLSWMELTLRIMLMYTDR |
| **12** | GASISLADLVPDDEGSSSTPPMPQRTNGPR |
| **12** | GASISLADLVPDDEGSSSTPPMPQRTNGPR |
| **12** | CFLTPDEGFGDAIESLVIVLYRLPIIAK |
| **12** | LLEEFNCVPVFIPHDTLKHFYQGFCK |
| **12** | YYLHSSNEVGALLETLVTGSYPRGVRPR |
| **12** | EMRDHLESLLTPFSVQVVSGYGWLQVR |
| **12** | EMRDHLESLLTPFSVQVVSGYGWLQVR |
| **12** | MTPGSVAADAAGSVGADTMDGSMKWELFEENR |
| **12** | MTPGSVAADAAGSVGADTMDGSMKWELFEENR |
| **12** | MTPGSVAADAAGSVGADTMDGSMKWELFEENR |
| **12** | MTPGSVAADAAGSVGADTMDGSMKWELFEENR |
| **12** | MTPGSVAADAAGSVGADTMDGSMKWELFEENR |
| **12** | MTPGSVAADAAGSVGADTMDGSMKWELFEENR |
| **12** | VVEVYDGDRTLIWVHDYHLMLLPQALR |
| **12** | MTPGSVAADAAGSVGADTMDGSMKWELFEENR |
| **12** | MTPGSVAADAAGSVGADTMDGSMKWELFEENR |
| **12** | VVEVYDGDRTLIWVHDYHLMLLPQALR |
| **12** | MTPGSVAADAAGSVGADTMDGSMKWELFEENR |
| **12** | MTPGSVAADAAGSVGADTMDGSMKWELFEENR |
| **12** | MTPGSVAADAAGSVGADTMDGSMKWELFEENR |
| **12** | MTPGSVAADAAGSVGADTMDGSMKWELFEENR |
| **12** | MTPGSVAADAAGSVGADTMDGSMKWELFEENR |
| **12** | DGLNLIPYEYIVSTSEGKGQLVLSEFTGCSR |
| **12** | DHLESLLTPFSVQVVSGYGWLQVRMAAMNK |
| **12** | MTPGSVAADAAGSVGADTMDGSMKWELFEENR |
| **12** | EASEPAQVVVKVGPLAGLPGVLTYDEFTLLNR |
| **12** | DHLESLLTPFSVQVVSGYGWLQVRMAAMNK |
| **12** | DHLESLLTPFSVQVVSGYGWLQVRMAAMNK |
| **12** | MAAMNKGVMVETILQDMPEPPDFVLCCGDDR |
| **12** | AYVSAHSSQQWAQSFLHDLKEASEPAQVVVK |
| **12** | MAAMNKGVMVETILQDMPEPPDFVLCCGDDR |
| **12** | MAAMNKGVMVETILQDMPEPPDFVLCCGDDR |
| **12** | MAAMNKGVMVETILQDMPEPPDFVLCCGDDR |
| **12** | MAAMNKGVMVETILQDMPEPPDFVLCCGDDR |
| **12** | GTLWPLFHMVSSATDHTEHTTRFDDHLWR |
| **12** | GTLWPLFHMVSSATDHTEHTTRFDDHLWR |
| **12** | HFYQGFCKGTLWPLFHMVSSATDHTEHTTR |
| **12** | HFYQGFCKGTLWPLFHMVSSATDHTEHTTR |
| **12** | TDEDMFTYLDTHLDPSVKQFTCTVGVKPSNAR |
| **12** | TDEDMFTYLDTHLDPSVKQFTCTVGVKPSNAR |
| **12** | RPVWYLEESISFESRLALYSIMDALVVTPIR |
| **12** | RPVWYLEESISFESRLALYSIMDALVVTPIR |
| **12** | LALYSIMDALVVTPIRDGLNLIPYEYIVSTSEGK |
| **12** | LALYSIMDALVVTPIRDGLNLIPYEYIVSTSEGK |
| **13** | QAAFVR |
| **13** | QAAFVR |
| **13** | ALDEKK |
| **13** | TDAELR |
| **13** | QPTKYK |
| **13** | VEVRMK |
| **13** | QPTKYK |
| **13** | YKAFSR |
| **13** | VEVRMK |
| **13** | AGKALDEK |
| **13** | LILDCLR |
| **13** | EDIGTEGR |
| **13** | GKQAAFVR |
| **13** | QHAGEEER |
| **13** | QHAGEEER |
| **13** | VPANELVLR |
| **13** | LVVEKPFGK |
| **13** | QMTIVGYAR |
| **13** | LILDCLRGK |
| **13** | WAGVPFVMK |
| **13** | QMTIVGYAR |
| **13** | QMTIVGYAR |
| **13** | SEKTDAELR |
| **13** | WAGVPFVMK |
| **13** | QMTIVGYAR |
| **13** | DAWRPVLVK |
| **13** | MADSPSAYTR |
| **13** | DDELRAAWK |
| **13** | MADSPSAYTR |
| **13** | DTASFEELSK |
| **13** | GPAEADEFVSK |
| **13** | MAGGTAVAERPPK |
| **13** | VQPNEAVYMK |
| **13** | MAGGTAVAERPPK |
| **13** | ETADGQEVANR |
| **13** | VQPNEAVYMK |
| **13** | MAGGTAVAERPPK |
| **13** | MAGGTAVAERPPK |
| **13** | AAGENFGDYVR |
| **13** | EGALSKTGWNR |
| **13** | EMNQNLLVLR |
| **13** | GGYFDSFGIIR |
| **13** | EMNQNLLVLR |
| **13** | MAGGTAVAERPPK |
| **13** | WAGVPFVMKAGK |
| **13** | IDAFLSTVIYR |
| **13** | MAGGTAVAERPPK |
| **13** | QAAFVRDDELR |
| **13** | WAGVPFVMKAGK |
| **13** | QAAFVRDDELR |
| **13** | HYVESVVITFK |
| **13** | VLHAIKPISLDK |
| **13** | QMTIVGYARSEK |
| **13** | DPTYEWHPPCK |
| **13** | QMTIVGYARSEK |
| **13** | QMTIVGYARSEK |
| **13** | QMTIVGYARSEK |
| **13** | IFTPLLHAIEAGK |
| **13** | LTAPALYELYQR |
| **13** | IDAFLSTVIYRR |
| **13** | FANSMFEPLWNR |
| **13** | FANSMFEPLWNR |
| **13** | IFTPLLHAIEAGKK |
| **13** | VKVLHAIKPISLDK |
| **13** | KLTAPALYELYQR |
| **13** | AAGENFGDYVRDEK |
| **13** | FDDKMADSPSAYTR |
| **13** | AFSRGPAEADEFVSK |
| **13** | FDDKMADSPSAYTR |
| **13** | VQPNEAVYMKTNVK |
| **13** | TGWNRLVVEKPFGK |
| **13** | VQPNEAVYMKTNVK |
| **13** | DYLPKQMTIVGYAR |
| **13** | DYLPKQMTIVGYAR |
| **13** | FHTPAAATTMFPDIR |
| **13** | FHTPAAATTMFPDIR |
| **13** | VAAGAPPTQEEVAAAPDK |
| **13** | GPAEADEFVSKFYQR |
| **13** | TDAELRDAWRPVLVK |
| **13** | DPTYEWHPPCKSDAK |
| **13** | AAWKIFTPLLHAIEAGK |
| **13** | MADSPSAYTRLILDCLR |
| **13** | YLAESLTILVIGASGDLAK |
| **13** | MKFHTPAAATTMFPDIR |
| **13** | MADSPSAYTRLILDCLR |
| **13** | MKFHTPAAATTMFPDIR |
| **13** | TPGLAGAPVASELDLSYGSR |
| **13** | FYQRDPTYEWHPPCK |
| **13** | MKFHTPAAATTMFPDIR |
| **13** | LFYFAVPPSVFADIGGAIK |
| **13** | DAWRPVLVKQHAGEEER |
| **13** | LTAPALYELYQRDYLPK |
| **13** | YLAESLTILVIGASGDLAKK |
| **13** | EDIGTEGRGGYFDSFGIIR |
| **13** | GQYDSAEDVAGVFAEMEER |
| **13** | GQYDSAEDVAGVFAEMEER |
| **13** | LVVEKPFGKDTASFEELSK |
| **13** | VPANELVLRVQPNEAVYMK |
| **13** | HYVESVVITFKEDIGTEGR |
| **13** | VPANELVLRVQPNEAVYMK |
| **13** | QHAGEEERIDAFLSTVIYR |
| **13** | QHAGEEERIDAFLSTVIYR |
| **13** | VAAGAPPTQEEVAAAPDKAAAGEK |
| **13** | RGQYDSAEDVAGVFAEMEER |
| **13** | RGQYDSAEDVAGVFAEMEER |
| **13** | TINGLFTPEQTYFVDHYLGK |
| **13** | DVMQNHLTQMLALIAMDPPVR |
| **13** | TNVKTPGLAGAPVASELDLSYGSR |
| **13** | DVMQNHLTQMLALIAMDPPVR |
| **13** | DVMQNHLTQMLALIAMDPPVR |
| **13** | DVMQNHLTQMLALIAMDPPVR |
| **13** | AAAGEKYLAESLTILVIGASGDLAK |
| **13** | TPGLAGAPVASELDLSYGSRFDDK |
| **13** | LFYFAVPPSVFADIGGAIKEGALSK |
| **13** | FHTPAAATTMFPDIRVPANELVLR |
| **13** | FHTPAAATTMFPDIRVPANELVLR |
| **13** | EMNQNLLVLRFANSMFEPLWNR |
| **13** | EMNQNLLVLRFANSMFEPLWNR |
| **13** | EMNQNLLVLRFANSMFEPLWNR |
| **13** | FANSMFEPLWNRHYVESVVITFK |
| **13** | FANSMFEPLWNRHYVESVVITFK |
| **13** | MAGGTAVAERPPKVAAGAPPTQEEVAAAPDK |
| **13** | MAGGTAVAERPPKVAAGAPPTQEEVAAAPDK |
| **13** | MAGGTAVAERPPKVAAGAPPTQEEVAAAPDK |
| **13** | MAGGTAVAERPPKVAAGAPPTQEEVAAAPDK |
| **13** | MAGGTAVAERPPKVAAGAPPTQEEVAAAPDK |
| **13** | MAGGTAVAERPPKVAAGAPPTQEEVAAAPDK |
| **13** | ETADGQEVANRLFYFAVPPSVFADIGGAIK |
| **13** | GQYDSAEDVAGVFAEMEERETADGQEVANR |
| **13** | GQYDSAEDVAGVFAEMEERETADGQEVANR |
| **13** | DTASFEELSKTINGLFTPEQTYFVDHYLGK |
| **13** | TINGLFTPEQTYFVDHYLGKEMNQNLLVLR |
| **13** | TINGLFTPEQTYFVDHYLGKEMNQNLLVLR |
| **13** | DVMQNHLTQMLALIAMDPPVRAAGENFGDYVR |
| **13** | DVMQNHLTQMLALIAMDPPVRAAGENFGDYVR |
| **13** | DVMQNHLTQMLALIAMDPPVRAAGENFGDYVR |
| **13** | GGYFDSFGIIRDVMQNHLTQMLALIAMDPPVR |
| **13** | DVMQNHLTQMLALIAMDPPVRAAGENFGDYVR |
| **13** | GGYFDSFGIIRDVMQNHLTQMLALIAMDPPVR |
| **13** | GGYFDSFGIIRDVMQNHLTQMLALIAMDPPVR |
| **13** | GGYFDSFGIIRDVMQNHLTQMLALIAMDPPVR |
| **14** | STLGPPR |
| **14** | RSSSAAR |
| **14** | SVGSFGGR |
| **14** | APWTRR |
| **14** | RAPWTR |
| **14** | RSTLGPPR |
| **14** | TAPWGSGPK |
| **14** | SDPMAILR |
| **14** | SDPMAILR |
| **14** | THSIDACLR |
| **14** | TLLMLNTLK |
| **14** | SDPMAILRR |
| **14** | TLLMLNTLK |
| **14** | SDPMAILRR |
| **14** | EECEEPIIVK |
| **14** | DMGAIFGVFR |
| **14** | DMGAIFGVFR |
| **14** | RTHSIDACLR |
| **14** | THSIDACLRR |
| **14** | EECEEPIIVK |
| **14** | EEPTLADGDPR |
| **14** | SVGSFGGRSPTR |
| **14** | YIGSMVPEVHR |
| **14** | YIGSMVPEVHR |
| **14** | SSSAARSVGSFGGR |
| **14** | EEPTLADGDPRSCR |
| **14** | SCRYIGSMVPEVHR |
| **14** | GGAASSGWATWPAAAAAAA |
| **14** | SCRYIGSMVPEVHR |
| **14** | TAPWGSGPKSDPMAILR |
| **14** | TAPWGSGPKSDPMAILR |
| **14** | QLTPGEAPCLSDVLQPAR |
| **14** | QLTPGEAPCLSDVLQPAR |
| **14** | VPVFIGSEDDVSMCCTHFR |
| **14** | VPVFIGSEDDVSMCCTHFR |
| **14** | TLLYGGVFIYPAHAGRPNGR |
| **14** | TLLMLNTLKEEPTLADGDPR |
| **14** | GVTLDEDIDGFVCAHLPTALR |
| **14** | TLLMLNTLKEEPTLADGDPR |
| **14** | STLGPPRGGAASSGWATWPAAAAAAA |
| **14** | EPGTGQYAVVFDPLTGLADPPTK |
| **14** | TLLYGGVFIYPAHAGRPNGRLK |
| **14** | GVTLDEDIDGFVCAHLPTALRSPR |
| **14** | SPTRGVTLDEDIDGFVCAHLPTALR |
| **14** | DMGAIFGVFRQLTPGEAPCLSDVLQPAR |
| **14** | DMGAIFGVFRQLTPGEAPCLSDVLQPAR |
| **14** | VPVFIGSEDDVSMCCTHFRTAPWGSGPK |
| **14** | VPVFIGSEDDVSMCCTHFRTAPWGSGPK |
| **14** | YIGSMVPEVHRTLLYGGVFIYPAHAGRPNGR |
| **14** | EECEEPIIVKEPGTGQYAVVFDPLTGLADPPTK |
| **14** | YIGSMVPEVHRTLLYGGVFIYPAHAGRPNGR |
| **14** | EPGTGQYAVVFDPLTGLADPPTKDMGAIFGVFR |
| **14** | EPGTGQYAVVFDPLTGLADPPTKDMGAIFGVFR |
| **14** | EECEEPIIVKEPGTGQYAVVFDPLTGLADPPTK |
| **15** | ASAHMK |
| **15** | TVDGPSGK |
| **15** | LTGMAFR |
| **15** | LWRDGR |
| **15** | LTGMAFR |
| **15** | PFPGTVEAK |
| **15** | AITVHACK |
| **15** | VIPELNGK |
| **15** | DPATIPWK |
| **15** | DGKLVVEGK |
| **15** | PFPGTVEAK |
| **15** | ASAHMKGGATK |
| **15** | ASAHMKGGATK |
| **15** | NSTNYDEIK |
| **15** | PFPGTVEAKDGK |
| **15** | AVQEASETSMK |
| **15** | AVQEASETSMK |
| **15** | VIDLLNHMNK |
| **15** | NSTNYDEIKK |
| **15** | VIDLLNHMNK |
| **15** | TVDGPSGKLWR |
| **15** | AVGKVIPELNGK |
| **15** | PFPGTVEAKDGK |
| **15** | KAVQEASETSMK |
| **15** | KAVQEASETSMK |
| **15** | LKNSTNYDEIK |
| **15** | GAYQNIIPASTGAAK |
| **15** | LVVEGKAITVHACK |
| **15** | VPVPDVSVVDLTVR |
| **15** | SSIGAGIQLSPTFVK |
| **15** | VIDLLNHMNKTDTA |
| **15** | VIDLLNHMNKTDTA |
| **15** | VIPELNGKLTGMAFR |
| **15** | VIPELNGKLTGMAFR |
| **15** | VPVPDVSVVDLTVRLK |
| **15** | AITVHACKDPATIPWK |
| **15** | LVSWYDNEYGYSCR |
| **15** | DGRGAYQNIIPASTGAAK |
| **15** | GILGYTEDQVVSQDFK |
| **15** | GAYQNIIPASTGAAKAVGK |
| **15** | GDARSSIGAGIQLSPTFVK |
| **15** | SMNVVSNASCTTNCLAPLAK |
| **15** | SMNVVSNASCTTNCLAPLAK |
| **15** | FGIEEGLMTTIHAVTATQK |
| **15** | DDGAEYVVESTGVFTTTEK |
| **15** | FGIEEGLMTTIHAVTATQK |
| **15** | GILGYTEDQVVSQDFKGDAR |
| **15** | VVISAPSADAPMFVGVGNNEDK |
| **15** | VVISAPSADAPMFVGVGNNEDK |
| **15** | LTGMAFRVPVPDVSVVDLTVR |
| **15** | LTGMAFRVPVPDVSVVDLTVR |
| **15** | YQKSMNVVSNASCTTNCLAPLAK |
| **15** | YQKSMNVVSNASCTTNCLAPLAK |
| **15** | SMNVVSNASCTTNCLAPLAKVINDK |
| **15** | SMNVVSNASCTTNCLAPLAKVINDK |
| **15** | VINDKFGIEEGLMTTIHAVTATQK |
| **15** | GGATKVVISAPSADAPMFVGVGNNEDK |
| **15** | VINDKFGIEEGLMTTIHAVTATQK |
| **15** | VVISAPSADAPMFVGVGNNEDKYQK |
| **15** | GGATKVVISAPSADAPMFVGVGNNEDK |
| **15** | VVISAPSADAPMFVGVGNNEDKYQK |
| **15** | DDGAEYVVESTGVFTTTEKASAHMK |
| **15** | DDGAEYVVESTGVFTTTEKASAHMK |
| **15** | FGIEEGLMTTIHAVTATQKTVDGPSGK |
| **15** | FGIEEGLMTTIHAVTATQKTVDGPSGK |
| **15** | LVSWYDNEYGYSCRVIDLLNHMNK |
| **15** | LVSWYDNEYGYSCRVIDLLNHMNK |
| **15** | DPATIPWKDDGAEYVVESTGVFTTTEK |
| **15** | AVQEASETSMKGILGYTEDQVVSQDFK |
| **15** | AVQEASETSMKGILGYTEDQVVSQDFK |
| **15** | SSIGAGIQLSPTFVKLVSWYDNEYGYSCR |
| **16** | VCASGLK |
| **16** | GALGRTR |
| **16** | LPADAVR |
| **16** | TRLPADAVR |
| **16** | TPIGSFLGSLR |
| **16** | AVALAADSLALGR |
| **16** | GVPAVELAAVAIR |
| **16** | ASLPAATVCTTVNK |
| **16** | TAASTLHDVYIVSAAR |
| **16** | TAASTLHDVYIVSAAR |
| **16** | GVPAVELAAVAIRGALGR |
| **16** | ECVLGNVLGANAGQAPAR |
| **16** | AVALAADSLALGRGGGAGAAV |
| **16** | VCASGLKAVALAADSLALGR |
| **16** | VAALRASLPAATVCTTVNK |
| **16** | ASLPAATVCTTVNKVCASGLK |
| **16** | ECVLGNVLGANAGQAPARVAALR |
| **16** | TPIGSFLGSLRGVPAVELAAVAIR |
| **16** | LPADAVRECVLGNVLGANAGQAPAR |
| **16** | TAASTLHDVYIVSAARTPIGSFLGSLR |
| **16** | TAASTLHDVYIVSAARTPIGSFLGSLR |
| **17** | SDLAATV |
| **17** | VAQETR |
| **17** | NLPQIR |
| **17** | SPSPVRK |
| **17** | TLDVCVR |
| **17** | TALSHGLK |
| **17** | DAITSLCK |
| **17** | RNLPQIR |
| **17** | IRHYLAK |
| **17** | TFLVGGNWK |
| **17** | TKTALSHGLK |
| **17** | ETDDFIAQK |
| **17** | HYLAKDVNEK |
| **17** | ASFLEVIESFK |
| **17** | ELAAAPDLETDK |
| **17** | EEGRTLDVCVR |
| **17** | DVNEKVAQETR |
| **17** | ETDDFIAQKTK |
| **17** | CNLSKDAITSLCK |
| **17** | VMFCIGETLQER |
| **17** | VMFCIGETLQER |
| **17** | VATPDQVEEVHEK |
| **17** | AIACTCSCPSPFPPR |
| **17** | TFLVGGNWKCNLSK |
| **17** | DVGCTWVILGHSER |
| **17** | QLHALVETIEAGDWK |
| **17** | QLHALVETIEAGDWK |
| **17** | DVVIAYEPVWAIGTGK |
| **17** | DVGCTWVILGHSERR |
| **17** | VATPDQVEEVHEKIR |
| **17** | NLPQIRETDDFIAQK |
| **17** | VEVVLAPPTPYLDHTR |
| **17** | CRAIACTCSCPSPFPPR |
| **17** | VMFCIGETLQEREEGR |
| **17** | VMFCIGETLQEREEGR |
| **17** | ASFLEVIESFKSDLAATV |
| **17** | DAITSLCKELAAAPDLETDK |
| **17** | AIACTCSCPSPFPPRSPSPVR |
| **17** | TALSHGLKVMFCIGETLQER |
| **17** | SPTPPRPSHILLSCEIGHDER |
| **17** | TALSHGLKVMFCIGETLQER |
| **17** | VEVVLAPPTPYLDHTRSVLR |
| **17** | SPTPPRPSHILLSCEIGHDER |
| **17** | SPTPPRPSHILLSCEIGHDERSR |
| **17** | TLDVCVRQLHALVETIEAGDWK |
| **17** | SPTPPRPSHILLSCEIGHDERSR |
| **17** | AFTGAAVCPVPAGPAAPTAATWSMSAGGLR |
| **17** | AFTGAAVCPVPAGPAAPTAATWSMSAGGLR |
| **17** | RAFTGAAVCPVPAGPAAPTAATWSMSAGGLR |
| **17** | QDFEVAAQNIWVGGPGAFTGETVAEMIK |
| **17** | RAFTGAAVCPVPAGPAAPTAATWSMSAGGLR |
| **17** | QDFEVAAQNIWVGGPGAFTGETVAEMIK |
| **17** | QDFEVAAQNIWVGGPGAFTGETVAEMIK |
| **17** | QDFEVAAQNIWVGGPGAFTGETVAEMIK |
| **17** | ELAAAPDLETDKVEVVLAPPTPYLDHTR |
| **17** | DVVIAYEPVWAIGTGKVATPDQVEEVHEK |
| **17** | ILYGGSVSPGNCNELAQLPDVDGFLVGGASLK |
| **17** | QLHALVETIEAGDWKDVVIAYEPVWAIGTGK |
| **17** | QLHALVETIEAGDWKDVVIAYEPVWAIGTGK |
| **17** | SVLRQDFEVAAQNIWVGGPGAFTGETVAEMIK |
| **17** | SVLRQDFEVAAQNIWVGGPGAFTGETVAEMIK |
| **17** | AFTGAAVCPVPAGPAAPTAATWSMSAGGLRTFLVGGNWK |
| **17** | AFTGAAVCPVPAGPAAPTAATWSMSAGGLRTFLVGGNWK |
| **18** | VAAGGGAR |
| **18** | TKYGTK |
| **18** | FTFPGR |
| **18** | YTSLER |
| **18** | IRAYTR |
| **18** | TVGFDGAR |
| **18** | SKPSGPPR |
| **18** | ELADWGR |
| **18** | QLIGWTR |
| **18** | QLIGWTR |
| **18** | FTFPGRR |
| **18** | QVIDVLLR |
| **18** | DSWLPAVR |
| **18** | MRVAAGGGAR |
| **18** | QVIDVLLR |
| **18** | MRVAAGGGAR |
| **18** | SKPSGPPRR |
| **18** | FVDALGHVK |
| **18** | RELADWGR |
| **18** | HMDARFFR |
| **18** | YSAYEWDR |
| **18** | HMDARFFR |
| **18** | QLIGWTRAGR |
| **18** | LDAVKHMDAR |
| **18** | QLIGWTRAGR |
| **18** | LDAVKHMDAR |
| **18** | QVIDVLLRLR |
| **18** | AYTRFTFPGR |
| **18** | QVIDVLLRLR |
| **18** | SFDKYTSLER |
| **18** | ALAVVLSSGPGGAR |
| **18** | GKYSAYEWDR |
| **18** | TVGFDGARLDAVK |
| **18** | FVDALGHVKGEVT |
| **18** | FFRDSWLPAVR |
| **18** | ALAVVLSSGPGGARR |
| **18** | WLFRTVGFDGAR |
| **18** | GKALAVVLSSGPGGAR |
| **18** | ELADWGRWLFR |
| **18** | NGATQQVITLPSHR |
| **18** | VAAMASAADQGNTTAR |
| **18** | VAAMASAADQGNTTAR |
| **18** | VAAGGGARFVDALGHVK |
| **18** | DHAYGEEVDFFGGGSR |
| **18** | GGYPAVFHADYYGGITR |
| **18** | RDHAYGEEVDFFGGGSR |
| **18** | VGADGTEEVTATPYAANDR |
| **18** | ADAPRVAAMASAADQGNTTAR |
| **18** | ADAPRVAAMASAADQGNTTAR |
| **18** | RGGYPAVFHADYYGGITR |
| **18** | ADAPRVAAMASAADQGNTTAR |
| **18** | ADAPRVAAMASAADQGNTTAR |
| **18** | GNYDYLLGADTDTDAEWVR |
| **18** | GNYDYLLGADTDTDAEWVRR |
| **18** | QLAVDAVDLAAAGVTAVWLPPPYK |
| **18** | NGATQQVITLPSHRQVIDVLLR |
| **18** | QLAVDAVDLAAAGVTAVWLPPPYK |
| **18** | GSFDAVDYDANLGGAGGTVYLLEGK |
| **18** | GDAGAADVGYGVYDTYDLGEFDQK |
| **18** | DHAYGEEVDFFGGGSRQLIGWTR |
| **18** | AELVSAVSAAHDAGVQVYADVVLNHR |
| **18** | VGADGTEEVTATPYAANDRSKPSGPPR |
| **18** | YTSLERGNYDYLLGADTDTDAEWVR |
| **18** | GDAGAADVGYGVYDTYDLGEFDQKGSVR |
| **18** | GSFDAVDYDANLGGAGGTVYLLEGKSFDK |
| **18** | AAQVAEGNGVLFQYFYWDLPADGGLWR |
| **18** | YGTKAELVSAVSAAHDAGVQVYADVVLNHR |
| **18** | GGYPAVFHADYYGGITRNGATQQVITLPSHR |
| **18** | YSAYEWDRGSFDAVDYDANLGGAGGTVYLLEGK |
| **19** | LHLTSR |
| **19** | TDSFDR |
| **19** | VSDSALR |
| **19** | LKRPDK |
| **19** | ENRPVR |
| **19** | AYEARAK |
| **19** | GFLGTPVK |
| **19** | LMIGFQK |
| **19** | YDADFGR |
| **19** | LMIGFQK |
| **19** | ISEIDPSLG |
| **19** | NLFYGDSK |
| **19** | LMIGFQKR |
| **19** | RYDADFGR |
| **19** | LMIGFQKR |
| **19** | VHVGIIGCGR |
| **19** | AKGFLGTPVK |
| **19** | ATPYGYDQK |
| **19** | MYKPDHEAGK |
| **19** | MYKPDHEAGK |
| **19** | RPDKSTLTR |
| **19** | MYKPDHEAGK |
| **19** | MYKPDHEAGK |
| **19** | SIKENRPVR |
| **19** | LEDSVSEEAK |
| **19** | DPAPPPVGYLK |
| **19** | IPVVYAMAATK |
| **19** | MYKPDHEAGK |
| **19** | IPVVYAMAATK |
| **19** | MYKPDHEAGK |
| **19** | HIFCEKPIDK |
| **19** | IGQCHAANLANK |
| **19** | MYKPDHEAGKLK |
| **19** | STLTRTDSFDR |
| **19** | MYKPDHEAGKLK |
| **19** | NLFYGDSKLHK |
| **19** | MYKPDHEAGKLK |
| **19** | MYKPDHEAGKLK |
| **19** | VMVQQSAESFTK |
| **19** | FANGCIGTIDNSR |
| **19** | VMVQQSAESFTK |
| **19** | QYNVPMACTDYK |
| **19** | MYKPDHEAGKLK |
| **19** | QYNVPMACTDYK |
| **19** | QYNVPMACTDYK |
| **19** | MYKPDHEAGKLK |
| **19** | YDADFGRAYEAR |
| **19** | QYNVPMACTDYK |
| **19** | IPVVYAMAATKSIK |
| **19** | IPVVYAMAATKSIK |
| **19** | GFLGTPVKLHLTSR |
| **19** | TDSFDRNLFYGDSK |
| **19** | ENRPVRISEIDPSLG |
| **19** | LHKVMVQQSAESFTK |
| **19** | LAKQYNVPMACTDYK |
| **19** | LHKVMVQQSAESFTK |
| **19** | LAKQYNVPMACTDYK |
| **19** | TGCHTDLPVYFFMQR |
| **19** | EAAAAGKHIFCEKPIDK |
| **19** | TGCHTDLPVYFFMQR |
| **19** | TLSVIDESLAAVEEAGVK |
| **19** | VSDSALRLEDSVSEEAK |
| **19** | LHLTSRDPAPPPVGYLK |
| **19** | VPDAELVCVSDFFEESAR |
| **19** | NSGGVFLDQTIHDFDMAR |
| **19** | NSGGVFLDQTIHDFDMAR |
| **19** | VMVQQSAESFTKVSDSALR |
| **19** | LEDSVSEEAKVHVGIIGCGR |
| **19** | VMVQQSAESFTKVSDSALR |
| **19** | VPDAELVCVSDFFEESARR |
| **19** | VHVGIIGCGRIGQCHAANLANK |
| **19** | FANGCIGTIDNSRATPYGYDQK |
| **19** | TLSVIDESLAAVEEAGVKLMIGFQK |
| **19** | TLSVIDESLAAVEEAGVKLMIGFQK |
| **19** | AEFFGTSGAITVNNNFPNTATYADR |
| **19** | DLINNADVHAIIVCSPTDTHADIIK |
| **19** | HIFCEKPIDKTLSVIDESLAAVEEAGVK |
| **19** | DPAPPPVGYLKNSGGVFLDQTIHDFDMAR |
| **19** | DPAPPPVGYLKNSGGVFLDQTIHDFDMAR |
| **19** | IGQCHAANLANKVPDAELVCVSDFFEESAR |
| **19** | FASAFLMEMIAFIDCIVQDTPVPCTGNDGR |
| **19** | FASAFLMEMIAFIDCIVQDTPVPCTGNDGR |
| **19** | DLINNADVHAIIVCSPTDTHADIIKEAAAAGK |
| **19** | FASAFLMEMIAFIDCIVQDTPVPCTGNDGR |
| **19** | ATPYGYDQKAEFFGTSGAITVNNNFPNTATYADR |
| **19** | YLVGSDVVEIYATGLAVNPEIAALGDYDNTICHLK |
| **20** | LHASAR |
| **20** | HVIGLK |
| **20** | NAAIYR |
| **20** | ALAEVAR |
| **20** | LSGFPAGR |
| **20** | QRPGESR |
| **20** | QRPGESR |
| **20** | GVGSLRGAK |
| **20** | VVPVSVAAK |
| **20** | GGVERVLR |
| **20** | SILPPLVAR |
| **20** | RVVPVSVAAK |
| **20** | VPLSEAEHTK |
| **20** | ALAEVARSVEA |
| **20** | VALASELGVAPK |
| **20** | VVGAAKHVIGLK |
| **20** | VFGSGTYLDSSR |
| **20** | MTDGAWAALHAR |
| **20** | MTDGAWAALHAR |
| **20** | LALVARNAAIYR |
| **20** | LHASARALAEVAR |
| **20** | LRVALASELGVAPK |
| **20** | QRPGESRLALVAR |
| **20** | QRPGESRLALVAR |
| **20** | VLRVPLSEAEHTK |
| **20** | VFGSGTYLDSSRLR |
| **20** | NAAIYRSILPPLVAR |
| **20** | VPLSEAEHTKLHASAR |
| **20** | MTDGAWAALHARVVGAAK |
| **20** | MTDGAWAALHARVVGAAK |
| **20** | FADIRMTDGAWAALHAR |
| **20** | FADIRMTDGAWAALHAR |
| **20** | GYTNWGVGAAVGALVGFILR |
| **20** | GTYGIEEDVFLSLPAVLGR |
| **20** | LSGFPAGRVFGSGTYLDSSR |
| **20** | AAAPNYDGSEASDVIIITAGAR |
| **20** | VEGEVLDFTHGGAFYHANVK |
| **20** | SPDAILLIVSNPVDILTAIAAR |
| **20** | KVEGEVLDFTHGGAFYHANVK |
| **20** | GYTNWGVGAAVGALVGFILRDEK |
| **20** | GTYGIEEDVFLSLPAVLGRGGVER |
| **20** | SVHASILGEHGDSSVAVASMANVGGAR |
| **20** | SVHASILGEHGDSSVAVASMANVGGAR |
| **20** | HVIGLKGYTNWGVGAAVGALVGFILR |
| **20** | GGGNDSGGSSGGIAGSLVDELCFPSVGGGGDR |
| **20** | GGGNDSGGSSGGIAGSLVDELCFPSVGGGGDR |
| **20** | VVPVSVAAKGTYGIEEDVFLSLPAVLGR |
| **20** | AAAPNYDGSEASDVIIITAGARQRPGESR |
| **20** | SPDAILLIVSNPVDILTAIAARLSGFPAGR |
| **20** | SVHASILGEHGDSSVAVASMANVGGARFADIR |
| **20** | SVHASILGEHGDSSVAVASMANVGGARFADIR |
| **20** | SILPPLVARSPDAILLIVSNPVDILTAIAAR |
| **20** | VTIVGCGSVGMACASAILSTGLASTLVFADVDAK |
| **20** | VTIVGCGSVGMACASAILSTGLASTLVFADVDAK |
| **20** | GGGNDSGGSSGGIAGSLVDELCFPSVGGGGDRGVGSLR |
| **20** | VTIVGCGSVGMACASAILSTGLASTLVFADVDAKK |
| **20** | VTIVGCGSVGMACASAILSTGLASTLVFADVDAKK |
| **20** | GGGNDSGGSSGGIAGSLVDELCFPSVGGGGDRGVGSLR |
| **20** | GAKVTIVGCGSVGMACASAILSTGLASTLVFADVDAK |
| **20** | GAKVTIVGCGSVGMACASAILSTGLASTLVFADVDAK |
| **20** | VALASELGVAPKSVHASILGEHGDSSVAVASMANVGGAR |
| **20** | VALASELGVAPKSVHASILGEHGDSSVAVASMANVGGAR |
| **21** | DTGDTR |
| **21** | VVKACR |
| **21** | LNTNFC |
| **21** | AVGMDSR |
| **21** | AVGMDSR |
| **21** | KVGLGAGR |
| **21** | KDTGDTR |
| **21** | ERFVSR |
| **21** | IGPKFLK |
| **21** | RLIEER |
| **21** | IASVAESPK |
| **21** | IALLGFAFK |
| **21** | ESAAIEICR |
| **21** | ASVCIYDPK |
| **21** | LDRTELEK |
| **21** | NVLDHAALR |
| **21** | RIASVAESPK |
| **21** | MVSNPVQTGLR |
| **21** | MVSNPVQTGLR |
| **21** | IALLGFAFKK |
| **21** | MVSNPVQTGLR |
| **21** | MVSNPVQTGLR |
| **21** | LVANSFLAQR |
| **21** | AVGMDSRIGPK |
| **21** | RIALLGFAFK |
| **21** | NVLDHAALRK |
| **21** | AVGMDSRIGPK |
| **21** | ESAAIEICRR |
| **21** | ASVGFGGSCFQK |
| **21** | VVVEKSTVPIK |
| **21** | MVSNPVQTGLR |
| **21** | MVSNPVQTGLR |
| **21** | IIHGLFNTVTGK |
| **21** | LADVYAHWVSR |
| **21** | TAEAISAVLHGAGK |
| **21** | AADLTYWELAAR |
| **21** | IASVAESPKVVVEK |
| **21** | IIHGLFNTVTGKR |
| **21** | ILTTNVWSSELSK |
| **21** | DADVIFVAVNTPTK |
| **21** | NLFFSTDVDQGIR |
| **21** | AADLTYWELAARR |
| **21** | FLKASVGFGGSCFQK |
| **21** | TAEAISAVLHGAGKTR |
| **21** | LADVYAHWVSRER |
| **21** | DADVIFVAVNTPTKK |
| **21** | CPEVTVTIVDISVPR |
| **21** | LIEERASVCIYDPK |
| **21** | DTGDTRESAAIEICR |
| **21** | LGYIVYAIGKPLDPK |
| **21** | GKNLFFSTDVDQGIR |
| **21** | VLIGGNMNESGQAAVSR |
| **21** | IQDGMVKPCFVFDGR |
| **21** | VLIGGNMNESGQAAVSR |
| **21** | VEANQIWMDLTAATR |
| **21** | IQDGMVKPCFVFDGR |
| **21** | VEANQIWMDLTAATR |
| **21** | ERILTTNVWSSELSK |
| **21** | KLGYIVYAIGKPLDPK |
| **21** | FVSRIIHGLFNTVTGK |
| **21** | STVPIKTAEAISAVLHGAGK |
| **21** | ICCIGAGYVGGPTMAMMALK |
| **21** | VGLGAGRAADLTYWELAAR |
| **21** | ICCIGAGYVGGPTMAMMALK |
| **21** | ICCIGAGYVGGPTMAMMALK |
| **21** | IAAWNSDELPIYEPGLDK |
| **21** | ICCIGAGYVGGPTMAMMALK |
| **21** | VEANQIWMDLTAATRLDR |
| **21** | VEANQIWMDLTAATRLDR |
| **21** | VSSINAVSAICEASGADVDEVAR |
| **21** | LGYIVYAIGKPLDPKLNTNFC |
| **21** | GLDFAKIQDGMVKPCFVFDGR |
| **21** | IAAWNSDELPIYEPGLDKVVK |
| **21** | GLDFAKIQDGMVKPCFVFDGR |
| **21** | ILTTNVWSSELSKLVANSFLAQR |
| **21** | FDVLSNPEFLAEGTAMTDLDNPDR |
| **21** | FDVLSNPEFLAEGTAMTDLDNPDR |
| **21** | ASVCIYDPKVEANQIWMDLTAATR |
| **21** | IQDGMVKPCFVFDGRNVLDHAALR |
| **21** | ASVCIYDPKVEANQIWMDLTAATR |
| **21** | IQDGMVKPCFVFDGRNVLDHAALR |
| **21** | TRFDVLSNPEFLAEGTAMTDLDNPDR |
| **21** | TRFDVLSNPEFLAEGTAMTDLDNPDR |
| **21** | VSSINAVSAICEASGADVDEVARAVGMDSR |
| **21** | NLFFSTDVDQGIRDADVIFVAVNTPTK |
| **21** | VSSINAVSAICEASGADVDEVARAVGMDSR |
| **21** | VLIGGNMNESGQAAVSRLADVYAHWVSR |
| **21** | YVTLCDSAYEAAAGAHALAVATEWDEFK |
| **21** | VLIGGNMNESGQAAVSRLADVYAHWVSR |
| **21** | MVSNPVQTGLRICCIGAGYVGGPTMAMMALK |
| **21** | MVSNPVQTGLRICCIGAGYVGGPTMAMMALK |
| **21** | MVSNPVQTGLRICCIGAGYVGGPTMAMMALK |
| **21** | MVSNPVQTGLRICCIGAGYVGGPTMAMMALK |
| **21** | MVSNPVQTGLRICCIGAGYVGGPTMAMMALK |
| **21** | MVSNPVQTGLRICCIGAGYVGGPTMAMMALK |
| **21** | MVSNPVQTGLRICCIGAGYVGGPTMAMMALK |
| **21** | MVSNPVQTGLRICCIGAGYVGGPTMAMMALK |
| **21** | MVSNPVQTGLRICCIGAGYVGGPTMAMMALK |
| **21** | MVSNPVQTGLRICCIGAGYVGGPTMAMMALK |
| **21** | MVSNPVQTGLRICCIGAGYVGGPTMAMMALK |
| **21** | MVSNPVQTGLRICCIGAGYVGGPTMAMMALK |
| **21** | MVSNPVQTGLRICCIGAGYVGGPTMAMMALK |
| **21** | MVSNPVQTGLRICCIGAGYVGGPTMAMMALK |
| **21** | MVSNPVQTGLRICCIGAGYVGGPTMAMMALK |
| **21** | MVSNPVQTGLRICCIGAGYVGGPTMAMMALK |
| **21** | MVSNPVQTGLRICCIGAGYVGGPTMAMMALK |
| **21** | MVSNPVQTGLRICCIGAGYVGGPTMAMMALK |
| **21** | LVANSFLAQRVSSINAVSAICEASGADVDEVAR |
| **21** | DILNLVYLCQSMGLPDVAEYFHSVVTMNDR |
| **21** | DILNLVYLCQSMGLPDVAEYFHSVVTMNDR |
| **21** | DILNLVYLCQSMGLPDVAEYFHSVVTMNDR |
| **21** | ICCIGAGYVGGPTMAMMALKCPEVTVTIVDISVPR |
| **21** | TELEKYVTLCDSAYEAAAGAHALAVATEWDEFK |
| **21** | ICCIGAGYVGGPTMAMMALKCPEVTVTIVDISVPR |
| **21** | ICCIGAGYVGGPTMAMMALKCPEVTVTIVDISVPR |
| **21** | YVTLCDSAYEAAAGAHALAVATEWDEFKGLDFAK |
| **21** | CPEVTVTIVDISVPRIAAWNSDELPIYEPGLDK |
| **21** | ICCIGAGYVGGPTMAMMALKCPEVTVTIVDISVPR |
| **21** | DILNLVYLCQSMGLPDVAEYFHSVVTMNDRQK |
| **21** | DILNLVYLCQSMGLPDVAEYFHSVVTMNDRQK |
| **21** | DILNLVYLCQSMGLPDVAEYFHSVVTMNDRQK |
| **22** | VLIKAK |
| **22** | LTESLR |
| **22** | VESMVAK |
| **22** | VYPAASR |
| **22** | VESMVAK |
| **22** | AVRAAWAR |
| **22** | FTAYNAR |
| **22** | DVIDVVAK |
| **22** | VVLLHGCR |
| **22** | AVRAAWAR |
| **22** | LTESLRVR |
| **22** | VESMVAKHR |
| **22** | ARFTAYNAR |
| **22** | VESMVAKHR |
| **22** | AAWARADGDR |
| **22** | ADGDRSGGLTR |
| **22** | SGGLTRAEVLR |
| **22** | NVTRVYPAASR |
| **22** | AEVLRLTESLR |
| **22** | FTAYNARNVTR |
| **22** | VPFRDVIDVVAK |
| **22** | LTFPQFVTFFR |
| **22** | MATHLTAGLGELLR |
| **22** | MATHLTAGLGELLR |
| **22** | LTFPQFVTFFRR |
| **22** | AVAVELASLVTLGGGSR |
| **22** | VKAVAVELASLVTLGGGSR |
| **22** | GTAAHPPAAACGFLTVHIL |
| **22** | LPQPADVDALPTLGSLLGK |
| **22** | FLANNACGYVPRPALTVR |
| **22** | LPQPADVDALPTLGSLLGKVLIK |
| **22** | YAFVASTAPVILSLENHCCLLQQVR |
| **22** | CVEIDVWDGDEGEPVVYHGHTLTSK |
| **22** | ADISAAAAAAAAAAVSSDDDSFSSSDLGAGPR |
| **22** | AAVQAAFEGGDTHPPGQPVTDVCSFNETK |
| **22** | VDKADISAAAAAAAAAAVSSDDDSFSSSDLGAGPR |
| **22** | CVEIDVWDGDEGEPVVYHGHTLTSKVPFR |
| **22** | MATHLTAGLGELLRLPQPADVDALPTLGSLLGK |
| **22** | MATHLTAGLGELLRLPQPADVDALPTLGSLLGK |
| **22** | DVIDVVAKYAFVASTAPVILSLENHCCLLQQVR |
| **22** | VVLLHGCRCVEIDVWDGDEGEPVVYHGHTLTSK |
| **22** | AAVQAAFEGGDTHPPGQPVTDVCSFNETKVESMVAK |
| **22** | AAVQAAFEGGDTHPPGQPVTDVCSFNETKVESMVAK |
| **22** | FLANNACGYVPRPALTVRGTAAHPPAAACGFLTVHIL |
| **23** | VTAVHK |
| **23** | NLVAIK |
| **23** | LLRAAR |
| **23** | IITEVR |
| **23** | KVTAVHK |
| **23** | VLEGNGNK |
| **23** | GPFTTPVK |
| **23** | SAVAASQGLR |
| **23** | TLSPTMQSTR |
| **23** | TLSPTMQSTR |
| **23** | GPFTTPVKSK |
| **23** | YTSINLLLR |
| **23** | LGVDVVVMIR |
| **23** | IITEVRSMR |
| **23** | LGVDVVVMIR |
| **23** | IITEVRSMR |
| **23** | TLSPTMQSTR |
| **23** | TLSPTMQSTR |
| **23** | SFQKNLVAIK |
| **23** | SADGLFLDCCK |
| **23** | HADAIESAVMR |
| **23** | VTAVHKANIMK |
| **23** | HADAIESAVMR |
| **23** | VTAVHKANIMK |
| **23** | YTSINLLLRR |
| **23** | AARSAVAASQGLR |
| **23** | SKYTSINLLLR |
| **23** | SADGLFLDCCKR |
| **23** | SRLGVDVVVMIR |
| **23** | SRLGVDVVVMIR |
| **23** | TLSPTMQSTRLLR |
| **23** | TLSPTMQSTRLLR |
| **23** | NLVAIKGPFTTPVK |
| **23** | TLSPTMQSTRLLR |
| **23** | TLSPTMQSTRLLR |
| **23** | IAQYAFAYAAANNR |
| **23** | FSNYEDGLPQEVIK |
| **23** | IAQYAFAYAAANNRK |
| **23** | ANIMKSADGLFLDCCK |
| **23** | ANIMKSADGLFLDCCK |
| **23** | LGLGLGGGLGLQLTQLDK |
| **23** | ATPDIGSATTTDYTYTK |
| **23** | KLGLGLGGGLGLQLTQLDK |
| **23** | DFDLFANVHAYSIPGVK |
| **23** | SMRIAQYAFAYAAANNR |
| **23** | SMRIAQYAFAYAAANNR |
| **23** | ATPDIGSATTTDYTYTKAI |
| **23** | HADAIESAVMRVLEGNGNK |
| **23** | HADAIESAVMRVLEGNGNK |
| **23** | RDFDLFANVHAYSIPGVK |
| **23** | FSNYEDGLPQEVIKSFQK |
| **23** | HGSLAGGALLPTLEVTAAAGAPR |
| **23** | DFDLFANVHAYSIPGVKSR |
| **23** | ENTEGEYSGMEHEAVPGVVESLK |
| **23** | ENTEGEYSGMEHEAVPGVVESLK |
| **23** | VLEGNGNKATPDIGSATTTDYTYTK |
| **23** | LGLGLGGGLGLQLTQLDKHADAIESAVMR |
| **23** | LGLGLGGGLGLQLTQLDKHADAIESAVMR |
| **23** | SAVAASQGLRHGSLAGGALLPTLEVTAAAGAPR |
| **23** | ENTEGEYSGMEHEAVPGVVESLKIITEVR |
| **23** | ENTEGEYSGMEHEAVPGVVESLKIITEVR |
| **23** | LGVDVVVMIRENTEGEYSGMEHEAVPGVVESLK |
| **23** | LGVDVVVMIRENTEGEYSGMEHEAVPGVVESLK |
| **23** | LGVDVVVMIRENTEGEYSGMEHEAVPGVVESLK |
